# Supplementary material for: Starship giant transposons dominate plastic genomic regions in a fungal plant pathogen and drive virulence evolution
Source: Nat Commun. 2025 Jul 24;16:6806. doi: 10.1038/s41467-025-61986-6 (PMC12289983; doi:10.1038/s41467-025-61986-6)
Supplement: Supplementary file 1 — Supplementary Information [file 41467_2025_61986_MOESM1_ESM.pdf]

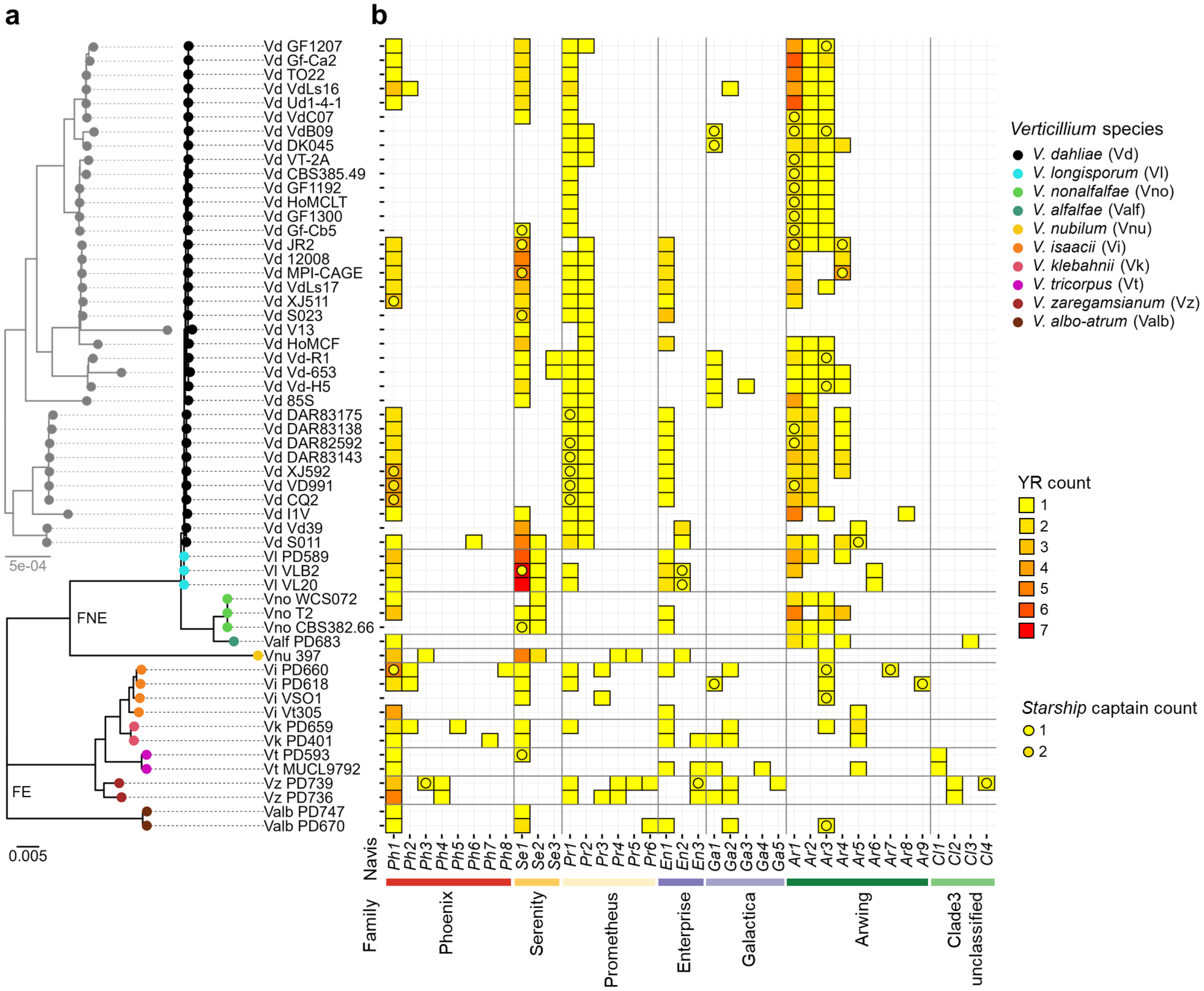

Figure S1. Differential captain and captain-like tyrosine recombinase (YR) repertoires across the *Verticillium* genus. a, *Verticillium* phylogeny as described in Fig. 1a. **b**, Repertoires of YR naves per strain grouped by amino acid sequence similarity and named according to the YR family to which they belong. Heatmap colors and values indicate YR gene counts.

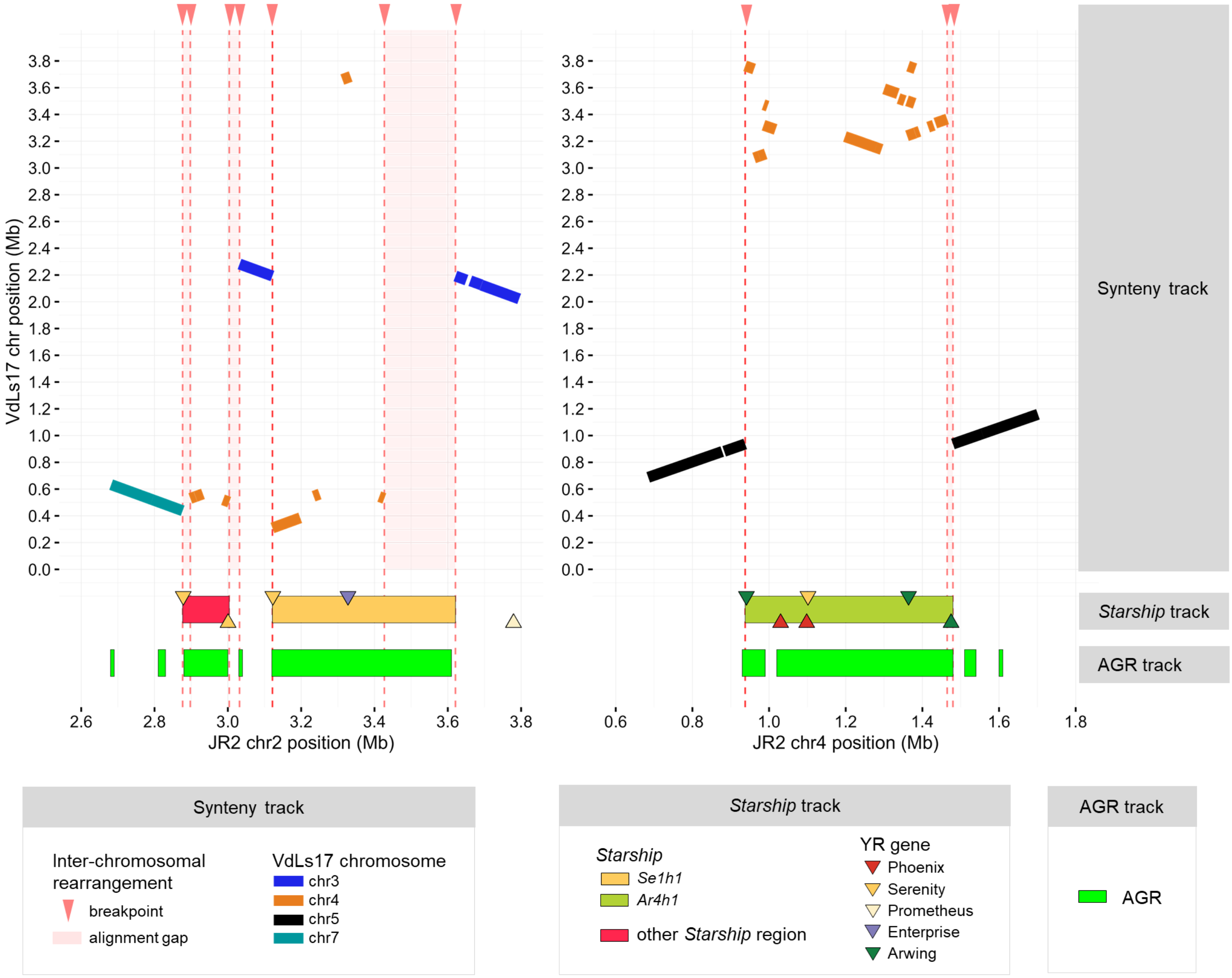

Figure S2. Correspondence between *Starship* boundaries and inter-chromosomal rearrangement breakpoints. Diagonal lines indicate the synteny between the chromosomes of *V. dahliae* strain VdLs17 (Y-axis) and each *Starship* insertion site of *V. dahliae* strain JR2 (X-axis) with the color representing syntenic VdLs17 chromosome. Bars and triangles aligned to the plots indicate the positions of *Starships*, captain/captain-like tyrosine recombinase (YR) genes, and adaptive genomic regions (AGRs) in the strain JR2.

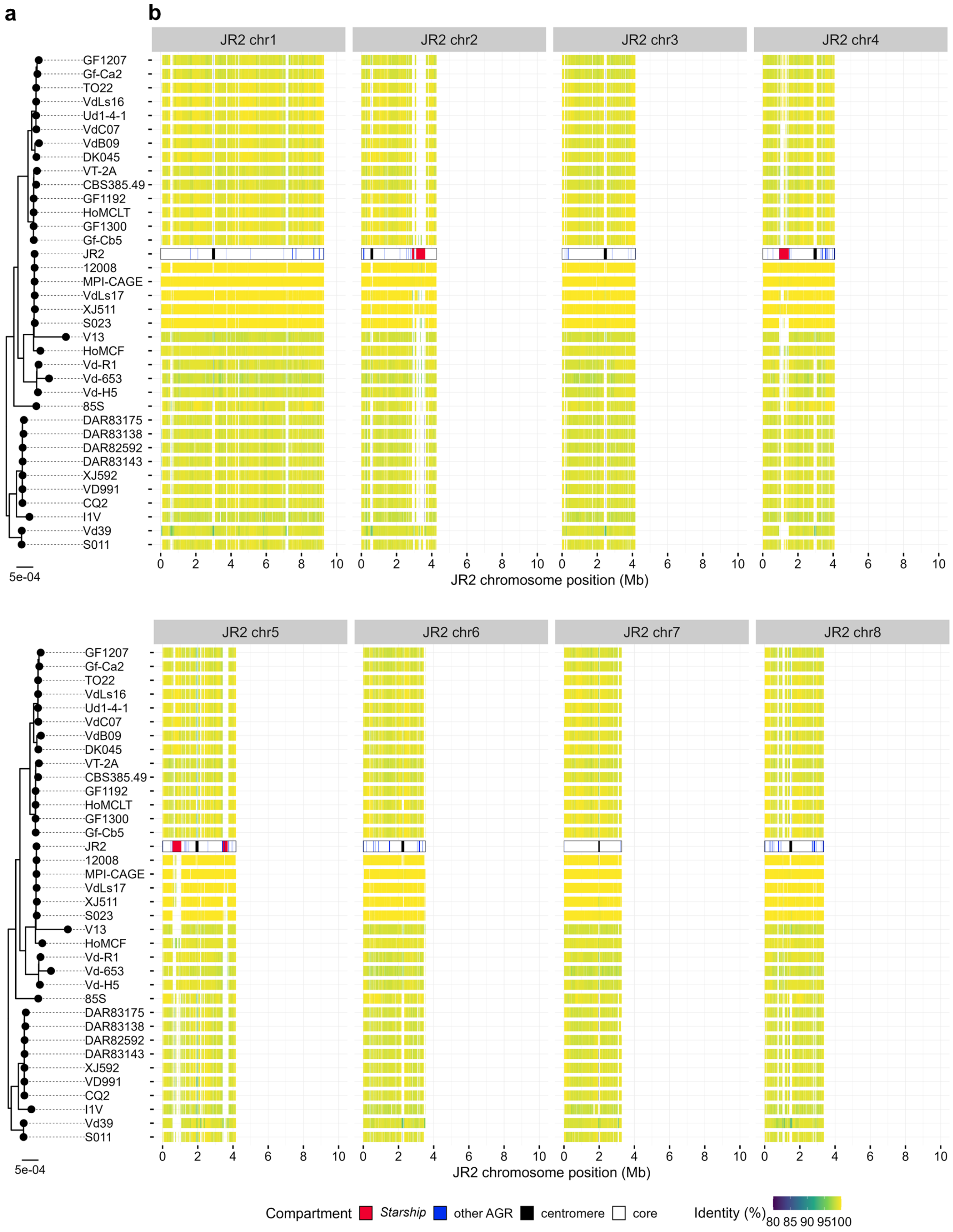

Figure S3. Alignment of 35 *V. dahliae* genomes to the *V. dahliae* JR2 genome. a, *Verticillium* phylogeny as described in Fig. 1a. **b**, Coverage plots of pairwise sequence alignments of 35 *V. dahliae* genomes to the JR2

genome in (**a**). Bar colors in JR2 represent the four genomic compartments "*Starship*", "other adaptive genomic regions (AGR)", "centromere", and "other core". Bar colors for the 35 *V. dahliae* strains represent nucleotide identities of syntenic regions.

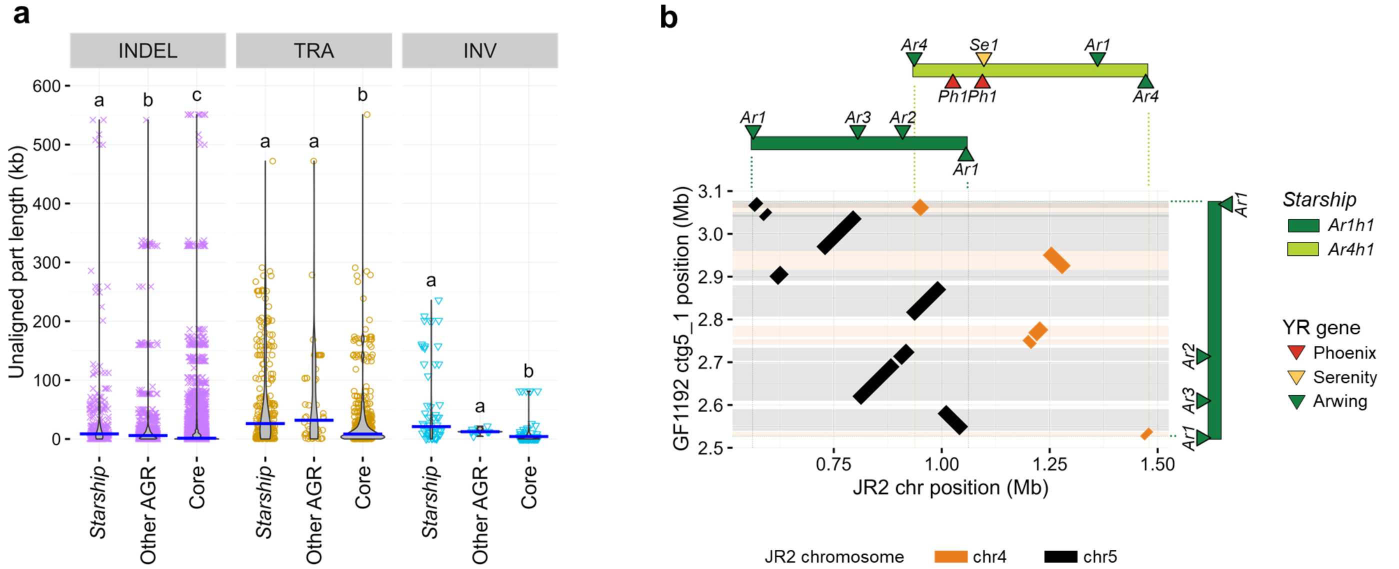

Figure S4. ***Starships*** **are hotspots of large-scale genomic rearrangements. a**, Violin plots depicting length of unaligned regions accompanied by each SV (insertion and deletion (INDEL), translocation (TRA), inversion (INV) in the JR2 genomic compartments. Points indicate the length of every gap (INDEL: n = 320 in *Starship* regions, n = 1102 in other adaptive genomic regions (AGRs), and n = 8237 in core regions; TRA: n = 260 in *Starship* regions, n = 50 in other AGRs, and n = 549 in core regions; INV: n = 64 in *Starship* regions, n = 11 in other AGRs, and n = 91 in core regions) found between JR2 genome and 35 *V. dahliae* genomes, while blue crossbars indicate the median values. Different letter labels indicate significant differences (two-sided Dunn's test, adjusted $p < 0.05$). **b**, *Starship* rearrangements between the genomes of *V. dahliae* strains JR2 (X-axis) and GF1192 (Y-axis). Diagonal lines indicate synteny colored by syntenic JR2 chromosome. Bars and triangles aligned to the plots indicate the positions of *Starships* and captain/captain-like tyrosine recombinase (YR) genes.

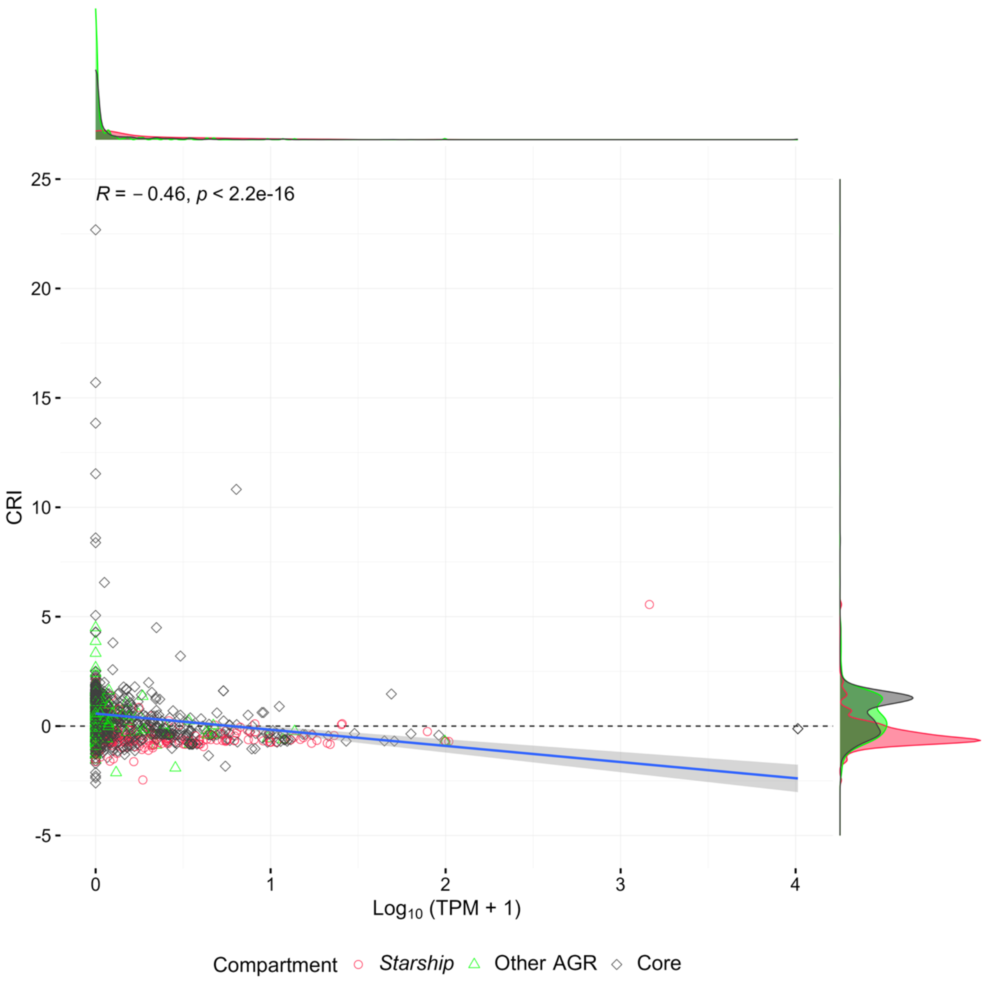

Figure S5. Correlation between the expression level of transposable elements (TEs) and their repeat-induced point mutation (RIP) signature. The X-axis indicates the expression level (in transcripts per million, TPM) for *V. dahliae* cultivated in potato dextrose broth, while the Y-axis indicates the composite RIP index (CRI). Points indicate values for individual TEs in the three JR2 genomic compartments (n = 284 in *Starship* regions, n = 202 in other AGRs, and n = 1261 in core regions). R indicates the Spearman's rank correlation coefficient.

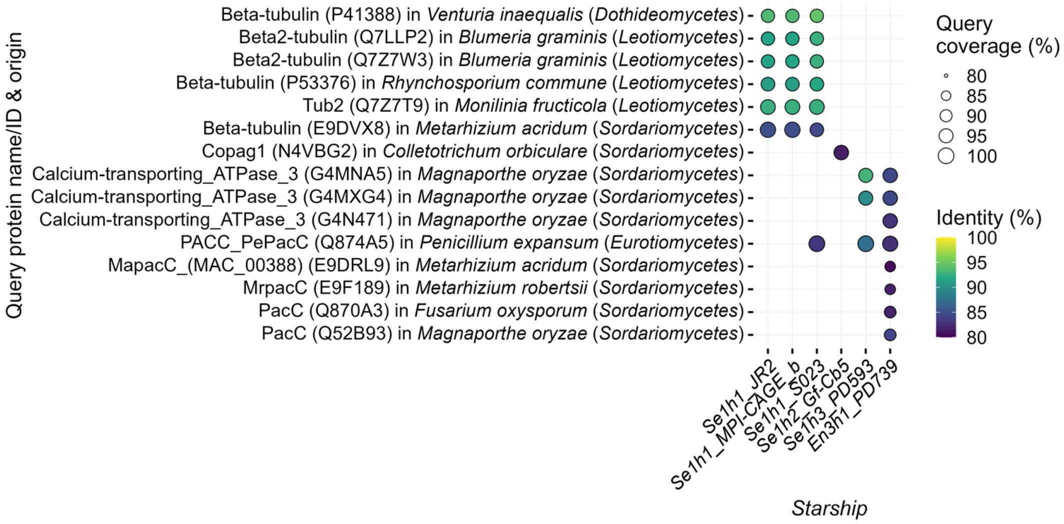

Figure S6. *Verticillium Starship* cargo elements orthologous to virulence-associated genes of other fungi. Hits (coverage and identity >80%) in the similarity search of *Verticillium Starships* with other fungal proteins that are described as virulence-associated in the Pathogen-Host Interactions Database (PHI-base).

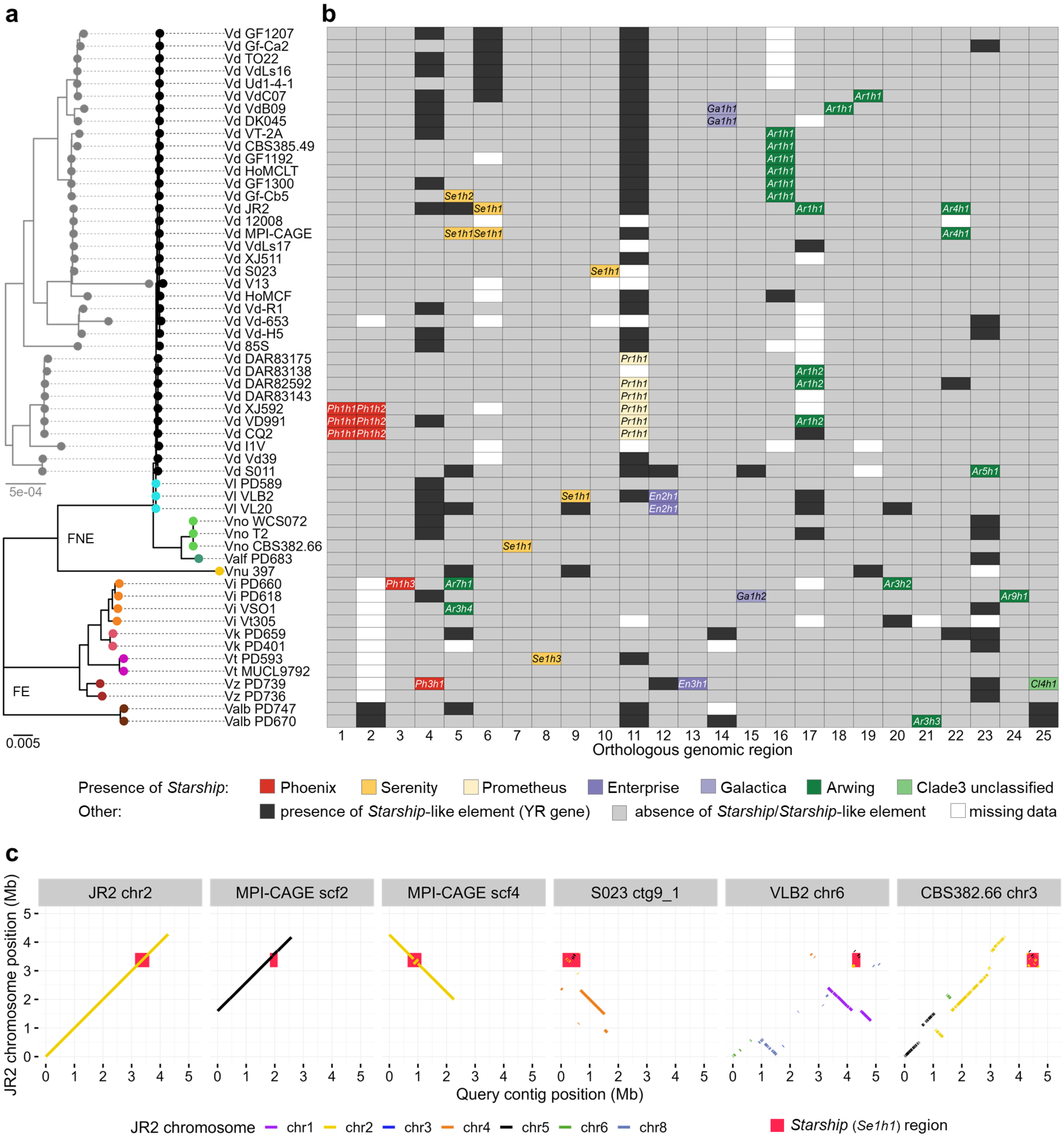

Figure S7. *Starship* regions are dynamic. a, *Verticillium* phylogeny as described in Fig. 1a. **b**, *Starship* occurrence in different genomic regions across the *Verticillium* genomes. Columns indicate orthologous genomic regions identified based on the presence of flanking orthologous genes. Colors indicate the presence/absence of *Starships* or of truncated *Starship*-like elements. Haplotype names are described for *Starships* identified with Starfish. **c**. Synteny plots between the *V. dahliae* JR2 genome and the *Se1h1 Starship* regions. Each plot shows the synteny between the JR2 chromosomes (Y-axis) and each chromosome (chr), contig (ctg), or scaffold (scf) containing the *Se1h1 Starship* (X-axis), with diagonal lines colored by syntenic JR2 chromosome. Red background indicates the coordinates of the *Se1h1 Starship*.

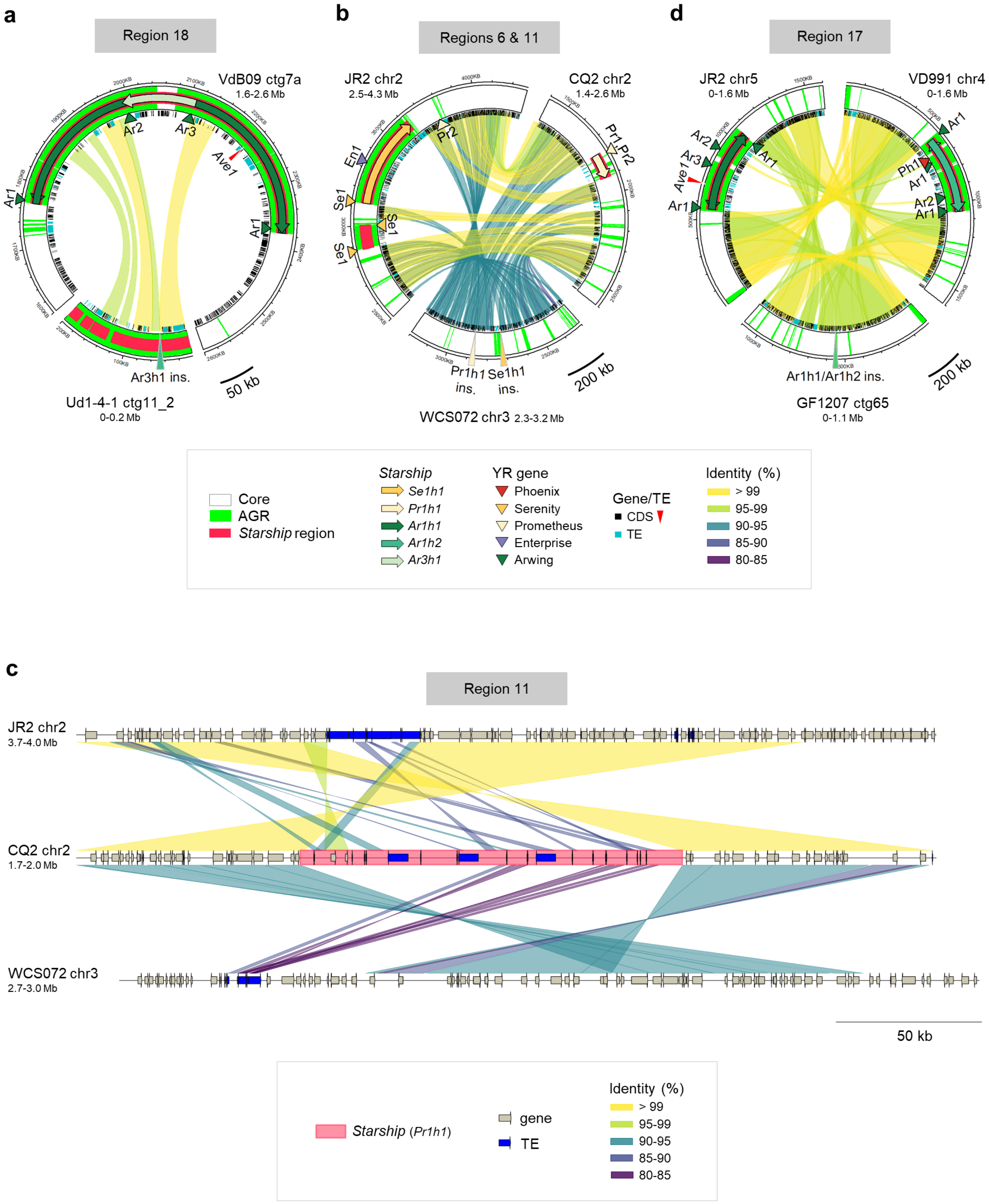

Figure S8. Plasticity of *Starship* regions between *Verticillium* genomes. **a**, Nested *Starship* insertion. **b-c**, *Starship* fragmentation. **d**, Invasion of different *Starships*. See Fig. 2a legend for the details of symbols. The region number corresponds to Supplementary Fig. S7b.

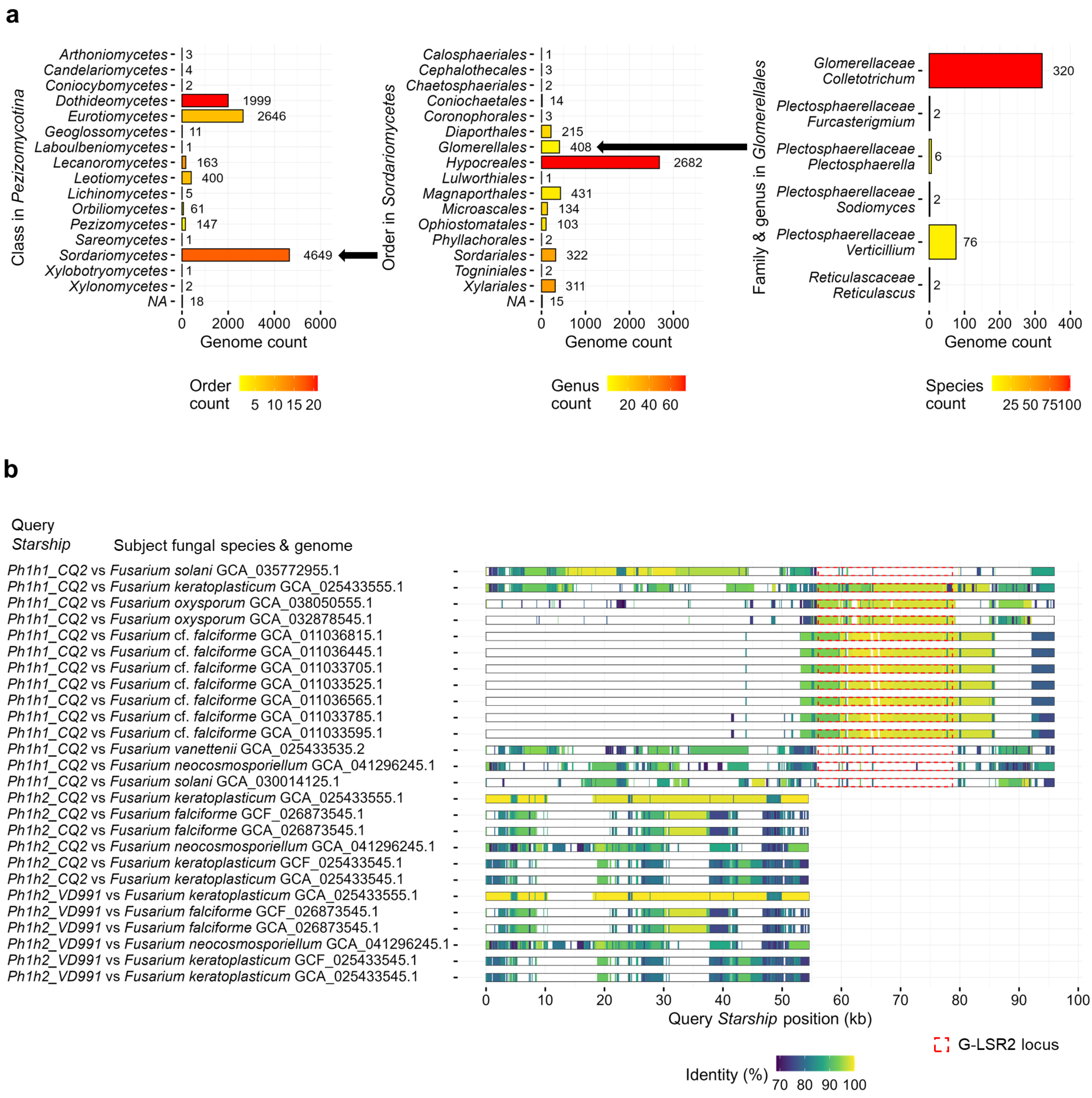

Figure S9. Search of Pezizomycotina genomes for *Verticillium Starship* orthologs. a, Taxa of the input *Pezizomicotina* genomes used for the similarity search in Fig. 5. The left, middle, and right panels indicate the genomes of the phylum Pezizomycotina, the class Sordariomycetes, and the order Glomerellales, respectively. **b**, Coverage plots for *Verticillium Starships* with hits in the similarity search of Pezizomycotina genomes. Bar colors represent nucleotide identities for individual hits. Dashed red lines indicate the position of the G-LSR2 region.

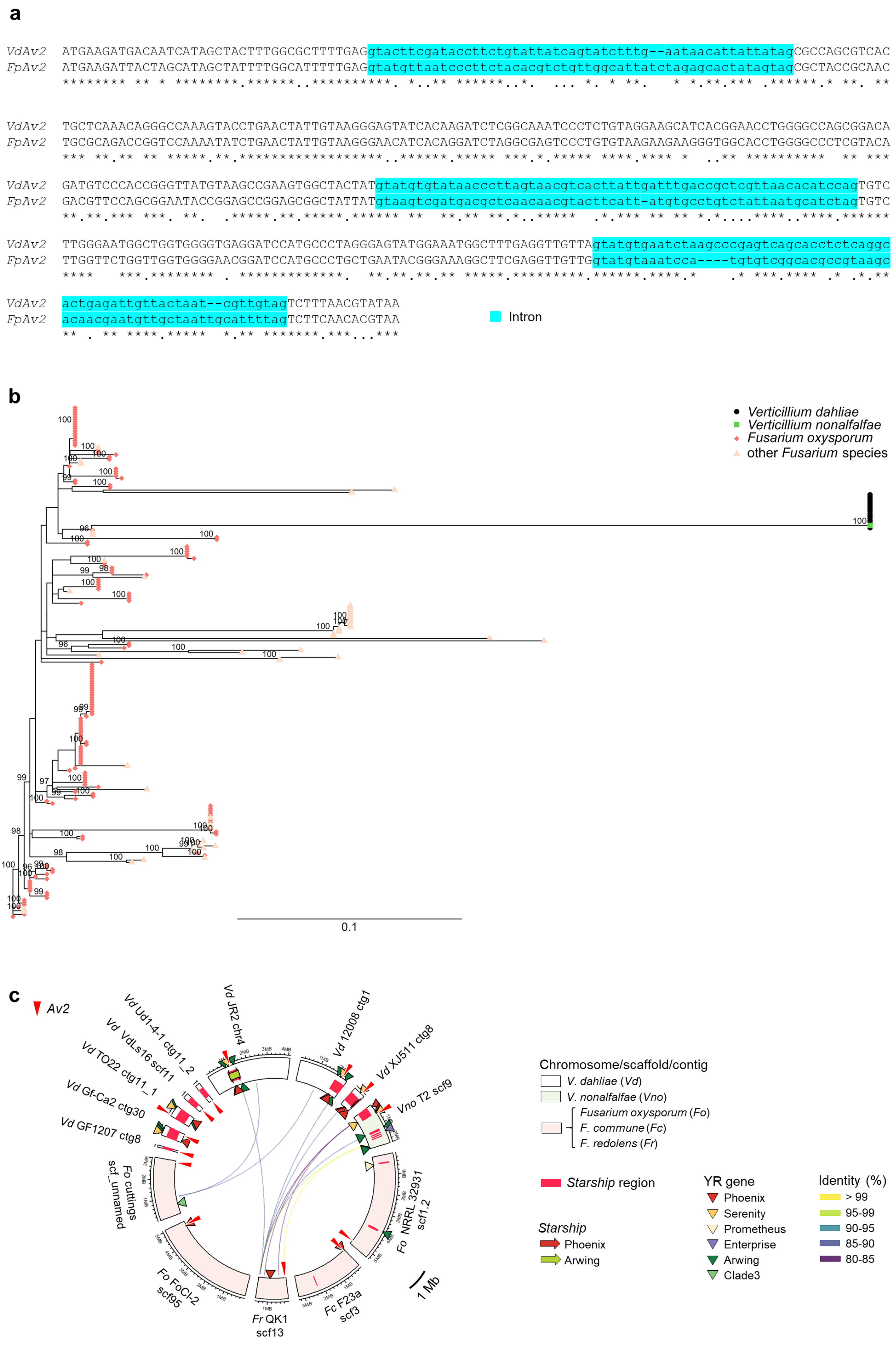

a
VdAv2 ATGAAGATGACAATCATAGCTACTTTGGCGCTTTTGAGgtacttcgataccttctgtattatcagtatctttg--aataacattattatagCGCCAGCGTCAC
FpAv2 ATGAAGATTACTAGCATAGCTATTTTGGCATTTTTGAGgtatgttaatcccttctacacgtctgttggcattatctagagcactatagtagCGCTACCGCAAC
VdAv2 TGCTCAAACAGGGCCAAAGTACCTGAACTATTGTAAGGGAGTATCACAAGATCTCGGCAAATCCCTCTGTAGGAAGCATCACGGAACCTGGGGCCAGCGGACA
FpAv2 TGCGCAGACCGGTCCAAAATATCTGAACTATTGTAAGGGAACATCACAGGATCTAGGCGAGTCCCTGTGTAAGAAGAAGGGTGGCACCTGGGGCCCTCGTACA
VdAv2 GATGTCCCACCGGGTTATGTAAGCCGAAGTGGCTACTATgtatgtgtataacccttagtaacgtcacttattgatttgaccgctcgttaacacatccagTGTC
FpAv2 GACGTTCCAGCGGAATACCGGAGCCGGAGCGGCTATTATgtaagtcgatgacgctcaacaacgtacttcatt-atgtgcctgtctattaatgcatctagTGTC
VdAv2 TTGGGAATGGCTGGTGGGGTGAGGATCCATGCCCTAGGGAGTATGGAAATGGCTTTGAGGTTGTTAgtatgtgaatctaagcccgagtcagcacctctcaggc
FpAv2 TTGGTTCTGGTTGGTGGGGAACGGATCCATGCCCTGCTGAATACGGGAAAGGCTTCGAGGTTGTTGgtatgtaaatcca----tgtgtcggcacgccgtaagc
VdAv2 actgagattgttactaat--cgttgtagTCTTTAACGTATAA
FpAv2 acaacgaatgttgctaattgcatttttagTCTTCAACACGTAA
Intron
b
Verticillium dahliae
Verticillium nonalfalfae
Fusarium oxysporum
other Fusarium species
0.1
c
Av2
Vd Ud1-4-1 ctg11_2
Vd VdLs16 scf11
Vd TO22 ctg11_1
Vd Gf-Ca2 ctg30
Vd GF1207 ctg8
Vd JR2 chr4
Vd 12008 ctg1
Vd XJ511 ctg8
Vno T2 scf9
Fo NRRL 32931 scf1.2
Fc F23a scf3
Fr QK1 scf13
Fo FoCl-2 scf95
Fo cuttings scf_unnamed
1 Mb
Chromosome/scaffold/contig
V. dahliae (Vd)
V. nonalfalfae (Vno)
Fusarium oxysporum (Fo)
F. commune (Fc)
F. redolens (Fr)
Starship region
Starship
Phoenix
Arwing
YR gene
Phoenix
Serenity
Prometheus
Enterprise
Arwing
Clade3
Identity (%)
> 99
95-99
90-95
85-90
80-85

Figure S10. Horizontal transfer of *Av2* between *Verticillium* and *Fusarium*. **a**, Nucleotide sequence alignment between *Av2* in *Verticillium dahliae* strain JR2 (*VdAv2*) and the ortholog in *Fusarium phyllophilum* strain NRRL 13617 (*FpAv2*). **b**, Phylogeny of *Av2* and its orthologs in *Verticillium* and *Fusarium*. Scale bars indicate nucleotide substitutions per site. Bootstrap values (>95%) for 1,000 iterations are shown at the nodes. **c**, Location of *Av2* in *Verticillium* and *Fusarium* chromosomes (chr), scaffolds (scf), or contigs (ctg). See Fig. 2a legend for the details of symbols. Ribbons connect syntenic regions between *Verticillium* and *Fusarium* genomic regions. Accession numbers of *Fusarium* genomes are described in Supplementary Data 16.

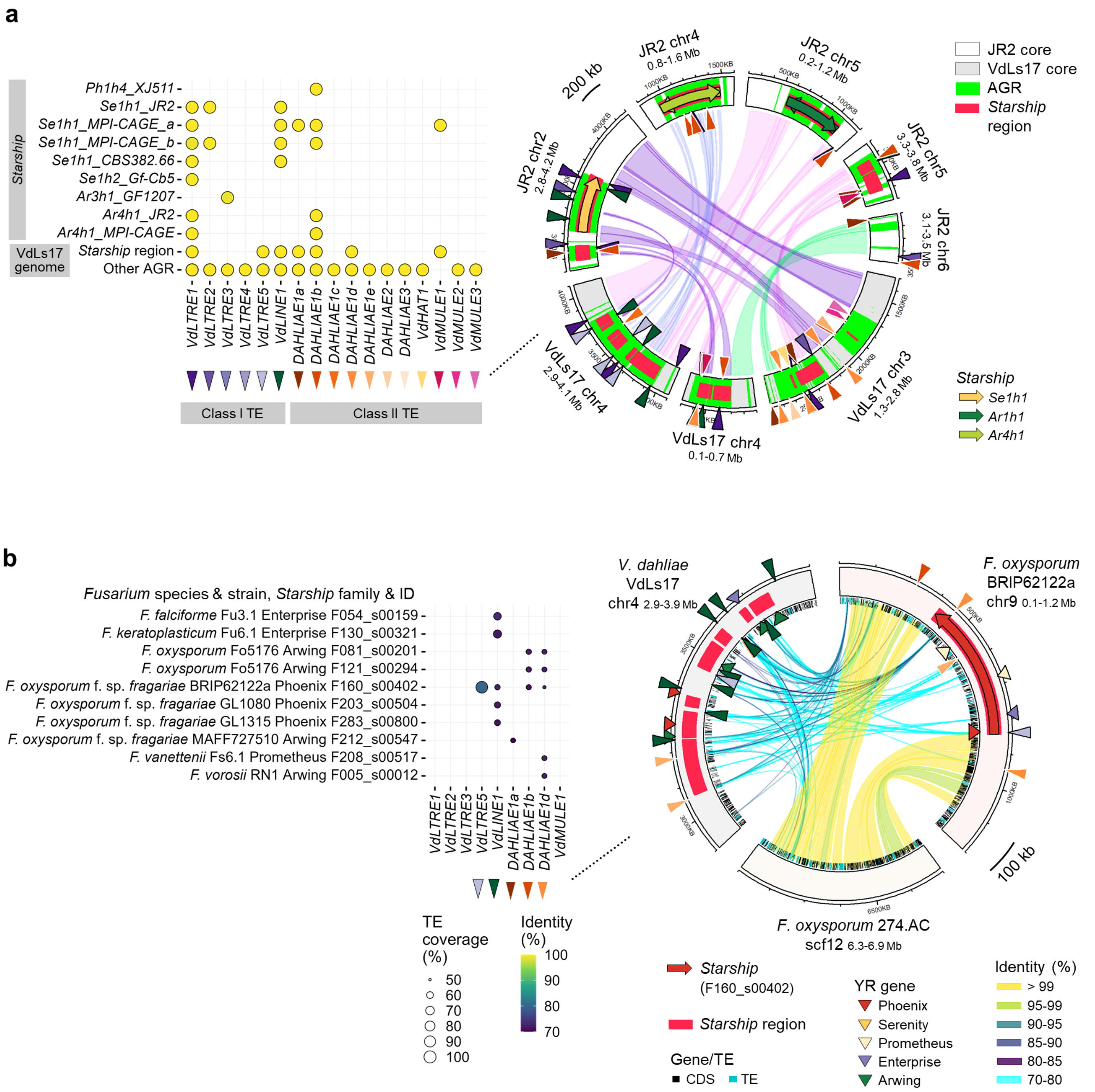

Figure S11. Occurrence of active transposable elements (TEs) in *Starships* of *Verticillium* and *Fusarium*. **a**, Occurrence of transcriptionally active TEs characterized in *V. dahliae* strain VdLs17 in diverse *Verticillium Starships* and adaptive genomic regions (AGRs). Circles in the left matrix represent the presence of the TEs (nucleotide identity >99% and coverage 100%) in the respective *Verticillium Starships* and VdLs17 genomic compartments. The circular plot shows the position of individual TEs with long triangles in/around *Starships* and *Starship* regions of *V. dahliae* strains JR2 and VdLs17. See Fig. 2a legend for the details of symbols. **b**, Occurrence of orthologs (nucleotide identity >70% and coverage >50%) of *Verticillium Starship* cargo TEs (**a**) in *Fusarium Starships*. Circles in the left matrix represent the presence of the respective TE orthologs in individual *Fusarium Starships*, with sizes representing TE coverage and colors representing nucleotide identity. The circular plot shows the position of individual TEs with long triangles in/around a *Starship* in *F. oxysporum* and *Starship* regions in VdLs17.

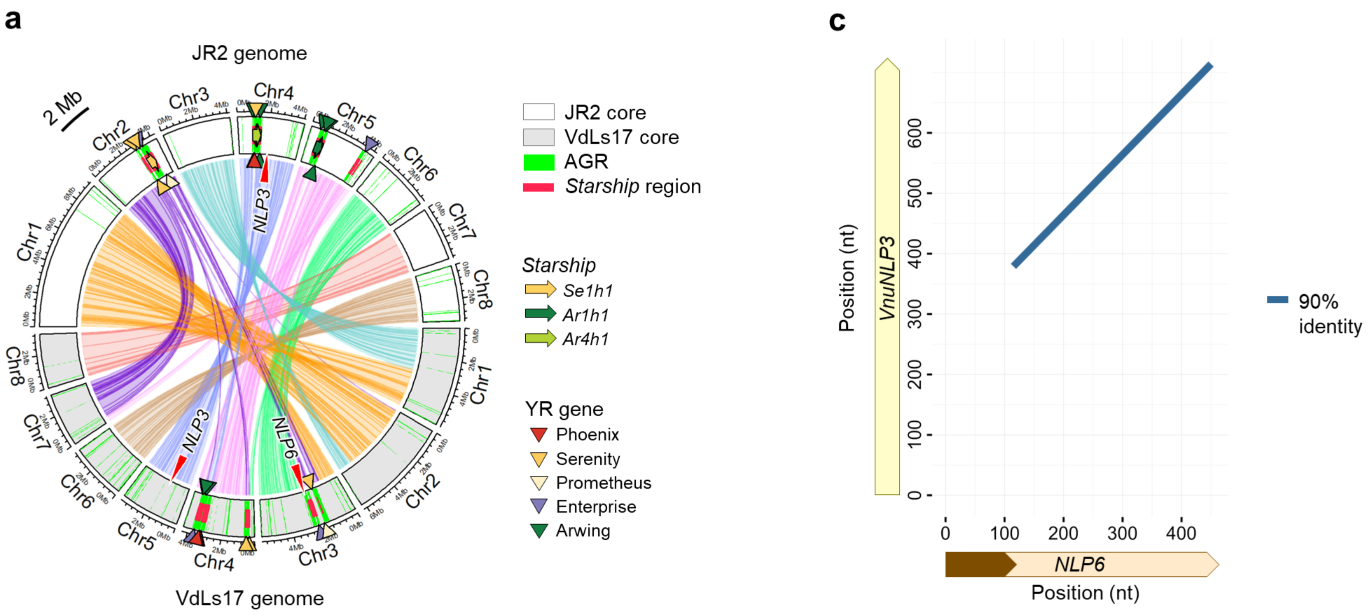

b

```
NLP6      MWTCRFMAPLADL------------VPTYGFLAPLTD----GA---------PR--------RYT------------------------------------
VnuNLP3   M-VSKIFATLASIAMVAAGPISLRAVVAHDSLNPVEQRVQTGAIGDAIAKFNPRLHIANGCQPYTAVNDAGDTSGGLQDSGNISAGCRDQSKGQTYARAKV
          * ..:::*.**.:            * ::. * *: :    **         **         **

NLP6      ---QLSII-----------------AHRHDFENIVVFVDDPAVNPIPAILGGAASGHGEYKTTATPDVEGDSVKVEYFTTFLTNHELQFTATSGKTYPISD
VnuNLP3   VNGQLAIMYSWYMPKDQPIAGNVPGGHRHDFENIVVFVDDPAANPTPAILGGAASGHGEYKKTATPDVEGDSVKVEYFTTFPTNHELQFTATSGKTYPISD
             **:*:                 .****************.** ***************.******************* *******************

NLP6      WDAMPQGARDALETTDFGSANVPFKDGNFDSNLAKAAL
VnuNLP3   WDAMPQATRDALEATDFGSANVPFKDANFDNNLAKAAL
          ******.:*****:************.***.*******
```

Putative signal peptide   Heptapeptide motif

Figure S12. Origin of *NLP6* in *V. dahliae*. a, Locations of *NLP3* and *NLP6* in *V. dahliae* strains JR2 and VdLs17. See Fig. 2a legend for the details of symbols. **b**, Amino acid sequence alignment between *NLP6* in *Verticillium dahliae* strain VdLs17 and NLP3 in *V. nubilum* strain 397 (*VnuNLP3*). The putative secretion signal and the conserved hexapeptide motif are highlighted. **c**, Coverage plot based on the pairwise nucleotide sequence alignment between *NLP6* and *VnuNLP3*.

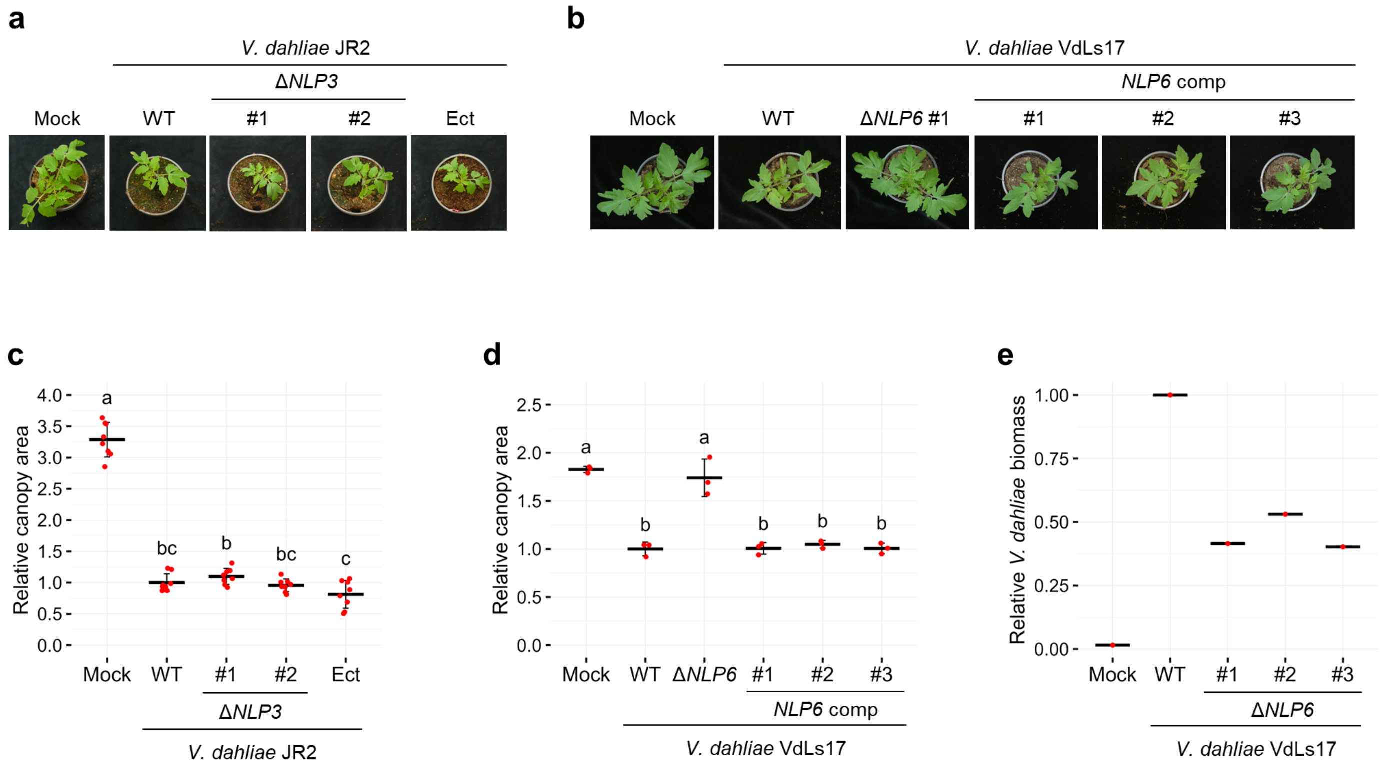

Figure S13. Test for virulence contribution of *NLP3* and *NLP6* in *V. dahliae*. Symptoms (**a**, **b**), canopy area (**c**, **d**), and *V. dahliae* biomass (**e**) in tomato plants inoculated with wild-type (WT) and mutants of *V. dahliae* at 14 days post inoculation. The contribution of *NLP3* to virulence in *V. dahliae* strain JR2 was assessed using two independent *NLP3* deletion (Δ) lines and an ectopic (Ect) control line transformed with the empty pRF-HU2 vector (**a**, **c**). The contribution of *NLP6* to virulence in *V. dahliae* strain VdLs17 was tested using three independent *NLP6* complementation (comp) lines in the Δ*NLP6* genetic background (**b**, **d**). The contribution of *NLP6* to *V. dahliae* colonization was assessed with three independent *NLP6* deletion lines (**e**). Points indicate relative values for individual plants ($n = 8$ in **c** and $n = 3$ in **d**) or pooled plant samples ($n = 1$ in **e**), divided by the mean of WT-inoculated plants. Crossbars and error bars indicate mean ± standard deviation. Different letter labels indicate significant differences (two-sided Tukey's test, adjusted $p < 0.05$).
