# Supplementary material for: Starship giant transposons dominate plastic genomic regions in a fungal plant pathogen and drive virulence evolution
Source: Nat Commun. 2025 Jul 24;16:6806. doi: 10.1038/s41467-025-61986-6 (PMC12289983; doi:10.1038/s41467-025-61986-6)
Supplement: Supplementary file 2 — Description of Additional Supplementary Files [file 41467_2025_61986_MOESM2_ESM.pdf]

Description of Additional Supplementary Files:

Supplementary Data 1: *Verticillium* genomes used for Starship identification.

Supplementary Data 2: Assembly statistics of *Verticillium* genomes.

Supplementary Data 3: *Verticillium Starships* identified in this study.

Supplementary Data 4: *Verticillium* tyrosine recombinase (YR) genes identified in this study.

Supplementary Data 5: Coordinates of *Starship* regions in *Verticillium* genomes.

Supplementary Data 6: Coordinates of genomic compartments in *Verticillium dahliae* strains JR2 and VdLs17.

Supplementary Data 7: Hits from the search of *Verticillium Starships* with genes in Pathogen-Host Interactions Database (PHI-base).

Supplementary Data 8: Average Nucleotide Identity (ANI) between the *Verticillium* genomes.

Supplementary Data 9: Pezizomycotina genomes used for similarity searches.

Supplementary Data 10: *Fusarium* genomes used for *Starship* identification.

Supplementary Data 11: *Fusarium Starships* identified in this study.

Supplementary Data 12: *Starship* regions identified with Starfish extend.

Supplementary Data 13: *Starship* regions identified by synteny search.

Supplementary Data 14: Accession numbers of RNA-Seq and ChIP-Seq reads.

Supplementary Data 15: Accession numbers of query genes and TEs.

Supplementary Data 16: Coordinates of *Av2* orthologs.

Supplementary Data 17: Primers used for gene deletion.
