# Supplementary material for: Starship giant transposons dominate plastic genomic regions in a fungal plant pathogen and drive virulence evolution
Source: Nat Commun. 2025 Jul 24;16:6806. doi: 10.1038/s41467-025-61986-6 (PMC12289983; doi:10.1038/s41467-025-61986-6)
Supplement: Supplementary file 4 — Reporting Summary [file 41467_2025_61986_MOESM4_ESM.pdf]

nature portfolio

Corresponding author(s): Bart Thomma

Last updated by author(s): Jul 2, 2025

Reporting Summary

Nature Portfolio wishes to improve the reproducibility of the work that we publish. This form provides structure for consistency and transparency in reporting. For further information on Nature Portfolio policies, see our Editorial Policies and the Editorial Policy Checklist.

Statistics

For all statistical analyses, confirm that the following items are present in the figure legend, table legend, main text, or Methods section.

n/a	Confirmed	
☐	☒	The exact sample size (*n*) for each experimental group/condition, given as a discrete number and unit of measurement
☐	☒	A statement on whether measurements were taken from distinct samples or whether the same sample was measured repeatedly
☐	☒	The statistical test(s) used AND whether they are one- or two-sided<br>*Only common tests should be described solely by name; describe more complex techniques in the Methods section.*
☐	☒	A description of all covariates tested
☐	☒	A description of any assumptions or corrections, such as tests of normality and adjustment for multiple comparisons
☐	☒	A full description of the statistical parameters including central tendency (e.g. means) or other basic estimates (e.g. regression coefficient) AND variation (e.g. standard deviation) or associated estimates of uncertainty (e.g. confidence intervals)
☐	☒	For null hypothesis testing, the test statistic (e.g. *F*, *t*, *r*) with confidence intervals, effect sizes, degrees of freedom and *P* value noted<br>*Give P values as exact values whenever suitable.*
☒	☐	For Bayesian analysis, information on the choice of priors and Markov chain Monte Carlo settings
☒	☐	For hierarchical and complex designs, identification of the appropriate level for tests and full reporting of outcomes
☐	☒	Estimates of effect sizes (e.g. Cohen's *d*, Pearson's *r*), indicating how they were calculated

Our web collection on statistics for biologists contains articles on many of the points above.

Software and code

Policy information about availability of computer code

Data collection	NCBI Short Read Archive data were collected using sra-tools version 3.1.0. NCBI genome data were collected using datasets version 16.17.3. Accession numbers of all sequence data are listed in Supplementary Tables.
Data analysis	The software used for the data analyses is described in Methods with the options. The software and versions are also listed here: Canu version 2.2; BUSCO version 5.7.0; FastANI version 1.33; RepeatModeler version 2.0.5; RepeatMasker version 4.1.5; BRAKER version 3.0.8; GeneMark-EP+ version 4.72_lic; AUGUSTUS version 3.0.8; eggNOG-mapper version 2.1.12; EDTA version 2.2.1; Starfish version 1.0.0; MetaEuk version 6.a5d39d9; HMMER version 3.3.2; MMseqs2 version 14.7e284; BLAST versions 2.12.0+, 2.15.0+, and 2.16.0+; sourmash version 4.8.3; mcl version 14-137; MUMmer version 4.0.0rc1; bedtools version 2.30.0; fastp version 0.19.5; STAR version 2.7.10a; TEtranscripts version 2.2.1; BWA version 0.7.17; samtools version 1.10; featureCounts version 2.0.1; PHI-base version 4.17; REALPHY version 1.13; Bowtie version 2.2.5; PhyML version 3.3.20220408; mashtree version 1.4.6; SeqKit version 2.3.0; MAFFT versions 7.511 and 7.526; trimAl version 1.4.rev15; IQ-TREE version 2.0.3; HMMER version 3.3.2; SignalP versions 3.0 and 6.0; R version 4.4.2; R package 'tidyverse' version 2.0.0; R package 'DESeq2' version 1.42.1; R package 'stats' version 4.3.1; R package 'dunn.test' version 1.3.6; R package 'multcomp' version 1.4.26; R package 'RVAideMemoire' version 0.9-83-11; R package 'circlize' version 0.4.16133; R package 'gggenomes' version 1.0.0134; R package 'genoPlotR' version 0.8.11; R package 'ggtree' v3.10.1136; R package 'ggplot2' version 3.5.1137

For manuscripts utilizing custom algorithms or software that are central to the research but not yet described in published literature, software must be made available to editors and reviewers. We strongly encourage code deposition in a community repository (e.g. GitHub). See the Nature Portfolio guidelines for submitting code & software for further information.

Data

Policy information about availability of data

All manuscripts must include a data availability statement. This statement should provide the following information, where applicable:

- Accession codes, unique identifiers, or web links for publicly available datasets
- A description of any restrictions on data availability
- For clinical datasets or third party data, please ensure that the statement adheres to our policy

The genomes assembled in this study and previous studies have been submitted to NCBI under the BioProject accession PRJNA1253319. Genome annotation files and genome assemblies with complex gap information have been deposited at Zenodo (https://zenodo.org/records/15450312). Other genome sequence, RNA-Seq, and ChIP-Seq data used in this study are available in the NCBI database under the accession numbers listed in Supplementary Tables. Source data are provided with this paper.

Research involving human participants, their data, or biological material

Policy information about studies with human participants or human data. See also policy information about sex, gender (identity/presentation), and sexual orientation and race, ethnicity and racism.

Reporting on sex and gender	Not applicable.
Reporting on race, ethnicity, or other socially relevant groupings	Not applicable.
Population characteristics	Not applicable.
Recruitment	Not applicable.
Ethics oversight	Not applicable.

Note that full information on the approval of the study protocol must also be provided in the manuscript.

Field-specific reporting

Please select the one below that is the best fit for your research. If you are not sure, read the appropriate sections before making your selection.

☐ Life sciences ☐ Behavioural & social sciences ☒ Ecological, evolutionary & environmental sciences

For a reference copy of the document with all sections, see nature.com/documents/nr-reporting-summary-flat.pdf

Ecological, evolutionary & environmental sciences study design

All studies must disclose on these points even when the disclosure is negative.

Study description	This study investigated the impact of Starship giant transposons on the genome evolution of fungi in the Verticillium genus. Starships were identified based on their insertion/deletion variations by whole genome alignment among 56 strains of 10 species of Verticillium. The genetic, transcriptomic, epigenetic, and 3D genomic characteristics of Starship regions and other genomic regions were analyzed by genome sequence, RNA-Seq, ChIP-Seq, and Hi-C analyses. Horizontal Starship transfers were inferred by phylogenetic analyses using genomes of publicly available Pezizomycotina genomes. The origin of a virulence gene associated with Starships was explored by homology searches and phylogenetic analyses using the Verticillium genomes.
Research sample	Assembled genomes or genomic sequencing reads of Pezizomycotina fungal genomes.
Sampling strategy	The sample size was determined in accordance with the standard study design for genomics and pathogenicity assays of plant pathogenic fungi.
Data collection	The data were collected from the databases and repository described in Methods and Supplementary Tables. Procedures for the collection of original data are provided in the database or literature cited in the manuscripts.
Timing and spatial scale	The fungal isolates and their genome data were collected at various dates in various places as described in the database or literature cited in the manuscripts. This information is not relevant to this study so the information is not described in the manuscript.
Data exclusions	No data were excluded.
Reproducibility	The reproducibility of bioinformatic analyses can be verified with public sequence data and software described in the manuscript. The results of pathogenicity assays were reproduced in independent experiments by two authors.
Randomization	Randomization was not applied because this study does not aim the sampling survey.

Blinding	Blinding was not applied because this study used fungi and plants as organisms so the results are not expected to be affected by blinding.

Did the study involve field work? ☐ Yes ☒ No

Reporting for specific materials, systems and methods

We require information from authors about some types of materials, experimental systems and methods used in many studies. Here, indicate whether each material, system or method listed is relevant to your study. If you are not sure if a list item applies to your research, read the appropriate section before selecting a response.

Materials & experimental systems

n/a	Involved in the study	
☒	☐	Antibodies
☒	☐	Eukaryotic cell lines
☒	☐	Palaeontology and archaeology
☒	☐	Animals and other organisms
☒	☐	Clinical data
☒	☐	Dual use research of concern
☐	☒	Plants

Methods

n/a	Involved in the study	
☐	☒	ChIP-seq
☒	☐	Flow cytometry
☒	☐	MRI-based neuroimaging

Dual use research of concern

Policy information about dual use research of concern

Hazards

Could the accidental, deliberate or reckless misuse of agents or technologies generated in the work, or the application of information presented in the manuscript, pose a threat to:

No	Yes	
☒	☐	Public health
☒	☐	National security
☒	☐	Crops and/or livestock
☒	☐	Ecosystems
☒	☐	Any other significant area

Experiments of concern

Does the work involve any of these experiments of concern:

No	Yes	
☒	☐	Demonstrate how to render a vaccine ineffective
☒	☐	Confer resistance to therapeutically useful antibiotics or antiviral agents
☒	☐	Enhance the virulence of a pathogen or render a nonpathogen virulent
☒	☐	Increase transmissibility of a pathogen
☒	☐	Alter the host range of a pathogen
☒	☐	Enable evasion of diagnostic/detection modalities
☒	☐	Enable the weaponization of a biological agent or toxin
☒	☐	Any other potentially harmful combination of experiments and agents

Plants

Seed stocks	Tomato cv. Moneymaker
Novel plant genotypes	Not applicable.
Authentication	Not applicable.

ChIP-seq

Data deposition

- [x] Confirm that both raw and final processed data have been deposited in a public database such as GEO.
- [ ] Confirm that you have deposited or provided access to graph files (e.g. BED files) for the called peaks.

Data access links<br>*May remain private before publication.*	Raw data are available from https://www.ncbi.nlm.nih.gov/sra/?term=SRR10571949 (replicate 1) and https://www.ncbi.nlm.nih.gov/sra/?term=SRR10571948 (replicate 2). Processed data are available in Source data files provided with this paper.
Files in database submission	Not applicable.
Genome browser session (e.g. UCSC)	Not applicable.

Methodology

Replicates	2
Sequencing depth	The original read information is available via the raw data links. The mean read lengths after trimming were 118 bp for replicate 1 and 116 bp for replicate 2. The total number of mapped reads was 8,551,984 for replicate 1 and 5,225,048 for replicate 2. The sequencing depths were 30X and 17X, respectively.
Antibodies	Rabbit anti H3K27me3 (Polyclonal) (Active Motif, catalog number 39155)
Peak calling parameters	Not applicable.
Data quality	Not applicable.
Software	Described in Methods with the options and versions.
