# Supplementary material for: Starship giant transposons dominate plastic genomic regions in a fungal plant pathogen and drive virulence evolution
Source: Nat Commun. 2025 Jul 24;16:6806. doi: 10.1038/s41467-025-61986-6 (PMC12289983; doi:10.1038/s41467-025-61986-6)
Supplement: Supplementary file 6 — Source Data [file 41467_2025_61986_MOESM6_ESM.zip › Source Data/FigS10b_MSA.html]

trimAl v1.4 Summary


```
    Selected Sequences:   217 /Selected Residues:     410
    Deleted Sequences:      0 /Deleted Residues:       55

    Gaps Scores:                                             =0=   <.001  <.050  <.100  <.150  <.200  <.250  <.350  <.500  <.750  <1.00   =1=  
    Similarity Scores:                                       =0=   <1e-6  <1e-5  <1e-4  <.001  <.010  <.100  <.250  <.500  <.750  <1.00   =1=  

                                                                   10        20        30        40        50        60        70        80        90       100       110       120
                                                           =========+=========+=========+=========+=========+=========+=========+=========+=========+=========+=========+=========+
    GCA_000260075.2#JH651001.1_53017-53465                 atgaagattactagcatagctattttggcatttttgaggtatgttaat-cccttctaaacgtctgttagcatt-----atctaaagca-ctatt-gtagcgctactgccactgcgcagac
    GCA_000271745.2#JH717840.1_5103546-5103992             atgaagatcattagcatagctgttttggcatttttgaggtatgttagt-cttttcaacatgtttgttagtgtt-----aactaaagca-ctatt-atagcgctacagctactgcgcagac
    GCA_000400815.2#CP009080.1_1179336-1179785             atgaagatgacaatcatagctactttggcgcttttgaggtacttcgat-accttctgtattatcagtatcttt-----gaataa--ca-ttatt-atagcgccagcgtcactgctcaaac
    GCA_000585705.1#AEYB01001584.1_5717-6165               atgaagcttactagcgtagctattttggcatttttgaggtctgttaat-cccttctacacgtctattgtcatt-----atctaacgca-ctgct-gtagcgccaccgtcactgcgcagac
    GCA_001599515.1#BCHB01000008.1_128823-129266           atgaagatcactagcatagctattttggcgtttttatggtatgttaat-cccttcgacacgtctgttagcatt-----atctagagca-taatt-atagcgctactgccactgcgcagac
    GCA_001680525.1#MADZ01000944.1_3517-3965               atgaagcttactagcgtagctattttggcatttttgaggtctgttaat-cccttctacacgtctattgtcatt-----atctaacgca-ctgct-gtagcgccaccgtcactgcgcagac
    GCA_001680535.1#MAED01002951.1_1336-1785               atgaagcttactagcgtagctattttggcatttttgaggtctgttaat-cccttctacacgtctattgtcatt-----atctaacgca-ctgctggtagcgccaccgtcactgcgcagac
    GCA_001680595.1#MAEE01000161.1_9576-10025              atgaagcttactagcgtagctattttggcatttttgaggtctgttaat-cccttctacacgtctattgtcatt-----atctaacgca-ctgctggtagcgccaccgtcactgcgcagac
    GCA_001680605.1#MADY01000512.1_3407-3855               atgaagcttactagcgtagctattttggcatttttgaggtctgttaat-cccttctacacgtctattgtcatt-----atctaacgca-ctgct-gtagcgccaccgtcactgcgcagac
    GCA_001680665.1#MADX01001027.1_5081-5529               atgaagcttactagcgtagctattttggcatttttgaggtctgttaat-cccttctacacgtctattgtcatt-----atctaacgca-ctgct-gtagcgccaccgtcactgcgcagac
    GCA_001680685.1#MAEC01002105.1_1371-1819               atgaagcttactagcgtagctattttggcaattttgaggtctgttaat-cccttctacacgtctattgtcatt-----atctaacgca-ctgct-gtagcgccaccgtcactgcgcagac
    GCA_001680725.1#MAEF01000026.1_12451-12900             atgaagcttactagcgtagctattttggcatttttgaggtctgttaat-cccttctacacgtctattgtcatt-----atctaacgca-ctgctggtagcgccaccgtcactgcgcagac
    GCA_001931975.2#MSJJ02000021.1_21498-21944             atgaagatcattagcatagctgttttggcatttttgaggtatgttagt-cttttcaacatgtttgttagtgtt-----aactaaagca-ctatt-atagcgctacagctactgcgcagac
    GCA_002233775.1#NJCV01000665.1_343-790                 atgaagattactagcatagctattatggcatttttgaggtatgttaat-cccttctgcacgtctgttagcatc-----atctaaacca-ctatt-gtagcgcgatcgccactgcgcagac
    GCA_002233895.1#NJCL01000052.1_6292-6740               atgaagattactggcatagctattttggcatttttgaggtatgttaat-cccttctacacgtctgttagcatt-----atctaaagca-ctatt-atagcgctaccgccactgcgcagac
    GCA_002233895.1#NJCL01000986.1_2608-3056               atgaagattactagcatagctattttggcatttttgaggtatgttaat-cccttctacacgtctgttggcatt-----atctaaagca-ctgtt-gtagcgctaccgccactgcgcagac
    GCA_002233895.1#NJCL01000862.1_714-1162                atgaagattactagcatagctattttggcatttttgaggtatgttaat-cccttctacacgtctgttagcatt-----atctaaagca-ctatt-gtagcgctaccgccactgcgcagac
    GCA_002233915.1#NJCM01002567.1_585-1030                atgaagattactagcatagctattttggcatttttgaggtatgttcat-cccttctacacgtctgttggcatt-----atctaaagca-ctgtt-gtagctctaccgccactgcgcagac
    GCA_002233915.1#NJCM01000349.1_3242-3690               atgaagattactagcatagctattttggcatttttgaggtatgttaat-cccttctacacgtctgttggcatt-----atctaaagca-ctgtt-gtagcgctaccgccactgcgcagac
    GCA_002233935.2#NJBT02000074.1_114534-114981           atgaagattactagcatagctattttggcgtttttgaggtatgttaat-cccttctgcacgtctgttagcatt-----atctaaagca-ctatt-gtagcgttaccgccactgcgcagac
    GCA_002234115.1#NJCF01001371.1_90-531                  -------ttactagtatagctattttggcatttttgaggtatgttaat-cccttctacacgtctgttagcatt-----atctaaagca-ctatt-gtagcgctaccgccactgcgcagac
    GCA_002234115.1#NJCF01000302.1_3435-3883               atgaagattactagcatagctattttggcatttttgaggtatgttaat-cccttctacacgtctgttagcatt-----atctaaagca-ctatt-gtagcgctaccgccactgcgcagac
    GCA_002234115.1#NJCF01000961.1_5490-5938               atgaagattactggcatagctattttggcatttttgaggtatgttaat-cccttctacacgtctgttagcatt-----atctaaagca-ctatt-atagcgctaccgccactgcgcagac
    GCA_002234195.1#NJCK01002649.1_264-712                 atgaagattactagcatagctattttggcatttttgaggtatgttaat-cccttctacacgtctgttggcatt-----atctaaagca-ctgtt-gtagcgctaccgccactgcgcagac
    GCA_002234205.1#NJCQ01003026.1_1778-2226               atgaagattactagcatagctattttggcatttttgaggtatgttaat-cccttctacacgtctgttagcatt-----atctaaagca-ctatt-atagcgctaccgccactgcgcagac
    GCA_002234205.1#NJCQ01001471.1_358-806                 atgaagattactagcatagctattttggcatttttgaggtatgttaat-cccttctacacgtctgttagcatt-----atctaaagca-ctatt-gtagcgctaccgccactgcgcagac
    GCA_002234235.1#NJCR01003189.1_7833-8281               atgaagattactagcatagctattttggcatttttgaggtatgttaat-cccttctacacgtctgttagcatt-----atctaaagca-ctatt-atagcgctaccgccactgcgcagac
    GCA_002234235.1#NJCR01000523.1_1652-2100               atgaagattactagcatagctattttggcatttttgaggtatgttaat-cccttctacacgtctgttagcatt-----atctaaagca-ctatt-gtagcgctaccgccactgcgcagac
    GCA_002234255.1#NJCS01000197.1_9074-9523               atgaagattactagcatagctattttggcatttttgaggtatattaat-cccttctgtatgtctattagcatt-----atctaaagcacctatt-gtagcgttactgctactatacaggc
    GCA_002234255.1#NJCS01000257.1_23088-23528             ---------actagcatagctattttagcatttttaaggtatattaat-cccttgtgcacgtctattagcatt-----atctaaagtacctatt-gtagtgctactgctactgtgcaggc
    GCA_002234285.1#NJCT01000251.1_2006-2454               atgaagattactagcatagctattttggcatttttgaggtatgttaat-cccttctacacgtctgttagcatt-----atctaaagca-ctatt-gtagcgctaccgccactgcgcagac
    GCA_002234285.1#NJCT01000112.1_2108-2556               atgaagattactggcatagctattttggcatttttgaggtatgttaat-cccttctacacgtctgttagcatt-----atctaaagca-ctatt-atagcgctaccgccactgcgcagac
    GCA_002776445.1#PDEZ01000231.1_7918-8368               atgaagatgacaatcatagctactttggcgcttttgaggtacttcgat-accttctgtattatcagtatcttt-----gaataa--ca-ttatt-atagcgccagcgtcactgctcaaac
    GCA_002892985.1#MPSH01000001.1_2159685-2160134         atgaagatgacaatcatagctactttggcgcttttgaggtacttcgat-accttctgtattatcagtatcttt-----gaataa--ca-ttatt-atagcgccagcgtcactgctcaaac
    GCA_002893025.1#PHNV01000722.1_4459-4908               atgaagatgacaatcatagctactttggcgcttttgaggtacttcgat-accttctgtattatcagtatcttt-----gaataa--ca-ttatt-atagcgccagcgtcactgctcaaac
    GCA_002893035.1#PHNW01000920.1_4459-4908               atgaagatgacaatcatagctactttggcgcttttgaggtacttcgat-accttctgtattatcagtatcttt-----gaataa--ca-ttatt-atagcgccagcgtcactgctcaaac
    GCA_002982035.1#PVPY01001450.1_5032-5474               atgaagatcactagcatagctattttggcattcttgaggtatgttaat-cccttagatacgtctgttagcatt-----atctaaagca-ctatt-gtagcgctactgccactgcgcagac
    GCA_002982055.1#PVPZ01001881.1_721-1163                atgaagatcactagcatagctattttggcattcttgaggtatgttaat-ccctcagacacgtctgttagcatt-----atctaaagca-ctatt-gtagcgctactgccactgcgtagac
    GCA_003025205.1#PXUO01000119.1_69286-69732             atgaagatcattagcatagctgttttggcatttttgaggtatgttagt-cttttcaacatgtttgttagtgtt-----aactaaagca-ctatt-atagcgctacagctactgcgcagac
    GCA_003025235.1#PXUN01000132.1_66066-66512             atgaagatcattagcatagctgttttggcatttttgaggtatgttagt-cttttcaacatgtttgttagtgtt-----aactaaagca-ctatt-atagcgctacagctactgcgcagac
    GCA_004109745.1#RSDZ01000158.1_4397-4846               atgaagatgacaatcatagctactttggcgcttttgaggtacttcgat-accttctgtattatcagtatcttt-----gaataa--ca-ttatt-atagcgccagcgtcactgctcaaac
    GCA_004141715.1#MQTW01000814.1_378-825                 atgaagattactagcatagctattatggcatttttgaggtatgttaat-cccttctgcacgtctgttagcatc-----atctaaacca-ctatt-gtagcgcgatcgccactgcgcagac
    GCA_004291455.1#QUWZ01000068.1_80333-80705             atgaagattactagcatagctattttggcatttctggggtatgttaat-cccttctacacgtctgttagcatt-----atctaaagca-ctatt-gtagcgctatctccactgcgcagac
    GCA_004292535.1#QUXA01000044.1_77168-77540             atgaagattactagcatagctattttggcatttctggggtatgttaat-cccttctacacgtctgttagcatt-----atctaaagca-ctatt-gtagcgctatctccactgcgcagac
    GCA_009297365.1#WGOL01000900.1_612-1060                atgaagattactagcatagctattttggcattcttgaggtatgttaat-cccttctacacgtctgttagcatt-----atctaaagca-ctatt-gtagcgctaccgccactgcgcagac
    GCA_009297515.1#WGOI01001607.1_1477-1925               atgaaggttactagcatagctattttggcatttttgaggtatgttaat-cccttctacacgcctgttaacatt-----atctaaagca-ctatt-gtagcgctactgccactgcgcagac
    GCA_009297675.1#WGOJ01003344.1_1223-1671               atgaaggttactagcatagctattttggcatttttgaggtatgttaat-cccttctacacgtctgttagcatt-----atctaaagca-ctatt-gtagcgctactgccactgcgcagac
    GCA_009298545.1#WGQK01000668.1_4136-4584               atgaagattactagcatagctattttggcatttttgaggtatgttaat-cccttctacacgtctgttagcatt-----atctaaagca-gtatt-gtagcgctaccgccactgcgcagac
    GCA_009298855.1#WGQN01001540.1_1461-1906               atgaagattactagcatagctattttggcatttttgaggtatgttaat-cccttctacacgtctgttagcatt-----atctaaacca-ctatc-gtagcgctactgccactgcgcagac
    GCA_009298985.1#WGRN01000796.1_1047-1495               atgaagcttactagcatagctattttggcatttttgaggtatgttaat-cccttcgacacgtctattagcatt-----atctaaagca-ctatt-atagcgctactgccactgcgcagac
    GCA_009299045.1#WGRK01001155.1_826-1274                atgaagcttactagcatagctattttggcatttttaaggtatattaat-cccttcgacacgtctattagcacc-----atctaaagta-ctatt-atagcgctactgccactgcgcagac
    GCA_009299075.1#WGRJ01000863.1_12409-12855             atgaagatcattagcatagctgttttggcatttttgaggtatgttagt-cttttcaacatgtttgttagtgtt-----aactaaagca-ctatt-atagcgctacagctactgcgcagac
    GCA_009299115.1#WGRH01001009.1_1459-1907               atgaagattactagcatagctattttggcatttttgaggtatgttaat-cccttctacacgtctgttagcatt-----atctaaagca-gtatt-gtagcgctaccgccactgcgcagac
    GCA_009299215.1#WGRS01000248.1_42092-42540             atgaagattactagcatagctattttggcatttttgaggtatgttaat-cccttctacacgtctgttagcatt-----atctaaagca-ctatt-gtagcgctactgccactgcgcagac
    GCA_009299215.1#WGRS01000488.1_939-1388                atgaagattactagcatagctattttggcatttttgaggtatgttaatccccttctacacgtctgttagcatt-----atctaaacca-ctatt-gtagcgctactgccactgcgcagac
    GCA_009299215.1#WGRS01000332.1_7989-8437               atgaaggttactagcatagctattttggcatttttgaggtatgttaat-cccttctacacgtctgttagcatc-----atctaaagca-ctatt-gtagcgctactgccactgcgcagac
    GCA_009663885.1#WJDV01000002.1_418952-419401           atgaagatgacaatcatagctactttggcgcttttgaggtacttcgat-accttctgtattatcagtatcttt-----gaataa--ca-ttatt-atagcgccagcgtcactgctcaaac
    GCA_009746015.1#VLJC01000049.1_70684-71130             atgaagatcattagcatagctgttttggcatttttgaggtatgttagt-cttttcaacatgtttgttagtgtt-----aactaaagca-ctatt-atagcgctacagctactgcgcagac
    GCA_011032855.1#WESG01000339.1_10823-11271             atgaagattactagcatagctattttggcatttttgaggtatgttaat-cccttctacacgtctgttagcatt-----atctaaagca-ttatc-gtagcgctaccgccactgcacagac
    GCA_011032945.1#WNXM01001784.1_7328-7777               atgaagatgacaatcatagctactttggcgcttttgaggtacttcgat-accttctgtattatcagtatcttt-----gaataa--ca-ttatt-atagcgccagcgtcactgctcaaac
    GCA_011032955.1#WNXL01001437.1_4250-4699               atgaagatgacaatcatagctactttggcgcttttgaggtacttcgat-accttctgtattatcagtatcttt-----gaataa--ca-ttatt-atagcgccagcgtcactgctcaaac
    GCA_011032965.1#WNXK01000961.1_4382-4831               atgaagatgacaatcatagctactttggcgcttttgaggtacttcgat-accttctgtattatcagtatcttt-----gaataa--ca-ttatt-atagcgccagcgtcactgctcaaac
    GCA_011033455.1#WESL01002533.1_4420-4868               atgaagcttactagcatagctattttggcatttttaaggtatattaat-cccttcgacacgtctattagcacc-----atctaaagta-ctatt-atagcgctactgccactgcgcagac
    GCA_011033455.1#WESL01001216.1_8955-9403               atgaagattactagcatagctattttggcatttttgaggtatgttaat-cccttctacacgtctgttagcatt-----atctaaagca-ctatt-atagcgctaccgccactgcgcagac
    GCA_011033575.1#WESJ01009652.1_394-842                 atgaagattactagcatagctattttggcatttttgaggtatgttaat-cccttctacacgtctgttagcatt-----atctaaagca-ctatt-atagcgctaccgccactgcgcagac
    GCA_011033575.1#WESJ01007920.1_731-1149                atgaagcttactagcatagctattttggcatttttaaggtatattaat-cccttcgacacgtctattagcacc-----atctaaagta-ctatt-atagcgctactgccactgcgcagac
    GCA_011033685.1#WESM01008445.1_1386-1834               atgaagattactagcatagctattttggcatttttgaggtatgttaat-cccttctacacgtctgttagcatt-----atctaaagca-ctatt-atagcgctaccgccactgcgcagac
    GCA_011033745.1#WETB01002153.1_7139-7587               atgaagcttactagcatagctattttggcatttttaaggtatattaat-cccttcgacacgtctattagcacc-----atctaaagta-ctatt-atagcgctactgctactgcgcagac
    GCA_011033805.1#WESY01002113.1_415-863                 atgaagcttactagcatagctattttggcatttttaaggtatattaat-cccttcgacacgtctattagcacc-----atctaaagta-ctatt-atagcgctactgctactgcgcagac
    GCA_011033815.1#WESW01000773.1_2707-3155               atgaagcttactagcatagctattttggcatttttaaggtatattaat-cccttcgacacgtctattagcacc-----atctaaagta-ctatt-atagcgctactgccactgcgcagac
    GCA_011033815.1#WESW01000649.1_851-1299                atgaagattactagcatagctattttggcatttttgaggtatgttaat-cccttctacacgtctgttagcatt-----atctaaagca-ctatt-atagcgctaccgccactgcgcagac
    GCA_011033895.1#WETL01000467.1_850-1298                atgaagattactagcatagctattttggcatttttgaggtatgttaat-cccttctacacgtctgttagcatt-----atctaaagca-ctatt-atagcgctaccgccactgcgcagac
    GCA_011033895.1#WETL01000641.1_3860-4278               atgaagcttactagcatagctattttggcatttttaaggtatattaat-cccttcgacacgtctattagcacc-----atctaaagta-ctatt-atagcgctactgccactgcgcagac
    GCA_011034195.1#WETT01000020.1_547-995                 atgaagcttactagcatagctattttggcatttttaaggtatattaat-cccttcgacacgtctattagcacc-----atctaaagta-ctatt-atagcgctactgctactgcgcagac
    GCA_011034205.1#WETS01005722.1_2442-2890               atgaagcttactagcatagctattttggcatttttaaggtatattaat-cccttcgacacgtctattagcacc-----atctaaagta-ctatt-atagcgctactgctactgcgcagac
    GCA_011034415.1#WEUC01001584.1_7719-8167               atgaagcttactagcatagctattttggcatttttaaggtatattaat-cccttcgacacgtctattagcacc-----atctaaagta-ctatt-atagcgctactgctactgcgcagac
    GCA_011034445.1#WEUB01001158.1_3523-3971               atgaagcttactagcatagctattttggcatttttaaggtatattaat-cccttcgacacgtctattagcacc-----atctaaagta-ctatt-atagcgctactgccactgcgcagac
    GCA_011034445.1#WEUB01000999.1_8836-9284               atgaagattactagcatagctattttggcatttttgaggtatgttaat-cccttctacacgtctgttagcatt-----atctaaagca-ctatt-atagcgctaccgccactgcgcagac
    GCA_011034575.1#WEUE01008972.1_493-941                 atgaagattactagcatagctattttggcatttttgaggtatgttaat-cccttctacacgtctgttagcatt-----atctaaagca-ctatt-atagcgctaccgccactgcgcagac
    GCA_011034625.1#WEUR01000714.1_7159-7607               atgaagcttactagcatagctattttggcatttttaaggtatattaat-cccttcgacacgtctattagcacc-----atctaaagta-ctatt-atagcgctactgctactgcgcagac
    GCA_011034645.1#WEUV01000741.1_2997-3445               atgaagattactaacatagctattttggcgtctttgaggtatgttaat-cccttcgacacgcctgttagtatt-----atctaaagta-ttatc-gtagcgctgctgccactgcgcagac
    GCA_011034655.1#WEUQ01004890.1_3105-3553               atgaagcttactagcatagctattttggcatttttaaggtatattaat-cccttcgacacgtctattagcacc-----atctaaagta-ctatt-atagcgctactgctactgcgcagac
    GCA_011034655.1#WEUQ01000935.1_566-1014                atgaagattactagcatagctattttggcatttttgaggtatgttaat-cccttctacacgtctgttagcatt-----atctaaagca-ttatc-gtagcgctaccgccactgcacagac
    GCA_011034775.1#WEVI01000501.1_5488-5930               atgaagattactagcatagctattttggcatttttgaggtatgttaat-cccttctacacgtctgttagcatt-----atctaaagca-ctatt-atagcgctaccgccactgcgcagac
    GCA_011034775.1#WEVI01000783.1_2046-2494               atgaagcttactagcatagctattttggcatttttaaggtatattaat-cccttcgacacgtctattagcacc-----atctaaagta-ctatt-atagcgctactgccactgcgcagac
    GCA_011034815.1#WEVE01000912.1_9755-10173              atgaagattactagcatagctattttggcatttttgaggtatgttaat-cccttctacacgtctgttagcatt-----atctaaagca-ctatt-atagcgctaccgccactgcgcagac
    GCA_011034815.1#WEVE01002086.1_1620-2038               atgaagcttactagcatagctattttggcatttttaaggtatattaat-cccttcgacacgtctattagcacc-----atctaaagta-ctatt-atagcgctactgccactgcgcagac
    GCA_011034845.1#WEVA01001230.1_1747-2195               atgaagcttactagcatagctattttggcatttttaaggtatattaat-cccttcgacacgtctattagcacc-----atctaaagta-ctatt-atagcgctactgctactgcgcagac
    GCA_011035075.1#WEVS01001164.1_694-1142                atgaagcttactagcatagctattttggcatttttaaggtatattaat-cccttcgacacgtctattagcacc-----atctaaagta-ctatt-atagcgctactgctactgcgcagac
    GCA_011035185.1#WEVO01012916.1_426-874                 atgaagcttactagcatagctattttggcatttttaaggtatattaat-cccttcgacacgtctattagcacc-----atctaaagta-ctatt-atagcgctactgccactgcgcagac
    GCA_011035185.1#WEVO01003306.1_4368-4816               atgaagattactagcatagctattttggcatttttgaggtatgttaat-cccttctacacgtctgttagcatt-----atctaaagca-ctatt-atagcgctaccgccactgcgcagac
    GCA_011035205.1#WEVG01003463.1_75-523                  atgaagcttactagcatagctattttggcatttttaaggtatattaat-cccttcgacacgtctattagcacc-----atctaaagta-ctatt-atagcgctactgccactgcgcagac
    GCA_011035235.1#WEWM01001159.1_2667-3085               atgaagcttactagcatagctattttggcatttttaaggtatattaat-cccttcgacacgtctattagcacc-----atctaaagta-ctatt-atagcgctactgccactgcgcagac
    GCA_011035235.1#WEWM01001540.1_2265-2683               atgaagattactagcatagctattttggcatttttgaggtatgttaat-cccttctacacgtctgttagcatt-----atctaaagca-ctatt-atagcgctaccgccactgcgcagac
    GCA_011035245.1#WEWK01000657.1_7022-7440               atgaagcttactagcatagctattttggcatttttaaggtatattaat-cccttcgacacgtctattagcacc-----atctaaagta-ctatt-atagcgctactgctactgcgcagac
    GCA_011035245.1#WEWK01000404.1_16648-17029             --------------------------------------------------------------------gcatt-----atctaaagca-ctatt-atagcgctactgccactgcgcagac
    GCA_011035255.1#WEWN01000527.1_2412-2830               atgaagcttactagcatagctattttggcatttttaaggtatattaat-cccttcgacacgtctattagcacc-----atctaaagta-ctatt-atagcgctactgccactgcgcagac
    GCA_011035255.1#WEWN01000751.1_2320-2738               atgaagattactagcatagctattttggcatttttgaggtatgttaat-cccttctacacgtctgttagcatt-----atctaaagca-ctatt-atagcgctaccgccactgcgcagac
    GCA_011035265.1#WEWJ01000412.1_17167-17548             --------------------------------------------------------------------gcatt-----atctaaagca-ctatt-atagcgctactgccactgcgcagac
    GCA_011035265.1#WEWJ01000569.1_529-977                 atgaagcttactagcatagctattttggcatttttaaggtatattaat-cccttcgacacgtctattagcacc-----atctaaagta-ctatt-atagcgctactgctactgcgcagac
    GCA_011035345.1#WEWE01000725.1_7055-7503               atgaagcttactagcatagctattttggcatttttaaggtatattaat-cccttcgacacgtctattagcacc-----atctaaagta-ctatt-atagcgctactgctactgcgcagac
    GCA_011035345.1#WEWE01000822.1_176-557                 --------------------------------------------------------------------gcatt-----atctaaagca-ctatt-atagcgctactgccactgcgcagac
    GCA_011035375.1#WEWB01000636.1_176-557                 --------------------------------------------------------------------gcatt-----atctaaagca-ctatt-atagcgctactgccactgcgcagac
    GCA_011035375.1#WEWB01000586.1_665-1113                atgaagcttactagcatagctattttggcatttttaaggtatattaat-cccttcgacacgtctattagcacc-----atctaaagta-ctatt-atagcgctactgctactgcgcagac
    GCA_011035435.1#WEWD01000787.1_1777-2225               atgaagcttactagcatagctattttggcatttttaaggtatattaat-cccttcgacacgtctattagcacc-----atctaaagta-ctatt-atagcgctactgctactgcgcagac
    GCA_011035455.1#WEVZ01000295.1_5889-6270               --------------------------------------------------------------------gcatt-----atctaaagca-ctatt-atagcgctactgccactgcgcagac
    GCA_011035485.1#WEVY01000687.1_5745-6126               --------------------------------------------------------------------gcatt-----atctaaagca-ctatt-atagcgctactgccactgcgcagac
    GCA_011035485.1#WEVY01000540.1_528-976                 atgaagcttactagcatagctattttggcatttttaaggtatattaat-cccttcgacacgtctattagcacc-----atctaaagta-ctatt-atagcgctactgctactgcgcagac
    GCA_011035495.1#WEVW01000533.1_1949-2397               atgaagcttactagcatagctattttggcatttttaaggtatattaat-cccttcgacacgtctattagcacc-----atctaaagta-ctatt-atagcgctactgctactgcgcagac
    GCA_011035625.1#WEWS01001096.1_809-1227                atgaagattactagcatagctattttggcatttttgaggtatgttaat-cccttctacacgtctgttagcatt-----atctaaagca-ctatt-atagcgctaccgccactgcgcagac
    GCA_011035625.1#WEWS01001310.1_2416-2834               atgaagcttactagcatagctattttggcatttttaaggtatattaat-cccttcgacacgtctattagcacc-----atctaaagta-ctatt-atagcgctactgccactgcgcagac
    GCA_011035755.1#WEXC01000589.1_22209-22657             atgaagattactagcatagctattttggcatttttgaggtatgttaat-cccttctacacgtctgttagcatt-----atctaaagca-ttatc-gtagcgctaccgccactgcacagac
    GCA_011035835.1#WEXF01000545.1_8821-9239               atgaagattactagcatagctattttggcatttttgaggtatgttaat-cccttctacacgtctgttagcatt-----atctaaagca-ctatt-atagcgctaccgccactgcgcagac
    GCA_011035835.1#WEXF01000653.1_3838-4256               atgaagcttactagcatagctattttggcatttttaaggtatattaat-cccttcgacacgtctattagcacc-----atctaaagta-ctatt-atagcgctactgccactgcgcagac
    GCA_011035975.1#WEXJ01007704.1_1039-1487               atgaagcttactagcatagctattttggcatttttgaggtatgttaat-cccttctacacgtctgttagcatt-----atctaaagta-ctatt-atagcgctactgccactgcgcagac
    GCA_011035995.1#WEXI01000903.1_7456-7904               atgaagcttactagcatagctattttggcatttttaaggtatattaat-cccttcgacacgtctattagcacc-----atctaaagta-ctatt-atagcgctactgctactgcgcagac
    GCA_011035995.1#WEXI01001093.1_807-1255                atgaagattactagcatagctattttggcatttttgaggtatgttaat-cccttctacacgtctgttagcatt-----atctaaagca-ttatc-gtagcgctaccgccactgcacagac
    GCA_011036045.1#WEXT01000869.1_163-611                 atgaagcttactagcataactattttggcatttttgaggtatgttaat-cccttcgacacgtctattagcatc-----atctaaagca-ctatt-atagcgctactgccactgcgcagac
    GCA_011036135.1#WEXQ01000241.1_21717-22165             atgaagattactagcatagctattttggcatttttgaggtatgttaat-cccttctacacgtctgttagcatt-----atctaaagca-ttatc-gtagcgctaccgccactgcacagac
    GCA_011036135.1#WEXQ01000570.1_528-976                 atgaagcttactagcatagctattttggcatttttaaggtatattaat-cccttcgacacgtctattagcacc-----atctaaagta-ctatt-atagcgctactgctactgcgcagac
    GCA_011036215.1#WEYG01000053.1_6223-6671               atgaagcttactagcataactattttggcatttttgaggtatgttaat-cccttcgacacgtctattagcatc-----atctaaagca-ctatt-atagcgctactgccactgcgcagac
    GCA_011036305.1#WEXY01003662.1_3416-3864               atgaagcttactagcatagctattttggcatttttaaggtatattaat-cccttcgacacgtctattagcacc-----atctaaagta-ctatt-atagcgctactgccactgcgcagac
    GCA_011036305.1#WEXY01002318.1_5546-5994               atgaagattactagcatagctattttggcatttttgaggtatgttaat-cccttctacacgtctgttagcatt-----atctaaagca-ctatt-atagcgctaccgccactgcgcagac
    GCA_011036365.1#WEYQ01001970.1_647-1095                atgaagcttactagcatagctattttggcatttttaaggtatattaat-cccttcgacacgtctattagcacc-----atctaaagta-ctatt-atagcgctactgctactgcgcagac
    GCA_011036595.1#WEYT01000407.1_15167-15585             atgaagcttactagcatagctattttggcatttttaaggtatattaat-cccttcgacacgtctattagcacc-----atctaaagta-ctatt-atagcgctactgccactgcgcagac
    GCA_011036595.1#WEYT01000705.1_8787-9235               atgaagattactagcatagctattttggcatttttgaggtatgttaat-cccttctacacgtctgttagcatt-----atctaaagca-ctatt-atagcgctaccgccactgcgcagac
    GCA_011036765.1#WEUS01002153.1_588-1036                atgaagcttactagcatagctattttggcatttttaaggtatattaat-cccttcgacacgtctattagcacc-----atctaaagta-ctatt-atagcgctactgctactgcgcagac
    GCA_011036795.1#WEUZ01000271.1_2987-3435               atgaagattactaacatagctattttggcgtctttgaggtatgttaat-cccttcgacacgcctgttagtatt-----atctaaagta-ttatc-gtagcgctgctgccactgcgcagac
    GCA_011036875.1#WEWI01000026.1_11685-12103             atgaagattactagcatagctattttggcatttttgaggtatgttaat-cccttctacacgtctgttagcatt-----atctaaagca-ctatt-atagcgctaccgccactgcgcagac
    GCA_011036875.1#WEWI01000910.1_3838-4256               atgaagcttactagcatagctattttggcatttttaaggtatattaat-cccttcgacacgtctattagcacc-----atctaaagta-ctatt-atagcgctactgccactgcgcagac
    GCA_011037075.1#WEZD01015082.1_365-770                 -------------------------------------------ttaat-cccttcgacacgtctattagcacc-----atctaaagta-ctatt-atagcgctactgccactgcgcagac
    GCA_011421275.1#WIKV01000306.1_26907-27325             atgaagattactagcatagctattttggcatttttgaggtatgttaat-cccttctacacgtctgttagcatt-----atctaaagca-ctatt-atagcgctaccgccactgcgcagac
    GCA_011421275.1#WIKV01000622.1_3838-4256               atgaagcttactagcatagctattttggcatttttaaggtatattaat-cccttcgacacgtctattagcacc-----atctaaagta-ctatt-atagcgctactgccactgcgcagac
    GCA_011421285.1#WIKU01001812.1_697-1145                atgaagcttactagcatagctattttggcatttttaaggtatattaat-cccttcgacacgtctattagcacc-----atctaaagta-ctatt-atagcgctactgctactgcgcagac
    GCA_011421305.1#WIKW01002869.1_809-1227                atgaagattactagcatagctattttggcatttttgaggtatgttaat-cccttctacacgtctgttagcatt-----atctaaagca-ctatt-atagcgctaccgccactgcgcagac
    GCA_011421305.1#WIKW01002126.1_1789-2207               atgaagcttactagcatagctattttggcatttttaaggtatattaat-cccttcgacacgtctattagcacc-----atctaaagta-ctatt-atagcgctactgccactgcgcagac
    GCA_011421375.1#WILC01001342.1_696-1144                atgaagcttactagcatagctattttggcatttttaaggtatattaat-cccttcgacacgtctattagcacc-----atctaaagta-ctatt-atagcgctactgctactgcgcagac
    GCA_011426335.1#WILD01001610.1_1018-1466               atgaagcttactagcatagctattttggcatttttaaggtatattaat-cccttcgacacgtctattagcacc-----atctaaagta-ctatt-atagcgctactgccactgcgcagac
    GCA_011426355.1#WILF01007384.1_372-820                 atgaagcttactagcatagctattttggcatttttaaggtatattaat-cccttcgacacgtctattagcacc-----atctaaagta-ctatt-atagcgctactgccactgcgcagac
    GCA_011426355.1#WILF01000505.1_6553-7001               atgaagattactagcatagctattttggcatttttgaggtatgttaat-cccttctacacgtctgttagcatt-----atctaaagca-ctatt-atagcgctaccgccactgcgcagac
    GCA_013170945.1#MU047874.1_170025-170474               atgaagatgacaatcatagctactttggcgcttttgaggtacttcgat-accttctgtattatcagtatcttt-----gaataa--ca-ttatt-atagcgccagcgtcactgctcaaac
    GCA_013184365.1#JABCJX010000598.1_3113-3561            atgaagattactagcatagctattttggcatttttgaggtatgttatt-cccttctacacgtctgttagcatt-----atctaaagca-ctact-gtagcgctaccgccactgcgcagac
    GCA_013266185.1#JABEXW010001135.1_3715-4163            atgaagcttactagcatagctattttggcatttttgaggtatgttaat-cccctcgacacgtctatcagcatc-----atctaaagcc-ctatc-atagtgttactgccactgcgcagac
    GCA_013347355.2#JAALGN020000013.1_426461-426909        atgaagattactagcatagctattttggcatttttgaggtatgttaat-cccttctacacgtctgttagcatt-----atctaaagca-ctatt-gtagcgctactgccactgcgcagac
    GCA_013363175.1#JABEEP010001877.1_2080-2528            atgaagcttactagcgtagctattttggcaattttgaggtctgttaat-cccttctacacgtctattgtcatt-----atctaacgca-ctgct-gtagcgccaccgtcactgcgcagac
    GCA_013396025.1#JAAOAQ010001214.1_4450-4898            atgaagattactagcatagctattttggcatttttgaggtatgttaat-cccttctacacgtctgttggcatt-----atctagagca-ctata-gtagcgctaccgcaactgcgcagac
    GCA_013396205.1#JAAOAN010000160.1_45920-46352          atgaagcttactagcattgttatcttggcatttctgaggtatgtttat-cccttgaataagtctgtttgcatt-----atctaaagca-ctgtt-gcagcgccactgccactgcgcagac
    GCA_013618355.1#JABFES010000455.1_7017-7460            atgaggattactagcatagctattttggcgtttttgaggtatgttaat-cccttcgacacgtctgttagcatt-----atctagagca-caatt-gtagcgctactgccactgcgcagac
    GCA_013623525.1#JABGLY010000028.1_42787-43233          atgaagattactaccgtcgctattgtggcattcttgaggtatgttgtt-tccttctcaacgtctgtcaatactacagcatctaaagca-acatt-atagcgccactgccactgcgagcac
    GCA_013623715.1#JABFFJ010000182.1_85034-85470          atgaagattacaagcattgctattctggcatttttgaggtatgtttat-cccttttacacatctattagcaat-----atctaaagca-ctttt-atagcgctagcgccactgcgcagac
    GCA_014324575.1#WXUM01000474.1_1010-1458               atgaagattactagcatagctattttggcatttttgaggtatgttaat-cccttctacacgtctgttagcatt-----atctaaagca-ctatt-gtagcgctactgccactgcgcagac
    GCA_014324595.1#WXUN01001159.1_797-1245                atgaagattactagcatagctattttggcatttttgaggtatgttaat-cccttctacacgtctgttagcatt-----atctaaagca-ctatt-gtagcgctactgccactgcgcagac
    GCA_014324665.1#WXUR01000643.1_5483-5930               atgaagattactagcatagctattttggcatttttgaggtatgttaat-cccttctacacgtctgttagca-t-----atctaaagca-ctatt-gtagcgctactgccactgcgcagac
    GCA_014324745.1#JAAMUY010001820.1_1146-1594            atgaagattactagcatagctattttggcatttttgaggtatgttaat-cccttctacacgtctgttagcatt-----atctaaagca-ctatt-gtagcgctactgccactgcgcagac
    GCA_014324775.1#WXUT01000779.1_3905-4353               atgaagattactagcatagctattttggcatttttgaggtatgttaat-cccttctacacgtctgttagcatt-----atctaaagca-ctatt-gtagcgctactgccactgcgcagac
    GCA_014324795.1#WXUU01000551.1_4342-4789               atgaagattactagcatagctattttggcatttttgaggtatgttaat-cccttctacacgtctgttagca-t-----atctaaagca-ctatt-gtagcgctactgccactgcgcagac
    GCA_014324835.1#WXUX01001216.1_2785-3233               atgaagattactagcatagctattttggcatttttgaggtatgttaat-cccttctacacgtctgttagcatt-----atctaaagca-ctatt-gtagcgctactgccactgcgcagac
    GCA_014324865.1#WXUY01001109.1_5504-5951               atgaagattactagcatagctattttggcatttttgaggtatgttaat-cccttctacacgtctgttagca-t-----atctaaagca-ctatt-gtagcgctactgccactgcgcagac
    GCA_014324895.1#WXUZ01000915.1_1546-1994               atgaagattactagcgtagctattttggcatttttgaggtatgttaat-cccttctacacgtctgttagcatt-----atctaaagca-ctatt-gtagcgctactgccactgcgcagac
    GCA_014325045.1#WXVI01000570.1_8895-9343               atgaaggttactagcatagctattttggcatttttgaggtatgttaat-cccttctacacgcctgttaacatt-----atctaaagca-ctatt-gtagcgctactgccactgcgcagac
    GCA_014325205.1#JAAMVE010001426.1_3625-4073            atgaaggttactagcataactattttggcatttttgtggtatgttaat-cccttctacacgtctgttagcatt-----atctaaagca-ctatt-gtagcgctactgccactgcgcagac
    GCA_014325315.1#WXVP01000164.1_88523-88895             atgaagattactagcatagctattttggcatttctggggtatgttaat-cccttctacacgtctgttagcatt-----atctaaagca-ctatt-gtagcgctatctccactgcgcagac
    GCA_014843555.1#JAAOOQ010000005.1_239540-239989        atgaagattactagcatagctattttggcatttttgaggtatgttaat-cccttctacaagtctgttagcatt-----atccaaagca-ctatt-atagcgctactgccactgcgcagac
    GCA_014843565.1#JAAOOP010000035.1_171592-172041        atgaagattactagcatagctattttggcatttttgaggtatgttaat-cccttctacaagtctgttagcatt-----atccaaagca-ctatt-atagcgctactgccactgcgcagac
    GCA_014884825.1#JACTAS010000222.1_17723-18171          atgaagattactagcatagctattttggcatttttgaggtatgttaat-cccttctacacgtctgttagcatt-----atctaaagca-ctatc-gtagcgctaccgctactgcccagac
    GCA_018440645.1#JACOQZ010000013.1_178443-178892        atgaagatgacaatcatagctactttggcgcttttgaggtacttcgat-accttctgtattatcagtatcttt-----gaataa--ca-ttatt-atagcgccagcgtcactgctcaaac
    GCA_018440655.1#JACORB010000012.1_54438-54887          atgaagatgacaatcatagctactttggcgcttttgaggtacttcgat-accttctgtattatcagtatcttt-----gaataa--ca-ttatt-atagcgccagcgtcactgctcaaac
    GCA_019055395.1#JAHKNW010006905.1_873-1321             atgaagattactagcatagctattttggcatttttgaggtatgttaat-cccttctacacgtctgttagcatt-----atctaaagca-ctatt-gtagcgctactgccactgcgcagac
    GCA_019157295.1#JAELUQ010000008.1_3397787-3398230      atgaagattactagcatagctattttggcgtttttgaggtatgttaat-cccttcgacacgtctgttagcatt-----atctagagca-caatt-gtagcgctactgccactgcgcagac
    GCA_019191165.1#JAHARQ010000139.1_67224-67670          atgaagatcattagcatagctgttttggcatttttgaggtatgttagt-cttttcaacatgtttgttagtgtt-----aactaaagca-ctatt-atagcgctacagctactgcgcagac
    GCA_019191175.1#JAHARR010000129.1_15625-16071          atgaagatcattagcatagctgttttggcatttttgaggtatgttagt-cttttcaacatgtttgttagtgtt-----aactaaagca-ctatt-atagcgctacagctactgcgcagac
    GCA_019843925.1#JACDIY010000477.1_28970-29342          atgaagattactagcatagctattttggcatttctggggtatgttaat-cccttctacacgtctgttagcatt-----atctaaagca-ctatt-gtagcgctatctccactgcgcagac
    GCA_020726535.1#JAHEWJ010000011.1_1000856-1001305      atgaagatgacaatcatagctactttggcgcttttgaggtacttcgat-accttctgtattatcagtatcttt-----gaataa--ca-ttatt-atagcgccagcgtcactgctcaaac
    GCA_020744475.1#JAGMUX010000018.1_315043-315491        atgaagattactagcatagctattttggcatttttgaggtatgttaat-cccttctacacgtctgttagcatt-----atttaaagca-ctatt-gtagcgctactgctactgcgcagac
    GCA_020883595.1#JAJGQA010000014.1_323964-324412        atgaagcttactagcgtagctattttggcatttttgaggtctgttaat-cccttctacacgtctattgtcatt-----atctaacgca-ctgct-gtagcgccaccgtcactgcgcagac
    GCA_020883605.1#JAJHJC010000014.1_396196-396644        atgaagcttactagcgtagctattttggcatttttgaggtctgttaat-cccttctacacgtctattgtcatt-----atctaacgca-ctgct-gtagcgccaccgtcactgcgcagac
    GCA_020883615.1#JAJHJD010000017.1_393187-393635        atgaagcttactagcgtagctattttggcatttttgaggtctgttaat-cccttctacacgtctattgtcatt-----atctaacgca-ctgct-gtagcgccaccgtcactgcgcagac
    GCA_020883625.1#JAJGQB010000012.1_381552-382000        atgaagcttactagcgtagctattttggcatttttgaggtctgttaat-cccttctacacgtctattgtcatt-----atctaacgca-ctgct-gtagcgccaccgtcactgcgcagac
    GCA_021655895.1#JAJJWO010000410.1_1878-2303            --gaagatcactagcataactatcttggcattgctaaggtatgtcgat-cccttctatacgtc----agcatt-----atctaaag---gcact-gtagtgttaccgttactgcgcagac
    GCA_021730365.1#JAJOOV010000405.1_1213-1662            atgaagcttactagcgtagctattttggcatttttgaggtctgttaat-cccttctacacgtctattgtcatt-----atctaacgca-ctgctggtagcgccaccgtcactgcgcagac
    GCA_023065405.1#JAJTCY010000003.1_74358-74801          atgaagattactagcatagctattttggcgtttttgaggtatgttaat-cccttcgacacgtctgttagcatt-----atctagagca-caatt-gtagcgctactgccactgcgcagac
    GCA_023628715.1#JAMAKD010000122.1_67003-67449          atgaagatcattagcatagctgttttggcatttttgaggtatgttagt-cttttcaacatgtttgttagtgtt-----aactaaagca-ctatt-atagcgctacagctactgcgcagac
    GCA_024865645.1#JANKHN010000668.1_8435-8878            atgaagatcactagcatagctattttggcgtttttatggtatgttaat-cccttcgacacgtctgttagcatt-----atctagagca-taatt-atagcgctactgccactgcgcagac
    GCA_025201995.1#JAMSCT010000483.1_2189-2637            atgaagtttaccagcatagctattttggcctttttgaggtatgttaaa-cccttctacacgtctgttagcatt-----gtctaaagca-ctatt-atagcgctaccgccactgcgcagac
    GCA_025203115.1#JAMSFS010000057.1_218973-219344        atgaagattactagcatagctattttggcatttctggggtatgttaat-cccttctacacgtctgttagcatt-----atctaaagca-ctatt-gtagcgctatctccactgcgcagac
    GCA_025203755.1#JAMSFW010000517.1_9896-10342           atgaagcttactagtatagctattttggcatttttgaggtatgttaat-cccttctacacgtctgttagcatt-----acctaaagca-ctatt-gtagcgctaccgccactgcccagac
    GCA_025203755.1#JAMSFW010000664.1_112-560              atgaagcttactagtatagctattttggcatttttgaggtatgttaat-cccttcgacacgtctgttagcatt-----atctaaagca-ctatt-gtagcgctaccgccactgcgcagac
    GCA_025215475.1#JAMSDB010000983.1_1119-1567            atgaaggttactagcatagctattttggcatttttgaggtatgttaat-cccttctacacgtctgttaacatt-----atctaaagca-ctatt-gtagcgctactgccactgcgcagac
    GCA_025215845.1#JAMSCV010001163.1_880-1265             ----------------------------------------------------------------gttagcatt-----atctaaagca-ctatt-gtagcgctaccgccgctgcgcagac
    GCA_025215865.1#JAMSCS010000321.1_8136-8584            atgaagattactagcatagctattttggcatttttgaggtatgttaat-cccttctacacgtctgttagcatt-----atctaaagca-ctatt-gtagcgctaccgccactgcgcagac
    GCA_025215865.1#JAMSCS010000173.1_620-1068             atgaagcttactagcatagctattttggcatttttgaggtatgttaat-cccttcgacacgtctattagcatc-----atctaaagca-ctatt-atagcgctactgccactgcgcagac
    GCA_025215985.1#JAMSDF010000755.1_3429-3877            atgaaggttactagcatagctattttggcatttttgaggtatgttaat-cccttctacacgtctgttaacatt-----atctaaagca-ctatt-gtagcgctactgccactgcgcagac
    GCA_025216965.1#JAMSEY010000527.1_112-558              atgaagcttactagtatagctattttggcatttttgaggtatgttaat-cccttctacacgtctgttagcatt-----acctaaagca-ctatt-gtagcgctaccgccactgcccagac
    GCA_025216965.1#JAMSEY010000698.1_1866-2314            atgaagcttactagtatagctattttggcatttttgaggtatgttaat-cccttcgacacgtctgttagcatt-----atctaaagca-ctatt-gtagcgctaccgccactgcgcagac
    GCA_025217005.1#JAMSEZ010000669.1_2174-2622            atgaagcttactagtatagctattttggcatttttgaggtatgttaat-cccttcgacacgtctgttagcatt-----atctaaagca-ctatt-gtagcgctaccgccactgcgcagac
    GCA_025217005.1#JAMSEZ010000515.1_9896-10342           atgaagcttactagtatagctattttggcatttttgaggtatgttaat-cccttctacacgtctgttagcatt-----acctaaagca-ctatt-gtagcgctaccgccactgcccagac
    GCA_025217045.1#JAMSFA010000530.1_112-558              atgaagcttactagtatagctattttggcatttttgaggtatgttaat-cccttctacacgtctgttagcatt-----acctaaagca-ctatt-gtagcgctaccgccactgcccagac
    GCA_025217045.1#JAMSFA010000726.1_112-560              atgaagcttactagtatagctattttggcatttttgaggtatgttaat-cccttcgacacgtctgttagcatt-----atctaaagca-ctatt-gtagcgctaccgccactgcgcagac
    GCA_027886205.1#RZGM01000180.1_8903-9341               atgaagattactagcattgctattttggcatttctaaggtatgttagt-cccttctacacgcctgtcagcatt-----atctaaagca-ccatt-atagcgccaccgtcactgcgcagac
    GCA_027945405.1#JAOQBI010000031.1_402171-402615        atgaagattactagcatagctattttggcatttttgaggtatgttaat-cccttctacacgtccgtcagcatt-----atctaaagca-ctatt-gtagcgctaccgccactgcgcagac
    GCA_027945555.1#JAOQAY010000137.1_8199-8643            atgaagattactagcatagctattttggcatttttgaggtatgttaat-cccttctacacgtccgtcagcatt-----atctaaagca-ctatt-gtagcgctaccgccactgcgcagac
    GCA_027946385.1#JAPEUR010000436.1_787-1225             atgaagattactagcattgctattttggcatttctaaggtatgttagt-cccttctacacgcctgtcagcatt-----atctaaagca-ccatt-atagcgccaccgtcactgcgcagac
    GCA_029607815.1#JAKCXH010001036.1_885-1334             atgaagatgacaatcatagctactttggcgcttttgaggtacttcgat-accttctgtattatcagtatcttt-----gaataa--ca-ttatt-atagcgccagcgtcactgctcaaac
    GCA_030064525.1#JARFYI010000012.1_2234554-2235001      atgaagcttactagcatagctattttggcatttttgaggtatgttaa--cccttcgacacgtctattagcatc-----ttctaaagca-ctatt-atagcgctactgtcactgcgcagac
    GCA_032884055.1#JAULRZ010000496.1_1817-2265            atgaagattactagcatagctattttggcatttttgaggtatgttaat-cccttctacacgtctgttagcatt-----atctaaagca-ctatt-gtagcgctactgccactgcgcagac
    GCA_033782975.1#JAWJZW010004347.1_1810-2253            atgaagattactagcatagctattttggcgtttttgaggtatgttaat-cccttcgacacgtctgttagcatt-----atctagagca-caatt-gtagcgctactgccactgcgcagac
    GCA_033782995.1#JAWJZX010004021.1_7189-7632            atgaggattactagcatagctattttggcgtttttgaggtatgttaat-cccttcgacacgtctgttagcatt-----atctagagca-caatt-gtagcgctactgctactgcgcagac
    GCA_034642005.1#JAWNYV010000013.1_1595757-1596200      atgaagattactagcatagctattttggcgtttttgaggtatgttaat-cccttcgacacgtctgttagcatt-----atctagagca-caatt-gtagcgctactgccactgcgcagac
    GCA_035773055.1#JAPMJO010000013.1_58458-58906          atgaagcttactagcatagctattttggcatttttaaggtatattcat-cccttcgacacgtctattagcatc-----atctgaagta-ttatt-atagcgctactgccactgcgcagac
    GCA_037126525.1#JAZDSW010000046.1_287452-287824        atgaagattactagcatagctattttggcatttctggggtatgttaat-cccttctacacgtctgttagcatt-----atctaaagca-ctatt-gtagcgctatctccactgcgcagac
    GCA_038502765.1#JALBXV010000004.1_150133-150579        atgaagcttaccagcatagctattttggcatttttgaggtatgttaat-cccttcgacacgcctgttagcatt-----atctaaagca-ctatt-gtagcgctactgccactgcgcagac
    GCA_038502765.1#JALBXV010000003.1_1183198-1183644      atgaagcttaccagcatagctattttggcatttttgaggtatgttaat-cccttcgacacgcctgttagcatt-----atctaaagca-ctatt-gtagcgctactgccactgcgcagac
    GCA_040938085.1#JBDQYN010000095.1_4849420-4849866      atgaagatcattagcatagctgttttggcatttttgaggtatgttagt-cttttcaacatgtttgttagtgtt-----aactaaagca-ctatt-atagcgctacagctactgcgcagac
    GCA_041380375.1#JAZEUI010000212.1_778435-778881        atgaagatcattagcatagctgttttggcatttttgaggtatgttagt-cttttcaacatgtttgttagtgtt-----aactaaagca-ctatt-atagcgctacagctactgcgcagac
    GCA_041380545.1#JAZEUM010000394.1_2965598-2966044      atgaagatcattagcatagctgttttggcatttttgaggtatgttagt-cttttcaacatgtttgttagtgtt-----aactaaagca-ctatt-atagcgctacagctactgcgcagac
    GCF_000271745.1#NW_022158520.1_5103546-5103992         atgaagatcattagcatagctgttttggcatttttgaggtatgttagt-cttttcaacatgtttgttagtgtt-----aactaaagca-ctatt-atagcgctacagctactgcgcagac
    GCF_020744475.1#NW_025763681.1_315043-315491           atgaagattactagcatagctattttggcatttttgaggtatgttaat-cccttctacacgtctgttagcatt-----atttaaagca-ctatt-gtagcgctactgctactgcgcagac

    Selected Cols:                                                                                                                                                                 

    Gaps Scores:                                                                                                                                                                   
    Similarity Scores:                                                                                                                                                             

                                                                  130       140       150       160       170       180       190       200       210       220       230       240
                                                           =========+=========+=========+=========+=========+=========+=========+=========+=========+=========+=========+=========+
    GCA_000260075.2#JH651001.1_53017-53465                 cggttcaaaatggctgagctattgtaagggtgcatcacaggatctaggccagtccctctgtaagaagaaggggggcacctgggggcctcgtacagacgctccagcggaatacaagagccg
    GCA_000271745.2#JH717840.1_5103546-5103992             cggctcaaaatggctgagctactgtaagggcgcatcacaagatctaggccagtccctctgcaagaagaaggggggcacctgggggcctcgtacagacgttccagcagaatacaggagccg
    GCA_000400815.2#CP009080.1_1179336-1179785             agggccaaagtacctgaactattgtaagggagtatcacaagatctcggcaaatccctctgtaggaagcatcacggaacctggggccagcggacagatgtcccaccgggttatgtaagccg
    GCA_000585705.1#AEYB01001584.1_5717-6165               cggttcgaaatggctgagctattgtaagggcacatctcaggatctaggcgaatccctctgtgcgaagaagggtggcagctgggggccccgtacagacgccccagatcaatacaagagccg
    GCA_001599515.1#BCHB01000008.1_128823-129266           cggctcaaaatggctgagctattgtaagggagtatcacaggatctaggccaatccctatgtaagaagaaggggggcacctgggggcctcgtacagatgctccagcggaatacagaagccg
    GCA_001680525.1#MADZ01000944.1_3517-3965               cggttcgaaatggctgagctattgtaagggcacatctcaggatctaggcgaatccctctgtgcgaagaagggtggcagctgggggccccgtacagacgccccagatcaatacaagagccg
    GCA_001680535.1#MAED01002951.1_1336-1785               cggttcgaaatggctgagctattgtaagggcacatctcaggatctaggcgaatccctctgtgcgaagaagggtggcagctgggggcctcgtacagatgccccagataaatacaagagccg
    GCA_001680595.1#MAEE01000161.1_9576-10025              cggttcgaaatggctgagctattgtaagggcacatctcaggatctaggcgaatccctctgtgcgaagaagggtggcagctgggggcctcgtacagatgccccagatcaatacaagagccg
    GCA_001680605.1#MADY01000512.1_3407-3855               cggttcgaaatggctgagctattgtaagggcacatctcaggatctaggcgaatccctctgtgcgaagaagggtggcagctgggggccccgtacagacgccccagatcaatacaagagccg
    GCA_001680665.1#MADX01001027.1_5081-5529               cggttcgaaatggctgagctattgtaagggcacatctcaggatctaggcgaatccctctgtgcgaagaagggtggcagctgggggccccgtacagacgccccagatcaatacaagagccg
    GCA_001680685.1#MAEC01002105.1_1371-1819               cggttcgaaatggctgagctattgtaagggcacatctcaggatctaggcgaatccctctgtgcgaagaagggtggcagctgggggcctcgtacagacgccccagatcaatacaagagccg
    GCA_001680725.1#MAEF01000026.1_12451-12900             cggttcgaaatggctgagctattgtaagggcacatctcaggatctaggcgaatccctctgtgcgaagaagggtggcagctgggggcctcgtacagatgccccagatcaatacaagagccg
    GCA_001931975.2#MSJJ02000021.1_21498-21944             cggctcaaaatggctgagctactgtaagggcgcatcacaagatctaggccagtccctctgcaagaagaaggggggcacctgggggcctcgtacagacgttccagcagaatacaggagccg
    GCA_002233775.1#NJCV01000665.1_343-790                 aggctcaaaatggctgagctactgtaagggcgcatcacaggatctaggcaagtccctctgtaagaagaaggggggcacct-ggggcctcgtacagacgctccagcgggatacaggagccg
    GCA_002233895.1#NJCL01000052.1_6292-6740               cggttcaaaatatctgagctattgtaagggccaatcacaggatctaggccagtccctgtgtaagaagaaggggggcacctgggggccccgtacagacgctccagcggaatacaggagccg
    GCA_002233895.1#NJCL01000986.1_2608-3056               cggtccaaaatatctgaactattgtaagggcgcatcacaggatctaggcgagtccctgtgtaagaagaaggggggcacctggggccctcgtacagacgttccagaggaatacaggagccg
    GCA_002233895.1#NJCL01000862.1_714-1162                cggttcaaaatggctgggctattgtaagggcacatcacaggatctaggcgagtccctgtgtaagaagaaggggggcacctgggggcctcgtacagacgttccagtgaaatacaagagccg
    GCA_002233915.1#NJCM01002567.1_585-1030                cggtccaaaatatctgaactattgtaagggcgcatcacaggatctaggcaagtccctgtgtaaaaagaagggtggcacctggggccctcgtacagatgttccagaggaatacaggagccg
    GCA_002233915.1#NJCM01000349.1_3242-3690               cggtccaaaatatctgaactattgtaagggcgcatcacaggatctaggcgagtccctgtgtaagaagaaggggggcacctggggccctcgtacagacgttccagaggaatacaggagccg
    GCA_002233935.2#NJBT02000074.1_114534-114981           aggctcaaaatggctgagctactgtaagggcgcatcacaggatctaggcaagtccctctgtaagaagaaggggggcacctgggggcctcgtacggacgctccagcgggatacaggagccg
    GCA_002234115.1#NJCF01001371.1_90-531                  cggttcaaaatatctgagctattgtaagggcacatcacaggatctaggccagtccctgtgtaagaagaaagggggcacctgggggccccgtacagacgctccagcggaatacaggagccg
    GCA_002234115.1#NJCF01000302.1_3435-3883               cggttcaaaatggctgggctattgtaagggcacatcacaggatctaggcgagtccctgtgtaagaagaaggggggcacctgggggcctcgtacagacgttccagtgaaatacaagagccg
    GCA_002234115.1#NJCF01000961.1_5490-5938               cggttcaaaatatctgagctattgtaagggccaatcacaggatctaggccagtccctgtgtaagaagaaggggggcacctgggggccccgtacagacgctccagcggaatacaggagccg
    GCA_002234195.1#NJCK01002649.1_264-712                 cggtccaaaatatctgaactattgtaagggcgcatcacaggatctaggcgagtccctgtgtaagaagaaggggggcacctggggccctcgtacagacgttccagaggaatacaggagccg
    GCA_002234205.1#NJCQ01003026.1_1778-2226               cggttcaaaatatctgagctattgtaagggcacatcacaggatctaggccagtccctctgtaagaagaaggggggcacctgggggccccgtacagacgctccagcggaatacaggagccg
    GCA_002234205.1#NJCQ01001471.1_358-806                 cggttcaaaatacctgagctattgtaagggcacatcacaggatctaggcaagtccctctgtaagaagaagggaggcacctgggggcctcgtacagacgctccagcggaatacaggagccg
    GCA_002234235.1#NJCR01003189.1_7833-8281               cggttcaaaatatctgagctattgtaagggcacatcacaggatctaggccagtccctctgtaagaagaaggggggcacctgggggccccgtacagacgctccagcggaatacaggagccg
    GCA_002234235.1#NJCR01000523.1_1652-2100               cggttcaaaatacctgagctattgtaagggcacatcacaggatctaggcaagtccctctgtaagaagaagggaggcacctgggggcctcgtacagacgctccagcggaatacaggagccg
    GCA_002234255.1#NJCS01000197.1_9074-9523               cggctcaaaatgggtaagctactataaggccgtattacaggatctaggcgaatccctctgtaagaaaaaggggggtacctgggggccttgtacagacgctctagcggaatacaggagcag
    GCA_002234255.1#NJCS01000257.1_23088-23528             cggcttaaaatgggtgagctactgtaaggctgtattacaggatctaggcgaatctttctgtaagaagaaggggggtacctaggggccttgtatagacgctccagcggaatacaggagtag
    GCA_002234285.1#NJCT01000251.1_2006-2454               cggttcaaaatggctgggctattgtaagggcacatcacaggatctaggcgagtccctgtgtaagaagaaggggggcacctgggggcctcgtacagacgttccagtgaaatacaagagccg
    GCA_002234285.1#NJCT01000112.1_2108-2556               cggttcaaaatatctgagctattgtaagggccaatcacaggatctaggccagtccctgtgtaagaagaaggggggcacctgggggccccgtacagacgctccagcggaatacaggagccg
    GCA_002776445.1#PDEZ01000231.1_7918-8368               agggccaaagtacctgaactattgtaagggagtatcacaagatctcggcaaatccctctgtaggaagcatcacggaacctggggccagcggacagatgtcccaccgggttatgtaagccg
    GCA_002892985.1#MPSH01000001.1_2159685-2160134         agggccaaagtacctgaactattgtaagggagtatcacaagatctcggcaaatccctctgtaggaagcatcacggaacctggggccagcggacagatgtcccaccgggttatgtaagccg
    GCA_002893025.1#PHNV01000722.1_4459-4908               agggccaaagtacctgaactattgtaagggagtatcacaagatctcggcaaatccctctgtaggaagcatcacggaacctggggccagcggacagatgtcccaccgggttatgtaagccg
    GCA_002893035.1#PHNW01000920.1_4459-4908               agggccaaagtacctgaactattgtaagggagtatcacaagatctcggcaaatccctctgtaggaagcatcacggaacctggggccagcggacagatgtcccaccgggttatgtaagccg
    GCA_002982035.1#PVPY01001450.1_5032-5474               cggttcaaaatggctaagctattgtaagggagtatcacaggatctaggccaatccttctgtaagaagaaggggggcacctgggggcctcgtacagacgctccagcgcaatacaggagccg
    GCA_002982055.1#PVPZ01001881.1_721-1163                cggttcaaaatggctgagctattgtaagggagtatcacaggatctaggccaatccttctgtaagaagaaggggggcacctgggggcctcgtacagacgctccagcgcaatacaggagccg
    GCA_003025205.1#PXUO01000119.1_69286-69732             cggctcaaaatggctgagctactgtaagggcgcatcacaagatctaggccagtccctctgcaagaagaaggggggcacctgggggcctcgtacagacgttccagcagaatacaggagccg
    GCA_003025235.1#PXUN01000132.1_66066-66512             cggctcaaaatggctgagctactgtaagggcgcatcacaagatctaggccagtccctctgcaagaagaaggggggcacctgggggcctcgtacagacgttccagcagaatacaggagccg
    GCA_004109745.1#RSDZ01000158.1_4397-4846               agggccaaagtacctgaactattgtaagggagtatcacaagatctcggcaaatccctctgtaggaagcatcacggaacctggggccagcggacagatgtcccaccgggttatgtaagccg
    GCA_004141715.1#MQTW01000814.1_378-825                 aggctcaaaatggctgagctactgtaagggcgcatcacaggatctaggcaagtccctctgtaagaagaaggggggcacct-ggggcctcgtacagacgctccagcgggatacaggagccg
    GCA_004291455.1#QUWZ01000068.1_80333-80705             ccgagaaaaatggctgagctgttgtaagggcacatggcaggatctaggcgagtccctgtataagaagaaggggggcacctgggggcctcgtacagacgctccagtggtatacaagagccg
    GCA_004292535.1#QUXA01000044.1_77168-77540             ccgagaaaaatggctgagctgttgtaagggcacatggcaggatctaggcgagtccctgtataagaagaaggggggcacctgggggcctcgtacagacgctccagtggtatacaagagccg
    GCA_009297365.1#WGOL01000900.1_612-1060                cggttcaaaatggctgagctattgtaagggcacatcacaggatctaggcgagtccctgtgtaagaagaaggggggcacctgggggcctcgtacagacgctccagcggaatacaggagccg
    GCA_009297515.1#WGOI01001607.1_1477-1925               cggttcaaaatggctgagctattgtaagggtgcatcacaggatctaggccagtcactctgtaagaagaaggggggcacctgggggcctcgtacagacgctccagcggaatacaggagccg
    GCA_009297675.1#WGOJ01003344.1_1223-1671               cggttcaaaatggctgagctattgtaagggtgcatcacaggatctaggccagtccctctgtaagaagaagggcggcacctgggggcctcgtacagacgctccagcggaatacaggagccg
    GCA_009298545.1#WGQK01000668.1_4136-4584               cggttcaaaatggctgagctattgtaagggcacatcacaggatctaggccagtccctctgtaagaagaaggggggcacctgggggcctcgtacagacgctccagcggaattcaagagccg
    GCA_009298855.1#WGQN01001540.1_1461-1906               cggttcaaaatggctgagctattgtaagggtgcatcacaggatctaggccagtccctctgtaagaagaaggggggcacctgggggcctcgtacagacgctccagcggaatacaggagccg
    GCA_009298985.1#WGRN01000796.1_1047-1495               cggttcaaaatggctgagctattgtaagggtgcatcacaggatctaggccagtccctctgtaagaagaaggggggcacctgggggcctcgtacagacgctccagcggaatacaggagccg
    GCA_009299045.1#WGRK01001155.1_826-1274                cggttcaaaatggctgagctattgtaagggcgcatcacaggatctaggccagtccctctgtaagaagaaggggggcacctgggggcctcgtacagacgctccagcggaataccggagccg
    GCA_009299075.1#WGRJ01000863.1_12409-12855             cggctcaaaatggctgagctactgtaagggcgcatcacaagatctaggccagtccctctgcaagaagaaggggggcacctgggggcctcgtacagacgttccagcagaatacaggagccg
    GCA_009299115.1#WGRH01001009.1_1459-1907               cggttcaaaatggctgagctattgtaagggcacatcacaggatctaggccagtccctctgtaagaagaaggggggcacctgggggcctcgtacagacgctccagcggaattcaagagccg
    GCA_009299215.1#WGRS01000248.1_42092-42540             cggttcaaaatggctgagctattgtaagggtgcatcacaggatctaggccagtccctctgtaagaagaaggggggcacctgggggcctcgtacagacgctccagcggaatacaagagccg
    GCA_009299215.1#WGRS01000488.1_939-1388                cggttcaaaatggctgagctattgtaagggtgcatcacaggatctaggccagtccctctgtaagaagaaggggggcacctgggggcctcgtacagacgctccagcggaatacaggagccg
    GCA_009299215.1#WGRS01000332.1_7989-8437               cggttcaaaatggctgagctattgtaagggtgcatcacaggatctaggccagtccctctgtaagaagaagggcggcacctgggggcctcgtacagacgctccagcggaatacaggagccg
    GCA_009663885.1#WJDV01000002.1_418952-419401           agggccaaagtacctgaactattgtaagggagtatcacaagatctcggcaaatccctctgtaggaagcatcacggaacctggggccagcggacagatgtcccaccgggttatgtaagccg
    GCA_009746015.1#VLJC01000049.1_70684-71130             cggctcaaaatggctgagctactgtaagggcgcatcacaagatctaggccagtccctctgcaagaagaaggggggcacctgggggcctcgtacagacgttccagcagaatacaggagccg
    GCA_011032855.1#WESG01000339.1_10823-11271             cggttcaaaatggctgagctattgtaagggcgcatcacaggatctaggcgagtccctgtgtaagaagaaggggggcacctgggggcctcgtacagacgctccagcggaatacaggagccg
    GCA_011032945.1#WNXM01001784.1_7328-7777               agggccaaagtacctgaactattgtaagggagtatcacaagatctcggcaaatccctctgtaggaagcatcacggaacctggggccagcggacagatgtcccaccgggttatgtaagccg
    GCA_011032955.1#WNXL01001437.1_4250-4699               agggccaaagtacctgaactattgtaagggagtatcacaagatctcggcaaatccctctgtaggaagcatcacggaacctggggccagcggacagatgtcccaccgggttatgtaagccg
    GCA_011032965.1#WNXK01000961.1_4382-4831               agggccaaagtacctgaactattgtaagggagtatcacaagatctcggcaaatccctctgtaggaagcatcacggaacctggggccagcggacagatgtcccaccgggttatgtaagccg
    GCA_011033455.1#WESL01002533.1_4420-4868               cggttcaaaatggctgagctattgtaagggcgcatcacaggatctaggccagtccctctgtaagaagaaggggggcacctgggggcctcgtacagacgctccagcggaataccggagccg
    GCA_011033455.1#WESL01001216.1_8955-9403               cggttcaaaatatctgagctattgtaagggcacatcacaggatctaggccagtccctctgtaagaagaaggggggcacctgggggccccgtacagacgctccagcggaatacaggagccg
    GCA_011033575.1#WESJ01009652.1_394-842                 cggttcaaaatatctgagctattgtaagggcacatcacaggatctaggccagtccctctgtaagaagaaggggggcacctgggggccccgtacagacgctccagcggaatacaggagccg
    GCA_011033575.1#WESJ01007920.1_731-1149                cggttcaaaatggctgagctattgtaagggcgcatcacaggatctaggccagtccctctgtaagaagaaggggggcacctgggggcctcgtacagacgctccagcggaataccggagccg
    GCA_011033685.1#WESM01008445.1_1386-1834               cggttcaaaatatctgagctattgtaagggcacatcacaggatctaggccagtccctctgtaagaagaaggggggcacctgggggcctcgtacagacgctccagcggaataccggagccg
    GCA_011033745.1#WETB01002153.1_7139-7587               cggttcaaaatggctgagttattgtaagggcgcatcacaggatctaggccagtccctctgtaagaagaaggggggcacctgggggcctcgtacagacgctccagcggaataccggagccg
    GCA_011033805.1#WESY01002113.1_415-863                 cggttcaaaatggctgagttattgtaagggcgcatcacaggatctaggccagtccctctgtaagaagaaggggggcacctgggggcctcgtacagacgctccagcggaataccggagccg
    GCA_011033815.1#WESW01000773.1_2707-3155               cggttcaaaatggctgagctattgtaagggcgcatcacaggatctaggccagtccctctgtaagaagaaggggggcacctgggggcctcgtacagacgctccagcggaataccggagccg
    GCA_011033815.1#WESW01000649.1_851-1299                cggttcaaaatatctgagctattgtaagggcacatcacaggatctaggccagtccctctgtaagaagaaggggggcacctgggggccccgtacagacgctccagcggaatacaggagccg
    GCA_011033895.1#WETL01000467.1_850-1298                cggttcaaaatatctgagctattgtaagggcacatcacaggatctaggccagtccctctgtaagaagaaggggggcacctgggggccccgtacagacgctccagcggaatacaggagccg
    GCA_011033895.1#WETL01000641.1_3860-4278               cggttcaaaatggctgagctattgtaagggcgcatcacaggatctaggccagtccctctgtaagaagaaggggggcacctgggggcctcgtacagacgctccagcggaataccggagccg
    GCA_011034195.1#WETT01000020.1_547-995                 cggttcaaaatggctgagttattgtaagggcgcatcacaggatctaggccagtccctctgtaagaagaaggggggcacctgggggcctcgtacagacgctccagcggaataccggagccg
    GCA_011034205.1#WETS01005722.1_2442-2890               cggttcaaaatggctgagttattgtaagggcgcatcacaggatctaggccagtccctctgtaagaagaaggggggcacctgggggcctcgtacagacgctccagcggaataccggagccg
    GCA_011034415.1#WEUC01001584.1_7719-8167               cggttcaaaatggctgagttattgtaagggcgcatcacaggatctaggccagtccctctgtaagaagaaggggggcacctgggggcctcgtacagacgctccagcggaataccggagccg
    GCA_011034445.1#WEUB01001158.1_3523-3971               cggttcaaaatggctgagctattgtaagggcgcatcacaggatctaggccagtccctctgtaagaagaaggggggcacctgggggcctcgtacagacgctccagcggaataccggagccg
    GCA_011034445.1#WEUB01000999.1_8836-9284               cggttcaaaatatctgagctattgtaagggcacatcacaggatctaggccagtccctctgtaagaagaaggggggcacctgggggccccgtacagacgctccagcggaatacaggagccg
    GCA_011034575.1#WEUE01008972.1_493-941                 cggttcaaaatatctgagctattgtaagggcacatcacaggatctaggccagtccctctgtaagaagaaggggggcacctgggggccccgtacagacgctccagcggaatacaggagccg
    GCA_011034625.1#WEUR01000714.1_7159-7607               cggttcaaaatggctgagttattgtaagggcgcatcacaggatctaggccagtccctctgtaagaagaaggggggcacctgggggcctcgtacagacgctccagcggaataccggagccg
    GCA_011034645.1#WEUV01000741.1_2997-3445               cggttcaaaatatttgagctattgtaagggcacatcacaggacctaggccagtctctgtgtaagaagaaggggggcagctgggggcctcgtacagacgctccagcggaattcaggagccg
    GCA_011034655.1#WEUQ01004890.1_3105-3553               cggttcaaaatggctgagttattgtaagggcgcatcacaggatctaggccagtccctctgtaagaagaaggggggcacctgggggcctcgtacagacgctccagcggaataccggagccg
    GCA_011034655.1#WEUQ01000935.1_566-1014                cggttcaaaatggctgagctattgtaagggcgcatcacaggatctaggcgagtccctgtgtaagaagaaggggggcacctgggggcctcgtacagacgctccagcggaatacaggagccg
    GCA_011034775.1#WEVI01000501.1_5488-5930               cggttcaaaatatctgagctattgtaagggcacatcacaggatctaggccagtccctctgtaagaagaaggggggcacctgggggccccgtacagacgctccagcggaatacaggagccg
    GCA_011034775.1#WEVI01000783.1_2046-2494               cggttcaaaatggctgagctattgtaagggcgcatcacaggatctaggccagtccctctgtaagaagaaggggggcacctgggggcctcgtacagacgctccagcggaataccggagccg
    GCA_011034815.1#WEVE01000912.1_9755-10173              cggttcaaaatatctgagctattgtaagggcacatcacaggatctaggccagtccctctgtaagaagaaggggggcacctgggggccccgtacagacgctccagcggaatacaggagccg
    GCA_011034815.1#WEVE01002086.1_1620-2038               cggttcaaaatggctgagctattgtaagggcgcatcacaggatctaggccagtccctctgtaagaagaaggggggcacctgggggcctcgtacagacgctccagcggaataccggagccg
    GCA_011034845.1#WEVA01001230.1_1747-2195               cggttcaaaatggctgagttattgtaagggcgcatcacaggatctaggccagtccctctgtaagaagaaggggggcacctgggggcctcgtacagacgctccagcggaataccggagccg
    GCA_011035075.1#WEVS01001164.1_694-1142                cggttcaaaatggctgagttattgtaagggcgcatcacaggatctaggccagtccctctgtaagaagaaggggggcacctgggggcctcgtacagacgctccagcggaataccggagccg
    GCA_011035185.1#WEVO01012916.1_426-874                 cggttcaaaatggctgagctattgtaagggcgcatcacaggatctaggccagtccctctgtaagaagaaggggggcacctgggggcctcgtacagacgctccagcggaataccggagccg
    GCA_011035185.1#WEVO01003306.1_4368-4816               cggttcaaaatatctgagctattgtaagggcacatcacaggatctaggccagtccctctgtaagaagaaggggggcacctgggggccccgtacagacgctccagcggaatacaggagccg
    GCA_011035205.1#WEVG01003463.1_75-523                  cggttcaaaatggctgagttattgtaagggcgcatcacaggatctaggccagtccctctgtaagaagaaggggggcacctgggggcctcgtacagacgctccagcggaataccggagccg
    GCA_011035235.1#WEWM01001159.1_2667-3085               cggttcaaaatggctgagctattgtaagggcgcatcacaggatctaggccagtccctctgtaagaagaaggggggcacctgggggcctcgtacagacgctccagcggaataccggagccg
    GCA_011035235.1#WEWM01001540.1_2265-2683               cggttcaaaatatctgagctattgtaagggcacatcacaggatctaggccagtccctctgtaagaagaaggggggcacctgggggccccgtacagacgctccagcggaatacaggagccg
    GCA_011035245.1#WEWK01000657.1_7022-7440               cggttcaaaatggctgagttattgtaagggcgcatcacaggatctaggccagtccctctgtaagaagaaggggggcacctgggggcctcgtacagacgctccagcggaataccggagccg
    GCA_011035245.1#WEWK01000404.1_16648-17029             cggttcaaaatggctgagctattgcaagggtgcatcacaggatctaggccagtccctctgtaagaagaaggggggcacctgggagcctcgtacagacgctccagcggaatggaggagccg
    GCA_011035255.1#WEWN01000527.1_2412-2830               cggttcaaaatggctgagctattgtaagggcgcatcacaggatctaggccagtccctctgtaagaagaaggggggcacctgggggcctcgtacagacgctccagcggaataccggagccg
    GCA_011035255.1#WEWN01000751.1_2320-2738               cggttcaaaatatctgagctattgtaagggcacatcacaggatctaggccagtccctctgtaagaagaaggggggcacctgggggccccgtacagacgctccagcggaatacaggagccg
    GCA_011035265.1#WEWJ01000412.1_17167-17548             cggttcaaaatggctgagctattgcaagggtgcatcacaggatctaggccagtccctctgtaagaagaaggggggcacctgggagcctcgtacagacgctccagcggaatggaggagccg
    GCA_011035265.1#WEWJ01000569.1_529-977                 cggttcaaaatggctgagttattgtaagggcgcatcacaggatctaggccagtccctctgtaagaagaaggggggcacctgggggcctcgtacagacgctccagcggaataccggagccg
    GCA_011035345.1#WEWE01000725.1_7055-7503               cggttcaaaatggctgagttattgtaagggcgcatcacaggatctaggccagtccctctgtaagaagaaggggggcacctgggggcctcgtacagacgctccagcggaataccggagccg
    GCA_011035345.1#WEWE01000822.1_176-557                 cggttcaaaatggctgagctattgcaagggtgcatcacaggatctaggccagtccctctgtaagaagaaggggggcacctgggagcctcgtacagacgctccagcggaatggaggagccg
    GCA_011035375.1#WEWB01000636.1_176-557                 cggttcaaaatggctgagctattgcaagggtgcatcacaggatctaggccagtccctctgtaagaagaaggggggcacctgggagcctcgtacagacgctccagcggaatggaggagccg
    GCA_011035375.1#WEWB01000586.1_665-1113                cggttcaaaatggctgagttattgtaagggcgcatcacaggatctaggccagtccctctgtaagaagaaggggggcacctgggggcctcgtacagacgctccagcggaataccggagccg
    GCA_011035435.1#WEWD01000787.1_1777-2225               cggttcaaaatggctgagttattgtaagggcgcatcacaggatctaggccagtccctctgtaagaagaaggggggcacctgggggcctcgtacagacgctccagcggaataccggagccg
    GCA_011035455.1#WEVZ01000295.1_5889-6270               cggttcaaaatggctgagctattgcaagggtgcatcacaggatctaggccagtccctctgtaagaagaaggggggcacctgggagcctcgtacagacgctccagcggaatggaggagccg
    GCA_011035485.1#WEVY01000687.1_5745-6126               cggttcaaaatggctgagctattgcaagggtgcatcacaggatctaggccagtccctctgtaagaagaaggggggcacctgggagcctcgtacagacgctccagcggaatggaggagccg
    GCA_011035485.1#WEVY01000540.1_528-976                 cggttcaaaatggctgagttattgtaagggcgcatcacaggatctaggccagtccctctgtaagaagaaggggggcacctgggggcctcgtacagacgctccagcggaataccggagccg
    GCA_011035495.1#WEVW01000533.1_1949-2397               cggttcaaaatggctgagttattgtaagggcgcatcacaggatctaggccagtccctctgtaagaagaaggggggcacctgggggcctcgtacagacgctccagcggaataccggagccg
    GCA_011035625.1#WEWS01001096.1_809-1227                cggttcaaaatatctgagctattgtaagggcacatcacaggatctaggccagtccctctgtaagaagaaggggggcacctgggggccccgtacagacgctccagcggaatacaggagccg
    GCA_011035625.1#WEWS01001310.1_2416-2834               cggttcaaaatggctgagctattgtaagggcgcatcacaggatctaggccagtccctctgtaagaagaaggggggcacctgggggcctcgtacagacgctccagcggaataccggagccg
    GCA_011035755.1#WEXC01000589.1_22209-22657             cggttcaaaatggctgagctattgtaagggcgcatcacaggatctaggcgagtccctgtgtaagaagaaggggggcacctgggggcctcgtacagacgctccagcggaatacaggagccg
    GCA_011035835.1#WEXF01000545.1_8821-9239               cggttcaaaatatctgagctattgtaagggcacatcacaggatctaggccagtccctctgtaagaagaaggggggcacctgggggccccgtacagacgctccagcggaatacaggagccg
    GCA_011035835.1#WEXF01000653.1_3838-4256               cggttcaaaatggctgagctattgtaagggcgcatcacaggatctaggccagtccctctgtaagaagaaggggggcacctgggggcctcgtacagacgctccagcggaataccggagccg
    GCA_011035975.1#WEXJ01007704.1_1039-1487               cggttcaaaatgtctgagctattgtaagggcgcatcacaggatctaggccagtccctctgtaagaagaaggggggcacctgggggcctcgtacagacgctccagcggaataccggagccg
    GCA_011035995.1#WEXI01000903.1_7456-7904               cggttcaaaatggctgagttattgtaagggcgcatcacaggatctaggccagtccctctgtaagaagaaggggggcacctgggggcctcgtacagacgctccagcggaataccggagccg
    GCA_011035995.1#WEXI01001093.1_807-1255                cggttcaaaatggctgagctattgtaagggcgcatcacaggatctaggcgagtccctgtgtaagaagaaggggggcacctgggggcctcgtacagacgctccagcggaatacaggagccg
    GCA_011036045.1#WEXT01000869.1_163-611                 cggttcaaaatggctgagctattgtaagggtgcatcgcaggatctaggccagtccctctgtaagaagaaggggggcacctgggggcctcgtacagacgctccagcggaatacaggagccg
    GCA_011036135.1#WEXQ01000241.1_21717-22165             cggttcaaaatggctgagctattgtaagggcgcatcacaggatctaggcgagtccctgtgtaagaagaaggggggcacctgggggcctcgtacagacgctccagcggaatacaggagccg
    GCA_011036135.1#WEXQ01000570.1_528-976                 cggttcaaaatggctgagttattgtaagggcgcatcacaggatctaggccagtccctctgtaagaagaaggggggcacctgggggcctcgtacagacgctccagcggaataccggagccg
    GCA_011036215.1#WEYG01000053.1_6223-6671               cggttcaaaatggctgagctattgtaagggtgcatcgcaggatctaggccagtccctctgtaagaagaaggggggcacctgggggcctcgtacagacgctccagcggaatacaggagccg
    GCA_011036305.1#WEXY01003662.1_3416-3864               cggttcaaaatggctgagctattgtaagggcgcatcacaggatctaggccagtccctctgtaagaagaaggggggcacctgggggcctcgtacagacgctccagcggaataccggagccg
    GCA_011036305.1#WEXY01002318.1_5546-5994               cggttcaaaatatctgagctattgtaagggcacatcacaggatctaggccagtccctctgtaagaagaaggggggcacctgggggccccgtacagacgctccagcggaatacaggagccg
    GCA_011036365.1#WEYQ01001970.1_647-1095                cggttcaaaatggctgagttattgtaagggcgcatcacaggatctaggccagtccctctgtaagaagaaggggggcacctgggggcctcgtacagacgctccagcggaataccggagccg
    GCA_011036595.1#WEYT01000407.1_15167-15585             cggttcaaaatggctgagctattgtaagggcgcatcacaggatctaggccagtccctctgtaagaagaaggggggcacctgggggcctcgtacagacgctccagcggaataccggagccg
    GCA_011036595.1#WEYT01000705.1_8787-9235               cggttcaaaatatctgagctattgtaagggcacatcacaggatctaggccagtccctctgtaagaagaaggggggcacctgggggccccgtacagacgctccagcggaatacaggagccg
    GCA_011036765.1#WEUS01002153.1_588-1036                cggttcaaaatggctgagttattgtaagggcgcatcacaggatctaggccagtccctctgtaagaagaaggggggcacctgggggcctcgtacagacgctccagcggaataccggagccg
    GCA_011036795.1#WEUZ01000271.1_2987-3435               cggttcaaaatatttgagctattgtaagggcacatcacaggacctaggccagtctctgtgtaagaagaaggggggcagctgggggcctcgtacagacgctccagcggaattcaggagccg
    GCA_011036875.1#WEWI01000026.1_11685-12103             cggttcaaaatatctgagctattgtaagggcacatcacaggatctaggccagtccctctgtaagaagaaggggggcacctgggggccccgtacagacgctccagcggaatacaggagccg
    GCA_011036875.1#WEWI01000910.1_3838-4256               cggttcaaaatggctgagctattgtaagggcgcatcacaggatctaggccagtccctctgtaagaagaaggggggcacctgggggcctcgtacagacgctccagcggaataccggagccg
    GCA_011037075.1#WEZD01015082.1_365-770                 cggttcaaaatggctgagctattgtaagggcgcatcacaggatctaggccagtccctctgtaagaagaaggggggcacctgggggcctcgtacagacgctccagcggaataccggagccg
    GCA_011421275.1#WIKV01000306.1_26907-27325             cggttcaaaatatctgagctattgtaagggcacatcacaggatctaggccagtccctctgtaagaagaaggggggcacctgggggccccgtacagacgctccagcggaatacaggagccg
    GCA_011421275.1#WIKV01000622.1_3838-4256               cggttcaaaatggctgagctattgtaagggcgcatcacaggatctaggccagtccctctgtaagaagaaggggggcacctgggggcctcgtacagacgctccagcggaataccggagccg
    GCA_011421285.1#WIKU01001812.1_697-1145                cggttcaaaatggctgagttattgtaagggcgcatcacaggatctaggccagtccctctgtaagaagaaggggggcacctgggggcctcgtacagacgctccagcggaataccggagccg
    GCA_011421305.1#WIKW01002869.1_809-1227                cggttcaaaatatctgagctattgtaagggcacatcacaggatctaggccagtccctctgtaagaagaaggggggcacctgggggccccgtacagacgctccagcggaatacaggagccg
    GCA_011421305.1#WIKW01002126.1_1789-2207               cggttcaaaatggctgagctattgtaagggcgcatcacaggatctaggccagtccctctgtaagaagaaggggggcacctgggggcctcgtacagacgctccagcggaataccggagccg
    GCA_011421375.1#WILC01001342.1_696-1144                cggttcaaaatggctgagttattgtaagggcgcatcacaggatctaggccagtccctctgtaagaagaaggggggcacctgggggcctcgtacagacgctccagcggaataccggagccg
    GCA_011426335.1#WILD01001610.1_1018-1466               cggttcaaaatggctgagctattgtaagggcgcatcacaggatctaggccagtccctctgtaagaagaaggggggcacctgggggcctcgtacagacgctccagcggaataccggagccg
    GCA_011426355.1#WILF01007384.1_372-820                 cggttcaaaatggctgagctattgtaagggcgcatcacaggatctaggccagtccctctgtaagaagaaggggggcacctgggggcctcgtacagacgctccagcggaataccggagccg
    GCA_011426355.1#WILF01000505.1_6553-7001               cggttcaaaatatctgagctattgtaagggcacatcacaggatctaggccagtccctctgtaagaagaaggggggcacctgggggcctcgtacagacgctccagcggaataccggagccg
    GCA_013170945.1#MU047874.1_170025-170474               agggccaaagtacctgaactattgtaagggagtatcacaagatctcggcaaatccctctgtaggaagcatcacggaacctggggccagcggacagatgtcccaccgggttatgtaagccg
    GCA_013184365.1#JABCJX010000598.1_3113-3561            cggttcaaaatggctgagctattgtcagggcgtatcacaggatctaggcgagtccctctgtaagaagaaggggggcacctgggggcctcgtacagacccttcagcggaatacagaagccg
    GCA_013266185.1#JABEXW010001135.1_3715-4163            tggttcaaaatggctgagctattgtaagggcgcatcacaggatctgggccagtccctctgtaagaagaaggggggcacctgggggcctcgtacagacgctccagcggaatacaagagccg
    GCA_013347355.2#JAALGN020000013.1_426461-426909        cggttcaaaatggctgagctattgtaagggtgcatcacaggatctaggccagtccctctgtaagaagaaggggggcacctgggggcctcgtacagacgctccagcggaatacaggagccg
    GCA_013363175.1#JABEEP010001877.1_2080-2528            cggttcgaaatggctgagctattgtaagggcacatctcaggatctaggcgaatccctctgtgcgaagaagggtggcagctgggggcctcgtacagacgccccagatcaatacaagagccg
    GCA_013396025.1#JAAOAQ010001214.1_4450-4898            cggtccaaaatatctgaactattgtaagggaacatcacaggatctaggcgagtccctgtgtaagaagaagggtggcacctggggccctcgtacagacgttccagcggaataccggagccg
    GCA_013396205.1#JAAOAN010000160.1_45920-46352          cggttcaaagtacctgagctattgtaagggagtgtcacaggatctaggcaaatccctctgcaagaagaagggtggcagctgggggcctcgtaccgacgctccagcggaatacaaaagccg
    GCA_013618355.1#JABFES010000455.1_7017-7460            cggctcaaaatggctgagctattgtaagggagtatcacaggatctaggccaatccctgtgtaagaagaagggggggacctgggggcctcgtacagacgctccagcggaatacagaagccg
    GCA_013623525.1#JABGLY010000028.1_42787-43233          cggcccaaaatggctgagctattgtaagggtgtctcgcaggatctcggcaagaccctctgtaagaagaaggggggcacctgggggcctcgtatagacgttccagacgattataagagccg
    GCA_013623715.1#JABFFJ010000182.1_85034-85470          tggctcaaaatacctgagttattgtcagggctcatcgcaggatctaggccagtccctctgtcggaagttcaaaggcacctggggccctcgtacagacgttccagagaaatacaaaagccg
    GCA_014324575.1#WXUM01000474.1_1010-1458               cggttcaaaatggctgagctattgtaagggtgcatcacaggatctaggccagtccctctgtaagaagaaggggggcacctgggggcctcgtacagacgctccagcggaatacaggagccg
    GCA_014324595.1#WXUN01001159.1_797-1245                cggttcaaaatggctgagctattgtaagggtgcatcacaggatctaggccagtccctctgtaagaagaaggggggcacctgggggcctcgtacagacgctccagcggaatacaggagccg
    GCA_014324665.1#WXUR01000643.1_5483-5930               cggttcaaaatggctgagctattgtaagggtgcatcacaggatctaggccagtccctctgtaagaagaaggggggcacctgggggcctcgtacagacgctccagcggcatacaggagccg
    GCA_014324745.1#JAAMUY010001820.1_1146-1594            cggttcaaaatggctgagctattgtaagggtgcatcacaggatctaggccagtccctctgtaagaagaaggggggcacctgggggcctcgtacagacgctccagcggaatacaggagccg
    GCA_014324775.1#WXUT01000779.1_3905-4353               cggttcaaaatggctgagctattgtaagggtgcatcacaggatctaggccagtccctctgtaagaagaaggggggcacctgggggcctcgtacagacgctccagcggaatacaggagccg
    GCA_014324795.1#WXUU01000551.1_4342-4789               cggttcaaaatggctgagctattgtaagggtgcatcacaggatctaggccagtccctctgtaagaagaaggggggcacctgggggcctcgtacagacgctccagcggcatacaggagccg
    GCA_014324835.1#WXUX01001216.1_2785-3233               cggttcaaaatggctgagctattgtaagggtgcatcacaggatctaggccagtccctctgtaagaagaaggggggcacctgggggcctcgtacagacgctccagcggaatacaggagccg
    GCA_014324865.1#WXUY01001109.1_5504-5951               cggttcaaaatggctgagctattgtaagggtgcatcacaggatctaggccagtccctctgtaagaagaaggggggcacctgggggcctcgtacagacgctccagcggcatacaggagccg
    GCA_014324895.1#WXUZ01000915.1_1546-1994               cggttcaaaatggctgagctattgtaagggtgcatcacaggatctaggccagtccctctgtaagaagaaggggggcacctgggggcctcgtacagacgctccagcggaatacaagagccg
    GCA_014325045.1#WXVI01000570.1_8895-9343               cggttcaaaatggctgagctattgtaagggtgcatcacaggatctaggccagtcactctgtaagaagaaggggggcacctgggggcctcgtacagacgctccagcggaatacaggagccg
    GCA_014325205.1#JAAMVE010001426.1_3625-4073            cggttcaaaatggctgagctattgtaagggtgcatcacaggatctaggccagtccctctgtaagaagaaggggggcacctgggggcctcgtacagacgctccagcggaatacaggagccg
    GCA_014325315.1#WXVP01000164.1_88523-88895             ccgagaaaaatggctgagctgttgtaagggcacatggcaggatctaggcgagtccctgtataagaagaaggggggcacctgggggcctcgtacagacgctccagtggtatacaagagccg
    GCA_014843555.1#JAAOOQ010000005.1_239540-239989        cggttcaaaatatctgagctattgcaaaggcacatcacaggatctaggccagtccctctgtaagaagaaggggggcacctgggggccccgtacagacgctccagcggaatacaggagccg
    GCA_014843565.1#JAAOOP010000035.1_171592-172041        cggttcaaaatatctgagctattgcaaaggcacatcacaggatctaggccagtccctctgtaagaagaaggggggcacctgggggccccgtacagacgctccagcggaatacaggagccg
    GCA_014884825.1#JACTAS010000222.1_17723-18171          cggttcaaaatggctgagctattgtaagggcgcatcacaggatctaggcgagtccctgtgtaagaagaaggggggcacctgggggcctcgtacagacgctccagcggaatacaggagccg
    GCA_018440645.1#JACOQZ010000013.1_178443-178892        agggccaaagtacctgaactattgtaagggagtatcacaagatctcggcaaatccctctgtaggaagcatcacggaacctggggccagcggacagatgtcccaccgggttatgtaagccg
    GCA_018440655.1#JACORB010000012.1_54438-54887          agggccaaagtacctgaactattgtaagggagtatcacaagatctcggcaaatccctctgtaggaagcatcacggaacctggggccagcggacagatgtcccaccgggttatgtaagccg
    GCA_019055395.1#JAHKNW010006905.1_873-1321             cggttcaaaatggctgagctattgtaagggtgcatcacaggatctaggccagtccctctgtaagaagaaggggggcacctgggggcctcgtacagacgctccagcggaatacaggagccg
    GCA_019157295.1#JAELUQ010000008.1_3397787-3398230      cggctcaaaatggctgagctattgtaagggagtatcacaggatctaggccaatccctgtgtaagaagaagggggggacctgggggccgcgtacagacgctccagcggaatacagaagccg
    GCA_019191165.1#JAHARQ010000139.1_67224-67670          cggctcaaaatggctgagctactgtaagggcgcatcacaagatctaggccagtccctctgcaagaagaaggggggcacctgggggcctcgtacagacgttccagcagaatacaggagccg
    GCA_019191175.1#JAHARR010000129.1_15625-16071          cggctcaaaatggctgagctactgtaagggcgcatcacaagatctaggccagtccctctgcaagaagaaggggggcacctgggggcctcgtacagacgttccagcagaatacaggagccg
    GCA_019843925.1#JACDIY010000477.1_28970-29342          ccgagaaaaatggctgagctgttgtaagggcacatggcaggatctaggcgagtccctgtataagaagaaggggggcacctgggggcctcgtacagacgctccagtggtatacaagagccg
    GCA_020726535.1#JAHEWJ010000011.1_1000856-1001305      agggccaaagtacctgaactattgtaagggagtatcacaagatctcggcaaatccctctgtaggaagcatcacggaacctggggccagcggacagatgtcccaccgggttatgtaagccg
    GCA_020744475.1#JAGMUX010000018.1_315043-315491        cggttcaaaatggctgagctattgtaagggtgcatcacaggatctaggccagtccctctgtaagaagaaggggggcacctgggggcctcgtacagacgctccagcggaatacaagagccg
    GCA_020883595.1#JAJGQA010000014.1_323964-324412        cggttcgaaatggctgagctattgtaagggcacatctcaggatctaggcgaatccctctgtgcgaagaagggtggcagctgggggccccgtacagacgccccagatcaatacaagagccg
    GCA_020883605.1#JAJHJC010000014.1_396196-396644        cggttcgaaatggctgagctattgtaagggcacatctcaggatctaggcgaatccctctgtgcgaagaagggtggcagctgggggccccgtacagacgccccagatcaatacaagagccg
    GCA_020883615.1#JAJHJD010000017.1_393187-393635        cggttcgaaatggctgagctattgtaagggcacatctcaggatctaggcgaatccctctgtgcgaagaagggtggcagctgggggccccgtacagacgccccagatcaatacaagagccg
    GCA_020883625.1#JAJGQB010000012.1_381552-382000        cggttcgaaatggctgagctattgtaagggcacatctcaggatctaggcgaatccctctgtgcgaagaagggtggcagctgggggccccgtacagacgccccagatcaatacaagagccg
    GCA_021655895.1#JAJJWO010000410.1_1878-2303            cggccaaaagtcgctgagctattgcaagggtgtatcataggacctaggcaagtccctttgtaagaaggaggggggcagctggggacctcatacagacgttccagcgaaccagaagagtcg
    GCA_021730365.1#JAJOOV010000405.1_1213-1662            cggttcgaaatggctgagctattgtaagggcacatctcaggatctaggcgaatccctctgtgcgaagaagggtggcagctgggggcctcgtacagatgccccagataaatacaagagccg
    GCA_023065405.1#JAJTCY010000003.1_74358-74801          cggctcaaaatggctgagctattgtaagggagtatcacaggatctaggccaatccctgtgtaagaagaagggggggacctgggggcctcgtacagacgctccagcggaatacagaagccg
    GCA_023628715.1#JAMAKD010000122.1_67003-67449          cggctcaaaatggctgagctactgtaagggcgcatcacaagatctaggccagtccctctgcaagaagaaggggggcacctgggggcctcgtacagacgttccagcagaatacaggagccg
    GCA_024865645.1#JANKHN010000668.1_8435-8878            cggctcaaaatggctgagctattgtaagggagtatcacaggatctaggccaatccctatgtaagaagaaggggggcacctgggggcctcgtacagatgctccagcggaatacagaagccg
    GCA_025201995.1#JAMSCT010000483.1_2189-2637            cggttcaaaatatctgagctattgtaagggcacatcacaggatctaggccagtccctatgtaagaagaaggggggcacctgggggccccgtacagacgctccagcggaatacaggagccg
    GCA_025203115.1#JAMSFS010000057.1_218973-219344        ccgagaaaaatggctgagctgttgtaagggcacatggcaggatctaggcgagt-cctgtataagaagaaggggggcacctgggggcctcgtacagacgctccagtggtatacaagagccg
    GCA_025203755.1#JAMSFW010000517.1_9896-10342           cggtccgaaatggctgagctattgtaagggcgcatcacaggatctaggcgagtccctgtgtaagaagaaggggggcacctgggggcctcgtacagatactccagcggaatacaggagccg
    GCA_025203755.1#JAMSFW010000664.1_112-560              cggttcaaaatatctgagctattgtaagggcacatcacaggatctaggccagtccctctgtaagaagaaggggggcacctgggggccccgtacagacgctccagcggaatacaggagccg
    GCA_025215475.1#JAMSDB010000983.1_1119-1567            cggttcaaaatggctgagctattgtaagggtgcatcacaggatctaggccagtccctctgtaagaagaaggggggcacctgggggcctcgtacagacgctccagcggaatacaggagccg
    GCA_025215845.1#JAMSCV010001163.1_880-1265             cggttcaaaatggctgagctattgtaagggcgcatcacaggatctaggccagtccctctgtaagaagaagggaggcacctgggggcctcgtacagacgctccagcggaatacaggagccg
    GCA_025215865.1#JAMSCS010000321.1_8136-8584            cggttcaaaatacctgagctattgtaagggcacatcacaggatctaggcaagtccctctgtaagaagaagggaggcacctgggggcctcgtacagacgctccagcggaatacaggagccg
    GCA_025215865.1#JAMSCS010000173.1_620-1068             cggttcaaaatggctgagttattgtaagggcgcatcacaagatctgggccagtccctctgtaagaagaaagggggcacctgggggcctcgtacagacgctccagcggaatacaggagccg
    GCA_025215985.1#JAMSDF010000755.1_3429-3877            cggttcaaaatggctgagctattgtaagggtgcatcacaggatctaggccagtccctctgtaagaagaaggggggcacctgggggcctcgtacagacgctccagcggaatacaggagccg
    GCA_025216965.1#JAMSEY010000527.1_112-558              cggtccgaaatggctgagctattgtaagggcgcatcacaggatctaggcgagtccctgtgtaagaagaaggggggcacctgggggcctcgtacagatactccagcggaatacaggagccg
    GCA_025216965.1#JAMSEY010000698.1_1866-2314            cggttcaaaatatctgagctattgtaagggcacatcacaggatctaggccagtccctctgtaagaagaaggggggcacctgggggccccgtacagacgctccagcggaatacaggagccg
    GCA_025217005.1#JAMSEZ010000669.1_2174-2622            cggttcaaaatatctgagctattgtaagggcacatcacaggatctaggccagtccctctgtaagaagaaggggggcacctgggggccccgtacagacgctccagcggaatacaggagccg
    GCA_025217005.1#JAMSEZ010000515.1_9896-10342           cggtccgaaatggctgagctattgtaagggcgcatcacaggatctaggcgagtccctgtgtaagaagaaggggggcacctgggggcctcgtacagatactccagcggaatacaggagccg
    GCA_025217045.1#JAMSFA010000530.1_112-558              cggtccgaaatggctgagctattgtaagggcgcatcacaggatctaggcgagtccctgtgtaagaagaaggggggcacctgggggcctcgtacagatactccagcggaatacaggagccg
    GCA_025217045.1#JAMSFA010000726.1_112-560              cggttcaaaatatctgagctattgtaagggcacatcacaggatctaggccagtccctctgtaagaagaaggggggcacctgggggccccgtacagacgctccagcggaatacaggagccg
    GCA_027886205.1#RZGM01000180.1_8903-9341               tggttctaaatggctgagctactgtaagagcgtatcacaggatctaggccattccctctgtacgaagaagaaggtcacctgggggcctcgtacagatgttccagtggaatggaagaaccg
    GCA_027945405.1#JAOQBI010000031.1_402171-402615        tggtccaaaatatctgagctattgtaagggcacgtcacaggatctaggccagtccctctgtaagaagaaggggggcacctgggggcctcgtacagacgctccagcggaatacaggagccg
    GCA_027945555.1#JAOQAY010000137.1_8199-8643            tggtccaaaatatctgagctattgtaagggcacgtcacaggatctaggccagtccctctgtaagaagaaggggggcacctgggggcctcgtacagacgctccagcggaatacaggagccg
    GCA_027946385.1#JAPEUR010000436.1_787-1225             tggttctaaatggctgagctactgtaagagcgtatcactggatctaggccattccctctgtacgaagaagaaggtcacctgggggcctcgtacagatgttccagtgaaatggaagaaccg
    GCA_029607815.1#JAKCXH010001036.1_885-1334             agggccaaagtacctgaactattgtaagggagtatcacaagatctcggcaaatccctctgtaggaagcatcacggaacctggggccagcggacagatgtcccaccgggttatgtaagccg
    GCA_030064525.1#JARFYI010000012.1_2234554-2235001      cggatcaaaatggctgagctattgtaagggcgcatcacaggatctaggccagtccctctgtaagaagaagggaggcacctgggggcctcgtacagacgctccagcggaatacaagagccg
    GCA_032884055.1#JAULRZ010000496.1_1817-2265            cggttcaaaatggctgagctattgtaagggtgcatcacaggatctaggccagtccctctgtaagaagaaggggggcacctgggggcctcgtacagacgctccagcggaatacaggagccg
    GCA_033782975.1#JAWJZW010004347.1_1810-2253            cggctcaaaatggctgagctattgtaagggagtatcacaggatctaggccaatccctgtgtaagaagaagggggggacctgggggcctcgtacagacgctccagcggaatacagaagccg
    GCA_033782995.1#JAWJZX010004021.1_7189-7632            cggctcaaaatggctgagctattgtaagggagtatcacaggatctaggccaatccctgtgtaagaagaagggggggacctgggggcctcgtacagacgctccagcggaatacagaagccg
    GCA_034642005.1#JAWNYV010000013.1_1595757-1596200      cggctcaaaatggctgagctattgtaagggagtatcacaggatctaggccaatccctgtgtaagaagaagggggggacctgggggcctcgtacagacgctccagcggaatacagaagccg
    GCA_035773055.1#JAPMJO010000013.1_58458-58906          cggttcaaaatggctgagctattgtaagggcgcatcagaggatctaggccagtccctctgtaagaagaaggggggcacctgggagcctcgtacagacgctccagcggaatacaggagccg
    GCA_037126525.1#JAZDSW010000046.1_287452-287824        ccgagaaaaatggctgagctgttgtaagggcacatggcaggatctaggcgagtccctgtataagaagaaggggggcacctgggggccttgtacagacgctccagtggtatacaagagccg
    GCA_038502765.1#JALBXV010000004.1_150133-150579        cggttccaaatggctgagctattgtaagggagcatcacaggatctaggccagtccctctgtaagaagaaagggggcacctgggggcctcgtacagacgcgccagcggaatacaaaagccg
    GCA_038502765.1#JALBXV010000003.1_1183198-1183644      cggttccaaatggctgagctattgtaagggagcatcacaggatctaggccagtccctctgtaagaagaaagggggcacctgggggcctcgtacagacgcgccagcggaatacaaaagccg
    GCA_040938085.1#JBDQYN010000095.1_4849420-4849866      cggctcaaaatggctgagctactgtaagggcgcatcacaagatctaggccagtccctctgcaagaagaaggggggcacctgggggcctcgtacagacgttccagcagaatacaggagccg
    GCA_041380375.1#JAZEUI010000212.1_778435-778881        cggctcaaaatggctgagctactgtaagggcgcatcacaagatctaggccagtccctctgcaagaagaaggggggcacctgggggcctcgtacagacgttccagcagaatacaggagccg
    GCA_041380545.1#JAZEUM010000394.1_2965598-2966044      cggctcaaaatggctgagctactgtaagggcgcatcacaagatctaggccagtccctctgcaagaagaaggggggcacctgggggcctcgtacagacgttccagcagaatacaggagccg
    GCF_000271745.1#NW_022158520.1_5103546-5103992         cggctcaaaatggctgagctactgtaagggcgcatcacaagatctaggccagtccctctgcaagaagaaggggggcacctgggggcctcgtacagacgttccagcagaatacaggagccg
    GCF_020744475.1#NW_025763681.1_315043-315491           cggttcaaaatggctgagctattgtaagggtgcatcacaggatctaggccagtccctctgtaagaagaaggggggcacctgggggcctcgtacagacgctccagcggaatacaagagccg

    Selected Cols:                                                                                                                                                                 

    Gaps Scores:                                                                                                                                                                   
    Similarity Scores:                                                                                                                                                             

                                                                  250       260       270       280       290       300       310       320       330       340       350       360
                                                           =========+=========+=========+=========+=========+=========+=========+=========+=========+=========+=========+=========+
    GCA_000260075.2#JH651001.1_53017-53465                 ttccggctattacgtaagtcgatggcgctcaactacgtaattcatt-atgtgcctgtctattaatgcatc-tagtgtcttggtgctggttggtggggaaaggatccatgccctgctgaat
    GCA_000271745.2#JH717840.1_5103546-5103992             ttccggctattacgtaagtcgatgacgctttaccatgtagttcatt-atgtgcctg--tattgatgcatc-tagtgtcttggtgccggttggtggggagaggatccatgccctgctgaat
    GCA_000400815.2#CP009080.1_1179336-1179785             aagtggctactatgtatgtgtataacccttagtaacgtcacttattgatttgaccgctcgttaacacatc-cagtgtcttgggaatggctggtggggtgaggatccatgccctagggagt
    GCA_000585705.1#AEYB01001584.1_5717-6165               ttccggctattacgtaagtctatgacgctcaataacgtatttcata-ttatgtctgcgtattaatgcatc-tagtgtcttggtgatggttggtggggaaacgatccatgccctgctgaat
    GCA_001599515.1#BCHB01000008.1_128823-129266           ctccggctattacgtaagtcgacgatgctcaaaaacgtaacacatt-atgtgcctg--tattaatgaatc-tagtgccttggtgctggttggtggggagaagacccatgccctgctgaat
    GCA_001680525.1#MADZ01000944.1_3517-3965               ttccggctattacgtaagtctatgacgctcaataacgtatttcata-ttatgtctgcgtattaatgcatc-tagtgtcttggtgatggttggtggggaaacgatccatgccctgctgaat
    GCA_001680535.1#MAED01002951.1_1336-1785               ttccggctattacgtaagtctatgacgctcaataacgtatttcata-ttatgtctgcgtattaatgcatc-tagtgtcttggtgatggttggtggggaaacgatccatgccctgctgaat
    GCA_001680595.1#MAEE01000161.1_9576-10025              ttccggctattacgtaagtctatgacgctcaataacgtatttcata-ttatgtctgcgtattaatgcatc-tagtgtcttggtgatggttggtggggaaacgatccatgccctgctgaat
    GCA_001680605.1#MADY01000512.1_3407-3855               ttccggctattacgtaagtctatgacgctcaataacgtatttcata-ttatgtctgcgtattaatgcatc-tagtgtcttggtgatggttggtggggaaacgatccatgccctgctgaat
    GCA_001680665.1#MADX01001027.1_5081-5529               ttccggctattacgtaagtctatgacgctcaataacgtatttcata-ttatgtctgcgtattaatgcatc-tagtgtcttggtgatggttggtggggaaacgatccatgccctgctgaat
    GCA_001680685.1#MAEC01002105.1_1371-1819               ttccggctattacgtaagtctatgacgctcaataacgtatttcata-ttatgtctgcgtattaatgcatc-tagtgtcttggtgatggttggtggggaaacgatccatgccctgctgaat
    GCA_001680725.1#MAEF01000026.1_12451-12900             ttccggctattacgtaagtctatgacgctcaataacgtatttcata-ttatgtctgcgtattaatgcatc-tagtgtcttggtgatggttggtggggaaacgatccatgccctgctgaat
    GCA_001931975.2#MSJJ02000021.1_21498-21944             ttccggctattacgtaagtcgatgacgctttaccatgtagttcatt-atgtgcctg--tattaatgcatc-tagtgtcttggtgccggttggtggggagaggatccatgccctgctgaat
    GCA_002233775.1#NJCV01000665.1_343-790                 gagcggctattacgtaagtcgatgacgctcaacaacgtaattcatc-atgtgggtgtctattaatgcatc-tagtgtcttggtgctggttggtggggagtggatccatgccctgctgaat
    GCA_002233895.1#NJCL01000052.1_6292-6740               gagcggctattacgtaagtcgatgacgctcaacaacgcaattcatc-atgtgcctgtctattaatgcatc-tagtgtcttggtgctggttggtggggagaggatccatgccctgctgaat
    GCA_002233895.1#NJCL01000986.1_2608-3056               gagcggctattacgtaagtcgatgacgctcaacaacgtaattcatc-atgtgcctgtctattaattcatc-tagtgtcttggtgctggttggtggggagaggatccatgccctgctgaat
    GCA_002233895.1#NJCL01000862.1_714-1162                gagcggctattatgtaagtcgatgacgctcaacaacgtaattcatc-atgtgcctgtctattaatgcatc-tagtgtcttggtgatggttggtggggaattgatccatgccctgctgaat
    GCA_002233915.1#NJCM01002567.1_585-1030                gagcggctattacgtaagtcgatgacgctcaacaacgtaat---tc-atgtgcctgtctattaattcatc-tagtgtcttggtgctggttggtggggagaggatccatgccctgctgaat
    GCA_002233915.1#NJCM01000349.1_3242-3690               gagcggctattacgtaagtcgatgacgctcaacaacgtaattcatc-atgtgcctgtctattaattcatc-tagtgtcttggtgctggttggtggggagaggatccatgccctgctgaat
    GCA_002233935.2#NJBT02000074.1_114534-114981           gagcggctattacgtaagtcgatgatgctcaacaacgtaattcatc-atgtgggtgtctattaacgcatc-tagtgtcttggtgctggttggtggggagtggatccatgccctgctgaat
    GCA_002234115.1#NJCF01001371.1_90-531                  gagcggcgattacgtaagtcgatgacgctcaacaacgcaattcatc-atgtgcctgtctattaatgcata-tagtgtcttggtgctggttggtggggaaatgatccatgccctgctgaat
    GCA_002234115.1#NJCF01000302.1_3435-3883               gagcggctattatgtaagtcgatgacgctcaacaacgtaattcatc-atgtgcctgtctattaatgcatc-tagtgtcttggtgatggttggtggggaattgatccatgccctgctgaat
    GCA_002234115.1#NJCF01000961.1_5490-5938               gagcggctattacgtaagtcgatgacgctcaacaacgcaattcatc-atgtgcctgtctattaatgcatc-tagtgtcttggtgctggttggtggggagaggatccatgccctgctgaat
    GCA_002234195.1#NJCK01002649.1_264-712                 gagcggctattacgtaagtcgatgacgctcaacaacgtaattcatc-atgtgcctgtctattaattcatc-tagtgtcttggtgctggttggtggggagaggatccatgccctgctgaat
    GCA_002234205.1#NJCQ01003026.1_1778-2226               gagcggctattacgtaagtcgatgacgctcaacaacgcaattcatc-atgtgcctgtctattaatgcatc-tagtgtcttggtgctggttggtggggaactgatccatgccctgctgaat
    GCA_002234205.1#NJCQ01001471.1_358-806                 gagcggctattacgtaagtggatgacgctcaataacgcaattcatc-atgtgcctgtctattaatacatc-tagtgtcttggtgctggttggtggggagaagatccatgccctgctgaat
    GCA_002234235.1#NJCR01003189.1_7833-8281               gagcggctattacgtaagtcgatgacgctcaacaacgcaattcatc-atgtgcctgtctattaatgcatc-tagtgtcttggtgctggttggtggggaactgatccatgccctgctgaat
    GCA_002234235.1#NJCR01000523.1_1652-2100               gagcggctattacgtaagtggatgacgctcaataacgcaattcatc-atgtgcctgtctattaatacatc-tagtgtcttggtgctggttggtggggagaagatccatgccctgctgaat
    GCA_002234255.1#NJCS01000197.1_9074-9523               gagcggctattacgtaagtcgatgacgcttaataacgtaatttatc-atgtaggtgtctattaatgcatc-tagtgtcttggtgctggttggtggggagtggatctatgccctgctgaat
    GCA_002234255.1#NJCS01000257.1_23088-23528             gagcggctattacgtaagttaatgacgcttaataacgtaatttatc-atatgggtgtctattaatgcatc-tagtgtcttggtgctggttagtggggagtggatctatgccctgctgaat
    GCA_002234285.1#NJCT01000251.1_2006-2454               gagcggctattatgtaagtcgatgacgctcaacaacgtaattcatc-atgtgcctgtctattaatgcatc-tagtgtcttggtgatggttggtggggaattgatccatgccctgctgaat
    GCA_002234285.1#NJCT01000112.1_2108-2556               gagcggctattacgtaagtcgatgacgctcaacaacgcaattcatc-atgtgcctgtctattaatgcatc-tagtgtcttggtgctggttggtggggagaggatccatgccctgctgaat
    GCA_002776445.1#PDEZ01000231.1_7918-8368               aagtggctactatgtatgtgtataacccttagtaacgtcacttattgatttgaccgctcgttaacacatc-cagtgtcttgggaatggctggtggggtgaggatccatgccctagggagt
    GCA_002892985.1#MPSH01000001.1_2159685-2160134         aagtggctactatgtatgtgtataacccttagtaacgtcacttattgatttgaccgctcgttaacacatc-cagtgtcttgggaatggctggtggggtgaggatccatgccctagggagt
    GCA_002893025.1#PHNV01000722.1_4459-4908               aagtggctactatgtatgtgtataacccttagtaacgtcacttattgatttgaccgctcgttaacacatc-cagtgtcttgggaatggctggtggggtgaggatccatgccctagggagt
    GCA_002893035.1#PHNW01000920.1_4459-4908               aagtggctactatgtatgtgtataacccttagtaacgtcacttattgatttgaccgctcgttaacacatc-cagtgtcttgggaatggctggtggggtgaggatccatgccctagggagt
    GCA_002982035.1#PVPY01001450.1_5032-5474               ttccggctattacgtaagtggatgatgctcaacaacgtaattcatt-a-gtgcctg--tattaatgcatc-tagtgtcttggtgctggttggtggggagaggatccatgccctgccgaat
    GCA_002982055.1#PVPZ01001881.1_721-1163                ttccggctattacgtaagtggatgatgctcaacaacgtaattcatt-a-gtgcctg--tattaatgcatc-tagtgtcttggtgctggttggtggggagaggatccatgccctgccgaat
    GCA_003025205.1#PXUO01000119.1_69286-69732             ttccggctattacgtaagtcgatgacgctttaccatgtagttcatt-atgtgcctg--tattaatgcatc-tagtgtcttggtgccggttggtggggagaggatccatgccctgctgaat
    GCA_003025235.1#PXUN01000132.1_66066-66512             ttccggctattacgtaagtcgatgacgctttaccatgtagttcatt-atgtgcctg--tattaatgcatc-tagtgtcttggtgccggttggtggggagaggatccatgccctgctgaat
    GCA_004109745.1#RSDZ01000158.1_4397-4846               aagtggctactatgtatgtgtataacccttagtaacgtcacttattgatttgaccgctcgttaacacatc-cagtgtcttgggaatggctggtggggtgtggatccatgccctagggagt
    GCA_004141715.1#MQTW01000814.1_378-825                 gagcggctattacgtaagtcgatgacgctcaacaacgtaattcatc-atgtgggtgtctattaatgcatc-tagtgtcttggtgctggttggtggggagtggatccatgccctgctgaat
    GCA_004291455.1#QUWZ01000068.1_80333-80705             gagcggctattatgtaagtcgatgacgctcaaaaacgtaattcatc-atgtgcctgtctattaatgcatg-tagtgtcttggtgatggttggtggggaacggatccatgccctgctgaat
    GCA_004292535.1#QUXA01000044.1_77168-77540             gagcggctattatgtaagtcgatgacgctcaaaaacgtaattcatc-atgtgcctgtctattaatgcatg-tagtgtcttggtgatggttggtggggaacggatccatgccctgctgaat
    GCA_009297365.1#WGOL01000900.1_612-1060                gagcggctattacgtaagtcgatggcgctcagcaacgtaattcatc-atgtgcctgtctattaatgcatc-tagtgtcttggtgctggttggtggggaaacgatccatgccctgctgaat
    GCA_009297515.1#WGOI01001607.1_1477-1925               ttccggctattacgtaagtcgatgacgctcaacaacgtaattcatt-atgtacctgtctattaatgcacc-tagtgtcttggtgttggttggtggggagaggatccatgccctgctgaat
    GCA_009297675.1#WGOJ01003344.1_1223-1671               ttccggctattacgtaagtcgatgacgctcaacaacgtaattcatc-atgtgcctgtctattaatgcatg-tagtgtcttggtgctggttggtggggagttgatccatgccctgctgaat
    GCA_009298545.1#WGQK01000668.1_4136-4584               gagcggctattacgtaagtcgatgacgctcaacaacgcaattcatc-atgtgcctgtctattaatgcatc-tagtgtcttggtgctggttggtggggagaggatccatgtcctgctgaat
    GCA_009298855.1#WGQN01001540.1_1461-1906               ttccggctattacgtaagtc---gacgctcaacaacgtaattcatt-atgtgcctgtctattaatgcacc-tagtgtcttggtgctggttggtggggagaggacccatgccctgctgaat
    GCA_009298985.1#WGRN01000796.1_1047-1495               gagcggctattacgtaagtcgatgacgttcaacaacgtaattcatc-atgtgcctgtctattaatgcatc-tagtgtcttggtgctggttggtggggagaagatccatgtcctgctgaat
    GCA_009299045.1#WGRK01001155.1_826-1274                gagcggatattatgtaagtcgatgacgctcaacaacgcaattcatc-atgtgcctgtctattaatgcatc-tagtgtcttggtgctggttggtggggaactgatccatgccctgctgaat
    GCA_009299075.1#WGRJ01000863.1_12409-12855             ttccggctattacgtaagtcgatgacgctttaccatgtagttcatt-atgtgcctg--tattaatgcatc-tagtgtcttggtgccggttggtggggagaggatccatgccctgctgaat
    GCA_009299115.1#WGRH01001009.1_1459-1907               gagcggctattacgtaagtcgatgacgctcaacaacgcaattcatc-atgtgcctgtctattaatgcatc-tagtgtcttggtgctggttggtggggagaggatccatgtcctgctgaat
    GCA_009299215.1#WGRS01000248.1_42092-42540             ttccggctattacgtaagtcgatggcgctcgactacgtaattcatt-atgtgcctgtctattaatgcgtc-tagtgtcttggtgctggttggtggggagaggatccatgccctgctgaat
    GCA_009299215.1#WGRS01000488.1_939-1388                ttccggctattacgtaagtcgatgacgctcaacaacgtaattcatt-atgtgcctgtctattaatgcacc-tagtgtcttggtgctggttggtggggagaggatccatgccctgctgaat
    GCA_009299215.1#WGRS01000332.1_7989-8437               ttccggctattacgtaagtcgatgacgctcaacaacgtaattcatc-atgtgcctgtctattaatgcatg-tagtgtcttggtgctggttggtggggagttgatccatgccctgctgaat
    GCA_009663885.1#WJDV01000002.1_418952-419401           aagtggctactatgtatgtgtataacccttagtaacgtcacttattgatttgaccgctcgttaacacatc-cagtgtcttgggaatggctggtggggtgaggatccatgccctagggagt
    GCA_009746015.1#VLJC01000049.1_70684-71130             ttccggctattacgtaagtcgatgacgctttaccatgtagttcatt-atgtgcctg--tattaatgcatc-tagtgtcttggtgccggttggtggggagaggatccatgccctgctgaat
    GCA_011032855.1#WESG01000339.1_10823-11271             gagcggctattacgtaagtcgatgacgctctacaacgtaattcatc-atgtgcctgtctattaatgcatc-tagtgtcttggtgccggttggtggggaagggatccatgccctgctgaat
    GCA_011032945.1#WNXM01001784.1_7328-7777               aagtggctactatgtatgtgtataacccttagtaacgtcacttattgatttgaccgctcgttaacacatc-cagtgtcttgggaatggctggtggggtgaggatccatgccctagggagt
    GCA_011032955.1#WNXL01001437.1_4250-4699               aagtggctactatgtatgtgtataacccttagtaacgtcacttattgatttgaccgctcgttaacacatc-cagtgtcttgggaatggctggtggggtgaggatccatgccctagggagt
    GCA_011032965.1#WNXK01000961.1_4382-4831               aagtggctactatgtatgtgtataacccttagtaacgtcacttattgatttgaccgctcgttaacacatc-cagtgtcttgggaatggctggtggggtgaggatccatgccctagggagt
    GCA_011033455.1#WESL01002533.1_4420-4868               gagcggatattatgtaagtcgatgacgctcaacaacgcaattcatc-atgtgcctgtctattaatgcatc-tagtgtcttggtgctggttggtggggaactgatccatgccctgctgaat
    GCA_011033455.1#WESL01001216.1_8955-9403               gagcggctattacgtaagtcgatgacgctcaacaacgcaattcatc-atgtgcctgtctattaatgcatc-tagtgtcttggtgctggttggtggggaaatgatccatgccctgctgaat
    GCA_011033575.1#WESJ01009652.1_394-842                 gagcggctattacgtaagtcgatgacgctcaacaacgcaattcatc-atgtgcctgtctattaatgcatc-tagtgtcttggtgctggttggtggggaaatgatccatgccctgctgaat
    GCA_011033575.1#WESJ01007920.1_731-1149                gagcggatattatgtaagtcgatgacgctcaacaacgcaattcatc-atgtgcctgtctattaatgcatc-tagtgtcttggtgctggttggtggggaactgatccatgccctgctgaat
    GCA_011033685.1#WESM01008445.1_1386-1834               gagcggatattatgtaagtcgatgacgctcaacaacgcaattcatc-atgtgcctgtctattaatgcatc-tagtgtcttggtgctggttggtggggaaatgatccatgccctgctgaat
    GCA_011033745.1#WETB01002153.1_7139-7587               gagcggatattatgtaagtcgatgacgctcaacaacgcaattcatc-atgtgcctgtctattaatgcatc-tagtgtcttggtgctggttggtggggaactgatccatgccctgctgaat
    GCA_011033805.1#WESY01002113.1_415-863                 gagcggatattatgtaagtcgatgacgctcaacaacgcaattcatc-atgtgcctgtctattaatgcatc-tagtgtcttggtgctggttggtggggaactgatccatgccctgctgaat
    GCA_011033815.1#WESW01000773.1_2707-3155               gagcggatattatgtaagtcgatgacgctcaacaacgcaattcatc-atgtgcctgtctattaatgcatc-tagtgtcttggtgctggttggtggggaactgatccatgccctgctgaat
    GCA_011033815.1#WESW01000649.1_851-1299                gagcggctattacgtaagtcgatgacgctcaacaacgcaattcatc-atgtgcctgtctattaatgcatc-tagtgtcttggtgctggttggtggggaaatgatccatgccctgctgaat
    GCA_011033895.1#WETL01000467.1_850-1298                gagcggctattacgtaagtcgatgacgctcaacaacgcaattcatc-atgtgcctgtctattaatgcatc-tagtgtcttggtgctggttggtggggaaatgatccatgccctgctgaat
    GCA_011033895.1#WETL01000641.1_3860-4278               gagcggatattatgtaagtcgatgacgctcaacaacgcaattcatc-atgtgcctgtctattaatgcatc-tagtgtcttggtgctggttggtggggaactgatccatgccctgctgaat
    GCA_011034195.1#WETT01000020.1_547-995                 gagcggatattatgtaagtcgatgacgctcaacaacgcaattcatc-atgtgcctgtctattaatgcatc-tagtgtcttggtgctggttggtggggaactgatccatgccctgctgaat
    GCA_011034205.1#WETS01005722.1_2442-2890               gagcggatattatgtaagtcgatgacgctcaacaacgcaattcatc-atgtgcctgtctattaatgcatc-tagtgtcttggtgctggttggtggggaactgatccatgccctgctgaat
    GCA_011034415.1#WEUC01001584.1_7719-8167               gagcggatattatgtaagtcgatgacgctcaacaacgcaattcatc-atgtgcctgtctattaatgcatc-tagtgtcttggtgctggttggtggggaactgatccatgccctgctgaat
    GCA_011034445.1#WEUB01001158.1_3523-3971               gagcggatattatgtaagtcgatgacgctcaacaacgcaattcatc-atgtgcctgtctattaatgcatc-tagtgtcttggtgctggttggtggggaaatgatccatgccctgctgaat
    GCA_011034445.1#WEUB01000999.1_8836-9284               gagcggctattacgtaagtcgatgacgctcaacaacgcaattcatc-atgtgcctgtctattaatgcatc-tagtgtcttggtgctggttggtggggaaatgatccatgccctgctgaat
    GCA_011034575.1#WEUE01008972.1_493-941                 gagcggctattacgtaagtcgatgacgctcaacaacgcaattcatc-atgtgcctgtctattaatgcatc-tagtgtcttggtgctggttggtggggaaatgatccatgccctgctgaat
    GCA_011034625.1#WEUR01000714.1_7159-7607               gagcggatattatgtaagtcgatgacgctcaacaacgcaattcatc-atgtgcctgtctattaatgcatc-tagtgtcttggtgctggttggtggggaactgatccatgccctgctgaat
    GCA_011034645.1#WEUV01000741.1_2997-3445               gagcggctattacgtaagtcgatgacgctcgacaaagtaatttatc-atgtgcctttctattaatacatc-tagtgtcttggtactggttggtggggaaatgatccatgccctgctgaat
    GCA_011034655.1#WEUQ01004890.1_3105-3553               gagcggatattatgtaagtcgatgacgctcaacaacgcaattcatc-atgtgcctgtctattaatgcatc-tagtgtcttggtgctggttggtggggaactgatccatgccctgctgaat
    GCA_011034655.1#WEUQ01000935.1_566-1014                gagcggctattacgtaagtcgatgacgctctacaacgtaattcatc-atgtgcctgtctattaatgcatc-tagtgtcttggtgccggttggtggggaagggatccatgccctgctgaat
    GCA_011034775.1#WEVI01000501.1_5488-5930               gagcggctattacgtaagtcgatgacgctcaacaacgcaattcatc-atgtgcctgtctattaatgcatc-tagtgtcttggtgctggttggtggggaaatgatccatgccctgctgaat
    GCA_011034775.1#WEVI01000783.1_2046-2494               gagcggatattatgtaagtcgatgacgctcaacaacgcaattcatc-atgtgcctgtctattaatgcatc-tagtgtcttggtgctggttggtggggaactgatccatgccctgctgaat
    GCA_011034815.1#WEVE01000912.1_9755-10173              gagcggctattacgtaagtcgatgacgctcaacaacgcaattcatc-atgtgcctgtctattaatgcatc-tagtgtcttggtgctggttggtggggaaatgatccatgccctgctgaat
    GCA_011034815.1#WEVE01002086.1_1620-2038               gagcggatattatgtaagtcgatgacgctcaacaacgcaattcatc-atgtgcctgtctattaatgcatc-tagtgtcttggtgctggttggtggggaactgatccatgccctgctgaat
    GCA_011034845.1#WEVA01001230.1_1747-2195               gagcggatattatgtaagtcgatgacgctcaacaacgcaattcatc-atgtgcctgtctattaatgcatc-tagtgtcttggtgctggttggtggggaactgatccatgccctgctgaat
    GCA_011035075.1#WEVS01001164.1_694-1142                gagcggatattatgtaagtcgatgacgctcaacaacgcaattcatc-atgtgcctgtctattaatgcatc-tagtgtcttggtgctggttggtggggaactgatccatgccctgctgaat
    GCA_011035185.1#WEVO01012916.1_426-874                 gagcggatattatgtaagtcgatgacgctcaacaacgcaattcatc-atgtgcctgtctattaatgcatc-tagtgtcttggtgctggttggtggggaactgatccatgccctgctgaat
    GCA_011035185.1#WEVO01003306.1_4368-4816               gagcggctattacgtaagtcgatgacgctcaacaacgcaattcatc-atgtgcctgtctattaatgcatc-tagtgtcttggtgctggttggtggggaaatgatccatgccctgctgaat
    GCA_011035205.1#WEVG01003463.1_75-523                  gagcggatattatgtaagtcgatgacgctcaacaacgcaattcatc-atgtgcctgtctattaatgcatc-tagtgtcttggtgctggttggtggggaactgatccatgccctgctgaat
    GCA_011035235.1#WEWM01001159.1_2667-3085               gagcggatattatgtaagtcgatgacgctcaacaacgcaattcatc-atgtgcctgtctattaatgcatc-tagtgtcttggtgctggttggtggggaactgatccatgccctgctgaat
    GCA_011035235.1#WEWM01001540.1_2265-2683               gagcggctattacgtaagtcgatgacgctcaacaacgcaattcatc-atgtgcctgtctattaatgcatc-tagtgtcttggtgctggttggtggggaaatgatccatgccctgctgaat
    GCA_011035245.1#WEWK01000657.1_7022-7440               gagcggatattatgtaagtcgatgacgctcaacaacgcaattcatc-atgtgcctgtctattaatgcatc-tagtgtcttggtgctggttggtggggaactgatccatgccctgctgaat
    GCA_011035245.1#WEWK01000404.1_16648-17029             gagcgggtattacgtaagtcgatgacgctcaacaacgcaattcatc-atgtgcctgtctgttaatgcatc-tagtgtcttggtgctggttggtggggaaatgatccatgccctgctgaat
    GCA_011035255.1#WEWN01000527.1_2412-2830               gagcggatattatgtaagtcgatgacgctcaacaacgcaattcatc-atgtgcctgtctattaatgcatc-tagtgtcttggtgctggttggtggggaactgatccatgccctgctgaat
    GCA_011035255.1#WEWN01000751.1_2320-2738               gagcggctattacgtaagtcgatgacgctcaacaacgcaattcatc-atgtgcctgtctattaatgcatc-tagtgtcttggtgctggttggtggggaaatgatccatgccctgctgaat
    GCA_011035265.1#WEWJ01000412.1_17167-17548             gagcgggtattacgtaagtcgatgacgctcaacaacgcaattcatc-atgtgcctgtctgttaatgcatc-tagtgtcttggtgctggttggtggggaaatgatccatgccctgctgaat
    GCA_011035265.1#WEWJ01000569.1_529-977                 gagcggatattatgtaagtcgatgacgctcaacaacgcaattcatc-atgtgcctgtctattaatgcatc-tagtgtcttggtgctggttggtggggaactgatccatgccctgctgaat
    GCA_011035345.1#WEWE01000725.1_7055-7503               gagcggatattatgtaagtcgatgacgctcaacaacgcaattcatc-atgtgcctgtctattaatgcatc-tagtgtcttggtgctggttggtggggaactgatccatgccctgctgaat
    GCA_011035345.1#WEWE01000822.1_176-557                 gagcgggtattacgtaagtcgatgacgctcaacaacgcaattcatc-atgtgcctgtctgttaatgcatc-tagtgtcttggtgctggttggtggggaaatgatccatgccctgctgaat
    GCA_011035375.1#WEWB01000636.1_176-557                 gagcgggtattacgtaagtcgatgacgctcaacaacgcaattcatc-atgtgcctgtctgttaatgcatc-tagtgtcttggtgctggttggtggggaaatgatccatgccctgctgaat
    GCA_011035375.1#WEWB01000586.1_665-1113                gagcggatattatgtaagtcgatgacgctcaacaacgcaattcatc-atgtgcctgtctattaatgcatc-tagtgtcttggtgctggttggtggggaactgatccatgccctgctgaat
    GCA_011035435.1#WEWD01000787.1_1777-2225               gagcggatattatgtaagtcgatgacgctcaacaacgcaattcatc-atgtgcctgtctattaatgcatc-tagtgtcttggtgctggttggtggggaactgatccatgccctgctgaat
    GCA_011035455.1#WEVZ01000295.1_5889-6270               gagcgggtattacgtaagtcgatgacgctcaacaacgcaattcatc-atgtgcctgtctgttaatgcatc-tagtgtcttggtgctggttggtggggaaatgatccatgccctgctgaat
    GCA_011035485.1#WEVY01000687.1_5745-6126               gagcgggtattacgtaagtcgatgacgctcaacaacgcaattcatc-atgtgcctgtctgttaatgcatc-tagtgtcttggtgctggttggtggggaaatgatccatgccctgctgaat
    GCA_011035485.1#WEVY01000540.1_528-976                 gagcggatattatgtaagtcgatgacgctcaacaacgcaattcatc-atgtgcctgtctattaatgcatc-tagtgtcttggtgctggttggtggggaactgatccatgccctgctgaat
    GCA_011035495.1#WEVW01000533.1_1949-2397               gagcggatattatgtaagtcgatgacgctcaacaacgcaattcatc-atgtgcctgtctattaatgcatc-tagtgtcttggtgctggttggtggggaactgatccatgccctgctgaat
    GCA_011035625.1#WEWS01001096.1_809-1227                gagcggctattacgtaagtcgatgacgctcaacaacgcaattcatc-atgtgcctgtctattaatgcatc-tagtgtcttggtgctggttggtggggaaatgatccatgccctgctgaat
    GCA_011035625.1#WEWS01001310.1_2416-2834               gagcggatattatgtaagtcgatgacgctcaacaacgcaattcatc-atgtgcctgtctattaatgcatc-tagtgtcttggtgctggttggtggggaactgatccatgccctgctgaat
    GCA_011035755.1#WEXC01000589.1_22209-22657             gagcggctattacgtaagtcgatgacgctctacaacgtaattcatc-atgtgcctgtctattaatgcatc-tagtgtcttggtgccggttggtggggaagggatccatgccctgctgaat
    GCA_011035835.1#WEXF01000545.1_8821-9239               gagcggctattacgtaagtcgatgacgctcaacaacgcaattcatc-atgtgcctgtctattaatgcatc-tagtgtcttggtgctggttggtggggaaatgatccatgccctgctgaat
    GCA_011035835.1#WEXF01000653.1_3838-4256               gagcggatattatgtaagtcgatgacgctcaacaacgcaattcatc-atgtgcctgtctattaatgcatc-tagtgtcttggtgctggttggtggggaactgatccatgccctgctgaat
    GCA_011035975.1#WEXJ01007704.1_1039-1487               gagcggctattatgtaagtcgatgacgctcaacaacgcaattcatc-atgtgcctgtctattaatgcatc-tagtgtcttggtgctggttggtggggaaatgatccatgccctgctgaat
    GCA_011035995.1#WEXI01000903.1_7456-7904               gagcggatattatgtaagtcgatgacgctcaacaacgcaattcatc-atgtgcctgtctattaatgcatc-tagtgtcttggtgctggttggtggggaactgatccatgccctgctgaat
    GCA_011035995.1#WEXI01001093.1_807-1255                gagcggctattacgtaagtcgatgacgctctacaacgtaattcatc-atgtgcctgtctattaatgcatc-tagtgtcttggtgccggttggtggggaagggatccatgccctgctgaat
    GCA_011036045.1#WEXT01000869.1_163-611                 gagcggctattacgtaagtcgatgacgctcaacaacataattcatc-atgtgcctgtctattaatgcatc-tagtgtcttggtgctggttggtggggagaagatccatgccctgctgaat
    GCA_011036135.1#WEXQ01000241.1_21717-22165             gagcggctattacgtaagtcgatgacgctctacaacgtaattcatc-atgtgcctgtctattaatgcatc-tagtgtcttggtgccggttggtggggaagggatccatgccctgctgaat
    GCA_011036135.1#WEXQ01000570.1_528-976                 gagcggatattatgtaagtcgatgacgctcaacaacgcaattcatc-atgtgcctgtctattaatgcatc-tagtgtcttggtgctggttggtggggaactgatccatgccctgctgaat
    GCA_011036215.1#WEYG01000053.1_6223-6671               gagcggctattacgtaagtcgatgacgctcaacaacataattcatc-atgtgcctgtctattaatgcatc-tagtgtcttggtgctggttggtggggagaagatccatgccctgctgaat
    GCA_011036305.1#WEXY01003662.1_3416-3864               gagcggatattatgtaagtcgatgacgctcaacaacgcaattcatc-atgtgcctgtctattaatgcatc-tagtgtcttggtgctggttggtggggaactgatccatgccctgctgaat
    GCA_011036305.1#WEXY01002318.1_5546-5994               gagcggctattacgtaagtcgatgacgctcaacaacgcaattcatc-atgtgcctgtctattaatgcatc-tagtgtcttggtgctggttggtggggaaatgatccatgccctgctgaat
    GCA_011036365.1#WEYQ01001970.1_647-1095                gagcggatattatgtaagtcgatgacgctcaacaacgcaattcatc-atgtgcctgtctattaatgcatc-tagtgtcttggtgctggttggtggggaactgatccatgccctgctgaat
    GCA_011036595.1#WEYT01000407.1_15167-15585             gagcggatattatgtaagtcgatgacgctcaacaacgcaattcatc-atgtgcctgtctattaatgcatc-tagtgtcttggtgctggttggtggggaactgatccatgccctgctgaat
    GCA_011036595.1#WEYT01000705.1_8787-9235               gagcggctattacgtaagtcgatgacgctcaacaacgcaattcatc-atgtgcctgtctattaatgcatc-tagtgtcttggtgctggttggtggggaaatgatccatgccctgctgaat
    GCA_011036765.1#WEUS01002153.1_588-1036                gagcggatattatgtaagtcgatgacgctcaacaacgcaattcatc-atgtgcctgtctattaatgcatc-tagtgtcttggtgctggttggtggggaactgatccatgccctgctgaat
    GCA_011036795.1#WEUZ01000271.1_2987-3435               gagcggctattacgtaagtcgatgacgctcgacaaagtaatttatc-atgtgcctttctattaatacatc-tagtgtcttggtactggttggtggggaaatgatccatgccctgctgaat
    GCA_011036875.1#WEWI01000026.1_11685-12103             gagcggctattacgtaagtcgatgacgctcaacaacgcaattcatc-atgtgcctgtctattaatgcatc-tagtgtcttggtgctggttggtggggaaatgatccatgccctgctgaat
    GCA_011036875.1#WEWI01000910.1_3838-4256               gagcggatattatgtaagtcgatgacgctcaacaacgcaattcatc-atgtgcctgtctattaatgcatc-tagtgtcttggtgctggttggtggggaactgatccatgccctgctgaat
    GCA_011037075.1#WEZD01015082.1_365-770                 gagcggatattatgtaagtcgatgacgctcaacaacgcaattcatc-atgtgcctgtctattaatgcatc-tagtgtcttggtgctggttggtggggaactgatccatgccctgctgaat
    GCA_011421275.1#WIKV01000306.1_26907-27325             gagcggctattacgtaagtcgatgacgctcaacaacgcaattcatc-atgtgcctgtctattaatgcatc-tagtgtcttggtgctggttggtggggaaatgatccatgccctgctgaat
    GCA_011421275.1#WIKV01000622.1_3838-4256               gagcggatattatgtaagtcgatgacgctcaacaacgcaattcatc-atgtgcctgtctattaatgcatc-tagtgtcttggtgctggttggtggggaactgatccatgccctgctgaat
    GCA_011421285.1#WIKU01001812.1_697-1145                gagcggatattatgtaagtcgatgacgctcaacaacgcaattcatc-atgtgcctgtctattaatgcatc-tagtgtcttggtgctggttggtggggaactgatccatgccctgctgaat
    GCA_011421305.1#WIKW01002869.1_809-1227                gagcggctattacgtaagtcgatgacgctcaacaacgcaattcatc-atgtgcctgtctattaatgcatc-tagtgtcttggtgctggttggtggggaaatgatccatgccctgctgaat
    GCA_011421305.1#WIKW01002126.1_1789-2207               gagcggatattatgtaagtcgatgacgctcaacaacgcaattcatc-atgtgcctgtctattaatgcatc-tagtgtcttggtgctggttggtggggaactgatccatgccctgctgaat
    GCA_011421375.1#WILC01001342.1_696-1144                gagcggatattatgtaagtcgatgacgctcaacaacgcaattcatc-atgtgcctgtctattaatgcatc-tagtgtcttggtgctggttggtggggaactgatccatgccctgctgaat
    GCA_011426335.1#WILD01001610.1_1018-1466               gagcggatattacgtaagtcgatgacgctcaacaacgcaattcatc-atgtgcctgtctattaatgcatc-tagtgtcttggtgctggttggtggggaaatgatccatgccctgctgaat
    GCA_011426355.1#WILF01007384.1_372-820                 gagcggatattatgtaagtcgatgacgctcaacaacgcaattcatc-atgtgcctgtctattaatgcatc-tagtgtcttggtgctggttggtggggaactgatccatgccctgctgaat
    GCA_011426355.1#WILF01000505.1_6553-7001               gagcggatattatgtaagtcgatgacgctcaacaacgcaattcatc-atgtgcctgtctattaatgcatc-tagtgtcttggtgctggttggtggggaaatgatccatgccctgctgaat
    GCA_013170945.1#MU047874.1_170025-170474               aagtggctactatgtatgtgtataacccttagtaacgtcacttattgatttgaccgctcgttaacacatc-cagtgtcttgggaatggctggtggggtgtggatccatgccctagggagt
    GCA_013184365.1#JABCJX010000598.1_3113-3561            ttccggctattacgtaagtcgatgacgctcaacaacgtaattcatc-atgtgcctgtctattaatgcatc-tagtgtcttggtgctggttggtggggaacggatccatgccctgctgaat
    GCA_013266185.1#JABEXW010001135.1_3715-4163            gagcggctattacgtgagtcgatgatgttcaacaacgcaattcatc-atgtgcctgtgtattaatgcttc-tagtgtcttggtgctggttggtggggaaatgatccatgccctgctgaat
    GCA_013347355.2#JAALGN020000013.1_426461-426909        ttcaggctattacgtaagtcaatggcgctcaacaacgtaattcatt-atgtgcctgtctattaatgcatc-tagtgtcttggtgctggttggtggggagaggatccatgccctgctgaat
    GCA_013363175.1#JABEEP010001877.1_2080-2528            ttccggctattacgtaagtctatgacgctcaataacgtatttcata-ttatgtctgcgtattaatgcatc-tagtgtcttggtgatggttggtggggaaacgatccatgccctgctgaat
    GCA_013396025.1#JAAOAQ010001214.1_4450-4898            gagcggctattatgtaagtcgatgacgctcaacaacgtacttcatt-atgtgcctgtctattaatgcatc-tagtgtcttggttctggttggtggggaacggatccatgccctgctgaat
    GCA_013396205.1#JAAOAN010000160.1_45920-46352          ttccggctactacgtaagtcggtgacactcaagaa-------catt-atgtgcttgtctattaatgcgta-tagtgtcttggtgatggttggtggggagaagacccgtgccctgctgaat
    GCA_013618355.1#JABFES010000455.1_7017-7460            ctccggctattacgtaagtcgacgatgctcaaaaacgtaatacatt-atgtgccta--tattaatgaatc-tagtgccttggtggtggttggtggggagaagacccatgccctgctgaat
    GCA_013623525.1#JABGLY010000028.1_42787-43233          tgacggctactatgtaagttgatgatgctcaaaaacttacaccatc-atgtgtctgtctactaatgcatc-cagtgtctcggtaatggttggtggggtcatgatccatgccctgctgagt
    GCA_013623715.1#JABFFJ010000182.1_85034-85470          gagcggctattacgtaagtccatgacgtttaacaacggaattc----atgtgcttgtgtattaatgcgtc-tagtgtcttggtggtggttggtggggaaatgatccatgccctgttgaat
    GCA_014324575.1#WXUM01000474.1_1010-1458               ttccggctattacgtaagtcgatgacgctcaacaacgtaattcatt-atgtgcctgtctattaatgcacc-tagtgtcttggtgctggttggtggggagaggatccatgccctgctgaat
    GCA_014324595.1#WXUN01001159.1_797-1245                ttccggctattacgtaagtcgatgacgctcaacaacgtaattcatt-atgtgcctgtctattaatgcacc-tagtgtcttggtgctggttggtggggagaggatccatgccctgctgaat
    GCA_014324665.1#WXUR01000643.1_5483-5930               ttccggctattacgtaagtcgatggcgctcaacaacgtaattcatt-atgtgcctgtctattaatgcacc-tagtgtcttggtgctggttggtggggagaggatccatgccctgctgaat
    GCA_014324745.1#JAAMUY010001820.1_1146-1594            ttccggctattacgtaagtcgatgacgctcaacaacgtaattcatt-atgtgcctgtctattaatgcacc-tagtgtcttggtgctggttggtggggagaggatccatgccctgctgaat
    GCA_014324775.1#WXUT01000779.1_3905-4353               ttcaggctattacgtaagtcaatggcgctcaacaacgtaattcatt-atgtgcctgtctattaatgcatc-tagtgtcttggtgctggttggtggggagaggatccatgccctgctgaat
    GCA_014324795.1#WXUU01000551.1_4342-4789               ttccggctattacgtaagtcgatggcgctcaacaacgtaattcatt-atgtgcctgtctattaatgcacc-tagtgtcttggtgctggttggtggggagaggatccatgccctgctgaat
    GCA_014324835.1#WXUX01001216.1_2785-3233               ttcaggctattacgtaagtcaatggcgctcaacaacgtaattcatt-atgtgcctgtctattaatgcatc-tagtgtcttggtgctggttggtggggagaggatccatgccctgctgaat
    GCA_014324865.1#WXUY01001109.1_5504-5951               ttccggctattacgtaagtcgatggcgctcaacaacgtaattcatt-atgtgcctgtctattaatgcacc-tagtgtcttggtgctggttggtggggagaggatccatgccctgctgaat
    GCA_014324895.1#WXUZ01000915.1_1546-1994               ttccggctattacgtaagtcgatggcgctcaactacgtaattcatt-atgtgcctgtctattaatgcatc-tagtgtcttggtgctggttggtggggagaggatccatgccctgctgaat
    GCA_014325045.1#WXVI01000570.1_8895-9343               ttccggctattacgtaagtcgatgacgctcaacaacgtaattcatt-atgtacctgtctattaatgcacc-tagtgtcttggtgttggttggtggggagaggatccatgccctgctgaat
    GCA_014325205.1#JAAMVE010001426.1_3625-4073            ttccggctattacgtaagtcgatgacgctcaacaacgtaattcatt-atgtgcctgtctattaatgcacc-tagtgtcttggtgctggttggtggggagaggatccatgccctgctgaat
    GCA_014325315.1#WXVP01000164.1_88523-88895             gagcggctattatgtaagtcgatgacgctcaaaaacgtaattcatc-atgtgcctgtctattaatgcatg-tagtgtcttggtgatggttggtggggaacggatccatgccctgctgaat
    GCA_014843555.1#JAAOOQ010000005.1_239540-239989        gagcggctattacgtaagtcgctgacgctcaacaacgcaattcatc-atgtgcctgtctattaatgcatcttagtgtcttggtgctggttggtggggaactgatccatgccctgctgaat
    GCA_014843565.1#JAAOOP010000035.1_171592-172041        gagcggctattacgtaagtcgctgacgctcaacaacgcaattcatc-atgtgcctgtctattaatgcatcttagtgtcttggtgctggttggtggggaactgatccatgccctgctgaat
    GCA_014884825.1#JACTAS010000222.1_17723-18171          gagcggctattacgtaagtcgatgacgctcaacaacgtaattcatc-atgtgcctgtctattaatgcatc-tagtgtcttggtgctggttggtggggaactgatccatgccctgctgaat
    GCA_018440645.1#JACOQZ010000013.1_178443-178892        aagtggctactatgtatgtgtataacccttagtaacgtcacttattgatttgaccgctcgttaacacatc-cagtgtcttgggaatggctggtggggtgtggatccatgccctagggagt
    GCA_018440655.1#JACORB010000012.1_54438-54887          aagtggctactatgtatgtgtataacccttagtaacgtcacttattgatttgaccgctcgttaacacatc-cagtgtcttgggaatggctggtggggtgtggatccatgccctagggagt
    GCA_019055395.1#JAHKNW010006905.1_873-1321             ttccggctattacgtaagtcgatgacgctcaacaacgtaattcatt-atgtgcctgtctattaatgcacc-tagtgtcttggtgctggttggtggggagaggatccatgccctgctgaat
    GCA_019157295.1#JAELUQ010000008.1_3397787-3398230      ctccggctattacgtaagtcgacgatgctcaaaaacgtaatacatt-atgtgccta--tattaatgaatc-tagtgccttggtggtggttggtggggagaagacccatgccctgctgaat
    GCA_019191165.1#JAHARQ010000139.1_67224-67670          ttccggctattacgtaagtcgatgacgctttaccatgtagttcatt-atgtgcctg--tattaatgcatc-tagtgtcttggtgccggttggtggggagaggatccatgccctgctgaat
    GCA_019191175.1#JAHARR010000129.1_15625-16071          ttccggctattacgtaagtcgatgacgctttaccatgtagttcatt-atgtgcctg--tattaatgcatc-tagtgtcttggtgccggttggtggggagaggatccatgccctgctgaat
    GCA_019843925.1#JACDIY010000477.1_28970-29342          gagcggctattatgtaagtcgatgacgctcaaaaacgtaattcatc-atgtgcctgtctattaatgcatg-tagtgtcttggtgatggttggtggggaacggatccatgccctgctgaat
    GCA_020726535.1#JAHEWJ010000011.1_1000856-1001305      aagtggctactatgtatgtgtataacccttagtaacgtcacttattgatttgaccgctcgttaacacatc-cagtgtcttgggaatggctggtggggtgaggatccatgccctagggagt
    GCA_020744475.1#JAGMUX010000018.1_315043-315491        ttccggctattacgtaagtcgatggcgctcaactacgtaattcatt-atgtgcctgtctattaatgcatc-tagtgtcttggtgctggttggtggggagaggatccatgccctgctgaat
    GCA_020883595.1#JAJGQA010000014.1_323964-324412        ttccggctattacgtaagtctatgacgctcaataacgtatttcata-ttatgtctgcgtattaatgcatc-tagtgtcttggtgatggttggtggggaaacgatccatgccctgctgaat
    GCA_020883605.1#JAJHJC010000014.1_396196-396644        ttccggctattacgtaagtctatgacgctcaataacgtatttcata-ttatgtctgcgtattaatgcatc-tagtgtcttggtgatggttggtggggaaacgatccatgccctgctgaat
    GCA_020883615.1#JAJHJD010000017.1_393187-393635        ttccggctattacgtaagtctatgacgctcaataacgtatttcata-ttatgtctgcgtattaatgcatc-tagtgtcttggtgatggttggtggggaaacgatccatgccctgctgaat
    GCA_020883625.1#JAJGQB010000012.1_381552-382000        ttccggctattacgtaagtctatgacgctcaataacgtatttcata-ttatgtctgcgtattaatgcatc-tagtgtcttggtgatggttggtggggaaacgatccatgccctgctgaat
    GCA_021655895.1#JAJJWO010000410.1_1878-2303            gagcggctattatgtaagttgatgacgtttaataatgcaatcc------gcgtccttgtgctaatgcata-gagtgtcttggtgatggttggtggggaaacgatctatgccctgctgaat
    GCA_021730365.1#JAJOOV010000405.1_1213-1662            ttccggctattacgtaagtctatgacgctcaataacgtatttcata-ttatgtctgcgtattaatgcatc-tagtgtcttggtgatggttggtggggaaacgatccatgccctgctgaat
    GCA_023065405.1#JAJTCY010000003.1_74358-74801          ctccggctattacgtaagtcgacgatgctcaaaaacgtaatacatt-atgtgccta--tattaatgaatc-tagtgccttggtggtggttggtggggagaagacccatgccctgctgaat
    GCA_023628715.1#JAMAKD010000122.1_67003-67449          ttccggctattacgtaagtcgatgacgctttaccatgtagttcatt-atgtgcctg--tattaatgcatc-tagtgtcttggtgccggttggtggggagaggatccatgccctgctgaat
    GCA_024865645.1#JANKHN010000668.1_8435-8878            ctccggctattacgtaagtcgacgatgctcaaaaacgtaacacatt-atgtgcctg--tattaatgaatc-tagtgccttggtgctggttggtggggagaagacccatgccctgctgaat
    GCA_025201995.1#JAMSCT010000483.1_2189-2637            gagcggctattacgtaagtcgatgtcgctcaacaacgcaattcatc-atgtgcctgtctattaatgcatc-tagtgtcttggtgctggttggtggggaaatgatccatgccctgctgaat
    GCA_025203115.1#JAMSFS010000057.1_218973-219344        gagcggctattatgtaagtcgatgacgctcaaaaacgtaattcatc-atgtgcctgtctattaatgcatg-tagtgtcttggtgatggttggtggggaacggatccatgccctgctgaat
    GCA_025203755.1#JAMSFW010000517.1_9896-10342           gagcggcgattacgtaagtcgatgacgctcaacaacgtaattcatc-atgtgcctgtctattaatgtatc-tagtgtcttggtgctggttggtggggaactgatccatgccctgctgaat
    GCA_025203755.1#JAMSFW010000664.1_112-560              gagcggcgattacgtaagtcgatgacgctcaacaacgcaattcatc-atgtgcctgtctattaatgcata-tagtgtcttggtgctggttggtggggaaatgatccatgccctgctgaat
    GCA_025215475.1#JAMSDB010000983.1_1119-1567            ttccggctattacgtaagtcgatgacgctcaacaacgtaattcatt-atgtacctgtctattaatgcacc-tagtgtcttggtgttggttggtggggagaggatccatgccctgctgaat
    GCA_025215845.1#JAMSCV010001163.1_880-1265             ttccggcgattacgtaagtcgatgacgctcaacaacgtaattcatc-atgtgcctgtctattaatgcgtc-tagtgtcttggtggtggttggtggggagaggatccatgccctgctgaat
    GCA_025215865.1#JAMSCS010000321.1_8136-8584            gagcggctattacgtaagtggatgacgctcaataacgcaattcatc-atgtgcctgtctattaatacatc-tagtgtcttggtgctggttggtggggagaagatccatgccctgctgaat
    GCA_025215865.1#JAMSCS010000173.1_620-1068             gagcggctaatacataagtcgatgacgctcagcaacgcaattcatc-atgtgcctgtctattaatgtatc-tagtgtcttggtgctggttggtggggaactgatccatgccttgctgaat
    GCA_025215985.1#JAMSDF010000755.1_3429-3877            ttccggctattacgtaagtcgatgacgctcaacaacgtaattcatt-atgtacctgtctattaatgcacc-tagtgtcttggtgttggttggtggggagaggatccatgccctgctgaat
    GCA_025216965.1#JAMSEY010000527.1_112-558              gagcggcgattacgtaagtcgatgacgctcaacaacgtaattcatc-atgtgcctgtctattaatgtatc-tagtgtcttggtgctggttggtggggaactgatccatgccctgctgaat
    GCA_025216965.1#JAMSEY010000698.1_1866-2314            gagcggcgattacgtaagtcgatgacgctcaacaacgcaattcatc-atgtgcctgtctattaatgcata-tagtgtcttggtgctggttggtggggaaatgatccatgccctgctgaat
    GCA_025217005.1#JAMSEZ010000669.1_2174-2622            gagcggcgattacgtaagtcgatgacgctcaacaacgcaattcatc-atgtgcctgtctattaatgcata-tagtgtcttggtgctggttggtggggaaatgatccatgccctgctgaat
    GCA_025217005.1#JAMSEZ010000515.1_9896-10342           gagcggcgattacgtaagtcgatgacgctcaacaacgtaattcatc-atgtgcctgtctattaatgtatc-tagtgtcttggtgctggttggtggggaactgatccatgccctgctgaat
    GCA_025217045.1#JAMSFA010000530.1_112-558              gagcggcgattacgtaagtcgatgacgctcaacaacgtaattcatc-atgtgcctgtctattaatgtatc-tagtgtcttggtgctggttggtggggaactgatccatgccctgctgaat
    GCA_025217045.1#JAMSFA010000726.1_112-560              gagcggcgattacgtaagtcgatgacgctcaacaacgcaattcatc-atgtgcctgtctattaatgcata-tagtgtcttggtgctggttggtggggaaatgatccatgccctgctgaat
    GCA_027886205.1#RZGM01000180.1_8903-9341               cgacggcttttacgtaagtcgataaagctcaacaacgtaattcaac-atgtgcatgtgtattaatatatt-tagtgtctcagtaatggctggtggggaggggatccatgccctgctgaat
    GCA_027945405.1#JAOQBI010000031.1_402171-402615        ttcaggctattacgtaggtcgatggcgctcaacaacggaattcatc-atgtgcctgtctattaatgcatc-tagtgtcttagtgatggttggtggggaacggatccatgtcctgctgaat
    GCA_027945555.1#JAOQAY010000137.1_8199-8643            ttcaggctattacgtaggtcgatggcgctcaacaacggaattcatc-atgtgcctgtctattaatgcatc-tagtgtcttagtgatggttggtggggaacggatccatgtcctgctgaat
    GCA_027946385.1#JAPEUR010000436.1_787-1225             cgacggcttttacgtaagtcgataaagctcaacaacgtagttcaac-atgtgcatgtgtattaacatatt-tcgtgtctcagtaatggctggtggggaggggatccatgccctgctgaat
    GCA_029607815.1#JAKCXH010001036.1_885-1334             aagtggctactatgtatgtgtataacccttagtaacgtcacttattgatttgaccgctcgttaacacatc-cagtgtcttgggaatggctggtggggtgtggatccatgccctagggagt
    GCA_030064525.1#JARFYI010000012.1_2234554-2235001      gagcggctattacgtaagtcgatgacgctcaacaacgcaattcatc-atgtgtctgtctattaatgcatc-tagtgtcttggtgctggttggtggggaactgatccatgccctgctgaat
    GCA_032884055.1#JAULRZ010000496.1_1817-2265            ttccggctattacgtaagtcgatgacgctcaacaacgtaattcatt-atgtgcctgtctattaatgcacc-tagtgtcttggtgctggttggtggggagaggatccatgccctgctgaat
    GCA_033782975.1#JAWJZW010004347.1_1810-2253            ctccggctattacgtaagtcgacgatgctcaaaaacgtaatacatt-atgtgccta--tattaatgaatc-tagtgccttggtggtggttggtggggagaagacccatgccctgctgaat
    GCA_033782995.1#JAWJZX010004021.1_7189-7632            ctccggctattacgtaagtcggcgatgctcaaaaacgtaatacatt-atgtgccta--tattaatgaatc-tagtgccttggtggtggttggtggggagaagacccatgccctgctgaat
    GCA_034642005.1#JAWNYV010000013.1_1595757-1596200      ctccggctattacgtaagtcgacgatggtcaaaaacgtaatacatt-atgtgccta--tattaatgaatc-tagtgccttggtggtggttggtggggagaagacccatgccctgctgaat
    GCA_035773055.1#JAPMJO010000013.1_58458-58906          gagcggatattacgtaagtcgatgacgctcaacaacgcaattcatc-atgtgcctgtctattaatgcatc-tagtgtcttggtgctggttggtggggaactgatccatgccctgctgaat
    GCA_037126525.1#JAZDSW010000046.1_287452-287824        gagcggctattatgtaagtcgatgacgctcaaaaacgtaattcatc-atgtgcctgtctattaatgcatg-tagtgtcttggtgatggttggtggggaacggatccatgccctgctgaat
    GCA_038502765.1#JALBXV010000004.1_150133-150579        ttccggctattacgtaagtcgatgacgctcaaaaatgtaattcatt-atgtgcctg--tattaatgcatc-tagtgtcttggtgctggttggtggggagaagatccatgccctgctgaat
    GCA_038502765.1#JALBXV010000003.1_1183198-1183644      ttccggctattacgtaagtcgatgacgctcaaagatgtaattcatt-atgtgcctg--tattaatgcatc-tagtgtcttggtgctggttggtggggagaagatccatgccctgctgaat
    GCA_040938085.1#JBDQYN010000095.1_4849420-4849866      ttccggctattacgtaagtcgatgacgctttaccatgtagttcatt-atgtgcctg--tattaatgcatc-tagtgtcttggtgccggttggtggggagaggatccatgccctgctgaat
    GCA_041380375.1#JAZEUI010000212.1_778435-778881        ttccggctattacgtaagtcgatgacgctttaccatgtagttcatt-atgtgcctg--tattaatgcatc-tagtgtcttggtgccggttggtggggagaggatccatgccctgctgaat
    GCA_041380545.1#JAZEUM010000394.1_2965598-2966044      ttccggctattacgtaagtcgatgacgctttaccatgtagttcatt-atgtgcctg--tattaatgcatc-tagtgtcttggtgccggttggtggggagaggatccatgccctgctgaat
    GCF_000271745.1#NW_022158520.1_5103546-5103992         ttccggctattacgtaagtcgatgacgctttaccatgtagttcatt-atgtgcctg--tattgatgcatc-tagtgtcttggtgccggttggtggggagaggatccatgccctgctgaat
    GCF_020744475.1#NW_025763681.1_315043-315491           ttccggctattacgtaagtcgatggcgctcaactacgtaattcatt-atgtgcctgtctattaatgcatc-tagtgtcttggtgctggttggtggggagaggatccatgccctgctgaat

    Selected Cols:                                                                                                                                                                 

    Gaps Scores:                                                                                                                                                                   
    Similarity Scores:                                                                                                                                                             

                                                                  370       380       390       400       410       420       430       440       450       460
                                                           =========+=========+=========+=========+=========+=========+=========+=========+=========+=========+=====
    GCA_000260075.2#JH651001.1_53017-53465                 acgggaaaggcttcgaggttattagtatgt-aaatcca----tgtgtcggcacgccgtaagcacaacgaatgttgctaattgcattttagac-ttcaacacgtaa
    GCA_000271745.2#JH717840.1_5103546-5103992             acgggaaaggcttcgtggttgttagtatgt-atatcca----tgtgccggcacgacgtaagcacaacgaaaattgctaattgcatttcagac-ttcaacacgtaa
    GCA_000400815.2#CP009080.1_1179336-1179785             atggaaatggctttgaggttgttagtatgt-gaatctaagcccgagtcagcacctctcaggcactgagattgttactaat--cgttgtagtc-tttaacgtataa
    GCA_000585705.1#AEYB01001584.1_5717-6165               acgggaacaccttcgaggttattagtatgt-aaatcaa----catgtcggcatgccagaagcacttcgagtattgctaattacattttagac-tttaatacgtaa
    GCA_001599515.1#BCHB01000008.1_128823-129266           atgggaaaggcttcgaggttgttagtgagt-aaatcca----tttgtcggcacgccgtaagtacaacga---ttgctgactgtattttagac-ttcaacacttaa
    GCA_001680525.1#MADZ01000944.1_3517-3965               acgggaacaccttcgaggttattagtatgt-aaatcaa----catgtcggcatgccagaagcacttcgagtattgctaattacattttagac-tttaatacgtaa
    GCA_001680535.1#MAED01002951.1_1336-1785               acgggaacaccttcgaggttattagtatgt-aaatcaa----catgtcggcatgccagaagcacttcgagtattgctaattacattttagac-tttaatacttaa
    GCA_001680595.1#MAEE01000161.1_9576-10025              acgggaacaccttcgaggttattagtatgt-aaatcaa----catgtcggcatgccagaagcacttcgagtattgctaattacattttagac-tttaatacttaa
    GCA_001680605.1#MADY01000512.1_3407-3855               acgggaacaccttcgaggttattagtatgt-aaatcaa----catgtcggcatgccagaagcacttcgagtattgctaattacattttagac-tttaatacgtaa
    GCA_001680665.1#MADX01001027.1_5081-5529               acgggaacaccttcgaggttattagtatgt-aaatcaa----catgtcggcatgccagaagcacttcgagtattgctaattacattttagac-tttaatacgtaa
    GCA_001680685.1#MAEC01002105.1_1371-1819               acgggaacaccttcgaggttattagtatgt-aaatcaa----catgtcggcatgccagaagcacttcgagtattgctaattacattttagac-tttaatacgtaa
    GCA_001680725.1#MAEF01000026.1_12451-12900             acgggaacaccttcgaggttattagtatgt-aaatcaa----catgtcggcatgccagaagcacttcgagtattgctaattacattttagac-tttaatacttaa
    GCA_001931975.2#MSJJ02000021.1_21498-21944             acgggaaaggcttcgtggttgttagtatgt-atatcca----tgtgccggcacgacgtaagcacaacgaaaattgctaattgcatttcagac-ttcaacacgtaa
    GCA_002233775.1#NJCV01000665.1_343-790                 atgggaaaggcttcgaggttgttagtatgt-aaatcca----tgtgtcggcacgctgtaagcacaatgaatgttgctaatcgcattttagac-ttcaacacgtaa
    GCA_002233895.1#NJCL01000052.1_6292-6740               acgggaaaggcttcgaggttattagtatgt-aaatcca----tgtgtcggcacgccgtaagcacaaggaatgttgctaatcacattttagac-ttcaacacgtaa
    GCA_002233895.1#NJCL01000986.1_2608-3056               acgggaaaggcttcgaggttgttagtatgt-aaatcca----tgtgtcggcacgccgtaagcacaacgaatgttgctaattgcattttagcc-ttcaacacgtaa
    GCA_002233895.1#NJCL01000862.1_714-1162                acgggaaaagcttcgaggttattagtatgt-aaatcca----tgtgtcggcacgccgtaagcacaacgaatgttgctaattgcattttagac-ttcaaaacgtaa
    GCA_002233915.1#NJCM01002567.1_585-1030                acgggaaaggcttcgaggttgttagtatgt-aaatcca----tgtgtcgacacgccgtaagcacaacgaatgttgctaattgcattttagcc-ttcaacacgtaa
    GCA_002233915.1#NJCM01000349.1_3242-3690               acgggaaaggcttcgaggttgttagtatgt-aaatcca----tgtgtcggcacgccgtaagcacaacgaatgttgctaattgcattttagcc-ttcaacacgtaa
    GCA_002233935.2#NJBT02000074.1_114534-114981           acgggaaaggcttcgaggttgttagtatgt-aaatcca----tatgtcggcacgccgtaagcacaatgaatgttgctaa-tgcattttagac-ttcaacacgtaa
    GCA_002234115.1#NJCF01001371.1_90-531                  acgggaaaggcttcgaggttgttagtatgt-aaatcca----tgtgtcggcacgccgtaagcacaacgtatgttgctaattgctttttagac-ttcaaaacgtaa
    GCA_002234115.1#NJCF01000302.1_3435-3883               acgggaaaagcttcgaggttattagtatgt-aaatcca----tgtgtcggcacgccgtaagcacaacgaatgttgctaattgcattttagac-ttcaaaacgtaa
    GCA_002234115.1#NJCF01000961.1_5490-5938               acgggaaaggcttcgaggttattagtatgt-aaatcca----tgtgtcggcacgccgtaagcacaaggaatgttgctaatcacattttagac-ttcaacacgtaa
    GCA_002234195.1#NJCK01002649.1_264-712                 acgggaaaggcttcgaggttgttagtatgt-aaatcca----tgtgtcggcacgccgtaagcacaacgaatgttgctaattgcattttagcc-ttcaacacgtaa
    GCA_002234205.1#NJCQ01003026.1_1778-2226               atgggaaaggcttcgaggttgttagtatgt-aaatcca----tgtgtcggcacgccgtaagcataacgaatgttgctaattgcattttagac-ttcaacacgtaa
    GCA_002234205.1#NJCQ01001471.1_358-806                 acgggaaaggcttcgaggttgttagtatgt-aaatcca----tgtgtcggcacggcgtaagcacaacgaatgttgctaattgcattttagac-ttcaacacgtaa
    GCA_002234235.1#NJCR01003189.1_7833-8281               atgggaaaggcttcgaggttgttagtatgt-aaatcca----tgtgtcggcacgccgtaagcataacgaatgttgctaattgcattttagac-ttcaacacgtaa
    GCA_002234235.1#NJCR01000523.1_1652-2100               acgggaaaggcttcgaggttgttagtatgt-aaatcca----tgtgtcggcacggcgtaagcacaacgaatgttgctaattgcattttagac-ttcaacacgtaa
    GCA_002234255.1#NJCS01000197.1_9074-9523               acgggaaaggcttcgaggttattagtatat-aaatcca----tgtgtcggcacgctgtaagcacaatgaatgttgctaattgcattttagac-ttcaatacgtaa
    GCA_002234255.1#NJCS01000257.1_23088-23528             atgggaaaggcttcgaggttattagtatgt-aaatcca----tgtgtcggtacgctgtaagcacaatgaatgttgctaattgcattttagac-tttaatacgtaa
    GCA_002234285.1#NJCT01000251.1_2006-2454               acgggaaaagcttcgaggttattagtatgt-aaatcca----tgtgtcggcacgccgtaagcacaacgaatgttgctaattgcattttagac-ttcaaaacgtaa
    GCA_002234285.1#NJCT01000112.1_2108-2556               acgggaaaggcttcgaggttattagtatgt-aaatcca----tgtgtcggcacgccgtaagcacaaggaatgttgctaatcacattttagac-ttcaacacgtaa
    GCA_002776445.1#PDEZ01000231.1_7918-8368               atggaaatggctttgaggttgttagtatgt-gaatctaagcccgagtcagcacctctcaggcactgagattgttactaat--cgttgtagtcttttaacgtataa
    GCA_002892985.1#MPSH01000001.1_2159685-2160134         atggaaatggctttgaggttgttagtatgt-gaatctaagcccgagtcagcacctctcaggcactgagattgttactaat--cgttgtagtc-tttaacgtataa
    GCA_002893025.1#PHNV01000722.1_4459-4908               atggaaatggctttgaggttgttagtatgt-gaatctaagcccgagtcagcacctctcaggcactgagattgttactaat--cgttgtagtc-tttaacgtataa
    GCA_002893035.1#PHNW01000920.1_4459-4908               atggaaatggctttgaggttgttagtatgt-gaatctaagcccgagtcagcacctctcaggcactgagattgttactaat--cgttgtagtc-tttaacgtataa
    GCA_002982035.1#PVPY01001450.1_5032-5474               acgggaaaggcttcgaggttgttagtgagt-aaaccca----tgtgtcggcacgccataagtacaacga---ttgctaactgcattttagac-tttaacacgtaa
    GCA_002982055.1#PVPZ01001881.1_721-1163                acgggaaaggcttcgaggttgttagtgagt-aaaccca----tatgtcggcacgccataagtacaacga---ttgctaactgcattttagac-tttaacacgtaa
    GCA_003025205.1#PXUO01000119.1_69286-69732             acgggaaaggcttcgtggttgttagtatgt-atatcca----tgtgccggcacgacgtaagcacaacgaaaattgctaattgcatttcagac-ttcaacacgtaa
    GCA_003025235.1#PXUN01000132.1_66066-66512             acgggaaaggcttcgtggttgttagtatgt-atatcca----tgtgccggcacgacgtaagcacaacgaaaattgctaattgcatttcagac-ttcaacacgtaa
    GCA_004109745.1#RSDZ01000158.1_4397-4846               atggaaatggctttgaggttgttagtatgt-gaatctaagcccgagtcagcacctctcaggcactgagattgttactaat--cgttgtagtc-tttaacgtataa
    GCA_004141715.1#MQTW01000814.1_378-825                 atgggaaaggcttcgaggttgttagtatgt-aaatcca----tgtgtcggcacgctgtaagcacaatgaatgttgctaatcgcattttagac-ttcaacacgtaa
    GCA_004291455.1#QUWZ01000068.1_80333-80705             acgggaaaggcttcgaggttgtt----------------------------------------------------------------------------------
    GCA_004292535.1#QUXA01000044.1_77168-77540             acgggaaaggcttcgaggttgtt----------------------------------------------------------------------------------
    GCA_009297365.1#WGOL01000900.1_612-1060                acgggaaaggcttcgaggttgttagtatgt-aaatcca----tgtgtcggcacgccttaagcataacgaatgttgctaattgcattttagac-ttcaacacgtaa
    GCA_009297515.1#WGOI01001607.1_1477-1925               acgggaaaggcttcgaggttattagtatgt-aaatcca----tgtgtcggcacgccgtaagcacaacgaatgttgctaattgcattttagac-ttcaacacgtaa
    GCA_009297675.1#WGOJ01003344.1_1223-1671               acgggaaaggcttcgaggttgttagtatgt-aaatcca----tgtgtcggcacgccgtaagcacaacgaatgttgctaaatgcattttagac-ttcaacacgtaa
    GCA_009298545.1#WGQK01000668.1_4136-4584               acgggaaaggcttcgaggctattagtatgt-aaatcca----tgtgtcggcacgccgtaaccacaacgaatgttgctaattgaattttagac-ttcaacacgtaa
    GCA_009298855.1#WGQN01001540.1_1461-1906               acgggaaaggcttcgaggttattagtatgt-aaatcca----tgtgtcggcacgccgtaagcacaacgaatgttgctaattgcattttagac-ttcaacacgtaa
    GCA_009298985.1#WGRN01000796.1_1047-1495               atgggaaaggcttcgaggttgttagtatgt-atatcca----tgtgtcggcacgccgtaagcacaacgaatgtttctaattgcattttagac-ttcaacacgtaa
    GCA_009299045.1#WGRK01001155.1_826-1274                acgggaaaggcttcgaggttgttagtatgt-aaatcca----tgtgtcggcacgccgtaagcacaacgaatgttgctaattgcattttagac-ttcaacacgtaa
    GCA_009299075.1#WGRJ01000863.1_12409-12855             acgggaaaggcttcgtggttgttagtatgt-atatcca----tgtgccggcacgacgtaagcacaacgaaaattgctaattgcatttcagac-ttcaacacgtaa
    GCA_009299115.1#WGRH01001009.1_1459-1907               acgggaaaggcttcgaggctattagtatgt-aaatcca----tgtgtcggcacgccgtaaccacaacgaatgttgctaattgaattttagac-ttcaacacgtaa
    GCA_009299215.1#WGRS01000248.1_42092-42540             acgggaaaggcttcgaggttattagtatgt-aaatcca----tgtgtcggcacgccgtaagcacaacgaatgttgctaattgcattttagac-ttcaacacgtaa
    GCA_009299215.1#WGRS01000488.1_939-1388                acgggaaaggcttcgaggttattagtatgt-aaatcca----tgtgtcggcacgccgtaagcacaacgaatgttgctaattgcattttagac-ttcaacacgtaa
    GCA_009299215.1#WGRS01000332.1_7989-8437               acgggaaaggcttcgaggttgttagtatgt-aaatcca----tgtgtcggcacgccgtaagcacaacgaatgttgctaattgcattttagac-ttcaacacgtaa
    GCA_009663885.1#WJDV01000002.1_418952-419401           atggaaatggctttgaggttgttagtatgt-gaatctaagcccgagtcagcacctctcaggcactgagattgttactaat--cgttgtagtc-tttaacgtataa
    GCA_009746015.1#VLJC01000049.1_70684-71130             acgggaaaggcttcgtggttgttagtatgt-atatcca----tgtgccggcacgacgtaagcacaacgaaaattgctaattgcatttcagac-ttcaacacgtaa
    GCA_011032855.1#WESG01000339.1_10823-11271             acgggaaaggcttcgaggttgttagtatgt-aaatcca----tgtatcggcacgccgtaagcacaacgaatgttgctaatcgcattttagac-ttcaacacgtaa
    GCA_011032945.1#WNXM01001784.1_7328-7777               atggaaatggctttgaggttgttagtatgt-gaatctaagcccgagtcagcacctctcaggcactgagattgttactaat--cgttgtagtc-tttaacgtataa
    GCA_011032955.1#WNXL01001437.1_4250-4699               atggaaatggctttgaggttgttagtatgt-gaatctaagcccgagtcagcacctctcaggcactgagattgttactaat--cgttgtagtc-tttaacgtataa
    GCA_011032965.1#WNXK01000961.1_4382-4831               atggaaatggctttgaggttgttagtatgt-gaatctaagcccgagtcagcacctctcaggcactgagattgttactaat--cgttgtagtc-tttaacgtataa
    GCA_011033455.1#WESL01002533.1_4420-4868               acgggaaaggcttcgaggttgttagtatgt-aaatcca----tgtgtcggcacgccgtaagcacaacgaatgttgctaattgcattttagac-ttcaacacgtaa
    GCA_011033455.1#WESL01001216.1_8955-9403               acgggaaaggcttcgaggttgttagtatgt-aaatcca----tgtgtcggcacgccgtaagcacaacgaatgttgctaattgcattctagac-ttcaacacgtaa
    GCA_011033575.1#WESJ01009652.1_394-842                 acgggaaaggcttcgaggttgttagtatgt-aaatcca----tgtgtcggcacgccgtaagcacaacgaatgttgctaattgcattctagac-ttcaacacgtaa
    GCA_011033575.1#WESJ01007920.1_731-1149                acgggaaaggcttcgaggttgttagtatgt-aaatcca----tgtgtcggcacgccgtaagcacaacgaatgtt-------------------------------
    GCA_011033685.1#WESM01008445.1_1386-1834               acgggaaaggcttcgaggttgttagtatgt-aaatcca----tgtgtcggcacgccgtaagcacaacgaatgttgctaattgcattttagac-ttcaacacgtaa
    GCA_011033745.1#WETB01002153.1_7139-7587               acgggaaaggcttcgaggttgttagtatgt-aaatcca----tgtgtcggcacgccgtaagcacaacgaatgttgctaattgcattttagac-ttcaacacgtaa
    GCA_011033805.1#WESY01002113.1_415-863                 acgggaaaggcttcgaggttgttagtatgt-aaatcca----tgtgtcggcacgccgtaagcacaacgaatgttgctaattgcattttagac-ttcaacacgtaa
    GCA_011033815.1#WESW01000773.1_2707-3155               acgggaaaggcttcgaggttgttagtatgt-aaatcca----tgtgtcggcacgccgtaagcacaacgaatgttgctaattgcattttagac-ttcaacacgtaa
    GCA_011033815.1#WESW01000649.1_851-1299                acgggaaaggcttcgaggttgttagtatgt-aaatcca----tgtgtcggcacgccgtaagcacaacgaatgttgctaattgcattctagac-ttcaacacgtaa
    GCA_011033895.1#WETL01000467.1_850-1298                acgggaaaggcttcgaggttgttagtatgt-aaatcca----tgtgtcggcacgccgtaagcacaacgaatgttgctaattgcattctagac-ttcaacacgtaa
    GCA_011033895.1#WETL01000641.1_3860-4278               acgggaaaggcttcgaggttgttagtatgt-aaatcca----tgtgtcggcacgccgtaagcacaacgaatgtt-------------------------------
    GCA_011034195.1#WETT01000020.1_547-995                 acgggaaaggcttcgaggttgttagtatgt-aaatcca----tgtgtcggcacgccgtaagcacaacgaatgttgctaattgcattttagac-ttcaacacgtaa
    GCA_011034205.1#WETS01005722.1_2442-2890               acgggaaaggcttcgaggttgttagtatgt-aaatcca----tgtgtcggcacgccgtaagcacaacgaatgttgctaattgcattttagac-ttcaacacgtaa
    GCA_011034415.1#WEUC01001584.1_7719-8167               acgggaaaggcttcgaggttgttagtatgt-aaatcca----tgtgtcggcacgccgtaagcacaacgaatgttgctaattgcattttagac-ttcaacacgtaa
    GCA_011034445.1#WEUB01001158.1_3523-3971               acgggaaaggcttcgaggttgttagtatgt-aaatcca----tgtgtcggcacgccgtaagcacaacgaatgttgctaattgcattctagac-ttcaacacgtaa
    GCA_011034445.1#WEUB01000999.1_8836-9284               acgggaaaggcttcgaggttgttagtatgt-aaatcca----tgtgtcggcacgccgtaagcacaacgaatgttgctaattgcattttagac-ttcaacacgtaa
    GCA_011034575.1#WEUE01008972.1_493-941                 acgggaaaggcttcgaggttgttagtatgt-aaatcca----tgtgtcggcacgccgtaagcacaacgaatgttgctaattgcattctagac-ttcaacacgtaa
    GCA_011034625.1#WEUR01000714.1_7159-7607               acgggaaaggcttcgaggttgttagtatgt-aaatcca----tgtgtcggcacgccgtaagcacaacgaatgttgctaattgcattttagac-ttcaacacgtaa
    GCA_011034645.1#WEUV01000741.1_2997-3445               acgggaaaggcttcgaggttgttagtaagt-aaatcca----tgtgccggcacgccgtaagcacaacgaatgtcgctaactgcattttagat-ttcaacacataa
    GCA_011034655.1#WEUQ01004890.1_3105-3553               acgggaaaggcttcgaggttgttagtatgt-aaatcca----tgtgtcggcacgccgtaagcacaacgaatgttgctaattgcattttagac-ttcaacacgtaa
    GCA_011034655.1#WEUQ01000935.1_566-1014                acgggaaaggcttcgaggttgttagtatgt-aaatcca----tgtatcggcacgccgtaagcacaacgaatgttgctaatcgcattttagac-ttcaacacgtaa
    GCA_011034775.1#WEVI01000501.1_5488-5930               acgggaaaggcttcgaggttgttagtatgt-aaatcca----tgtgtcggcacgccgtaagcacaacgaatgttgctaattgcattctagac-ttcaac------
    GCA_011034775.1#WEVI01000783.1_2046-2494               acgggaaaggcttcgaggttgttagtatgt-aaatcca----tgtgtcggcacgccgtaagcacaacgaatgttgctaattgcattttagac-ttcaacacgtaa
    GCA_011034815.1#WEVE01000912.1_9755-10173              acgggaaaggcttcgaggttgttagtatgt-aaatcca----tgtgtcggcacgccgtaagcacaacgaatgtt-------------------------------
    GCA_011034815.1#WEVE01002086.1_1620-2038               acgggaaaggcttcgaggttgttagtatgt-aaatcca----tgtgtcggcacgccgtaagcacaacgaatgtt-------------------------------
    GCA_011034845.1#WEVA01001230.1_1747-2195               acgggaaaggcttcgaggttgttagtatgt-aaatcca----tgtgtcggcacgccgtaagcacaacgaatgttgctaattgcattttagac-ttcaacacgtaa
    GCA_011035075.1#WEVS01001164.1_694-1142                acgggaaaggcttcgaggttgttagtatgt-aaatcca----tgtgtcggcacgccgtaagcacaacgaatgttgctaattgcattttagac-ttcaacacgtaa
    GCA_011035185.1#WEVO01012916.1_426-874                 acgggaaaggcttcgaggttgttagtatgt-aaatcca----tgtgtcggcacgccgtaagcacaacgaatgttgctaattgcattttagac-ttcaacacgtaa
    GCA_011035185.1#WEVO01003306.1_4368-4816               acgggaaaggcttcgaggttgttagtatgt-aaatcca----tgtgtcggcacgccgtaagcacaacgaatgttgctaattgcattctagac-ttcaacacgtaa
    GCA_011035205.1#WEVG01003463.1_75-523                  acgggaaaggcttcgaggttgttagtatgt-aaatcca----tgtgtcggcacgccgtaagcacaacgaatgttgctaattgcattttagac-ttcaacacgtaa
    GCA_011035235.1#WEWM01001159.1_2667-3085               acgggaaaggcttcgaggttgttagtatgt-aaatcca----tgtgtcggcacgccgtaagcacaacgaatgtt-------------------------------
    GCA_011035235.1#WEWM01001540.1_2265-2683               acgggaaaggcttcgaggttgttagtatgt-aaatcca----tgtgtcggcacgccgtaagcacaacgaatgtt-------------------------------
    GCA_011035245.1#WEWK01000657.1_7022-7440               acgggaaaggcttcgaggttgttagtatgt-aaatcca----tgtgtcggcacgccgtaagcacaacgaatgtt-------------------------------
    GCA_011035245.1#WEWK01000404.1_16648-17029             acgggaaaggcttcgaggttgttagtatgt-aaatcca----tgtgtcggcacgccgtaagcacaacgaatgttgctaattgcattttagac-ttcaacacgtaa
    GCA_011035255.1#WEWN01000527.1_2412-2830               acgggaaaggcttcgaggttgttagtatgt-aaatcca----tgtgtcggcacgccgtaagcacaacgaatgtt-------------------------------
    GCA_011035255.1#WEWN01000751.1_2320-2738               acgggaaaggcttcgaggttgttagtatgt-aaatcca----tgtgtcggcacgccgtaagcacaacgaatgtt-------------------------------
    GCA_011035265.1#WEWJ01000412.1_17167-17548             acgggaaaggcttcgaggttgttagtatgt-aaatcca----tgtgtcggcacgccgtaagcacaacgaatgttgctaattgcattttagac-ttcaacacgtaa
    GCA_011035265.1#WEWJ01000569.1_529-977                 acgggaaaggcttcgaggttgttagtatgt-aaatcca----tgtgtcggcacgccgtaagcacaacgaatgttgctaattgcattttagac-ttcaacacgtaa
    GCA_011035345.1#WEWE01000725.1_7055-7503               acgggaaaggcttcgaggttgttagtatgt-aaatcca----tgtgtcggcacgccgtaagcacaacgaatgttgctaattgcattttagac-ttcaacacgtaa
    GCA_011035345.1#WEWE01000822.1_176-557                 acgggaaaggcttcgaggttgttagtatgt-aaatcca----tgtgtcggcacgccgtaagcacaacgaatgttgctaattgcattttagac-ttcaacacgtaa
    GCA_011035375.1#WEWB01000636.1_176-557                 acgggaaaggcttcgaggttgttagtatgt-aaatcca----tgtgtcggcacgccgtaagcacaacgaatgttgctaattgcattttagac-ttcaacacgtaa
    GCA_011035375.1#WEWB01000586.1_665-1113                acgggaaaggcttcgaggttgttagtatgt-aaatcca----tgtgtcggcacgccgtaagcacaacgaatgttgctaattgcattttagac-ttcaacacgtaa
    GCA_011035435.1#WEWD01000787.1_1777-2225               acgggaaaggcttcgaggttgttagtatgt-aaatcca----tgtgtcggcacgccgtaagcacaacgaatgttgctaattgcattttagac-ttcaacacgtaa
    GCA_011035455.1#WEVZ01000295.1_5889-6270               acgggaaaggcttcgaggttgttagtatgt-aaatcca----tgtgtcggcacgccgtaagcacaacgaatgttgctaattgcattttagac-ttcaacacgtaa
    GCA_011035485.1#WEVY01000687.1_5745-6126               acgggaaaggcttcgaggttgttagtatgt-aaatcca----tgtgtcggcacgccgtaagcacaacgaatgttgctaattgcattttagac-ttcaacacgtaa
    GCA_011035485.1#WEVY01000540.1_528-976                 acgggaaaggcttcgaggttgttagtatgt-aaatcca----tgtgtcggcacgccgtaagcacaacgaatgttgctaattgcattttagac-ttcaacacgtaa
    GCA_011035495.1#WEVW01000533.1_1949-2397               acgggaaaggcttcgaggttgttagtatgt-aaatcca----tgtgtcggcacgccgtaagcacaacgaatgttgctaattgcattttagac-ttcaacacgtaa
    GCA_011035625.1#WEWS01001096.1_809-1227                acgggaaaggcttcgaggttgttagtatgt-aaatcca----tgtgtcggcacgccgtaagcacaacgaatgtt-------------------------------
    GCA_011035625.1#WEWS01001310.1_2416-2834               acgggaaaggcttcgaggttgttagtatgt-aaatcca----tgtgtcggcacgccgtaagcacaacgaatgtt-------------------------------
    GCA_011035755.1#WEXC01000589.1_22209-22657             acgggaaaggcttcgaggttgttagtatgt-aaatcca----tgtatcggcacgccgtaagcacaacgaatgttgctaatcgcattttagac-ttcaacacgtaa
    GCA_011035835.1#WEXF01000545.1_8821-9239               acgggaaaggcttcgaggttgttagtatgt-aaatcca----tgtgtcggcacgccgtaagcacaacgaatgtt-------------------------------
    GCA_011035835.1#WEXF01000653.1_3838-4256               acgggaaaggcttcgaggttgttagtatgt-aaatcca----tgtgtcggcacgccgtaagcacaacgaatgtt-------------------------------
    GCA_011035975.1#WEXJ01007704.1_1039-1487               acgggaaaggcttcgaggttgttagtatgt-aaatcca----tgtgtcggcacgccgtaagcacaacgaatgttgctaattgcattttagac-ttcaacacgtaa
    GCA_011035995.1#WEXI01000903.1_7456-7904               acgggaaaggcttcgaggttgttagtatgt-aaatcca----tgtgtcggcacgccgtaagcacaacgaatgttgctaattgcattttagac-ttcaacacgtaa
    GCA_011035995.1#WEXI01001093.1_807-1255                acgggaaaggcttcgaggttgttagtatgt-aaatcca----tgtatcggcacgccgtaagcacaacgaatgttgctaatcgcattttagac-ttcaacacgtaa
    GCA_011036045.1#WEXT01000869.1_163-611                 atgggaaaggcttcgaggttgttagtatgt-atatcca----tgtgtcggcacgccgtaagcacaacgaatgtttctaattgcattacagac-ttcaacacgtaa
    GCA_011036135.1#WEXQ01000241.1_21717-22165             acgggaaaggcttcgaggttgttagtatgt-aaatcca----tgtatcggcacgccgtaagcacaacgaatgttgctaatcgcattttagac-ttcaacacgtaa
    GCA_011036135.1#WEXQ01000570.1_528-976                 acgggaaaggcttcgaggttgttagtatgt-aaatcca----tgtgtcggcacgccgtaagcacaacgaatgttgctaattgcattttagac-ttcaacacgtaa
    GCA_011036215.1#WEYG01000053.1_6223-6671               atgggaaaggcttcgaggttgttagtatgt-atatcca----tgtgtcggcacgccgtaagcacaacgaatgtttctaattgcattacagac-ttcaacacgtaa
    GCA_011036305.1#WEXY01003662.1_3416-3864               acgggaaaggcttcgaggttgttagtatgt-aaatcca----tgtgtcggcacgccgtaagcacaacgaatgttgctaattgcattctagac-ttcaacacgtaa
    GCA_011036305.1#WEXY01002318.1_5546-5994               acgggaaaggcttcgaggttgttagtatgt-aaatcca----tgtgtcggcacgccgtaagcacaacgaatgttgctaattgcattctagac-ttcaacacgtaa
    GCA_011036365.1#WEYQ01001970.1_647-1095                acgggaaaggcttcgaggttgttagtatgt-aaatcca----tgtgtcggcacgccgtaagcacaacgaatgttgctaattgcattttagac-ttcaacacgtaa
    GCA_011036595.1#WEYT01000407.1_15167-15585             acgggaaaggcttcgaggttgttagtatgt-aaatcca----tgtgtcggcacgccgtaagcacaacgaatgtt-------------------------------
    GCA_011036595.1#WEYT01000705.1_8787-9235               acgggaaaggcttcgaggttgttagtatgt-aaatcca----tgtgtcggcacgccgtaagcacaacgaatgttgctaattgcattctagac-ttcaacacgtaa
    GCA_011036765.1#WEUS01002153.1_588-1036                acgggaaaggcttcgaggttgttagtatgt-aaatcca----tgtgtcggcacgccgtaagcacaacgaatgttgctaattgcattttagac-ttcaacacgtaa
    GCA_011036795.1#WEUZ01000271.1_2987-3435               acgggaaaggcttcgaggttgttagtaagt-aaatcca----tgtgccggcacgccgtaagcacaacgaatgtcgctaactgcattttagat-ttcaacacataa
    GCA_011036875.1#WEWI01000026.1_11685-12103             acgggaaaggcttcgaggttgttagtatgt-aaatcca----tgtgtcggcacgccgtaagcacaacgaatgtt-------------------------------
    GCA_011036875.1#WEWI01000910.1_3838-4256               acgggaaaggcttcgaggttgttagtatgt-aaatcca----tgtgtcggcacgccgtaagcacaacgaatgtt-------------------------------
    GCA_011037075.1#WEZD01015082.1_365-770                 acgggaaaggcttcgaggttgttagtatgt-aaatcca----tgtgtcggcacgccgtaagcacaacgaatgttgctaattgcattttagac-ttcaacacgtaa
    GCA_011421275.1#WIKV01000306.1_26907-27325             acgggaaaggcttcgaggttgttagtatgt-aaatcca----tgtgtcggcacgccgtaagcacaacgaatgtt-------------------------------
    GCA_011421275.1#WIKV01000622.1_3838-4256               acgggaaaggcttcgaggttgttagtatgt-aaatcca----tgtgtcggcacgccgtaagcacaacgaatgtt-------------------------------
    GCA_011421285.1#WIKU01001812.1_697-1145                acgggaaaggcttcgaggttgttagtatgt-aaatcca----tgtgtcggcacgccgtaagcacaacgaatgttgctaattgcattttagac-ttcaacacgtaa
    GCA_011421305.1#WIKW01002869.1_809-1227                acgggaaaggcttcgaggttgttagtatgt-aaatcca----tgtgtcggcacgccgtaagcacaacgaatgtt-------------------------------
    GCA_011421305.1#WIKW01002126.1_1789-2207               acgggaaaggcttcgaggttgttagtatgt-aaatcca----tgtgtcggcacgccgtaagcacaacgaatgtt-------------------------------
    GCA_011421375.1#WILC01001342.1_696-1144                acgggaaaggcttcgaggttgttagtatgt-aaatcca----tgtgtcggcacgccgtaagcacaacgaatgttgctaattgcattttagac-ttcaacacgtaa
    GCA_011426335.1#WILD01001610.1_1018-1466               acgggaaaggcttcgaggttgttagtatgt-aaatcca----tgtgtcggcacgccgtaagcacaacgaatgttgctaattgcattttagac-ttcaacacgtaa
    GCA_011426355.1#WILF01007384.1_372-820                 acgggaaaggcttcgaggttgttagtatgt-aaatcca----tgtgtcggcacgccgtaagcacaacgaatgttgctaattgcattttagac-ttcaacacgtaa
    GCA_011426355.1#WILF01000505.1_6553-7001               acgggaaaggcttcgaggttgttagtatgt-aaatcca----tgtgtcggcacgccgtaagcacaacgaatgttgctaattgcattttagac-ttcaacacgtaa
    GCA_013170945.1#MU047874.1_170025-170474               atggaaatggctttgaggttgttagtatgt-gaatctaagcccgagtcagcacctctcaggcactgagattgttactaat--cgttgtagtc-tttaacgtataa
    GCA_013184365.1#JABCJX010000598.1_3113-3561            atgggaaaggcttcgaggttgttagtatgt-aaatcca----tgtgtcggcacgccataagcgcaacgaaggttgctaattgcattttagac-ttcaacacgtaa
    GCA_013266185.1#JABEXW010001135.1_3715-4163            acgggaaaggcttcgaggttgttagtatgt-gaatcca----tgtgtcggcacgccggaagcccaacgaatgttgctaattacattttagac-ttcaacacataa
    GCA_013347355.2#JAALGN020000013.1_426461-426909        acgggaaaggcttcgaggttattagtatgt-aaatcca----tgtgtcggcacgccgtaagcacaacgaatgttgctaattgcattttagac-ttcaacacgtaa
    GCA_013363175.1#JABEEP010001877.1_2080-2528            acgggaacaccttcgaggttattagtatgt-aaatcaa----catgtcggcatgccagaagcacttcgagtattgctaattacattttagac-tttaatacgtaa
    GCA_013396025.1#JAAOAQ010001214.1_4450-4898            acgggaaaggcttcgaggttgttggtatgt-aaatcca----tgtgtcggcacgccgtaagcacaacgaatgttgctaattgcattttagtc-ttcaacacgtaa
    GCA_013396205.1#JAAOAN010000160.1_45920-46352          atggcaaaggctttgaggttgttagtatgt-atatcca----tgtgttgaca-gcggtattcacagtgaatgttgctaattatgttttagac-ttca--------
    GCA_013618355.1#JABFES010000455.1_7017-7460            atgggaaaggcttcgaggttgttagtgagt-aaatcca----tttgtcggcacgccgtaagtgcaacga---ttgctgactgtatttcagac-ttcaacacgtaa
    GCA_013623525.1#JABGLY010000028.1_42787-43233          acgggaaaaccttcgaggttgtcagtatgt-agacctc----tttgtcggcatgccg--agcaaagtaaacgttgctaatatcatttcagac-atcaaca-----
    GCA_013623715.1#JABFFJ010000182.1_85034-85470          atggcaaaaccttctcggttgtcagtaagt-aaatcca----tatatcagcacgccctagatacaatacatgttgctaattgcattttagac-ttc---------
    GCA_014324575.1#WXUM01000474.1_1010-1458               acgggaaaggcttcgaggttattagtatgt-aaatcca----tgtgtcggcacgccgtaagcacaacgaatgttgctaattgcattttagac-ttcaacacgtaa
    GCA_014324595.1#WXUN01001159.1_797-1245                acgggaaaggcttcgaggttattagtatgt-aaatcca----tgtgtcggcacgccgtaagcacaacgaatgttgctaattgcattttagac-ttcaacacgtaa
    GCA_014324665.1#WXUR01000643.1_5483-5930               acgggaaaggcttcgaagttattagtatgt-aaatcca----tgtgtcggcacgccgtaagcacaacgaatgttgctaattgcattttagac-ttcaacacgtaa
    GCA_014324745.1#JAAMUY010001820.1_1146-1594            acgggaaaggcttcgaggttattagtatgt-aaatcca----tgtgtcggcacgccgtaagcacaacgaatgttgctaattgcattttagac-ttcaacacgtaa
    GCA_014324775.1#WXUT01000779.1_3905-4353               acgggaaaggcttcgaggttattagtatgt-aaatcca----tgtgtcggcacgccgtaagcacaacgaatgttgctaattgcattttagac-ttcaacacgtaa
    GCA_014324795.1#WXUU01000551.1_4342-4789               acgggaaaggcttcgaagttattagtatgt-aaatcca----tgtgtcggcacgccgtaagcacaacgaatgttgctaattgcattttagac-ttcaacacgtaa
    GCA_014324835.1#WXUX01001216.1_2785-3233               acgggaaaggcttcgaggttattagtatgt-aaatcca----tgtgtcggcacgccgtaagcacaacgaatgttgctaattgcattttagac-ttcaacacgtaa
    GCA_014324865.1#WXUY01001109.1_5504-5951               acgggaaaggcttcgaagttattagtatgt-aaatcca----tgtgtcggcacgccgtaagcacaacgaatgttgctaattgcattttagac-ttcaacacgtaa
    GCA_014324895.1#WXUZ01000915.1_1546-1994               acgggaaaggcttcgaggttattagtatgt-aaatcca----tgtgtcggcacgccgtaagcacaacgaatgttgctaattgcattttagac-ttcaacacgtaa
    GCA_014325045.1#WXVI01000570.1_8895-9343               acgggaaaggcttcgaggttattagtatgt-aaatcca----tgtgtcggcacgccgtaagcacaacgaatgttgctaattgcattttagac-ttcaacacgtaa
    GCA_014325205.1#JAAMVE010001426.1_3625-4073            acgggaaaggcttcgaggttattagtatgt-aaatcca----tgtgtcggcacgccgtaagcacaacgaatgttgctaattgcattttagac-ttcaacacgtaa
    GCA_014325315.1#WXVP01000164.1_88523-88895             acgggaaaggcttcgaggttgtt----------------------------------------------------------------------------------
    GCA_014843555.1#JAAOOQ010000005.1_239540-239989        acgggaaaggcttccaggttgttagtatgt-aaatcca----tgtatcggcacgccgtaagcacaacgaatgttgctaattgcattttagac-ttcaacacgtaa
    GCA_014843565.1#JAAOOP010000035.1_171592-172041        acgggaaaggcttccaggttgttagtatgt-aaatcca----tgtatcggcacgccgtaagcacaacgaatgttgctaattgcattttagac-ttcaacacgtaa
    GCA_014884825.1#JACTAS010000222.1_17723-18171          acgggaaaggcttcgaggttgttagtatgt-aaatcca----tgtgtcggcacgccgtaagcacaacgaatgttgctaattgcattttagac-ttcaacacgtaa
    GCA_018440645.1#JACOQZ010000013.1_178443-178892        atggaaatggctttgaggttgttagtatgt-gaatctaagcccgagtcagcacctctcaggcactgagattgttactaat--cgttgtagtc-tttaacgtataa
    GCA_018440655.1#JACORB010000012.1_54438-54887          atggaaatggctttgaggttgttagtatgt-gaatctaagcccgagtcagcacctctcaggcactgagattgttactaat--cgttgtagtc-tttaacgtataa
    GCA_019055395.1#JAHKNW010006905.1_873-1321             acgggaaaggcttcgaggttattagtatgt-aaatcca----tgtgtcggcacgccgtaagcacaacgaatgttgctaattgcattttagac-ttcaacacgtaa
    GCA_019157295.1#JAELUQ010000008.1_3397787-3398230      atgggaaaggcttcgaggttgttagtgagt-aaatcca----tttgtcggcacgccgtaagtgcaacga---ttgctgactgtattttagac-ttcaacacgtaa
    GCA_019191165.1#JAHARQ010000139.1_67224-67670          acgggaaaggcttcgtggttgttagtatgt-atatcca----tgtgccggcacgacgtaagcacaacgaaaattgctaattgcatttcagac-ttcaacacgtaa
    GCA_019191175.1#JAHARR010000129.1_15625-16071          acgggaaaggcttcgtggttgttagtatgt-atatcca----tgtgccggcacgacgtaagcacaacgaaaattgctaattgcatttcagac-ttcaacacgtaa
    GCA_019843925.1#JACDIY010000477.1_28970-29342          acgggaaaggcttcgaggttgtt----------------------------------------------------------------------------------
    GCA_020726535.1#JAHEWJ010000011.1_1000856-1001305      atggaaatggctttgaggttgttagtatgt-gaatctaagcccgagtcagcacctctcaggcactgagattgttactaat--cgttgtagtc-tttaacgtataa
    GCA_020744475.1#JAGMUX010000018.1_315043-315491        acgggaaaggcttcgaggttattagtatgt-aaatcca----tgtgtcggcacgccgtaagcacaacgaatgttgctaattgcattttagac-ttcaacacgtaa
    GCA_020883595.1#JAJGQA010000014.1_323964-324412        acgggaacaccttcgaggttattagtatgt-aaatcaa----catgtcggcatgccagaagcacttcgagtattgctaattacattttagac-tttaatacgtaa
    GCA_020883605.1#JAJHJC010000014.1_396196-396644        acgggaacaccttcgaggttattagtatgt-aaatcaa----catgtcggcatgccagaagcacttcgagtattgctaattacattttagac-tttaatacgtaa
    GCA_020883615.1#JAJHJD010000017.1_393187-393635        acgggaacaccttcgaggttattagtatgt-aaatcaa----catgtcggcatgccagaagcacttcgagtattgctaattacattttagac-tttaatacgtaa
    GCA_020883625.1#JAJGQB010000012.1_381552-382000        acgggaacaccttcgaggttattagtatgt-aaatcaa----catgtcggcatgccagaagcacttcgagtattgctaattacattttagac-tttaatacgtaa
    GCA_021655895.1#JAJJWO010000410.1_1878-2303            acgggccaggctttgtggttgttagtatgtaaaaccct----tgtatcgaccagccgtaagtataatgagtg--gctaattacgttttagac-ttc---------
    GCA_021730365.1#JAJOOV010000405.1_1213-1662            acgggaacaccttcgaggttattagtatgt-aaatcaa----catgtcggcatgccagaagcacttcgagtattgctaattacattttagac-tttaatacttaa
    GCA_023065405.1#JAJTCY010000003.1_74358-74801          atgggaaaggcttcgaggttgttagtgagt-aaatcca----tttgtcggcacgccgtaagtgcaacga---ttgctgactgtattttagac-ttcaacacgtaa
    GCA_023628715.1#JAMAKD010000122.1_67003-67449          acgggaaaggcttcgtggttgttagtatgt-atatcca----tgtgccggcacgacgtaagcacaacgaaaattgctaattgcatttcagac-ttcaacacgtaa
    GCA_024865645.1#JANKHN010000668.1_8435-8878            atgggaaaggcttcgaggttgttagtgagt-aaatcca----tttgtcggcacgccgtaagtacaacga---ttgctgactgtattttagac-ttcaacacttaa
    GCA_025201995.1#JAMSCT010000483.1_2189-2637            acgggaaaggcttcgaggttgttagtatgt-aaatcca----tgtgtcggcacgccataagcacaacgaatgttgctaattgcattttagac-ttcaacacgtaa
    GCA_025203115.1#JAMSFS010000057.1_218973-219344        acgggaaaggcttcgaggttgtt----------------------------------------------------------------------------------
    GCA_025203755.1#JAMSFW010000517.1_9896-10342           acgggaaaggcttcgaggttgttagtatgt-aaatcta----tgtgtcgtcacgccgtaagcataacgaatgttgctaattgcattttagac-ttcaacacgt--
    GCA_025203755.1#JAMSFW010000664.1_112-560              acgggaaaggcttcgaggttgttagtatgt-aaatcca----tgtgtcggcacgccgtaagcacaacgtatgttgctaattgctttttagac-ttcaaaacgtaa
    GCA_025215475.1#JAMSDB010000983.1_1119-1567            acgggaaaggcttcgaggttattagtatgt-aaatcca----tgtgtcggcacgccgtaagcacaacgaatgttgctaattgcattttagac-ttcaacacgtaa
    GCA_025215845.1#JAMSCV010001163.1_880-1265             acgggaaaggcttcgaggttgtcagtatgt-aaatcca----tgtgtcggcacgtcgtaaacacaacgaatgttgctaatttcattttagac-ttcaaaacgtaa
    GCA_025215865.1#JAMSCS010000321.1_8136-8584            acgggaaaggcttcgaggttgttagtatgt-aaatcca----tgtgtcggcacggcgtaagcacaacgaatgttgctaattgcattttagac-ttcaacacgtaa
    GCA_025215865.1#JAMSCS010000173.1_620-1068             acgggaaaggcttcgaggttgttagtatgt-aaatcca----tgtgtcggcacgccgtaagcacaacgaatgttgctaattgcattttagac-ttcaacacgtaa
    GCA_025215985.1#JAMSDF010000755.1_3429-3877            acgggaaaggcttcgaggttattagtatgt-aaatcca----tgtgtcggcacgccgtaagcacaacgaatgttgctaattgcattttagac-ttcaacacgtaa
    GCA_025216965.1#JAMSEY010000527.1_112-558              acgggaaaggcttcgaggttgttagtatgt-aaatcta----tgtgtcgtcacgccgtaagcataacgaatgttgctaattgcattttagac-ttcaacacgt--
    GCA_025216965.1#JAMSEY010000698.1_1866-2314            acgggaaaggcttcgaggttgttagtatgt-aaatcca----tgtgtcggcacgccgtaagcacaacgtatgttgctaattgctttttagac-ttcaaaacgtaa
    GCA_025217005.1#JAMSEZ010000669.1_2174-2622            acgggaaaggcttcgaggttgttagtatgt-aaatcca----tgtgtcggcacgccgtaagcacaacgtatgttgctaattgctttttagac-ttcaaaacgtaa
    GCA_025217005.1#JAMSEZ010000515.1_9896-10342           acgggaaaggcttcgaggttgttagtatgt-aaatcta----tgtgtcgtcacgccgtaagcataacgaatgttgctaattgcattttagac-ttcaacacgt--
    GCA_025217045.1#JAMSFA010000530.1_112-558              acgggaaaggcttcgaggttgttagtatgt-aaatcta----tgtgtcgtcacgccgtaagcataacgaatgttgctaattgcattttagac-ttcaacacgt--
    GCA_025217045.1#JAMSFA010000726.1_112-560              acgggaaaggcttcgaggttgttagtatgt-aaatcca----tgtgtcggcacgccgtaagcacaacgtatgttgctaattgctttttagac-ttcaaaacgtaa
    GCA_027886205.1#RZGM01000180.1_8903-9341               acgggagaggcttcgaggttattagtatgt-aaatcca----cgtgtcggcacgccgtaagcattgcgaatgttgctaattgcattttagac-tt----------
    GCA_027945405.1#JAOQBI010000031.1_402171-402615        acgggaaaggcttcgaggttgttagtatgt-aaatcca----tgtgtcggcacgccgtaagcacaacgaatgttgctaattgcattttagac-ttcaacac----
    GCA_027945555.1#JAOQAY010000137.1_8199-8643            acgggaaaggcttcgaggttgttagtatgt-aaatcca----tgtgtcggcacgccgtaagcacaacgaatgttgctaattgcattttagac-ttcaacac----
    GCA_027946385.1#JAPEUR010000436.1_787-1225             acgggagaggcttcgaggttattagtatgt-aaatcca----cgtatcgacacgccgtaaacattgcaaatgttgctaattgaattttagac-tt----------
    GCA_029607815.1#JAKCXH010001036.1_885-1334             atggaaatggctttgaggttgttagtatgt-gaatctaagcccgagtcagcacctctcaggcactgagattgttactaat--cgttgtagtc-tttaacgtataa
    GCA_030064525.1#JARFYI010000012.1_2234554-2235001      acgggaaaggcttcgaggttgttagtatgt-aaatcca----tgtgtcggcacgccgtaagcacaacgaatgttgctaattgcattttagac-ttcaacacgtaa
    GCA_032884055.1#JAULRZ010000496.1_1817-2265            acgggaaaggcttcgaggttattagtatgt-aaatcca----tgtgtcggcacgccgtaagcacaacgaatgttgctaattgcattttagac-ttcaacacgtaa
    GCA_033782975.1#JAWJZW010004347.1_1810-2253            atgggaaaggcttcgaggttgttagtgagt-aaatcca----tttgtcggcacgccgtaagtgcaacga---ttgctgactgtatttcagac-ttcaacacgtaa
    GCA_033782995.1#JAWJZX010004021.1_7189-7632            atgggaaaggcttcgaggttgttagtgagt-aaatcca----tttgtcggcacgccgtaagtgcaacga---ttgctgactgtatttcagac-ttcaacacgtaa
    GCA_034642005.1#JAWNYV010000013.1_1595757-1596200      atgggaaaggcttcgaggttgttagtgagt-aaatcca----tttgtcggcacgccgtaagtgcaacga---ttgctgactgtatttcagac-ttcaacacgtaa
    GCA_035773055.1#JAPMJO010000013.1_58458-58906          acgggaaaggcttcggggttgttagtatgt-aaatcca----tgtgtcggcacaccgtaggcacaacgaatgttgctaattgcattttagac-ttcaagacgtaa
    GCA_037126525.1#JAZDSW010000046.1_287452-287824        acgggaaaggcttcgaggttgtt----------------------------------------------------------------------------------
    GCA_038502765.1#JALBXV010000004.1_150133-150579        acgggaaaggcttcgaggttgttagtatgt-aactcca----tgtgtcggcacgccataagcacaacgaatgttgctaattacattttagac-ttcaacacgtaa
    GCA_038502765.1#JALBXV010000003.1_1183198-1183644      acgggaaaggcttcgaggttgttagtatgt-aactcca----tgtgtcggcacgccataagcacaacgaatgttgctaattgcattttagac-ttcaacacgtaa
    GCA_040938085.1#JBDQYN010000095.1_4849420-4849866      acgggaaaggcttcgtggttgttagtatgt-atatcca----tgtgccggcacgacgtaagcacaacgaaaattgctaattgcatttcagac-ttcaacacgtaa
    GCA_041380375.1#JAZEUI010000212.1_778435-778881        acgggaaaggcttcgtggttgttagtatgt-atatcca----tgtgccggcacgacgtaagcacaacgaaaattgctaattgcatttcagac-ttcaacacgtaa
    GCA_041380545.1#JAZEUM010000394.1_2965598-2966044      acgggaaaggcttcgtggttgttagtatgt-atatcca----tgtgccggcacgacgtaagcacaacgaaaattgctaattgcatttcagac-ttcaacacgtaa
    GCF_000271745.1#NW_022158520.1_5103546-5103992         acgggaaaggcttcgtggttgttagtatgt-atatcca----tgtgccggcacgacgtaagcacaacgaaaattgctaattgcatttcagac-ttcaacacgtaa
    GCF_020744475.1#NW_025763681.1_315043-315491           acgggaaaggcttcgaggttattagtatgt-aaatcca----tgtgtcggcacgccgtaagcacaacgaatgttgctaattgcattttagac-ttcaacacgtaa

    Selected Cols:                                                                                                                                                  

    Gaps Scores:                                                                                                                                                    
    Similarity Scores:
```
