# Supplementary material for: Starship giant transposons dominate plastic genomic regions in a fungal plant pathogen and drive virulence evolution
Source: Nat Commun. 2025 Jul 24;16:6806. doi: 10.1038/s41467-025-61986-6 (PMC12289983; doi:10.1038/s41467-025-61986-6)
Supplement: Supplementary file 6 — Source Data [file 41467_2025_61986_MOESM6_ESM.zip › Source Data/Fig6a_MSA.html]

trimAl v1.4 Summary


```
    Selected Sequences:   451 /Selected Residues:     936
    Deleted Sequences:      0 /Deleted Residues:       97

    Gaps Scores:                      =0=   <.001  <.050  <.100  <.150  <.200  <.250  <.350  <.500  <.750  <1.00   =1=  
    Similarity Scores:                =0=   <1e-6  <1e-5  <1e-4  <.001  <.010  <.100  <.250  <.500  <.750  <1.00   =1=  

                                            10        20        30        40        50        60        70        80        90       100       110       120
                                    =========+=========+=========+=========+=========+=========+=========+=========+=========+=========+=========+=========+
    12008_g358.t1                   -------------------------------------------------------MVSKIFST---------LASIALVAAGP---------VSLR-AV-VPHD--------SLNPVTQR
    12008_g4461.t1                  ---------------------------------------------------MQHTLLSTAALL---------GALSAVNASPA-------PILRRD----------------IITALP--
    12008_g7340.t1                  ------------------------------------------------MPSLRTASFSAVAAL---------LLLPAVIATPLPDTP-TTKLIRRD----------------LRQPLG--
    12008_g7652.t1                  ---------------------------------------------------MSPSLISIVTWL---------AAASSTLAAPL---------LESR-AV-INHD--------AVVGFPQT
    12008_g7834.t1                  ------------------------------------------------------------MLFSVG-------LLALAALPSS-----FGAVIQAR-QD-DPENPPRDPQPPPPGPIF--
    12008_g8465.t1                  ------------------------------------------------------------MLFLQN----IAVVTAMVLSVPS-----TASVMRRQ-NN-SSRILSESP---ALEPIVNG
    12008_g8848.t1                  ---------------------------------------------------MLPSAVFSVFAL---------VGIALAQQPPK-----------------VNHD--------SINPVRDT
    85S_g58.t1                      ------------------------------------------------MPSLRTASFSAVAAL---------LLLPAVIATPLPDTP-TTKLIRRD----------------LLQPLG--
    85S_g9168.t1                    ---------------------------------------------------MSPSLISIVTWL---------AAASSTLAAPL---------LESR-AV-INHD--------AVVGFPQT
    85S_g9863.t1                    ---------------------------------------------------MQHTLLSTAALL---------GALSAVNASPA-------PILRRD----------------IITALP--
    85S_g7040.t1                    ------------------------------------------------------------MLFLRN----IAVVTAMVLSVPS-----TASVMRRQ-NN-SSRILSESP---ALEPIVNG
    85S_g3154.t1                    -------------------------------------------------------MVSKIFST---------LASIALVAAGP---------VSLR-AV-VPHD--------SLNPVTQR
    85S_g4608.t1                    ------------------------------------------------------------MLFSVG-------LLALAALPSS-----FGAVIQAR-QD-DPENPPRDPQPPPPGPIF--
    85S_g4744.t1                    ---------------------------------------------------MLPSAVFSVFAL---------VGIALAQQPPK-----------------VNHD--------SINPVRDT
    CBS385.49_g66.t1                ------------------------------------------------------------MLFSVG-------LLALAALPSS-----FGAVIQAR-QD-DPENPP--------------
    CBS385.49_g189.t1               MK--------------SEARC----------VNQPVCLQKGGRWKA--KAAASPDMI-----------------KRLGSAAPK-----------------VNHD--------SINPVRDT
    CBS385.49_g400.t1               ---------------------------------------------------MSPSLISIVTWL---------AAASSTLAAPL---------LESR-AV-INHD--------AVVGFPQT
    CBS385.49_g400.t2               ---------------------------------------------------MSPSLISIVTWL---------AAASSTLAAPL---------LESR-AV-INHD--------AVVGFPQT
    CBS385.49_g1381.t1              MVV------------------------------------KVRELIFYNIPSLRTASFSA--------------------------------LIRRD----------------LLQPLG--
    CBS385.49_g5607.t1              -------------------------------------------------------MVSKIFST---------LASIALVAAGP---------VSLR-AV-VPHD--------SLNPVTQR
    CBS385.49_g7723.t1              ---------------------------------------------------MQHTLLSTAALL---------GALSAVNASPA-------PILRRD----------------IITALP--
    CBS385.49_g8721.t1              ------------------------------------------------------------MLFLRN----IAVVTAMVLSVPS-----TASVMRRQ-NN-SSRILSESP---ALEPIVNG
    CQ2_g254.t1                     ------------------------------------------------------------MLFLRN----IAVVTAMVLSVPS-----TASVMRRQ-NN-SSRILSESP---ALEPIVNG
    CQ2_g2332.t3                    M----------------------------------------AHASF--SPGLWQPALHRIVLL---------LVLYLALVHPS-----NCSVIQRR-AA-------------PPQKLP--
    CQ2_g2332.t4                    M----------------------------------------AHASF--SPGLWQPALHRIVLL---------LVLYLALVHPS-----NCSVIQRR-AA-------------PPQKLP--
    CQ2_g3243.t1                    ---------------------------------------------------MSPSLISIVTWL---------AAASSTLAAPL---------LESR-AV-INHD--------AVVGFPQT
    CQ2_g3454.t1                    ---------------------------------------------------MLPSAVFSVFAL---------VGSALAQQPPK-----------------VNHD--------SINPVRDT
    CQ2_g3584.t1                    ------------------------------------------------------------MLFSVG-------LLALAALPSS-----FGA-------------------PPPPGPIF--
    CQ2_g4742.t1                    ---------------------------------------------------MQHTLLSTAALL---------GALSAVNASPA-------PILRRD----------------IITALP--
    CQ2_g7512.t1                    -------------------------------------------------------MVSKIFST---------LASIALVAAGP---------VSLR-AV-VPHD--------SLNPVTQR
    CQ2_g8820.t1                    --------------------------------------------------------------------------MVVKSSQLLPDTP-TTKLIRRD----------------LLQPLG--
    DAR82592_g1639.t1               ------------------------------------------------------------MLFLRN----IAVVTAMVLSVPS-----TASVMRRQ-NN-SSRILSESP---ALEPIVNG
    DAR82592_g8837.t1               --------------------------------------------------------------------------MVVKSSQLLPDTP-TTKLIRRD----------------LLQPLG--
    DAR82592_g4435.t1               ---------------------------------------------------ML---------------------------PSA-----------------VNHD--------SINPVRDT
    DAR82592_g4647.t1               ---------------------------------------------------MSPSLISIVTWL---------AAASSTLAAPL---------LESR-AV-INHD--------AVVGFPQT
    DAR82592_g7268.t1               -------------------------------------------------------MVSKIFST---------LASIALVAAGP---------VSLR-AV-VPHD--------SLNPVTQR
    DAR82592_g4307.t1               ------------------------------------------------------------MLFSVG-------LLALAALPSS-----FGAVIQAR-QD-DPENPPRDPQPPPPGPIF--
    DAR82592_g3508.t1               M----------------------------------------AHASF--SPGLWQPALHRIVLL---------LVLYLALVHPS-----NCSVIQRR-AA-------------PPQKLP--
    DAR82592_g62.t1                 ---------------------------------------------------MQHTLLSTAALL---------GALSAVNASPA-------PILRRD----------------IITALP--
    DAR83138_g1199.t1               ------------------------------------------------------------MLFLRN----IAVVTAMVLSVPS-----TASVMRRQ-NN-SSRILSESP---ALEPIVNG
    DAR83138_g60.t1                 ---------------------------------------------------MQHTLLSTAALL---------GALSAVNASPA-------PILRRD----------------IITALP--
    DAR83138_g7641.t1               -------------------------------------------------------MVSKIFST---------LASIALVAAGP---------VSLR-AV-VPHD--------SLNPVTQR
    DAR83138_g9085.t1               --------------------------------------------------------------------------MVVKSSQLLPDTP-TTKLIRRD----------------LLQPLG--
    DAR83138_g3565.t3               M----------------------------------------AHASF--SPGLWQPALHRIVLL---------LVLYLALVHPS-----NCSVIQRR-AA-------------PPQKLP--
    DAR83138_g4173.t1               ------------------------------------------------------------MLFSVG-------LLALAALPSS-----FGAVIQAR-QD-DPENPPRDPQPPPPGPIF--
    DAR83138_g4314.t1               ---------------------------------------------------MLPSAVFSVFAL---------VGSALAQQPPK-----------------VNHD--------SINPVRDT
    DAR83138_g4535.t1               ---------------------------------------------------MSPSLISIVTWL---------AAASSTLAAPL---------LESR-AV-INHD--------AVVGFPQT
    DAR83143_g1427.t1               ------------------------------------------------------------MLFLRN----IAVVTAMVLSVPS-----TASVMRRQ-NN-SSRILSESP---ALEPIVNG
    DAR83143_g4194.t1               ------------------------------------------------------------MLFSVG-------LLALAALPSS-----FGAVIQAR-QD-DPENPPRDPQPPPPGPIF--
    DAR83143_g4335.t1               ---------------------------------------------------MLPSAVFSVFAL---------VGSALAQQPPK-----------------VNHD--------SINPVRDT
    DAR83143_g4545.t1               ---------------------------------------------------MSPSLISIVTWL---------AAASSTLAAPL---------LESR-AV-INHD--------AVVGFPQT
    DAR83143_g7287.t1               -------------------------------------------------------MVSKIFST---------LASIALVAAGP---------VSLR-AV-VPHD--------SLNPVTQR
    DAR83143_g3521.t1               M----------------------------------------AHASF--SPGLWQPALHRIVLL---------LVLYLALVHPS-----NCSVIQRR-AA-------------PPQKLP--
    DAR83143_g8715.t1               --------------------------------------------------------------------------M---SSQLLPDTP-TTKLIRRD----------------LLQPLG--
    DAR83143_g61.t1                 ---------------------------------------------------MQHTLLSTAALL---------GALSAVNASPA-------PILRRD----------------IITALP--
    DAR83175_g3551.t1               M----------------------------------------AHASF--SPGLWQPALHRIVLL---------LVLYLALVHPS-----NCSVIQRR-AA-------------PPQKLP--
    DAR83175_g4514.t1               ---------------------------------------------------MSPSLISIVTWL---------AAASSTLAAPL---------LESR-AV-INHD--------AVVGFPQT
    DAR83175_g4731.t1               ---------------------------------------------------MLPSAVFSVFAL---------VGSALAQQPPK-----------------VNHD--------SINPVRDT
    DAR83175_g7439.t1               -------------------------------------------------------MVSKIFST---------LASIALVAAGP---------VSLR-AV-VPHD--------SLNPVTQR
    DAR83175_g2075.t1               ------------------------------------------------------------MLFLRN----IAVVTAMVLSVPS-----TASVMRRQ-NN-SSRILSESP---ALEPIVNG
    DAR83175_g5817.t1               -------------------------------------------------------------MF-VGSQGNMCRTLALAALPSS-----FGAVIQAR-QD-DPENPPRDPQPPPPGPIF--
    DAR83175_g61.t1                 ---------------------------------------------------MQHTLLSTAALL---------GALSAVNASPA-------PILRRD----------------IITALP--
    DAR83175_g8890.t1               --------------------------------------------------------------------------M---SSQLLPDTP-TTKLIRRD----------------LLQPLG--
    DK045_g927.t1                   ---------------------------------------------------MSPSLISIVTWL---------AAASSTLAAPL---------LESR-AV-INHD--------AVVGFPQT
    DK045_g1138.t1                  ---------------------------------------------------MLPSAVFSVFAL---------VGIALAQQPPK-----------------VNHD--------SINPVRDT
    DK045_g1272.t1                  ------------------------------------------------------------MLFSVG-------LLALAALPSS-----FGAVIQAR-QD-DPENPPRDPQPPPPGPIF--
    DK045_g2451.t1                  ---------------------------------------------------MQHTLLSTAALL---------GALSAVNASPA-------PILRRD----------------IITALP--
    DK045_g3667.t1                  ------------------------------------------------------------MLFLRN----IAVVTAMVLSVPS-----TASVMRRQ-NN-SSRILSESP---ALEPIVNG
    DK045_g5985.t1                  --------------------------------------------------------------------------M---SSQLLPDTP-TTKLIRRD----------------LLQPLG--
    DK045_g9032.t1                  -------------------------------------------------------MVSKIFST---------LASIALVAAGP---------VSLR-AV-VPHD--------SLNPVTQR
    GF1192_g8987.t1                 ---------------------------------------------------MLPSTVFSVFAL---------VGSALAQQPPK-----------------VNHD--------SINPVRDT
    GF1192_g9126.t1                 ------------------------------------------------------------MLFSVG-------LLALAALPSS-----FGAVIQAR-QD-DPENPPRDPQPPPPGPIF--
    GF1192_g257.t1                  ------------------------------------------------------------MLFLRN----IAVVTAMVLSVPS-----TASVMRRQ-NN-SSRILSESP---ALEPIVNG
    GF1192_g1728.t1                 -------------------------------------------------------MVSKIFST---------LASIALVAAGP---------VSLR-AV-VPHD--------SLNPVTQR
    GF1192_g2840.t1                 ---------------------------------------------------MQHTLLSTAALL---------GALSAVNASPA-------PILRRD----------------IITALP--
    GF1192_g5025.t1                 ---------------------------------------------------MSPSLISIVTWL---------AAASSTLAAPL---------LESR-AV-INHD--------AVVGFPQT
    GF1192_g6069.t1                 --------------------------------------------------------------------------M---SSQLLPDTP-TTKLIRRD----------------LLQPLG--
    GF1207_g2093.t1                 ------------------------------------------------------------MLFLRN----IAVVTAMVLSVPS-----TASVMRRQ-NN-SSRILSESP---ALEPIVNG
    GF1207_g4890.t1                 ---------------------------------------------------MQHTLLSTAALL---------GALSAVNASPA-------PILRRD----------------IITALP--
    GF1207_g5214.t1                 MVV------------------------------------KVRELIFYNIPSLRTASFSA--------------------------------LIRRD----------------LLQPLG--
    GF1207_g6669.t1                 ---------------------------------------------------MSPSLISIVTWL---------AAASSTLAAPL---------LESR-AV-INHD--------AVVGFPQT
    GF1207_g6876.t1                 ---------------------------------------------------ML---------------------------PPQ-----------------SSR----------INPVRDT
    GF1207_g6999.t1                 ------------------------------------------------------------MLFSVG-------LLALAALPSS-----FGAVIQAR-QD-DPENPPRDPQPPPPGPIF--
    GF1207_g7831.t1                 -------------------------------------------------------MVSKIFST---------LASIALVAAGP---------VSLR-AV-VPHD--------SLNPVTQR
    GF1300_g9576.t1                 ------------------------------------------------------------MLFSVG-------LLALAALPSS-----FGAVIQAR-QD-DPENPPRDPQPPPPGPIF--
    GF1300_g9714.t1                 ---------------------------------------------------MLPSTVFSVFAL---------VGSALAQQPPK-----------------VNHD--------SINPVRDT
    GF1300_g259.t1                  ------------------------------------------------------------MLFLRN----IAVVTAMVLSVPS-----TASVMRRQ-NN-SSRILSESP---ALEPIVNG
    GF1300_g1512.t1                 ---------------------------------------------------MQHTLLSTAALL---------GALSAVNASPA-------PILRRD----------------IITALP--
    GF1300_g3582.t1                 --------------------------------------------------------------------------M---SSQLLPDTP-TTKLIRRD----------------LLQPLG--
    GF1300_g3720.t1                 ---------------------------------------------------MSPSLISIVTWL---------AAASSTLAAPL---------LESR-AV-INHD--------AVVGFPQT
    GF1300_g6171.t1                 -------------------------------------------------------MVSKIFST---------LASIALVAAGP---------VSLR-AV-VPHD--------SLNPVTQR
    Gf-Ca2_g111.t1                  ---------------------------------------------------MQHTLLSTAALL---------GALSAVNASPA-------PILRRD----------------IITALP--
    Gf-Ca2_g2398.t1                 ------------------------------------------------------------MLFLRN----IAVVTAMVLSVPS-----TASVMRRQ-NN-SSRILSESP---ALEPIVNG
    Gf-Ca2_g3362.t1                 ------------------------------------------------------------MLFSVG-------LLALAALPSS-----FGAVIQAR-QD-DPENPPRDPQPPPPGPIF--
    Gf-Ca2_g3481.t1                 ---------------------------------------------------ML---------------------------PPQ-----------------SSRSLP------SINPVRDT
    Gf-Ca2_g3687.t1                 ---------------------------------------------------MSPSLISIVTWL---------AAASSTLAAPL---------LESR-AV-INHD--------AVVGFPQT
    Gf-Ca2_g4661.t1                 --------------------------------------------------------------------------MVVKSSQLLPDTP-TTKLIRRD----------------LLQPLG--
    Gf-Ca2_g8346.t1                 -------------------------------------------------------MVSKIFST---------LASIALVAAGP---------VSLR-AV-VPHD--------SLNPVTQR
    Gf-Cb5_g260.t1                  ------------------------------------------------------------MLFLRN----IAVVTAMVLSVPS-----TASVMRRQ-NN-SSRILSESP---ALEPIVNG
    Gf-Cb5_g3949.t1                 ---------------------------------------------------MLPSTVFSVFAL---------VGSALAQQPPK-----------------VNHD--------SINPVRDT
    Gf-Cb5_g4084.t1                 ------------------------------------------------------------MLFSVG-------LLALAALPSS-----FGAVIQAR-QD-DPENPPRDPQPPPPGPIF--
    Gf-Cb5_g4605.t1                 -------------------------------------------------------MVSKIFST---------LASIALVAAGP---------VSLR-AV-VPHD--------SLNPVTQR
    Gf-Cb5_g6249.t1                 M----------------------------------------AHASF--SPSLWQPALHRIVLL---------LVLYLALVHPS-----NCSVIQRR-AA-------------PPQKLP--
    Gf-Cb5_g7167.t1                 ---------------------------------------------------MQHTLLSTAALL---------GALSAVNASPA-------PILRRD----------------IITALP--
    Gf-Cb5_g7934.t1                 ---------------------------------------------------MSPSLISIVTWL---------AAASSTLAAPL---------LESR-AV-INHD--------AVVGFPQT
    Gf-Cb5_g8589.t1                 --------------------------------------------------------------------------M---SSQLLPDTP-TTKLIRRD----------------LLQPLG--
    HoMCF_g1490.t1                  ------------------------------------------------MPSLRTASFSAVAAL---------LLLPAVIATPLPDTP-TTKLIRRD----------------LRQP----
    HoMCF_g2875.t1                  ----------------------------------------------------------------------------------------------------------------------MR
    HoMCF_g3274.t1                  ---------------------------------------------------MLPSAVFSVFAL---------VGIALAQQPP--------------------------------------
    HoMCF_g4303.t1                  ------------------------------------------------------------MLFLQN----IAVVTAMVLSVPS-----TASVMRRQ-NN-SSRILSESP---ALEPIVNG
    HoMCF_g5967.t1                  ------------------------------------------------------------MLFSVG-------LLALAALPSS-----FGAVIQAR-QD-DPENPPRDPQPPPPGPIF--
    HoMCF_g6093.t1                  ---------------------------------------------------MLPSAVFSVFAL---------VGIALAQQPP--------------------------------------
    HoMCF_g6096.t1                  ---------------------------------------------------MLPSAVFSVFAL---------VGIALAQQPP--------------------------------------
    HoMCF_g6549.t1                  ---------------------------------------------------MSPSLISIVTWL---------AAASSTLAAPL---------LESR-AV-INHD--------AVVGFPQT
    HoMCF_g10173.t1                 ---------------------------------------------------MQHTLLSTAALL---------GALSAVNASPA-------PILRRD----------------IITALP--
    HoMCLT_g1190.t1                 ------------------------------------------------------------MLFLRN----IAVVTAMVLSVPS-----TASVMRRQ-NN-SSRILSESP---ALEPIVNG
    HoMCLT_g2195.t1                 -------------------------------------------------------MVSKIFST---------LASIALVAAGP---------VSLR-AV-VPHD--------SLNPVTQR
    HoMCLT_g2713.t1                 --------------------------------------------------------------------------MVVKSSQLLPDTP-TTKLIRRD----------------LLQPLG--
    HoMCLT_g3698.t1                 ---------------------------------------------------MSPSLISIVTWL---------AAASSTLAAPL---------LESR-AV-INHD--------AVVGFPQT
    HoMCLT_g4981.t1                 ---------------------------------------------------MQHTLLSTAALL---------GALSAVNASPA-------PILRRD----------------IITALP--
    HoMCLT_g6335.t1                 ---------------------------------------------------MLPSTVFSVFAL---------VGSALAQQPPK-----------------VNHD--------SINPVRDT
    HoMCLT_g6471.t1                 ------------------------------------------------------------MLFSVG-------LLALAALPSS-----FGAVIQAR-QD-DPENPPRDPQPPPPGPIF--
    I1V_g7945.t1                    -------------------------------------------------------MVSKIFST---------LASIALVAAGP---------VSLR-AV-VPHD--------SLNPVTQR
    I1V_g9550.t1                    ---------------------------------------------------MLPSAVF--------------------------------------------------------------
    I1V_g9695.t1                    ------------------------------------------------------------MLFLRN----IAVVTAMVLSVPS-----TASVMRRQ-NN-SSRILSESP---ALEPIVNG
    I1V_g10783.t1                   ---------------------------------------------------MSPSLISIVTWL---------AAASSTLAAPL---------LESR-AV-VNHD--------AVVGFPQT
    I1V_g12440.t1                   ------------------------------------------------------------------------------------------------------------------------
    I1V_g13776.t1                   -----------------------------------------------------------------------------------------------------------------MVPLG--
    MPI-CAGE_g5591.t1               ---------------------------------------------------MQHTLLSTAALL---------GALSAVNASPA-------PILRRD----------------IITALP--
    MPI-CAGE_g8268.t1               ---------------------------------------------------MLPSAVFSVFAL---------VGIALAQQPPK-----------------VNHD--------SINPVRDT
    MPI-CAGE_g8414.t1               ------------------------------------------------------------MLFSVG-------LLALAALPSS-----FGAVIQAR-QD-DPENPPRDPQPPPPGPIF--
    MPI-CAGE_g9956.t1               ---------------------------------------------------MSPSLISIVTWL---------AAASSTLAAPL---------LESR-AV-INHD--------AVVGFPQT
    MPI-CAGE_g1577.t1               ------------------------------------------------------------MLFLQN----IAVVTAMVLSVPS-----TASVMRRQ-NN-SSRILSESP---ALEPIVNG
    MPI-CAGE_g2204.t1               M----------------------------------------AHASF--SPSLWQPALHRIVLL---------LVLYLALVHPS-----NCSVIQRR-AA-------------PPQKLP--
    MPI-CAGE_g10572.t1              ------------------------------------------------------------MLFSVG-------LLALAALPSS-----FGAVIQAR-QD-DPENPPRDPQPPPPGPIF--
    MPI-CAGE_g3967.t1               ------------------------------------------------MPSLRTASFSAVAAL---------LLLPAVIATPLPDTP-TTKLIRRD----------------LRQPLG--
    MPI-CAGE_g4142.t1               -------------------------------------------------------MVSKIFST---------LASIALVAAGP---------VSLR-AV-VPHD--------SLNPVTQR
    S011_g7486.t1                   M----------------------------------------AHASF--SPGLWQPALHRIVLL---------LVLYLALVHPS-----NCSVIQRR-AA-------------PPQKLP--
    S011_g8092.t1                   -------------------------------------------------------MVSKIFST---------LASIALVAAGP---------VSLR-AV-VPHD--------SLNPVTQR
    S011_g8657.t1                   ------------------------------------------------------------MLFSVG-------LLALAALPSS-----FGAVIQAR-QD-DPENPPRDPQPPPPGPIF--
    S011_g8986.t1                   ---------------------------------------------------MSPSLISIVTWL---------AAASSTLAAPL---------LESR-AV-INHD--------AVVGFPQT
    S011_g9203.t1                   ---------------------------------------------------MLPSAVFSVFAL---------VGSALAQQPPK-----------------VNHD--------SINPVRDT
    S011_g1207.t1                   ------------------------------------------------------------MLFLRN----IAVVTAMVLSVPS-----TASVMRRQ-NN-SSRILSESP---ALEPIVNG
    S011_g2838.t1                   ---------------------------------------------------MQHTLLSTAALL---------GALSAVNASPA-------PILRRD----------------IITALP--
    S011_g5817.t1                   --------------------------------------------------------------------------M---SSQLLPDTP-TTKLIRSD----------------LLQPLG--
    S023_g1908.t1                   ------------------------------------------------------------MLFLQN----IAVVTAMVLSVPS-----TASVMRRQ-NN-SSRILSESP---ALEPIVNG
    S023_g3148.t1                   ---------------------------------------------------MSPSLISIVTWL---------AAASSTLAAPL---------LESR-AV-INHD--------AVVGFPQT
    S023_g4559.t1                   ---------------------------------------------------MQHTLLSTAALL---------GALSAVNASPA-------PILRRD----------------IITALP--
    S023_g5760.t1                   ------------------------------------------------------------MLFSVG-------LLALAALPSS-----FGAVIQAR-QD-DPENPPRDPQPPPPGPIF--
    S023_g5904.t1                   ---------------------------------------------------MLPSAVFSVFAL---------VGIALAQQPPK-----------------VNHD--------SINPVRDT
    S023_g7666.t1                   ------------------------------------------------MPSLRTASFSAVAAL---------LLLPAVIATPLPDTP-TTKLIRRD----------------LRQPLG--
    S023_g9104.t1                   -------------------------------------------------------MVSKIFST---------LASIALVAAGP---------VSLR-AV-VPHD--------SLNPVTQR
    TO22_g2077.t1                   ------------------------------------------------------------MLFLRN----IAVVTAMVLSVPS-----TASVMRRQ-NN-SSRILSESP---ALEPIVNG
    TO22_g2405.t1                   ------------------------------------------------------------MLFSVG-------LLALAALPSS-----FGAVIQAR-QD-DPENPPRDPQPPPPGPIF--
    TO22_g2541.t1                   ---------------------------------------------------MLPSAVFSVFAL---------VGIALAQQPPK-----------------VNHD--------SINPVRDT
    TO22_g2749.t1                   ---------------------------------------------------MSPSLISIVTWL---------AAASSTLAAPL---------LESR-AV-INHD--------AVVGFPQT
    TO22_g3776.t1                   ---------------------------------------------------MQHTLLSTAALL---------GALSAVNASPA-------PILRRD----------------IITALP--
    TO22_g6379.t1                   -------------------------------------------------------MVSKIFST---------LASIALVAAGP---------VSLR-AV-VPHD--------SLNPVTQR
    TO22_g9457.t1                   --------------------------------------------------------------------------M---SSQLLPDTP-TTKLIRRD----------------LLQPLG--
    Ud1-4-1_g9889.t1                --------------------------------------------------------------------------MVVKSSQLLPDTP-TTKLIRRD----------------LLQPLG--
    Ud1-4-1_g261.t1                 ------------------------------------------------------------MLFLRN----IAVVTAMVLSVPS-----TASVMRRQ-NN-SSRILSESP---ALEPIVNG
    Ud1-4-1_g2536.t1                ---------------------------------------------------MQHTLLSTAALL---------GALSAVNASPA-------PILRRD----------------IITALP--
    Ud1-4-1_g2721.t1                ------------------------------------------------------------MLFSVG-------LLALAALPSS-----FGAVIQAR-QD-DPENPPRDPQPPPPGPIF--
    Ud1-4-1_g2858.t1                ---------------------------------------------------MLPSAVFSVFAL---------VGIALAQQPPK-----------------VNHD--------SINPVRDT
    Ud1-4-1_g3064.t1                ---------------------------------------------------MSPSLISIVTWL---------AAASSTLAAPL---------LESR-AV-INHD--------AVVGFPQT
    Ud1-4-1_g3975.t1                M----------------------------------------AHASF--SPSLWQPALHRIVLL---------LVLYLALVHPS-----NCSVIQRR-AA-------------PPQKLP--
    Ud1-4-1_g4565.t1                -------------------------------------------------------MVSKIFST---------LASIALVAAGP---------VSLR-AV-VPHD--------SLNPVTQR
    V13_g3278.t1                    ------------------------------------------------------------MLFSAP----------------------------GR-----PENPPRDPQPPPPGPIF--
    V13_g3397.t1                    ---------------------------------------------------MLPSAVFSVFAL---------VGIALAQQPP--------------------------------------
    V13_g4590.t1                    -------------------------------------------------------MVSKIFST---------LASIALVAAGP------------------------------------R
    V13_g5067.t1                    ---------------------------------------------------MQHTLLSTAALL---------GALSAVNASPA-------PILRRD----------------IITALP--
    V13_g6708.t1                    ------------------------------------------------MPSLRTASFSAVAAL---------LLLPAVIATPLPDTP-TTKLIRRD----------------LRQPLG--
    V13_g9344.t1                    M----------------------------------------AH-----------------------------------------------------------------------------
    V13_g9739.t1                    ---------------------------------------------------MSPSLISIVTWL---------AAASSTLAAPL---------LESR-AV-INHD--------AVVGFPQT
    V13_g10958.t1                   ------------------------------------------------------------MLFLQN----IAVVTAMVLSVPS-----TASVMRRQ-NN-SSRILSESP---ALEPIVNG
    Vd39_g2112.t1                   M----------------------------------------AHASF--SPGLWQPALHRIVLL---------LVLYLALVHPS-----NCSVIQRR-AA-------------PPQKLP--
    Vd39_g2112.t3                   M----------------------------------------AHASF--SPGLWQPALHRIVLL---------LVLYLALVHPS-----NCSVIQRR-AA-------------PPQKLP--
    Vd39_g2716.t1                   ------------------------------------------------------------MLFLRN----IAVVTAMVLSVPS-----TASVMRRQ-NN-SSRILSESP---ALEPIVNG
    Vd39_g3506.t1                   ------------------------------------------------------------MLFSVG-------LLALAALPSS-----FGAVIQAR-QD-DPENPPRDPQPPPPGPIF--
    Vd39_g3907.t1                   --------------------------------------------------------------------------MVVKSSQLLPDTP-TTKLIRSD----------------LLQPLG--
    Vd39_g4379.t1                   ---------------------------------------------------MQHTLLSTAALL---------GALSAVNASPA-------PILRRD----------------IITALP--
    Vd39_g7329.t1                   -------------------------------------------------------MVSKIFST---------LASIALVAAGP---------VSLR-AV-VPHD--------SLNPVTQR
    Vd39_g8964.t1                   ---------------------------------------------------MSPSLISIVTWL---------AAASSTLAAPL---------LESR-AV-INHD--------AVVGFPQT
    Vd39_g9298.t1                   ---------------------------------------------------MLPSAVFSVFAL---------VGSALAQQPPK-----------------VNHD--------SINPVRDT
    Vd-653_g1291.t1                 ---------------------------------------------------MLPSAVFSVFAL---------VGIALAQQPP--------------------------------------
    Vd-653_g2557.t1                 ------------------------------------------------------------MLFLRN----IAVVTAMVLSVPS-----TASVMRRQ-NN-SSRILSESP---ALEPIVNG
    Vd-653_g3339.t1                 ------------------------------------------------MPSLRTASFSAVAAL---------LLHAYHKAHPPRSPP-APRRL---------------------------
    Vd-653_g3685.t1                 ------------------------------------------------------------------------------------------------------------------------
    Vd-653_g3686.t1                 ---------------------------------------------------MSPSLISIVTWL---------AAASSTLAAPL---------LESR-AV-INHD--------AV------
    Vd-653_g5777.t1                 ------------------------------------------------------------MLFSVG-------LLALAALPSS-----FGAVIQAR-QD-DPENPP--------------
    Vd-653_g8550.t1                 -------------------------------------------------------MVSKIFST---------LASIALVAAGP---------VSLR-AV-VPHD--------SLNP----
    Vd-653_g9566.t1                 ---------------------------------------------------MQHTLLSTAALL---------GALSAVNASPA-------PILRRD----------------IITALP--
    VD991_g2070.t1                  ------------------------------------------------------------MLFLRN----IAVVTAMVLSVPS-----TASVMRRQ-NN-SSRILSESP---ALEPIVNG
    VD991_g2394.t1                  ------------------------------------------------------------MLFSVG-------LLALAALPSS-----FGAVIQAR-QD-DPENPPRDPQPPPPGPIF--
    VD991_g2531.t1                  ---------------------------------------------------MLPSAVFSVFAL---------VGSALAQQPPK-----------------VNHD--------SINPVRDT
    VD991_g2742.t1                  ---------------------------------------------------MSPSLISIVTWL---------AAASSTLAAPL---------LESR-AV-INHD--------AVVGFPQT
    VD991_g3653.t1                  M----------------------------------------AHASF--SPGLWQPALHRIVLL---------LVLYLALVHPS-----NCSVIQRR-AA-------------PPQKLP--
    VD991_g3653.t2                  M----------------------------------------AHASF--SPGLWQPALHRIVLL---------LVLYLALVHPS-----NCSVIQRR-AA-------------PPQKLP--
    VD991_g3719.t1                  ---------------------------------------------------MQHTLLSTAALL---------GALSAVNASPA-------PILRRD----------------IITALP--
    VD991_g7479.t1                  -------------------------------------------------------MVSKIFST---------LASIALVAAGP---------VSLR-AV-VPHD--------SLNPVTQR
    VD991_g8969.t1                  --------------------------------------------------------------------------MVVKSSQLLPDTP-TTKLIRRD----------------LLQPLG--
    VdB09_g1055.t1                  ------------------------------------------------------------MLFSVG-------LLALAALPSS-----FGAVIQAR-QD-DPENPPRDPQPPPPGPIF--
    VdB09_g1685.t1                  --------------------------------------------------------------------------M---SSQLLPDTP-TTKLIRRD----------------LLQPLG--
    VdB09_g1751.t1                  ---------------------------------------------------MQHTLLSTAALL---------GALSAVNASPA-------PILRRD----------------IITALP--
    VdB09_g3918.t1                  M----------------------------------------AHASF--SPSLWQPALHRIVLL---------LVLYLALVHPS-----NCSVIQRR-AA-------------PPQKLP--
    VdB09_g4877.t1                  ---------------------------------------------------MSPSLISIVTWL---------AAASSTLAAPL---------LESR-AV-INHD--------AVVGFPQT
    VdB09_g5096.t1                  ---------------------------------------------------MLPSAVFSVFAL-------------------------------------VNHD--------SINPVRDT
    VdB09_g9703.t1                  -------------------------------------------------------MVSKIFST---------LASIALVAAGP---------VSLR-AV-VPHD--------SLNPVTQR
    VdB09_g7661.t1                  ------------------------------------------------------------MLFLRN----IAVVTAMVLSVPS-----TASVMRRQ-NN-SSRILSESP---ALEPIVNG
    VdC07_g4126.t1                  ------------------------------------------------------------MLFSVG-------LLALAALPSS-----FGAVIQAR-QD-DPENPPRDPQPPPPGPIF--
    VdC07_g4261.t1                  ---------------------------------------------------MLPSAVFSVFAL---------VGIALAQQPPK-----------------VNHD--------SINPVRDT
    VdC07_g4482.t1                  ---------------------------------------------------MSPSLISIVTWL---------AAASSTLAAPL---------LESR-AV-INHD--------AVVGFPQT
    VdC07_g605.t1                   -------------------------------------------------------MVSKIFST---------LASIALVAAGP---------VSLR-AV-VPHD--------SLNPVTQR
    VdC07_g1872.t1                  --------------------------------------------------------------------------MVVKSSQLLPDTP-TTKLIRRD----------------LLQPLG--
    VdC07_g2986.t1                  ---------------------------------------------------MQHTLLSTAALL---------GALSAVNASPA-------PILRRD----------------IITALP--
    VdC07_g7672.t1                  ------------------------------------------------------------MLFLRN----IAVVTAMVLSVPS-----TASVMRRQ-NN-SSRILSESP---ALEPIVNG
    Vd-H5_g2477.t1                  ---------------------------------------------------MSPSLISIVTWL---------AAASSTLAAPL---------LESR-AV-INHD--------AVVGFPQT
    Vd-H5_g5176.t1                  ------------------------------------------------MPSLRTASFSAVAAL---------LLLPAVIATPLPDTP-TTKLIRRD----------------LLQPLG--
    Vd-H5_g6144.t1                  ---------------------------------------------------MQHTLLSTAALL---------GALSAVNASPA-------PILRRD----------------IITALP--
    Vd-H5_g7203.t1                  -------------------------------------------------------MVSKIFST---------LASIALVAAGP---------VSLR-AV-VPHD--------SLNPVTQR
    Vd-H5_g8423.t1                  ------------------------------------------------------------MLFSVG-------LLALAALPSS-----FGA-------------------PPPPGPIF--
    Vd-H5_g8546.t1                  ------------------------------------------------------------------------------MSPPK-----------------VNHD--------SINPVRDT
    Vd-H5_g9396.t1                  ------------------------------------------------------------MLFLRN----IAVVTAMVLSVPS-----TASVMRRQ-NN-SSRILSESP---ALEPIVNG
    VdLs16_g2071.t1                 ------------------------------------------------------------MLFLRN----IAVVTAMVLSVPS-----TASVMRRQ-NN-SSRILSESP---ALEPIVNG
    VdLs16_g7836.t1                 -------------------------------------------------------MVSKIFST---------LASIALVAAGP---------VSLR-AV-VPHD--------SLNPVTQR
    VdLs16_g8455.t1                 ---------------------------------------------------MQHTLLSTAALL---------GALSAVNASPA-------PILRRD----------------IITALP--
    VdLs16_g5349.t1                 ---------------------------------------------------MSPSLISIVTWL---------AAASSTLAAPL---------LESR-AV-INHD--------AVVGFPQT
    VdLs16_g5560.t1                 ---------------------------------------------------MLPSAVFSVFAL---------VGIALAQQPPK-----------------VNHD--------SINPVRDT
    VdLs16_g5699.t1                 ------------------------------------------------------------MLFSVG-------LLALAALPSS-----FGAVIQAR-QD-DPENPPRDPQPPPPGPIF--
    VdLs16_g6952.t1                 --------------------------------------------------------------------------M---SSQLLPDTP-TTKLIRRD----------------LLQPLG--
    VdLs17_g64.t1                   ---------------------------------------------------MQHTLLSTAALL---------GALSAVNASPA-------PILRRD----------------IITALP--
    VdLs17_g1975.t1                 ------------------------------------------------------------MLFLQN----IAVVTAMVLSVPS-----TASVMRRQ-NN-SSRILSESP---ALEPIVNG
    VdLs17_g3499.t1                 M----------------------------------------AHASF--SPSLWQPALHRIVLL---------LVLYLALVHPS-----NCSVIQRR-AA-------------PPQKLP--
    VdLs17_g4253.t1                 -------------------------------------------------------MWT--------------------------------------------------------------
    VdLs17_g4391.t1                 ---------------------------------------------------MLPSAVFSVFAL---------VGIALAQQPPK-----------------VNHD--------SINPVRDT
    VdLs17_g5246.t1                 ------------------------------------------------------------MLFSVG-------LLALAALPSS-----FGAVIQAR-QD-DPENPPRDPQPPPPGPIF--
    VdLs17_g6770.t1                 -------------------------------------------------------MVSKIFST---------LASIALVAAGP---------VSLR-AV-VPHD--------SLNPVTQR
    VdLs17_g8273.t1                 ------------------------------------------------MPSLRTASFSAVAAL---------LLLPAVIATPLPDTP-TTKLIRRD----------------LRQPLG--
    VdLs17_g8809.t1                 ---------------------------------------------------MSPSLISIVTWL---------AAASSTLAAPL---------LESR-AV-INHD--------AVVGFPQT
    Vd-R1_g926.t1                   ---------------------------------------------------MSPSLISIVTWL---------AAASSTLAAPL---------LESR-AV-INHD--------AV------
    Vd-R1_g3679.t1                  ---------------------------------------------------MQHTLLSTAALL---------GALSAVNASPA-------PILRRD----------------IITALP--
    Vd-R1_g4718.t1                  -------------------------------------------------------MVSKIFST---------LASIALVAAGP---------VSLR-AV-VPHD--------SLNPVTQR
    Vd-R1_g5037.t1                  ------------------------------------------------MPSLRTASFSAVAAL---------LLLPAVIATPLPDTP-TTKLIRRD----------------LLQPLG--
    Vd-R1_g8886.t1                  ------------------------------------------------------------MLFLRN----IAVVTAMVLSVPS-----TASVMRRQ-NN-SSRILSESP---ALEPIVNG
    Vd-R1_g9491.t1                  ---------------------------------------------------MLPSAVFSVFAL---------VGIALAQQPP--------------------------------------
    Vd-R1_g9617.t1                  ------------------------------------------------------------MLFSVG-------LLALAALPSS-----FGAVIQAR-QD-DPENPPRDPQPPPPGPIF--
    VT-2A_g1161.t1                  ---------------------------------------------------MQHTLLSTAALL---------GALSAVNASPA-------PILRRD----------------IITALP--
    VT-2A_g1267.t1                  --------------------------------------------------------------------------MVVKSSQLLPDTP-TTKLIRRD----------------LLQPLG--
    VT-2A_g4726.t1                  -------------------------------------------------------MVSKIFST---------LASIALVAAGP---------VSLR-AV-VPHD--------SLNPVTQR
    VT-2A_g6128.t1                  ---------------------------------------------------MSPSLISIVTWL---------AAASSTLAAPL---------LESR-AV-INHD--------AVVGFPQT
    VT-2A_g6128.t2                  ---------------------------------------------------MSPSLISIVTWL---------AAASSTLAAPL---------LESR-AV-INHD--------AVVGFPQT
    VT-2A_g6341.t1                  ---------------------------------------------------MLPST-------------------RLGSAAPK-----------------VNHD--------SINPVRDT
    VT-2A_g6470.t1                  ------------------------------------------------------------MLFSVG-------LLALAALPSS-----FGAVIQAR-QD-DPENPPRDPQPPPPGPIF--
    VT-2A_g9510.t1                  ------------------------------------------------------------------------------------------------------------------------
    VT-2A_g9511.t1                  ------------------------------------------------------------MLFLRN----IAVVTAMVLSVPS-----TASVMRRQ-NN-SSRILSESP---ALEPIVNG
    XJ511_g1085.t1                  ------------------------------------------------------------MLFLQN----IAVVTAMVLSVPS-----TASVMRRQ-NN-SSRILSESP---ALEPIVNG
    XJ511_g1518.t1                  ---------------------------------------------------MQHTLLSTAALL---------GALSAVNASPA-------PILRRD----------------IITALP--
    XJ511_g4592.t1                  ------------------------------------------------MPSLRTASFSAVAAL---------LLLPAVIATPLPDTP-TTKLIRRD----------------LRQPLG--
    XJ511_g5794.t1                  ------------------------------------------------------------MLFSVG-------LLALAALPSS-----FGAVIQAR-QD-DPENPPRDPQPPPPGPIF--
    XJ511_g5932.t1                  ---------------------------------------------------MLPSAVFSVFAL---------VGIALAQQPPK-----------------VNHD--------SINPVRDT
    XJ511_g7291.t1                  -------------------------------------------------------MVSKIFST---------LASIALVAAGP---------VSLR-AV-VPHD--------SLNPVTQR
    XJ511_g8237.t1                  ---------------------------------------------------MSPSLISIVTWL---------AAASSTLAAPL---------LESR-AV-INHD--------AVVGFPQT
    XJ592_g261.t1                   ------------------------------------------------------------MLFLRN----IAVVTAMVLSVPS-----TASVMRRQ-NN-SSRILSESP---ALEPIVNG
    XJ592_g3433.t1                  ---------------------------------------------------MQHTLLSTAALL---------GALSAVNASPA-------PILRRD----------------IITALP--
    XJ592_g5005.t1                  -------------------------------------------------------MVSKIFST---------LASIALVAAGP---------VSLR-AV-VPHD--------SLNPVTQR
    XJ592_g6487.t1                  --------------------------------------------------------------------------M---SSQLLPDTP-TTKLIRRD----------------LLQPLG--
    XJ592_g7860.t1                  ---------------------------------------------------MSPSLISIVTWL---------AAASSTLAAPL---------LESR-AV-INHD--------AVVGFPQT
    XJ592_g8068.t1                  ---------------------------------------------------MLPSAVFSVFAL---------VGSALAQQPPK-----------------VNHD--------SINPVRDT
    XJ592_g8206.t1                  ------------------------------------------------------------MLFSVG-------LLALAALPSS-----FGAVIQAR-QD-DPENPPRDPQPPPPGPIF--
    XJ592_g9637.t1                  M----------------------------------------AHASF--SPGLWQPALHRIVLL---------LVLYLALVHPS-----NCSVIQRR-AA-------------PPQKLP--
    XJ592_g9637.t2                  M----------------------------------------AHASF--SPGLWQPALHRIVLL---------LVLYLALVHPS-----NCSVIQRR-AA-------------PPQKLP--
    397_g2445.t1                    ---------------------------------------------------MHPSKLAAIVAF---------FATGS-IAAPAADTPEGFSAIERR-AVEVAHS--------SLNALPQN
    397_g4853.t1                    -------------------------------------------------------MVSKIFAT---------LASIAMVAAGP---------ISLR-AV-VAHD--------SLNPVEQR
    397_g6363.t1                    ------------------------------------------------MPSLK--SISAAVAL---------LLLPMALTSPLPDPS-SVELSRRD----------------LLQPLG--
    397_g7397.t1                    ---------------------------------------------------MQHTILTTAALL---------SVLSAVNASPA-------PITRRD----------------ILTALP--
    397_g7755.t1                    ------------------------------------------------------------MPSLQK----VAAAAALVLSLPS-----FALVIQRQENN-ATRSLSNSD---TLKPIVDG
    397_g8350.t1                    ---------------------------------------------------MSPSLISIVTWL---------AAASSTLAAPL---------LESR-AV-IAHD--------AVVGFSQA
    397_g9733.t1                    ------------------------------------------------------------MLFSIG-------LLALAALPSS-----LGAAIQAR-QD-DPENPPRDPQPPPPGPIF--
    CBS382.66_g255.t1               -------------------------------------------------------MLYSKMLTLRN----IAVVTAMVLSVPS-----TASAMRRQ-NN-SSRILSESQ---ALEPIVDG
    CBS382.66_g255.t2               ------------------------------------------------------------MLTLRN----IAVVTAMVLSVPS-----TASAMRRQ-NN-SSRILSESQ---ALEPIVDG
    CBS382.66_g4327.t1              ---------------------------------------------------MSPSLISIVTWL---------AAASSTLAAPL---------LESR-AV-IGHD--------AVVGFPQT
    CBS382.66_g5334.t1              M----------------------------------------AHAIF--SPGLWQPALHRVVLL---------LVLYLALVHPS-----NSSVIQRR-AA-------------PPQKLP--
    CBS382.66_g5334.t2              ------------------------------------------------------------------------------------------------------------------------
    CBS382.66_g5795.t1              -------------------------------------------------------MVSKIFST---------LASIALVAAGP---------ISLR-AV-VPHD--------SLNPVTQR
    CBS382.66_g6578.t1              ---------------------------------------------------MQHTFFSTAALL---------GALSAVNASPA-------PILRRD----------------IITALP--
    CBS382.66_g8384.t1              ------------------------------------------------MPSLRTASFSAVAAL---------LLLPAVIATPLPDTP-TTKLIRRD----------------LLQPLG--
    CBS382.66_g9605.t1              ---------------------------------------------------MLPSTIFSVFAL---------VGSALAQQPPK-----------------VNHD--------SINPIRDT
    CBS382.66_g9740.t1              ------------------------------------------------------------MLFSVG-------LLALAAFPSS-----FGAVIQAR-QD-DPENPPRDPQPPPPGPIF--
    MUCL9792_g2463.t1               ---------------------------------------------------MLPSTILSVLAF---------AGSTLAQWGPP--------------GT-VNHD--------SLSPVSDT
    MUCL9792_g2802.t1               ---------------------------------------------------MHPSKIVALVAF---------FATGS-VAAPAADTPEGFSNIERR-AVEVPHS--------SLNALPQN
    MUCL9792_g3157.t1               ------------------------------------------------MPSLNMLAAIAVTSL---------NILPFALANPLS-------LERRE----------------LLTRLP--
    MUCL9792_g4589.t1               M----------------------------------------SQPDP--H-GLRITAMRRIVLF---------LLFFLNLVSPT-----HASVIQRR-AD-------------PPRKLP--
    MUCL9792_g4589.t2               ------------------------------------------------------------------------------------------------------------------------
    MUCL9792_g4589.t3               M----------------------------------------SQPDP--H-GLRITAMRRIVLF---------LLFFLNLVSPT-----HASVIQRR-AD-------------PPRKLP--
    MUCL9792_g6332.t1               ---------------------------------------------------MQNTLLTTAALL---------GALSAVHASPA-------PILRRD----------------ILTPLP--
    MUCL9792_g7308.t1               ---------------------------------------------------MSPSLISIVTWL---------AAASSTLAAPL---------LESR-AV-IAHD--------AVVGFPQT
    MUCL9792_g10611.t1              -------------------------------------------------------MVSKIMST---------LASIALVAASP---------LSLR-AV-VPHD--------SLNPVEQR
    PD401_g2361.t1                  ---------------------------------------------------MHPSKIVALVAY---------FATGS-IAAPAAGTPEGFSNIERR-AVQVPHS--------SLNALPQN
    PD401_g3373.t1                  ---------------------------------------------------MSPSLISIVTWL---------AAASSTLAAPL---------LESR-AV-IAHD--------AVVGFPQT
    PD401_g4730.t1                  ---------------------------------------------------MQNTLLTTAALL---------GALSAVHASPA-------PILKRD----------------ILTALP--
    PD401_g7339.t1                  -------------------------------------------------------MVSKIMST---------LASIALVAASP---------LSLR-AV-VAHD--------SLNPIDQR
    PD401_g10483.t1                 ---------------------------------------------------MLPSTILSVLAF---------AGSTLAQWGPP--------------GT-VNHD--------SLSPVSDT
    PD593_g749.t1                   M----------------------------------------SQPDP--H-GLRITAMRRIVLF---------LLFFLNLVSPT-----HASVIQRR-AD-------------PPRKLP--
    PD593_g2541.t1                  ---------------------------------------------------MHPSKIVALVAF---------FAKGS-VAAPAADTPEGFSNIERR-AVEVPHS--------SLNALPQN
    PD593_g2728.t1                  ---------------------------------------------------MQNTLLTTAALL---------GALSAVHASPA-------PILRRD----------------ILTPLP--
    PD593_g7030.t1                  ---------------------------------------------------MSPSLISIVTWL---------AAASSTLAAPL---------LESR-AV-IAHD--------AVVGFPQT
    PD593_g8432.t1                  -------------------------------------------------------MVSKIMST---------LASIALVAASP---------LSLR-AV-VPHD--------SLNPVEQR
    PD593_g9596.t1                  ---------------------------------------------------MLPSTILSVLAF---------AGSTLAQWGPP--------------GT-VNHD--------SLSPVSDT
    PD618_g114.t1                   ---------------------------------------------------MHPSKIVALVAY---------FATGS-IAAPAAGTPEGFSNIERR-AVEVPHS--------SLNALPQN
    PD618_g3974.t1                  ---------------------------------------------------MQNTLLTTAALL---------GALSAVHASPA-------PILKRD----------------ILTALP--
    PD618_g5755.t1                  ---------------------------------------------------MSPSLISIVTWL---------AAASSTLAAPL---------LESR-AV-IAHD--------AVVGFPQT
    PD618_g9213.t1                  -------------------------------------------------------MVSKIMST---------LASIALVAASP---------LSLR-AV-VAHD--------SLNPIDQR
    PD618_g9942.t1                  ---------------------------------------------------MLPSAILSVLAF---------AGSTLAQWGPP--------------GT-VNHD--------SLSPIPDT
    PD659_g6753.t1                  ---------------------------------------------------MHPSKIVALVAY---------FATGS-IAAPAAGTPEGFSNIERR-AVQVPHS--------SLNALPQN
    PD659_g7370.t1                  -------------------------------------------------------MVSKIMST---------LASIALVAASP---------LSLR-AV-VAHD--------SLNPIDQR
    PD659_g10785.t1                 ---------------------------------------------------MLPSTILSVLAF---------AGSTLAQWGPP--------------GT-VNHD--------SLSPVSDT
    PD659_g2262.t1                  ---------------------------------------------------MQNTLLTTAALL---------GALSAVHASPA-------PILKRD----------------ILTALP--
    PD659_g5819.t1                  ---------------------------------------------------MSPSLISIVTWL---------AAASSTLAAPL---------LESR-AV-IAHD--------AVVGFPQT
    PD660_g1229.t1                  ---------------------------------------------------MSPSLISIVTWL---------AAASSTLAAPL---------LESR-AV-IAHD--------AVVGFPQT
    PD660_g3973.t1                  ---------------------------------------------------MQNTLLTTAALL---------GALSAVHASPA-------PILKRD----------------ILTALP--
    PD660_g6305.t1                  ---------------------------------------------------MLPSTILSVLAF---------AGSTLAQWGPP--------------GT-VNHD--------SLSPIPDT
    PD660_g7826.t1                  -------------------------------------------------------MVSKIMST---------LASIALVAASP---------LGLR-AV-VAHD--------SLNPIDQR
    PD660_g9669.t1                  ---------------------------------------------------MHPSKIVALAAY---------FATGS-IAAPAAGTPEGFSNIERR-AVEVPHS--------SLNALPQN
    PD660_g10032.t1                 ------------------------------------------------MPSLNILAVIAVTSL---------TILPFALANPLP-------LERRE----------------LLTRLP--
    PD670_g4670.t1                  ---------------------------------------------------MQNTLLTTAALL---------GALSAVNASPA-------PIMRRD----------------ILTALP--
    PD670_g5219.t1                  ---------------------------------------------------MSPSLISIVTWL---------AAVSSTLAAPL---------LEAR-AV-IAHD--------AVVGFSQT
    PD670_g7258.t1                  -------------------------------------------------------MVSKIMTT---------LASIALAVASP---------LNLR-AV-VAHD--------SLNPIDQR
    PD670_g8696.t1                  ------------------------------------------------------------MLFPMS-------LLALSSLPLS-----WGLAIQAR-QD-DPLEPPEIPSGPPPGPIF--
    PD670_g10480.t1                 ---------------------------------------------------MGLARVLSLLVYF-------SALLVATVASPV-DVLEQSHELSRR-GT-IPHN--------QVKGFSQA
    PD670_g10529.t1                 ---------------------------------------------------MLPSTILSVLAF---------AGSAIAQWGPP--------------GT-VNHD--------SLNPVRDT
    PD670_g11082.t1                 ---------------------------------------------------MHASKIAAVLAF---------FASSS-IAAPAADALDGFSRIERR-AAEVPHS--------SLNALGQN
    PD670_g2580.t1                  ------------------------------------------------MPSFATLSATAVALL---------YLLPFTLANPTP-------LERRD----------------LLQPLG--
    PD670_g3093.t1                  M----------------------------------------SQPDP--H-GLRITAMRRIVLF---------LLFYLTLVSPT-----HASVIQRR-AD-------------PPQKLP--
    PD683_g248.t1                   ----------------------------------------------------------------------------MVLSAPS-----TASAMRRQ-SN-SSRILSESQ---ALEPIVDG
    PD683_g3766.t1                  ------------------------------------------------------------MLFSVG-------LLALAALPSS-----FGAVIQAR-QD-DPENPPRDPQPPPPGPIF--
    PD683_g3895.t1                  ---------------------------------------------------MLPSTIFSVFAL---------VGSALAQQPPK-----------------VNHD--------SINPIRDT
    PD683_g4100.t1                  ---------------------------------------------------MSPSLISIVTWL---------AAASSTLAAPL---------LESR-AV-IGHD--------AVVGFPQT
    PD683_g5003.t1                  M----------------------------------------AHAIF--SPGLWQPALHRVVLL---------LVLYLALVHPS-----NSSVIQRR-AA-------------PPQKLP--
    PD683_g5003.t2                  ------------------------------------------------------------------------------------------------------------------------
    PD683_g5713.t1                  -------------------------------------------------------MVSKIFST---------LASIALVAAGP---------ISLR-AV-VPHD--------SLNPVTQR
    PD683_g7092.t1                  ---------------------------------------------------MQHTLFSTAALL---------GALSVVNASPA-------PILRRD----------------IITALP--
    PD683_g8130.t1                  ------------------------------------------------MPSLRTASFSAVAAL---------LLLPAVIATPLPDTP-TTKLIRRD----------------LLQPLG--
    PD736_g919.t1                   ---------------------------------------------------MSPSLISIVTWL---------AAASSTLAAPL---------LESR-AV-IAHD--------AVVGFPQT
    PD736_g8670.t1                  ---------------------------------------------------MLPSTILSVLAF---------AGSTLAQWGPP--------------GT-VNHD--------SLNPVSDT
    PD736_g9411.t1                  -------------------------------------------------------MVSKIMST---------LASIALVAASP---------LSLR-AV-VAHD--------SLNPIEQR
    PD736_g10271.t1                 ---------------------------------------------------MQNTLLTTAALL---------GALSAVHASPA-------PILRRD----------------IITALP--
    PD736_g10840.t1                 M----------------------------------------SRSDF--R-EAWHPAITRILLF---------LVFCLVLVLPS-----DASVIQRR-AD-------------PPQKLP--
    PD736_g10939.t1                 ---------------------------------------------------MYPSKIVALVAF---------FATGS-IAAPAAGTPEGFSNIERR-AVQVPHS--------SLNALSQN
    PD739_g34.t1                    M----------------------------------------SRSEF--R-EAWHPAITRILLF---------LVFCLVLVLPA-----DASVIQRR-AD-------------PPQKLP--
    PD739_g131.t1                   ---------------------------------------------------MYPSKIVALVAF---------FATGS-IAAPAVGTPESFSNIERR-AVQVPHS--------SLNALDQN
    PD739_g4443.t1                  ---------------------------------------------------MQNTLLTTAALL---------GALSAVHASPA-------PIVRRD----------------IITALP--
    PD739_g7436.t1                  ---------------------------------------------------MSPSLISIVTWL---------AAASSTLAAPL---------LESR-AV-IAHD--------AVVGFPQT
    PD739_g8512.t1                  ---------------------------------------------------MLPSTILSVLAF---------AGSTLAQWGPP--------------GT-VNHD--------SLNPVSDT
    PD739_g10815.t1                 -------------------------------------------------------MVSKIMST---------LASIALVAASP---------LSLR-AV-VAHD--------SLNPIEQR
    PD747_g20.t1                    M----------------------------------------SQPDP--H-GLRITAMRRIVLF---------LLFYLTLVSPT-----HASVIQRR-AD-------------PPQKLP--
    PD747_g277.t1                   ---------------------------------------------------MHASKIAAVLAF---------FASSS-IAAPAADALDGFSRIERR-AAEVPHS--------SLNALGQN
    PD747_g4174.t1                  ---------------------------------------------------MQNTLLTTAALL---------GALSAVNASPA-------PIMRRD----------------ILTALP--
    PD747_g5167.t1                  ---------------------------------------------------MSPSLISIVTWL---------AAASSTLAAPL---------LEAR-AV-IAHD--------AVVGFSQT
    PD747_g9207.t1                  ------------------------------------------------MPSFATLSATAVALL---------YLLSSTLANPTP-------LERRD----------------LLQPLG--
    PD747_g9751.t1                  -------------------------------------------------------MVSKIMTT---------LASIALAVASP---------LNLR-AV-VAHD--------SLNPIDQR
    PD747_g10403.t1                 ------------------------------------------------------------MLFPMS-------LLALSSLPLS-----WGLAIQAR-QD-DPLEPPGIPSGPPPGPIF--
    PD747_g10498.t1                 ---------------------------------------------------MGLARVLSLLVYF-------SALLVATVASPV-DVLEQSHELSRR-GT-IPHN--------QVKGFSQA
    PD747_g10549.t1                 ---------------------------------------------------MLPSTILSVLAF---------AGSAIAQWGPP--------------GT-VNHD--------SLNPVRDT
    T2_g2030.t1                     -------------------------------------------------------MLYSKMLTLRN----IAVVTAMVLSVPS-----TASAMRRQ-NN-SSRILSESQ---ALEPIVDG
    T2_g2030.t2                     ------------------------------------------------------------MLTLRN----IAVVTAMVLSVPS-----TASAMRRQ-NN-SSRILSESQ---ALEPIVDG
    T2_g3751.t1                     M----------------------------------------AHAIF--SPGLWQPALHRVVLL---------LVLYLALVHPS-----NSSVIQRR-AA-------------PPQKLP--
    T2_g3751.t2                     ------------------------------------------------------------------------------------------------------------------------
    T2_g4661.t1                     ---------------------------------------------------MSPSLISIVTWL---------AAASSTLAAPL---------LESR-AV-IGHD--------AVVGFPQT
    T2_g4868.t1                     ---------------------------------------------------MLPSTIFSVFAL---------VGSALAQQPPK-----------------VNHD--------SINPIRDT
    T2_g5004.t1                     ------------------------------------------------------------MLFSVG-------LLALAAFPSS-----FGAVIQAR-QD-DPENPPRDPQPPPPGPIF--
    T2_g5801.t1                     -------------------------------------------------------MVSKIFST---------LASIALVAAGP---------ISLR-AV-VPHD--------SLNPVTQR
    T2_g7120.t1                     ------------------------------------------------MPSLRTASFSAVAAL---------LLLPAVIATPLPDTP-TTKLIRRD----------------LLQPLG--
    T2_g7997.t1                     ---------------------------------------------------MQHTFFSTAALL---------GALSAVNASPA-------PILRRD----------------IITALP--
    VSO1_g539.t1                    ---------------------------------------------------MSPSLISIVTWL---------AAASSTLAAPL---------LESR-AV-IAHD--------AVVGFPQT
    VSO1_g1387.t1                   ---------------------------------------------------MHPSKIVALAAY---------FATGS-IAAPAAGTPEGFSNIERR-AVEVPHS--------SLNALPQN
    VSO1_g1731.t1                   ---------------------------------------------------MLPSTILSVLAF---------AGSTLAQWGPP--------------GT-VNHD--------SLSPIPDT
    VSO1_g1937.t1                   ------------------------------------------------MPSLNMLAVIAVTSL---------TILPFALANPLP-------LERRE----------------LLTRLS--
    VSO1_g4972.t1                   ---------------------------------------------------MQNTLLTTAALL---------GALSAVHASPA-------PILKRD----------------ILTALP--
    VSO1_g5184.t1                   -------------------------------------------------------MVSKIMST---------LASIALVAASP---------LSLR-AV-VAHD--------SLNPIDQR
    VL20_g7800.t1                   ------------------------------------------------------------MLFLRN----IAVVTAMVLSVPS-----TASVMRRQ-NN-SSRILSESP---ALEPIVNG
    VL20_g9415.t1                   ---------------------------------------------------MQHTLLSTAALL---------GALSAVNASPA-------PILRRD----------------IITALP--
    VL20_g11025.t1                  ---------------------------------------------------MLPSMIFSVFAL---------VGSALAQQPPK-----------------VNHD--------SINPIRDT
    VL20_g11172.t1                  ------------------------------------------------------------MLSLRN----IAVVTAMVLSVPS-----TASVMRRQ-SN-SSRILSDSQ---ALEPIVGG
    VL20_g12965.t1                  ------------------------------------------------MTSLRTASFSAVAAL---------LLLPAVIATPLPDTP-TTKLTRRD----------------LLQPLG--
    VL20_g13837.t1                  ---------------------------------------------------MSPSLISIVTWL---------AAASSTLAAPL---------LESR-AV-INHD--------AVVGFPQT
    VL20_g14042.t1                  ---------------------------------------------------MLPSTIFSVFAL---------VGSALAQHPPK-----------------VNHD--------SINPVRDT
    VL20_g14171.t1                  ------------------------------------------------------------MLFSVG-------LLALAALPSS-----FGAVIQAR-QD-DPENPPRDPQPPPPGPIF--
    VL20_g14284.t1                  ---------------------------------------------------MQHTLLSTAALL---------GALSAVNASPA-------PILRRD----------------IITALP--
    VL20_g17450.t1                  -------------------------------------------------------MVSKIFST---------LASIALVAAGP---------ISLR-AV-VPHD--------SLNPVTQR
    VL20_g18857.t1                  -------------------------------------------------------MVSKIFST---------LASIALVAAGP---------VSLR-AV-VPHD--------SLNPVTQR
    VL20_g4752.t1                   ---------------------------------------------------MSPSLISIVTWL---------AAASSTLAAPL---------LESR-AV-IAHD--------AVVGFPQT
    VL20_g5938.t1                   MIA------------------------------------KVRELIFYNMPSLRTASFSAVAAL---------LLLPAVIATPLPDTP-PTKLIRRD----------------LLQPLG--
    VL20_g5938.t2                   ------------------------------------------------MPSLRTASFSAVAAL---------LLLPAVIATPLPDTP-PTKLIRRD----------------LLQPLG--
    VL20_g6061.t1                   ------------------------------------------------------------MLFSVG-------LLALAALPSS-----FGAVIQAR-QD-DPENPPRDPQPPPPGPIF--
    VL20_g18964.t1                  MIA------------------------------------KVRELIFYNMPSLRTASFSAVAAL---------LLLPAVIATPLPDTP-PTKLIRRD----------------LLQPLG--
    VL20_g18964.t2                  ------------------------------------------------MPSLRTASFSAVAAL---------LLLPAVIATPLPDTP-PTKLIRRD----------------LLQPLG--
    VLB2_g585.t1                    ------------------------------------------------------------MLFLRN----IAVVTAMVLSVPS-----TASVMRRQ-NN-SSRILSESP---ALEPIVNG
    VLB2_g1761.t1                   ---------------------------------------------------MLPSMIFSVFAL---------VGSALAQQPPK-----------------VNHD--------SINPIRDT
    VLB2_g2033.t1                   ------------------------------------------------------------MLSLRN----IAVVTAMVLSVPS-----TASVMRRQ-SN-SSRILSDSQ---ALEPIVGG
    VLB2_g12050.t1                  -------------------------------------------------------MVSKIFST---------LASIALVAAGP---------ISLR-AV-VPHD--------SLNPVTQR
    VLB2_g12617.t1                  ------------------------------------------------MTSLRTASFSAVAAL---------LLLPAVIATPLPDTP-TTKLTRRD----------------LLQPLG--
    VLB2_g13499.t1                  ---------------------------------------------------MSPSLISIVTWL---------AAASSTLAAPL---------LESR-AV-INHD--------AVVGFPQT
    VLB2_g13703.t1                  ---------------------------------------------------MLPSTIFSVFAL---------VGSALAQHPPK-----------------VNHD--------SINPVRDT
    VLB2_g13833.t1                  ------------------------------------------------------------MLFSVG-------LLALAALPSS-----FGAVIQAR-QD-DPENPPRDPQPPPPGPIF--
    VLB2_g14318.t1                  ---------------------------------------------------MQHTLLSTAALL---------GALSAVNASPA-------PILRRD----------------IITALP--
    VLB2_g15187.t1                  MIA------------------------------------KVRELIFYNMPSLRTASFSAVAAL---------LLLPAVIATPLPDTP-PTKLIRRD----------------LLQPLG--
    VLB2_g15187.t2                  ------------------------------------------------MPSLRTASFSAVAAL---------LLLPAVIATPLPDTP-PTKLIRRD----------------LLQPLG--
    VLB2_g16101.t1                  ---------------------------------------------------MSPSLISIVTWL---------AAASSTLAAPL---------LESR-AV-IAHD--------AVVGFPQT
    VLB2_g16543.t1                  ---------------------------------------------------MQHTLLSTAALL---------GALSAVNASPA-------PILRRD----------------IITALP--
    VLB2_g19098.t1                  M----------------------------------------AHASF--SPGLWQPALHRIVLL---------LVLYLALVHPS-----NSSVIQRR-AA-------------PPQKLP--
    VLB2_g5814.t1                   -------------------------------------------------------MVSKIFST---------LASIALVAAGP---------VSLR-AV-VPHD--------SLNPVTQR
    VLB2_g6534.t1                   ------------------------------------------------------------MLFSVG-------LLALAALPSS-----FGAVIQAR-QD-DPENPPRDPQPPPPGPIF--
    PD589_g51.t1                    --------------------------------------------------------------------------MVVKSSQLLPDTP-TTKLIRRD----------------LLQPLG--
    PD589_g856.t1                   ------------------------------------------------------------------------------------------------------------------------
    PD589_g7665.t1                  ---------------------------------------------------MQHTLLSTAALL---------GALSAVNASPA-------PILRRD----------------IITALP--
    PD589_g13197.t1                 ------------------------------------------------------------MLFLRN----IAVVTAMVLSVPS-----TASVMRRQ-NN-SSRILSESP---ALEPIVNG
    PD589_g13425.t1                 -------------------------------------------------------MVSKIFST---------LASIALVAAGP---------ISLR-AA-VPHD--------SLNPVTQR
    PD589_g14403.t1                 ------------------------------------------------------------MLFSVG-------LLALAALPSS-----FGAVIQAR-QD-DPENPPRDPQPPPPGPRI--
    PD589_g14539.t1                 ------------------------------------------------------------MLFSVG-------LLALAALPSS-----FGAVIQAR-QD-DPENPPRDPQPPPPGPIF--
    PD589_g14661.t1                 ---------------------------------------------------MLPSAVFSVFAL---------VGSALAQQPPK-----------------VNHD--------SINPVRDT
    PD589_g15310.t1                 -------------------------------------------------------MVSKIFST---------LASIALVAAGP---------VSLR-AV-VPHD--------SLNPVTQR
    PD589_g16171.t1                 MIA------------------------------------KVRELIFYNMPSLRTASFSAVAAL---------LLLPAVIATPLPDTP-PTKLIRRD----------------LLQPLG--
    PD589_g16171.t2                 ------------------------------------------------MPSLRTASFSAVAAL---------LLLPAVIATPLPDTP-PTKLIRRD----------------LLQPLG--
    PD589_g17659.t1                 ---------------------------------------------------MQHTLLSTAALL---------GALSAVNASPA-------PILRRD----------------IITALP--
    PD589_g18192.t1                 ---------------------------------------------------MSPSLISIVTWL---------AAASSTLAAPL---------LESR-AV-INHD--------AVVGFPQT
    PD589_g3050.t1                  ---------------------------------------------------MLPSMIFSVFAL---------VGSALAQQPPK-----------------VNHD--------SINPIRDT
    PD589_g6680.t1                  ------------------------------------------------------------MLSLRN----IAVVTAMVLSVPS-----TASVMRRQ-SN-SSRILSDSQ---ALEPIVGG
    PD589_g6775.t1                  ---------------------------------------------------MSPSLISIVTWL---------AAASSTLAAPL---------LESR-AV-IAHD--------AVVGFPQT
    Vt305_g1664.t1                  ---------------------------------------------------MLPSTILSVLAF---------AGSTLAQWGPP--------------GT-VNHD--------SLSPIPDT
    Vt305_g2624.t1                  ---------------------------------------------------MQNTLLTTAALL---------GALSAVHASPA-------PILRRD----------------ILTALP--
    Vt305_g3295.t1                  -------------------------------------------------------MVSKIMST---------LASIALVAASP---------LSLR-AV-VAHD--------SLNPIDQR
    Vt305_g4616.t1                  ---------------------------------------------------MHPSKIVALAAY---------FATGS-IAAPAAGTPEGFSNIERR-AVEVPHS--------SLNALPQN
    Vt305_g7985.t1                  ---------------------------------------------------MSPSLISIVTWL---------AAASSTLAAPL---------LESR-AV-IAHD--------AVVGFPQT
    WCS072_g2037.t1                 -------------------------------------------------------MLYSKMLTLRN----IAVVTAMVLSVPS-----TASAMRRQ-NN-SSRILSESQ---ALEPIVDG
    WCS072_g2037.t2                 ------------------------------------------------------------MLTLRN----IAVVTAMVLSVPS-----TASAMRRQ-NN-SSRILSESQ---ALEPIVDG
    WCS072_g3872.t1                 ------------------------------------------------------------MLFSVG-------LLALAAFPSS-----FGAVIQAR-QD-DPENPPRDPQPPPPGPIF--
    WCS072_g4007.t1                 ---------------------------------------------------MLPSTIFSVFAL---------VGSALAQQPPK-----------------VNHD--------SINPIRDT
    WCS072_g4219.t1                 ---------------------------------------------------MSPSLISIVTWL---------AAASSTLAAPL---------LESR-AV-IGHD--------AVVGFPQT
    WCS072_g5132.t1                 M----------------------------------------AHAIF--SPGLWQPALHRVVLL---------LVLYLALVHPS-----NSSVIQRR-AA-------------PPQKLP--
    WCS072_g5132.t2                 ------------------------------------------------------------------------------------------------------------------------
    WCS072_g5615.t1                 -------------------------------------------------------MVSKIFST---------LASIALVAAGP---------ISLR-AV-VPHD--------SLNPVTQR
    WCS072_g6396.t1                 ---------------------------------------------------MQHTFFSTAALL---------GALSAVNASPA-------PILRRD----------------IITALP--
    WCS072_g8181.t1                 ------------------------------------------------MPSLRTASFSAVAAL---------LLLPAVIATPLPDTP-TTKLIRRD----------------LLQPLG--
    VDAG_JR2_Chr1g22850a-00001      ------------------------------------------------------------MLFLQN----IAVVTAMVLSVPS-----TASVMRRQ-NN-SSRILSESP---ALEPIVNG
    VDAG_JR2_Chr2g05460a-00001      MMIRLAGTPRREKRPGSSLRTAFPPPPHRLLIHRPIW----SASCS--TVAMSPSLISIVTWL---------AAASSTLAAPL---------LESR-AV-INHD--------AVVGFPQT
    VDAG_JR2_Chr6g08770a-00001      ---------------------------------------------------MLPSAVFSVFAL---------VGIALAQQPPK-----------------VNHD--------SINPVRDT
    VDAG_JR2_Chr6g10260a-00001      ------------------------------------------------------------MLFSVG-------LLALAALPSS-----FGAVIQAR-QD-DPENPPRDPQPPPPGPIF--
    VDAG_JR2_Chr3g00800a-00001      ---------------------------------------------------MQHTLLSTAALL---------GALSAVNASPA-------PILRRD----------------IITALP--
    VDAG_JR2_Chr4g05950a-00001      -------------------------------------------------------MVSKIFST---------LASIALVAAGP---------VSLR-AV-VPHD--------SLNPVTQR
    VDAG_JR2_Chr8g10650a-00001      MVV------------------------------------KVRELIFYNMPSLRTASFSAVAAL---------LLLPAVIATPLPDTP-TTKLIRRD----------------LRQPLG--
    JR2_g2720.t1                    M----------------------------------------AHASF--SPSLWQPALHRIVLL---------LVLYLALVHPS-----NCSVIQRR-AA-------------PPQKLP--

    Selected Cols:                                                                                                                                          

    Gaps Scores:                                                                                                                                            
    Similarity Scores:                                                                                                                                      

                                           130       140       150       160       170       180       190       200       210       220       230       240
                                    =========+=========+=========+=========+=========+=========+=========+=========+=========+=========+=========+=========+
    12008_g358.t1                   VQTGAIGDAIAKFNPLL----HIANGCQPYTAVNDAGDT---S--GGLQDS--------------------G-NISAGCR------DQSKG----QTYARAKVVNGQLAIMYSFYMPKDQ
    12008_g4461.t1                  ---GNADEIENKFQPIL---DFDTDGCYNTAAIDPDGNI---N--PGKGAT--------------------G-TPQGDCRDPP---QLENS----NVYSRRRCNNGVCAIMYEYYFEKDQ
    12008_g7340.t1                  ---GSAWSEQEKWCPAL---DYDTDSCYNTVAISPSGQL---N--AGQDENK-----------------PAG-EILGWCRKEV---HLQQT----NIYVRSRCNNGWCVHMYDYYFEADF
    12008_g7652.t1                  VPSGVSGQLMLKFKPYL----KVFNGCVPFPAVNAGGDT---G--GGLATS--------------------G-SSNGGC-------SSSAG----QVYARAGSYNGANAILYAWYMPKDA
    12008_g7834.t1                  ---GRAPDLDKRFQPAL---DFDTDSCYNVPAIGPNGDL---A--IGMYPF--------------------EWPPQAGCRNEE---MLDRG----NVYSRQRCNNGYCVIFYAYYFQKDT
    12008_g8465.t1                  --HDFAYYFEVKFQPLV---DFDTDSCYSVPAMTMDGTA---S--EGLSPS--------------------D--DVGPCRPRS---ALDRT----NVYVRGRCNRGWCAFVYAYYFQMDW
    12008_g8848.t1                  L--GPNGDMIRKFQPLL----HIAHGCQPYSAVNTRGEV---N--AGLQDS--------------------G-TTAGGCK------ETSKG----QTYARSMTLNGQFGIMYAWYWPKDQ
    85S_g58.t1                      ---GSAWSEQEKWCPAL---DYDTDSCYNTVAISPSGQL---N--AGQDENK-----------------PAG-EILGWCRKEV---RLQQT----NIYVRSRCNNGWCVHMYDYYFEADF
    85S_g9168.t1                    VPSGVSGQLMLKFKPYL----KVFNGCVPFPAVNAGGDT---G--GGLATS--------------------G-SSNGGC-------SSSAG----QVYARAGSYNGANAILYAWYMPKDA
    85S_g9863.t1                    ---GNADEIENKFQPIL---DFDTDGCYNTAAIDPDGNI---N--PGKGAT--------------------G-TPQGDCRDPP---QLENS----NVYSRRRCNNGVCAIMYEYYFEKDQ
    85S_g7040.t1                    --HDFAYYFEVKFQPLV---DFDTDSCYSVPAMTMDGTA---S--EGLSPS--------------------D--DVGPCRPRS---ALDRT----NVYVRGRCNRGWCAFVYAYYFQMDW
    85S_g3154.t1                    VQTGAIGDAIAKFNPLL----HIANGCQPYTAVNDAGDT---S--GGLQDS--------------------G-NISAGCR------DQSKG----QTYARAKVVNGQLAIMYSFYMPKDQ
    85S_g4608.t1                    ---GRAPDLDKRFQPAL---DFDTDSCYNVPAIGPNGDL---A--IGMYPF--------------------EWPPQAGCRNEE---MLDRG----NVYSRQRCNNGYCVIFYAYYFQKDT
    85S_g4744.t1                    L--GPNGDMIRKFQPLL----HIAHGCQPYSAVNTRGEV---N--AGLQDS--------------------G-TTAGGCK------ETSKG----QTYARSMTLNGQFGIMYAWYWPKDQ
    CBS385.49_g66.t1                -----------RFQPAL---DFDTDSCYNVPAIGPNGDL---A--IGMYPF--------------------EWPPQAGCRNEE---MLDRG----NVYSRQRCNNGYCVIFYAYYFQKDT
    CBS385.49_g189.t1               L--GPNGDMIRKFQPLL----HIAHGCQPYSAVNTRGEV---N--AGLQDS--------------------G-TTAGGCK------ETSKG----QTYARSMTLNGQFGII---------
    CBS385.49_g400.t1               VPSGVSGQLMLKF--------AVPQGVQRLRAVSGRQRRRRHGEHGGLATS--------------------G-SSNGGC-------SSSAG----QVYARAGSYNGANAILYAWYMPKDA
    CBS385.49_g400.t2               VPSGVSGQLMLN---------------------------------GGLATS--------------------G-SSNGGC-------SSSAG----QVYARAGSYNGANAILYAWYMPKDA
    CBS385.49_g1381.t1              ---GSAWSEQEKWCPAL---DYDTDSCYNTVAISPSGQL---N--AGQDENK-----------------PAG-EILGWCRKEV---HLQQT----NIYVRSRCNNGWCVHMYDYYFEADF
    CBS385.49_g5607.t1              VQTGAIGDAIAKFNPLL----HIANGCQPYTAVNDAGDT---S--GGLQDS--------------------G-NISAGCR------DQSKG----QTYARAKVVNGQLAIMYSFYMPKDQ
    CBS385.49_g7723.t1              ---GNADEIENKFQPIL---DFDTDGCYNTAAIDPDGNI---N--PGKGAT--------------------G-TPQGDCRDP--------------LSSRTAMFTPVAAATTASALSCT-
    CBS385.49_g8721.t1              --HDFAYYFEVKFQPLV---DFDTDSCYSVPAMTMDGTA---S--EGLSPS--------------------D--DVGPCRPCS---ALDRT----NVYVRGRCNRGWCAFVYAYYFQMDW
    CQ2_g254.t1                     --HDFAYYFEVKFQPLV---DFDTDSCYSVPAMTMDGTA---S--EGLSPS--------------------D--DVGPCRPRS---ALDRT----NVYVRGRCNRGWCAFVYAYYFQMDW
    CQ2_g2332.t3                    ---KRATENDLRYQPAL---DFDTDSCYNVPAIGCDGKI---A--EGLEPD--------------------G--TTKDYRDLA---DLDNT----NVYSRQRCNSGWCAYMYDYYFEKDH
    CQ2_g2332.t4                    ---KRATENDLRYQPAL---DFDTDSCYNVPAIGCDGKI---A--EGLEPD--------------------G--TTKDYRDLA---DLDNT----NVYSRQRCNSGWCAYMYDYYFEKDH
    CQ2_g3243.t1                    VPSGVSGQLMLKFKPYL----KVFNGCVPFPAVNAGGDT---G--GGLATS--------------------G-SSNGGC-------SSSAG----QVYARAGSYNGANAILYAWYMPKDA
    CQ2_g3454.t1                    L--GPNGDMIRKFQPLL----HIAHGCQPYSAVNTRGEV---N--AGLQDS--------------------G-TTAGGCK------ETSKG----QTYARSMTLNGQFGIMYAWYWPKDQ
    CQ2_g3584.t1                    ---GRAPDLDKRFQPAL---DFDTDSCYNVPAIGPNGDL---A--IGMYPF--------------------EWPPQAGCRNEE---MLDRG----NVYSRQRCNNGYCVIFYAYYFQKDT
    CQ2_g4742.t1                    ---GNADEIENKFQPIL---DFDTDGCYNTAAIDPDGNI---N--PGKGAT--------------------G-TPQGDCRDPP---QLENS----NVYSRRRCNNGVCAIMYEYYFEKDQ
    CQ2_g7512.t1                    VQTGAIGDAIAKFNPLL----HIANGCQPYTAVNDAGDT---S--GGLQDS--------------------G-NISAGCR------DQSKG----QTYARAKVVNGQLAIMYSFYMPKDQ
    CQ2_g8820.t1                    ---GSAWSEQEKWCPAL---DYDTDSCYNTVAISPSGQL---N--AGQDENK-----------------PAG-EILGWCRKEV---HLQQT----NIYVRSRCNNGWCVHMYDYYFEADF
    DAR82592_g1639.t1               --HDFAYYFEVKFQPLV---DFDTDSCYSVPAMTMDGTA---S--EGLSPS--------------------D--DVGP----------------------GRCNRGWCAFVYAYYFQMDW
    DAR82592_g8837.t1               ---GSAWSEQEKWCPAL---DYDTDSCYNTVAISPSGQL---N--AGQDENK-----------------PAG-EILGWCRKEV---HLQQT----NIYVRSRCNNGWCVHMYDYYFEADF
    DAR82592_g4435.t1               L--GPNGDMIRKFQPLL----HIAHGCQPYSAVNTRGEV---N--AGLQDS--------------------G-TTAGGCK------ETSKG----QTYARSMTLNGQFGIMYAWYWPKDQ
    DAR82592_g4647.t1               VPSGVSGQLMLKFKPYL----KVFNGCVPFPAVNAGGDT---G--GGLATS--------------------G-SSNGGC-------SSSAG----QVYARAGSYNGANAILYAWYMPKDA
    DAR82592_g7268.t1               VQTGAIGDAIAKFNPLL----HIANGCQPYTAVNDAGDT---S--GGLQDS--------------------G-NISAGCR------DQSKG----QTYARAKVVNGQLAIMYSFYMPKDQ
    DAR82592_g4307.t1               ---GRAPDLDKRFQPAL---DFDTDSCYNVPAIGPNGDL---A--IGMYPF--------------------EWPPQAGCRNEE---MLDRG----NVYSRQRCNNGYCVIFYAYYFQKDT
    DAR82592_g3508.t1               ---KRATENDLRYQPAL---DFDTDSCYNVPAIGCDGKI---A--EGLEPD--------------------G--TTKDYRDLA---DLDNT----NVYSRQRCNSGWCAYMYDYYFEKDH
    DAR82592_g62.t1                 ---GNADEIENKFQPIL---DFDTDGCYNTAAIDPDGNI---N--PGKGAT--------------------G-TPQGDCRDP--------------LSSRTAMFTPVAAATTASALSCT-
    DAR83138_g1199.t1               --HDFAYYFEVKFQPLV---DFDTDSCYSVPAMTMDGTA---S--EGLSPS--------------------D--DVGPCRPRS---ALDRT----NVYVRGRCNRGWCAFVYAYYFQMDW
    DAR83138_g60.t1                 ---GNADEIENKFQPIL---DFDTDGCYNTAAIDPDGNI---N--PGKGAT--------------------G-TPQGDCRDPP---QLENS----NVYSRRRCNNGVCAIMYEYYFEKDQ
    DAR83138_g7641.t1               VQTGAIGDAIAKFNPLL----HIANGCQPYTAVNDAGDT---S--GGLQDS--------------------G-NISAGCR------DQSKG----QTYARAKVVNGQLAIMYSFYMPKDQ
    DAR83138_g9085.t1               ---GSAWSEQEKWCPAL---DYDTDSCYNTVAISPSGQL---N--AGQDENK-----------------PAG-EILGWCRKEV---HLQQT----NIYVRSRCNNGWCVHMYDYYFEADF
    DAR83138_g3565.t3               ---KRATENDLRYQPAL---DFDTDSCYNVPAIGCDGKI---A--EGLEPD--------------------G--TTKDYRDLA---DLDNT----NVYSRQRCNSGWCAYMYDYYFEKDH
    DAR83138_g4173.t1               ---GRAPDLDKRFQPAL---DFDTDSCYNVPAIGPNGDL---A--IGMYPF--------------------EWPPQAGCRNEE---MLDRG----NVYSRQRCNNGYCVIFYAYYFQKDT
    DAR83138_g4314.t1               L--GPNGDMIRKFQPLL----HIAHGCQPYSAVNTRGEV---N--AGLQDS--------------------G-TTAGGCK------ETSKG----QTYARSMTLNGQFGIMYAWYWPKDQ
    DAR83138_g4535.t1               VPSGVSGQLMLKFKPYL----KVFNGCVPFPAVNAGGDT---G--GGLATS--------------------G-SSNGGC-------SSSAG----QVYARAGSYNGANAILYAWYMPKDA
    DAR83143_g1427.t1               --HDFAYYFEVKFQPLV---DFDTDSCYSVPAMTMDGTA---S--EGLSPS--------------------D--DVGPCRPRS---ALDRT----NVYVRGRCNRGWCAFVYAYYFQMDW
    DAR83143_g4194.t1               ---GRAPDLDKRFQPAL---DFDTDSCYNVPAIGPNGDL---A--IGMYPF--------------------EWPPQAGCRNEE---MLDRG----NVYSRQRCNNGYCVIFYAYYFQKDT
    DAR83143_g4335.t1               L--GPNGDMIRKFQPLL----HIAHGCQPYSAVNTRGEV---N--AGLQDS--------------------G-TTAGGCK------ETSKG----QTYARSMTLNGQFGIMYAWYWPKDQ
    DAR83143_g4545.t1               VPSGVSGQLMLKFKPYL----KVFNGCVPFPAVNAGGDT---G--GGLATS--------------------G-SSNGGC-------SSSAG----QVYARAGSYNGANAILYAWYMPKDA
    DAR83143_g7287.t1               VQTGAIGDAIAKFNPLL----HIANGCQPYTAVNDAGDT---S--GGLQDS--------------------G-NISAGCR------DQSKG----QTYARAKVVNGQLAIMYSFYMPKDQ
    DAR83143_g3521.t1               ---KRATENDLRYQPAL---DFDTDSCYNVPAIGCDGKI---A--EGLEPD--------------------G--TTKDYRDLA---DLDNT----NVYSRQRCNSGWCAYMYDYYFEKDH
    DAR83143_g8715.t1               ---GSAWSEQEKWCPAL---DYDTDSCYNTVAISPSGQL---N--AGQDENK-----------------PAG-EILGWCRKEV---HLQQT----NIYVRSRCNNGWCVHMYDYYFEADF
    DAR83143_g61.t1                 ---GNADEIENKFQPIL---DFDTDGCYNTAAIDPDGNI---N--PGKGAT--------------------G-TPQGDCRDPP---QLENS----NVYSRRRCNNGVCAIMYEYYFEKDQ
    DAR83175_g3551.t1               ---KRATENDLRYQPAL---DFDTDSCYNVPAIGCDGKI---A--EGLEPD--------------------G--TTKDYRDLA---DLDNT----NVYSRQRCNSGWCAYMYDYYFEKDH
    DAR83175_g4514.t1               VPSGVSGQLMLKFKPYL----KVFNGCVPFPAVNAGGDT---G--GGLATS--------------------G-SSNGGC-------SSSAG----QVYARAGSYNGANAILYAWYMPKDA
    DAR83175_g4731.t1               L--GPNGDMIRKFQPLL----HIAHGCQPYSAVNTRGEV---N--AGLQDS--------------------G-TTAGGCK------ETSKG----QTYARSMTLNGQFGIMYAWYWPKDQ
    DAR83175_g7439.t1               VQTGAIGDAIAKFNPLL----HIANGCQPYTAVNDAGDT---S--GGLQDS--------------------G-NISAGCR------DQSKG----QTYARAKVVNGQLAIMYSFYMPKDQ
    DAR83175_g2075.t1               --HDFAYYFEVKFQPLV---DFDTDSCYSVPAMTMDGTA---S--EGLSPS--------------------D--DVGPCRPRS---ALDRT----NVYVRGRCNRGWCAFVYAYYFQMDW
    DAR83175_g5817.t1               ---GRAPDLDKRFQPAL---DFDTDSCYNVPAIGPNGDL---A--IGMYPF--------------------EWPPQAGCRNEE---MLDRG----NVYSRQRCNNGYCVIFYAYYFQKDT
    DAR83175_g61.t1                 ---GNADEIENKFQPIL---DFDTDGCYNTAAIDPDGNI---N--PGKGAT--------------------G-TPQGDCRDPP---QLENS----NVYSRRRCNNGVCAIMYEYYFEKDQ
    DAR83175_g8890.t1               ---GSAWSEQEKWCPAL---DYDTDSCYNTVAISPSGQL---N--AGQDENK-----------------PAG-EILGWCRKEV---HLQQT----NIYVRSRCNNGWCVHMYDYYFEADF
    DK045_g927.t1                   VPSGVSGQLMLKFKPYL----KVFNGCVPFPAVNAGGDT---G--GGLATS--------------------G-SSNGGC-------SSSAG----QVYARAGSYNGANAILYAWYMPKDA
    DK045_g1138.t1                  L--GPNGDMIRKFQPLL----HIAHGCQPYSAVNTRGEV---N--AGLQDS--------------------G-TTAGGCK------ETSKG----QTYARSMTLNGQFGIMYAWYWPKDQ
    DK045_g1272.t1                  ---GRAPDLDKRFQPAL---DFDTDSCYNVPAIGPNGDL---A--IGMYPF--------------------EWPPQAGCRNEE---MLDRG----NVYSRQRCNNGYCVIFYAYYFQKDT
    DK045_g2451.t1                  ---GNADEIENKFQPIL---DFDTDGCYNTAAIDPDGNI---N--PGKGAT--------------------G-TPQGDCRDPP---QLENS----NVYSRRRCNNGVCAIMYEYYFEKDQ
    DK045_g3667.t1                  --HDFAYYFEVKFQPLV---DFDTDSCYSVPAMTMDGTA---S--EGLSPS--------------------D--DVGPCRPRS---ALDRT----NVYVRGRCNRGWCAFVYAYYFQMDW
    DK045_g5985.t1                  ---GSAWSEQEKWCPAL---DYDTDSCYNTVAISPSGQL---N--AGQDENK-----------------PAG-EILGWCRKEV---HLQQT----NIYVRSRCNNGWCVHMYDYYFEADF
    DK045_g9032.t1                  VQTGAIGDAIAKFNPLL----HIANGCQPYTAVNDAGDT---S--GGLQDS--------------------G-NISAGCR------DQSKG----QTYARAKVVNGQLAIMYSFYMPKDQ
    GF1192_g8987.t1                 L--GPNGDMIRKFQPLL----HIAHGCQPYSAVNTRGEV---N--AGLQDS--------------------G-TTAGGCK------ETSKG----QTYARSMTLNGQFGIMYAWYWPKDQ
    GF1192_g9126.t1                 ---GRAPDLDKRFQPAL---DFDTDSCYNVPAIGPNGDL---A--IGMYPF--------------------EWPPQAGCRNEE---MLDRG----NVYSRQRCNNGYCVIFYAYYFQKDT
    GF1192_g257.t1                  --HDFAYYFEVKFQPLV---DFDTDSCYSVPAMTMDGTA---S--EGLSPS--------------------D--DVGPCRPRS---ALDRT----NVYVRGRCNRGWCAFVYAYYFQMDW
    GF1192_g1728.t1                 VQTGAIGDAIAKFNPLL----HIANGCQPYTAVNDAGDT---S--GGLQDS--------------------G-NISAGCR------DQSKG----QTYARAKVVNGQLAIMYSFYMPKDQ
    GF1192_g2840.t1                 ---GNADEIENKFQPIL---DFDTDGCYNTAAIDPDGNI---N--PGKGAT--------------------G-TPQGDCRDPP---QLENS----NVYSRRRCNNGVCAIMYEYYFEKDQ
    GF1192_g5025.t1                 VPSGVSGQLMLKFKPYL----KVFNGCVPFPAVNAGGDT---G--GGLATS--------------------G-SSNGGC-------SSSAG----QVYARAGSYNGANAILYAWYMPKDA
    GF1192_g6069.t1                 ---GSAWSEQEKWCPAL---DYDTDSCYNTVAISPSGQL---N--AGQDENK-----------------PAG-EILGWCRKEV---HLQQT----NIYVRSRCNNGWCVHMYDYYFEADF
    GF1207_g2093.t1                 --HDFAYYFEVKFQPLV---DFDTDSCYSVPAMTMDGTA---S--EGLSPS--------------------D--DVGP----------------------GRCNRGWCAFVYAYYFQMDW
    GF1207_g4890.t1                 ---GNADEIENKFQPIL---DFDTDGCYNTAAIDPDGNI---N--PGKGAT--------------------G-TPQGDCRDPP---QLENS----NVYSRRRCNNGVCAIMYEYYFER--
    GF1207_g5214.t1                 ---GSAWSEQEKWCPAL---DYDTDSCYNTVAISPSGQL---N--AGQDENK-----------------PAG-EILGWCRKEV---HLQQT----NIYVRSRCNNGWCVHMYDYYFEADF
    GF1207_g6669.t1                 VPSGVSGQLMLKFKPYL----KVFNGCVPFPAVNAGGDT------------------------------------------------RRPR----HVYARAGSYNGANAILYAWYMPKDA
    GF1207_g6876.t1                 L--GPNGDMIRKFQPLL----HIAHGCQPYSAVNTRGEV---N--AGLQDS--------------------G-TTAGGCK------ETSKG----QTYARSMTLNGQFGIMYAWYWPKDQ
    GF1207_g6999.t1                 ---GRAPDLDKRFQPAL---DFDTDSCYNVPAIGPNGDL---A--IGMYPF--------------------EWPPQAGCRNEE---MLDRG----NVYSRQRCNNGYCVIFYAYYFQKDT
    GF1207_g7831.t1                 VQTGAIGDAIAKFNPLL----HIANGCQPYTAVNDAGDT---S--GGLQDS--------------------G-NISAGCR------DQSKG----QTYARAKVVNGQLAIMYSFYMPKDQ
    GF1300_g9576.t1                 ---GRAPDLDKRFQPAL---DFDTDSCYNVPAIGPNGDL---A--IGMYPF--------------------EWPPQAGCRNEE---MLDRG----NVYSRQRCNNGYCVIFYAYYFQKDT
    GF1300_g9714.t1                 L--GPNGDMIRKFQPLL----HIAHGCQPYSAVNTRGEV---N--AGLQDS--------------------G-TTAGGCK------ETSKG----QTYARSMTLNGQFGIMYAWYWPKDQ
    GF1300_g259.t1                  --HDFAYYFEVKFQPLV---DFDTDSCYSVPAMTMDGTA---S--EGLSPS--------------------D--DVGPCRPRS---ALDRT----NVYVRGRCNRGWCAFVYAYYFQMDW
    GF1300_g1512.t1                 ---GNADEIENKFQPIL---DFDTDGCYNTAAIDPDGNI---N--PGKGAT--------------------G-TPQGDCRDPP---QLENS----NVYSRRRCNNGVCAIMYEYYFEKDQ
    GF1300_g3582.t1                 ---GSAWSEQEKWCPAL---DYDTDSCYNTVAISPSGQL---N--AGQDENK-----------------PAG-EILGWCRKEV---HLQQT----NIYVRSRCNNGWCVHMYDYYFEADF
    GF1300_g3720.t1                 VPSGVSGQLMLKFKPYL----KVFNGCVPFPAVNAGGDT---G--GGLATS--------------------G-SSNGGC-------SSSAG----QVYARAGSYNGANAILYAWYMPKDA
    GF1300_g6171.t1                 VQTGAIGDAIAKFNPLL----HIANGCQPYTAVNDAGDT---S--GGLQDS--------------------G-NISAGCR------DQSKG----QTYARAKVVNGQLAIMYSFYMPKDQ
    Gf-Ca2_g111.t1                  ---GNADEIENKFQPIL---DFDTDGCYNTAAIDPDGNI---N--PGKGAT--------------------G-TPQGDCRDPP---QLENS----NVYSRRRCNNGVCAIMYEYYFER--
    Gf-Ca2_g2398.t1                 --HDFAYYFEVKFQPLV---DFDTDSCYSVPAMTMDGTA---S--EGLSPS--------------------D--DVGP----------------------GRCNRGWCAFVYAYYFQMDW
    Gf-Ca2_g3362.t1                 ---GRAPDLDKRFQPAL---DFDTDSCYNVPAIGPNGDL---A--IGMYPF--------------------EWPPQAGCRNEE---MLDRG----NVYSRQRCNNGYCVIFYAYYFQKDT
    Gf-Ca2_g3481.t1                 L--GPNGDMIRKFQPLL----HIAHGCQPYSAVNTRGEV---N--AGLQDS--------------------G-TTAGGCK------ETSKG----QTYARSMTLNGQFGIMYAWYWPKDQ
    Gf-Ca2_g3687.t1                 VPSGVSGQLMLKFKPYL----KVFNGCVPFPAVNAGGDT------------------------------------------------RRPR----HVYARAGSYNGANAILYAWYMPKDA
    Gf-Ca2_g4661.t1                 ---GSAWSEQEKWCPAL---DYDTDSCYNTVAISPSGQL---N--AGQDENK-----------------PAG-EILGWCRKEV---HLQQT----NIYVRSRCNNGWCVHMYDYYFEADF
    Gf-Ca2_g8346.t1                 VQTGAIGDAIAKFNPLL----HIANGCQPYTAVNDAGDT---S--GGLQDS--------------------G-NISAGCR------DQSKG----QTYARAKVVNGQLAIMYSFYMPKDQ
    Gf-Cb5_g260.t1                  --HDFAYYFEVKFQPLV---DFDTDSCYSVPAMTMDGTA---S--EGLSPS--------------------D--DVGPCRPRS---ALDRT----NVYVRGRCNRGWCAFVYAYYFQMDW
    Gf-Cb5_g3949.t1                 L--GPNGDMIRKFQPLL----HIAHGCQPYSAVNTRGEV---N--AGLQDS--------------------G-TTAGGCK------ETSKG----QTYARSMTLNGQFGIMYAWYWPKDQ
    Gf-Cb5_g4084.t1                 ---GRAPDLDKRFQPAL---DFDTDSCYNVPAIGPNGDL---A--IGMYPF--------------------EWPPQAGCRNEE---MLDRG----NVYSRQRCNNGYCVIFYAYYFQKDT
    Gf-Cb5_g4605.t1                 VQTGAIGDAIAKFNPLL----HIANGCQPYTAVNDAGDT---S--GGLQDS--------------------G-NISAGCR------DQSKG----QTYARAKVVNGQLAIMYSFYMPKDQ
    Gf-Cb5_g6249.t1                 ---KRATENDLRYQPAL---DFDTDSCYNVPAIGCDGKI---A--EGLEPD--------------------G--TTKDYRDLA---DLDNT----NVYSRQRCNSGWCAYMYDYYFEKDH
    Gf-Cb5_g7167.t1                 ---GNADEIENKFQPIL---DFDTDGCYNTAAIDPDGNI---N--PGKGAT--------------------G-TPQGDCRDPP---QLENS----NVYSRRRCNNGVCAIMYEYYFEKDQ
    Gf-Cb5_g7934.t1                 VPSGVSGQLMLKFKPYL----KVFNGCVPFPAVNAGGDT---G--GGLATS--------------------G-SSNGGC-------SSSAG----QVYARAGSYNGANAILYAWYMPKDA
    Gf-Cb5_g8589.t1                 ---GSAWSEQEKWCPAL---DYDTDSCYNTVAISPSGQL---N--AGQDENK-----------------PAG-EILGWCRKEV---HLQQT----NIYVRSRCNNGWCVHMYDYYFEADF
    HoMCF_g1490.t1                  -------------------------------SAARHGQL---N--AGQDENK-----------------PAG-EILGWCRKEV---HLQQT----NIYVRSRCNNGWCVHMYD-YFEADF
    HoMCF_g2875.t1                  VQTGAIGDAIAKFNPLL----HIANGCQPYTAVNDAGDT---S--G------------------------------------------VKG----QTYARAKVVNGQLAIMYSFYMPKDQ
    HoMCF_g3274.t1                  ----------RKFQPLL----HIAHGCQPYSAVNTRGEV---N--AGLQDS--------------------G-TTAGGCK------ETSKG----QTYARSMTLNGQFGII---------
    HoMCF_g4303.t1                  --HDFAYYFEVKFQPLV---DFDTDSCYSVPAMTMDGTA---S--EGLSPS--------------------D--DVGP----------------------GRCNRGWCAFVYAYYFQMDW
    HoMCF_g5967.t1                  ---GRAPDLDKRFQPAL---DFDTDSCYNVPAIGPNGDL---A--IGMYPF--------------------EWPPQAGCRNEE---MLDRG----N---------------------KDT
    HoMCF_g6093.t1                  ----------RKFQPLL----HIAH-------------V---N--A---VS--------------------K-TAVPPQA------AARKP----ATYARSMTLNGQFGIMYAWYWPKDQ
    HoMCF_g6096.t1                  ----------RKFQPLL----HIAHGCQPYSAVNTRGEV---N--AGLQDS--------------------G-TTAGGCK------ETSKG----QTYARSMTLNGQFGII---------
    HoMCF_g6549.t1                  VPSGVSGQLMLKFKPYL----KVFNGCVPFPAVNAGGDT---GEHGGLATS--------------------G-SSNGG-------------------------YNGANAILYAWYMPKDA
    HoMCF_g10173.t1                 ---GNADEIENKFQPIL---DFDTDGCYNTAAIDPDGNI---N--PGKGAT--------------------G-TPQGDCRDP--------------LSSRTAMFTPVAAATTASALSCT-
    HoMCLT_g1190.t1                 --HDFAYYFEVKFQPLV---DFDTDSCYSVPAMTMDGTA---S--EGLSPS--------------------D--DVGPCRPRS---ALDRT----NVYVRGRCNRGWCAFVYAYYFQMDW
    HoMCLT_g2195.t1                 VQTGAIGDAIAKFNPLL----HIANGCQPYTAVNDAGDT---S--GGLQDS--------------------G-NISAGCR------DQSKG----QTYARAKVVNGQLAIMYSFYMPKDQ
    HoMCLT_g2713.t1                 ---GSAWSEQEKWCPAL---DYDTDSCYNTVAISPSGQL---N--AGQDENK-----------------PAG-EILGWCRKEV---HLQQT----NIYVRSRCNNGWCVHMYDYYFEADF
    HoMCLT_g3698.t1                 VPSGVSGQLMLKFKPYL----KVFNGCVPFPAVNAGGDT---G--GGLATS--------------------G-SSNGGC-------SSSAG----QVYARAGSYNGANAILYAWYMPKDA
    HoMCLT_g4981.t1                 ---GNADEIENKFQPIL---DFDTDGCYNTAAIDPDGNI---N--PGKGAT--------------------G-TPQGDCRDPP---QLENS----NVYSRRRCNNGVCAIMYEYYFEKDQ
    HoMCLT_g6335.t1                 L--GPNGDMIRKFQPLL----HIAHGCQPYSAVNTRGEV---N--AGLQDS--------------------G-TTAGGCK------ETSKG----QTYARSMTLNGQFGIMYAWYWPKDQ
    HoMCLT_g6471.t1                 ---GRAPDLDKRFQPAL---DFDTDSCYNVPAIGPNGDL---A--IGMYPF--------------------EWPPQAGCRNEE---MLDRG----NVYSRQRCNNGYCVIFYAYYFQKDT
    I1V_g7945.t1                    VQTGAIGDAIAKFNPLL----HIANGCQPYTAVNDAGDT---S--GGLQD--------------------------------------IKG----QTYARAKVVNGQLAIMYSFYMPKDQ
    I1V_g9550.t1                    ---------------------------SPYSAVNTRGEV---N--AGLQDS--------------------G-TTAGGCK------ETSKG----QTYARSMTLNGQFGIMYAWYWPKDQ
    I1V_g9695.t1                    --HDFAYYFEVKFQPLV---DFDTDSCYSVPAMTMDGTA---S--EGLSPS--------------------D--DVGP----------------------GRCNRGWCAFVYAYYFQMDW
    I1V_g10783.t1                   VPSGVSGQLMLKFKPYL----KVFNGCVPFPAVNAGGDT---G--GGLATS--------------------G-SSNGGC-------SSSAG----Q----------------------DA
    I1V_g12440.t1                   -----------------------------------------------MYPF--------------------DTLP---------------------------------------------
    I1V_g13776.t1                   ---GSAWSEQEKWCPAL---DYDTDSCYNTVAISPSGQL---N--AGQDENK-----------------PAG-EILGWCRKEV---HLQQT----NIYVRSRCNNGWCVHMYDYYFEADF
    MPI-CAGE_g5591.t1               ---GNADEIENKFQPIL---DFDTDGCYNTAAIDPDGNI---N--PGKGAT--------------------G-TPQGDCRDPP---QLENS----NVYSRRRCNNGVCAIMYEYYFEKDQ
    MPI-CAGE_g8268.t1               L--GPNGDMIRKFQPLL----HIAHGCQPYSAVNTRGEV---N--AGLQDS--------------------G-TTAGGCK------ETSKG----QTYARSMTLNGQFGIMYAWYWPKDQ
    MPI-CAGE_g8414.t1               ---GRAPDLDKRFQPAL---DFDTDSCYNVPAIGPNGDL---A--IGMYPF--------------------EWPPQAGCRNEE---MLDRG----NVYSRQRCNNGYCVIFYAYYFQKDT
    MPI-CAGE_g9956.t1               VPSGVSGQLMLKFKPYL----KVFNGCVPFPAVNAGGDT---G--GGLATS--------------------G-SSNGGC-------SSSAG----QVYARAGSYNGANAILYAWYMPKDA
    MPI-CAGE_g1577.t1               --HDFAYYFEVKFQPLV---DFDTDSCYSVPAMTMDGTA---S--EGLSPS--------------------D--DVGP----------------------GRCNRGWCAFVYAYYFQMDW
    MPI-CAGE_g2204.t1               ---KRATENDLRYQPAL---DFDTDSCYNVPAIGCDGKI---A--EGLEPD--------------------G--TTKDYRDLA---DLDNT----NVYSRQRCNSGWCAYMYDYYFEKDH
    MPI-CAGE_g10572.t1              ---GRAPDLDKRFQPAL---DFDTDSCYNVPAIGPNGDL---A--IGMYPF--------------------EWPPQAGCRNEE---MLDRG----NVYSRQRCNNGYCVIFYAYYFQKDT
    MPI-CAGE_g3967.t1               ---GSAWSEQEKWCPAL---DYDTDSCYNTVAISPSGQL---N--AGQDENK-----------------PAG-EILGWCRKEV---HLQQT----NIYVRSRCNNGWCVHMYDYYFEADF
    MPI-CAGE_g4142.t1               VQTGAIGDAIAKFNPLL----HIANGCQPYTAVNDAGDT---S--GGLQDS--------------------G-NISAGCR------DQSKG----QTYARAKVVNGQLAIMYSFYMPKDQ
    S011_g7486.t1                   ---KRATENDLRYQPAL---DFDTDSCYNVPAIGCDGKI---A--EGLEPD--------------------G--TTKDYRDLA---DLDNT----NVYSRQRCNSGWCAYMYDYYFEKDH
    S011_g8092.t1                   VQTGAIGDAIAKFNPLL----HIANGCQPYTAVNDAGDT---S--GGLQDS--------------------G-NISAGCR------DQSKG----QTYARAKVVNGQLAIMYSFYMPKDQ
    S011_g8657.t1                   ---GRAPDLDKRFQPAL---DFDTDSCYNVPAIGPNGDL---A--IGMYPF--------------------EWPPQAGCRNEE---MLDRG----NVYSRQRCNNGYCVIFYAYYFQKDT
    S011_g8986.t1                   VPSGVSGQLMLKFKPYL----KVFNGCVPFPAVNAGGDT---G--GGLATS--------------------G-SSNGGC-------SSSAG----QVYARAGSYNGANAILYAWYMPKDA
    S011_g9203.t1                   L--GPNGDMIRKFQPLL----HIAHGCQPYSAVNTRGEV---N--AGLQDS--------------------G-TTAGGCK------ETSKG----QTYARSMTLNGQFGIMYAWYWPKDQ
    S011_g1207.t1                   --HDFAYYFEVKFQPLV---DFDTDSCYSVPAMTMDGTA---S--EGLSPS--------------------D--DVGPCRPRS---ALDRT----NVYVRGRCNRGWCAFVYAYYFQMDW
    S011_g2838.t1                   ---GNADEIENKFQPIL---DFDTDGCYNTAAIDPDGNI---N--PGKGAT--------------------G-TPQGDCRDPP---QLENS----NVYSRRRCNNGVCAIMYEYYFEKDQ
    S011_g5817.t1                   ---GSAWSEQEKWCPAL---DYDTDSCYNTVAISPSGQL---N--AGQDENK-----------------PAG-EILGWCRKEV---HLQQT----NIYVRSRCNNGWCVHMYDYYFEADF
    S023_g1908.t1                   --HDFAYYFEVKFQPLV---DFDTDSCYSVPAMTMDGTA---S--EGLSPS--------------------D--DVGPCRPRS---ALDRT----NVYVRGRCNRGWCAFVYAYYFQMDW
    S023_g3148.t1                   VPSGVSGQLMLKFKPYL----KVFNGCVPFPAVNAGGDT---G--GGLATS--------------------G-SSNGGC-------SSSAG----QVYARAGSYNGANAILYAWYMPKDA
    S023_g4559.t1                   ---GNADEIENKFQPIL---DFDTDGCYNTAAIDPDGNI---N--PGKGAT--------------------G-TPQGDCRDPP---QLENS----NVYSRRRCNNGVCAIMYEYYFEKDQ
    S023_g5760.t1                   ---GRAPDLDKRFQPAL---DFDTDSCYNVPAIGPNGDL---A--IGMYPF--------------------EWPPQAGCRNEE---MLDRG----NVYSRQRCNNGYCVIFYAYYFQKDT
    S023_g5904.t1                   L--GPNGDMIRKFQPLL----HIAHGCQPYSAVNTRGEV---N--AGLQDS--------------------G-TTAGGCK------ETSKG----QTYARSMTLNGQFGIMYAWYWPKDQ
    S023_g7666.t1                   ---GSAWSEQEKWCPAL---DYDTDSCYNTVAISPSGQL---N--AGQDENK-----------------PAG-EILGWCRKEV---HLQQT----NIYVRSRCNNGWCVHMYDYYFEADF
    S023_g9104.t1                   VQTGAIGDAIAKFNPLL----HIANGCQPYTAVNDAGDT---S--GGLQDS--------------------G-NISAGCR------DQSKG----QTYARAKVVNGQLAIMYSFYMPKDQ
    TO22_g2077.t1                   --HDFAYYFEVKFQPLV---DFDTDSCYSVPAMTMDGTA---S--EGLSPS--------------------D--DVGPCRPRS---ALDRT----NVYVRGRCNRGWCAFVYAYYFQMDW
    TO22_g2405.t1                   ---GRAPDLDKRFQPAL---DFDTDSCYNVPAIGPNGDL---A--IGMYPF--------------------EWPPQAGCRNEE---MLDRG----NVYSRQRCNNGYCVIFYAYYFQKDT
    TO22_g2541.t1                   L--GPNGDMIRKFQPLL----HIAHGCQPYSAVNTRGEV---N--AGLQDS--------------------G-TTAGGCK------ETSKG----QTYARSMTLNGQFGIMYAWYWPKDQ
    TO22_g2749.t1                   VPSGVSGQLMLKFKPYL----KVFNGCVPFPAVNAGGDT---G--GGLATS--------------------G-SSNGGC-------SSSAG----QVYARAGSYNGANAILYAWYMPKDA
    TO22_g3776.t1                   ---GNADEIENKFQPIL---DFDTDGCYNTAAIDPDGNI---N--PGKGAT--------------------G-TPQGDCRDPP---QLENS----NVYSRRRCNNGVCAIMYEYYFEKDQ
    TO22_g6379.t1                   VQTGAIGDAIAKFNPLL----HIANGCQPYTAVNDAGDT---S--GGLQDS--------------------G-NISAGCR------DQSKG----QTYARAKVVNGQLAIMYSFYMPKDQ
    TO22_g9457.t1                   ---GSAWSEQEKWCPAL---DYDTDSCYNTVAISPSGQL---N--AGQDENK-----------------PAG-EILGWCRKEV---HLQQT----NIYVRSRCNNGWCVHMYDYYFEADF
    Ud1-4-1_g9889.t1                ---GSAWSEQEKWCPAL---DYDTDSCYNTVAISPSGQL---N--AGQDENK-----------------PAG-EILGWCRKEV---HLQQT----NIYVRSRCNNGWCVHMYDYYFEADF
    Ud1-4-1_g261.t1                 --HDFAYYFEVKFQPLV---DFDTDSCYSVPAMTMDGTA---S--EGLSPS--------------------D--DVGPCRPRS---ALDRT----NVYVRGRCNRGWCAFVYAYYFQMDW
    Ud1-4-1_g2536.t1                ---GNADEIENKFQPIL---DFDTDGCYNTAAIDPDGNI---N--PGKGAT--------------------G-TPQGDCRDPP---QLENS----NVYSRRRCNNGVCAIMYEYYFEKDQ
    Ud1-4-1_g2721.t1                ---GRAPDLDKRFQPAL---DFDTDSCYNVPAIGPNGDL---A--IGMYPF--------------------EWPPQAGCRNEE---MLDRG----NVYSRQRCNNGYCVIFYAYYFQKDT
    Ud1-4-1_g2858.t1                L--GPNGDMIRKFQPLL----HIAHGCQPYSAVNTRGEV---N--AGLQDS--------------------G-TTAGGCK------ETSKG----QTYARSMTLNGQFGIMYAWYWPKDQ
    Ud1-4-1_g3064.t1                VPSGVSGQLMLKFKPYL----KVFNGCVPFPAVNAGGDT---G--GGLATS--------------------G-SSNGGC-------SSSAG----QVYARAGSYNGANAILYAWYMPKDA
    Ud1-4-1_g3975.t1                ---KRATENDLRYQPAL---DFDTDSCYNVPAIGCDGKI---A--EGLEPD--------------------G--TTKDYRDLA---DLDNT----NVYSRQRCNSGWCAYMYDYYFEKDH
    Ud1-4-1_g4565.t1                VQTGAIGDAIAKFNPLL----HIANGCQPYTAVNDAGDT---S--GGLQDS--------------------G-NISAGCR------DQSKG----QTYARAKVVNGQLAIMYSFYMPKDQ
    V13_g3278.t1                    ---GRAPDLDKRFQPAL---DFDTDSCYNVPAIGPNGDL---A--IGMYPF--------------------EWPPQAGCRNEE---MLDRG----NVYP---------------------
    V13_g3397.t1                    ----------RKFQPLL----HIAHGCQPYSAVNTRGEV---N--AGLQDS--------------------A-RRRQ---------------------------------------PRQR
    V13_g4590.t1                    VQTGAIGDAIAKFNPLL----HIANGCQPYTAVNDAGDT---S--GGLQDS--------------------G-NISAGAGTSPG-PDVRPG----QGGQRAA---GHHVLVLHAQGPADR
    V13_g5067.t1                    ---GNADEIENKFQPIL---DFDTDGCYNTAAIDPDGNI---N--PGKAPP--------------------A-LLRATAAIPS---AREQQCLLPSPLQQRRLRYHV-------------
    V13_g6708.t1                    ---GSAWSEQEKWCPAL---DYDTDSCYNTVAISPSGQL---S--AGQDENK-----------------PAG-EILGWCRKEV---HLQQT----NIYVRSRCNNGWCVHMYDYYFEADF
    V13_g9344.t1                    ------AKNDLRYQPAL---DFDTDSCYNVPAIGCDGKI---A--EGLEPD--------------------G--TTKDYRDLA---DLDNT----NVYSRQRCNSGWCAYMYDYYFEKDH
    V13_g9739.t1                    VPSGVSGQLMLKFKPYL----KVFNGCVPFPAVNAGGDT---A--AASPRR--------------------A-AATAGA-------LRARA----RC-TRAGSYNGANAILYAWYMPKDA
    V13_g10958.t1                   --HDFAYYFEVKFQPLV---DFDTDSCYSVPAMTMDGTA---S--EGLSPS--------------------D--DVGP----------------------GRCNRGWCAFVYAYYFQMDW
    Vd39_g2112.t1                   ---KRATENDLRYQPAL---DFDTDSCYNVPAIGCDGKI---A--EGLEPD--------------------G--TTKDYRDLA---DLDNT----NVYSRQRCNSGWCAYMYDYYFEKDH
    Vd39_g2112.t3                   ---KRATENDLRYQPAL---DFDTDSCYNVPAIGCDGKI---A--EGLEPD--------------------G--TTKDYRDLA---DLDNT----NVYSRQRCNSGWCAYMYDYYFEKDH
    Vd39_g2716.t1                   --HDFAYYFEVKFQPLV---DFDTDSCYSVPAMTMDGTA---S--EGLSPS--------------------D--DVGPCRPRS---ALDRT----NVYVRGRCNRGWCAFVYAYYFQMDW
    Vd39_g3506.t1                   ---GRAPDLDKRFQPAL---DFDTDSCYNVPAIGPNGDL---A--IGMYPF--------------------EWPPQAGCRNEE---MLDRG----NVYSRQRCNNGYCVIFYAYYFQKDT
    Vd39_g3907.t1                   ---GSAWSEQEKWCPAL---DYDTDSCYNTVAISPSGQL---N--AGQDENK-----------------PAG-EILGWCRKEV---HLQQT----NIYVRSRCNNGWCVHMYDYYFEADF
    Vd39_g4379.t1                   ---GNADEIENKFQPIL---DFDTDGCYNTAAIDPDGNI---N--PGKGAT--------------------G-TPQGDCRDPP---QLENS----NVYSRRRCNNGVCAIMYEYYFEKDQ
    Vd39_g7329.t1                   VQTGAIGDAIAKFNPLL----HIANGCQPYTAVNDAGDT---S--GGLQDS--------------------G-NISAGCR------DQSKG----QTYARAKVVNGQLAIMYSFYMPKDQ
    Vd39_g8964.t1                   VPSGVSGQLMLKFKPYL----KVFNGCVPFPAVNAGGDT---G--GGLATS--------------------G-SSNGGC-------SSSAG----QVYARAGSYNGANAILYAWYMPKDA
    Vd39_g9298.t1                   L--GPNGDMIRKFQPLL----HIAHGCQPYSAVNTRGEV---N--AGLQDS--------------------G-TTAGGCK------ETSKG----QTYARSMTLNGQFGIMYAWYWPKDQ
    Vd-653_g1291.t1                 ----------RKFQPLL----HIAHGCQPYSAVNTRGEV---N--AGLQDS--------------------G-TTAGGCK------ETSKG----QTYARSMTLNGQFGIMYAWYWPKDQ
    Vd-653_g2557.t1                 --HDFAYYFEVKFQPLRARHDHGRNRI------------------RGPFAL--------------------R--RRRPCRPRS---ALDRT----NVYVRGRCNRGWCAFVYAYYFQMDW
    Vd-653_g3339.t1                 -----GMVEQEKWCPAL---DYDTDSCYNTVAISPSGQL---N--AGQDENK-----------------PAG-EILGWCRKEV---RLQQT----NIYVRSRCNNGWCVHMYDYYFEADF
    Vd-653_g3685.t1                 ------------------------------------------------------------------------------------------------------------------------
    Vd-653_g3686.t1                 --------------PYL----KVFNGCVPFPAVNAGGDT---R--RPRHV---------------------G-SSNGGC-------SSSAG----Q----------------------DA
    Vd-653_g5777.t1                 -----------RFQPAL---DFDTDSCYNVPAIGPNGDL---A--IGMYPF--------------------EWPPQAGCRNEE---MLDRG----NVYSRQRCNNGYCVIFYAYYFQKDT
    Vd-653_g8550.t1                 ------------FNPLL----HIANGCQPYTAVNDAGDT---S--GGLQDS--------------------G-NIS-GVQ------GPVQG----QTYARAKVVNGQLAIMYSFYMPKDQ
    Vd-653_g9566.t1                 ---GNADEIENKFQPIL---DFDTDGCYNTAAIDPDGNI---N--PGKGAT--------------------G-TPQGDCRDP--------------LSSRTAMFTPVAAATTASALSCT-
    VD991_g2070.t1                  --HDFAYYFEVKFQPLV---DFDTDSCYSVPAMTMDGTA---S--EGLSPS--------------------D--DVGPCRPRS---ALDRT----NVYVRGRCNRGWCAFVYAYYFQMDW
    VD991_g2394.t1                  ---GRAPDLDKRFQPAL---DFDTDSCYNVPAIGPNGDL---A--IGMYPF--------------------EWPPQAGCRNEE---MLDRG----NVYSRQRCNNGYCVIFYAYYFQKDT
    VD991_g2531.t1                  L--GPNGDMIRKFQPLL----HIAHGCQPYSAVNTRGEV---N--AGLQDS--------------------G-TTAGGCK------ETSKG----QTYARSMTLNGQFGIMYAWYWPKDQ
    VD991_g2742.t1                  VPSGVSGQLMLKFKPYL----KVFNGCVPFPAVNAGGDT---G--GGLATS--------------------G-SSNGGC-------SSSAG----QVYARAGSYNGANAILYAWYMPKDA
    VD991_g3653.t1                  ---KRATENDLRYQPAL---DFDTDSCYNVPAIGCDGKI---A--EGLEPD--------------------G--TTKDYRDLA---DLDNT----NVYSRQRCNSGWCAYMYDYYFEKDH
    VD991_g3653.t2                  ---KRATENDLRYQPAL---DFDTDSCYNVPAIGCDGKI---A--EGLEPD--------------------G--TTKDYRDLA---DLDNT----NVYSRQRCNSGWCAYMYDYYFEKDH
    VD991_g3719.t1                  ---GNADEIENKFQPIL---DFDTDGCYNTAAIDPDGNI---N--PGKGAT--------------------G-TPQGDCRDPP---QLENS----NVYSRRRCNNGVCAIMYEYYFEKDQ
    VD991_g7479.t1                  VQTGAIGDAIAKFNPLL----HIANGCQPYTAVNDAGDT---S--GGLQDS--------------------G-NISAGCR------DQSKG----QTYARAKVVNGQLAIMYSFYMPKDQ
    VD991_g8969.t1                  ---GSAWSEQEKWCPAL---DYDTDSCYNTVAISPSGQL---N--AGQDENK-----------------PAG-EILGWCRKEV---HLQQT----NIYVRSRCNNGWCVHMYDYYFEADF
    VdB09_g1055.t1                  ---GRAPDLDKRFQPAL---DFDTDSCYNVPAIGPNGDL---A--IGMYPF--------------------EWPPQAGCRNEE---MLDRG----NVYSRQRCNNGYCVIFYAYYFQKDT
    VdB09_g1685.t1                  ---GSAWSEQEKWCPAL---DYDTDSCYNTVAISPSGQL---N--AGQDENK-----------------PAG-EILGWCRKEV---HLQQT----NIYVRSRCNNGWCVHMYDYYFEADF
    VdB09_g1751.t1                  ---GNADEIENKFQPIL---DFDTDGCYNTAAIDPDGNI---N--PGKGAT--------------------G-TPQGDCRDP--------------LSSRTAMFTPVAAATTASALSCT-
    VdB09_g3918.t1                  ---KRATENDLRYQPAL---DFDTDSCYNVPAIGCDGKI---A--EGLEPD--------------------G--TTKDYRDLA---DLDNT----NVYSRQRCNSGWCAYMYDYYFEKDH
    VdB09_g4877.t1                  --------------PYL----KVFNGCVPFPAVNAGGDT---G--GGLATS--------------------G-SSNGGC-------SSSAG----QVYARAGSYNGANAILYAWYMPKDA
    VdB09_g5096.t1                  L--GPNGDMIRKFQPLL----HIAHGCQPYSAVNTRGEV---N--AGLQDS--------------------G-TTAGGCK------ETSKG----QTYARSMTLNGQFGIMYAWYWPKDQ
    VdB09_g9703.t1                  VQTGAIGDAIAKFNPLL----HIANGCQPYTAVNDAGDT---S--GGLQDS--------------------G-NISAGCR------DQSKG----QTYARAKVVNGQLAIMYSFYMPKDQ
    VdB09_g7661.t1                  --HDFAYYFEVKFQPLV---DFDTDSCYSVPAMTMDGTA---S--EGLSPS--------------------D--DVGPCRPRS---ALDRT----NVYVRGRCNRGWCAFVYAYYFQMDW
    VdC07_g4126.t1                  ---GRAPDLDKRFQPAL---DFDTDSCYNVPAIGPNGDL---A--IGMYPF--------------------EWPPQAGCRNEE---MLDRG----NVYSRQRCNNGYCVIFYAYYFQKDT
    VdC07_g4261.t1                  L--GPNGDMIRKFQPLL----HIAHGCQPYSAVNTRGEV---N--AGLQDS--------------------G-TTAGGCK------ETSKG----QTYARSMTLNGQFGIMYAWYWPKDQ
    VdC07_g4482.t1                  VPSGVSGQLMLKFKPYL----KVFNGCVPFPAVNAGGDT---G--GGLATS--------------------G-SSNGGC-------SSSAG----QVYARAGSYNGANAILYAWYMPKDA
    VdC07_g605.t1                   VQTGAIGDAIAKFNPLL----HIANGCQPYTAVNDAGDT---S--GGLQDS--------------------G-NISAGCR------DQSKG----QTYARAKVVNGQLAIMYSFYMPKDQ
    VdC07_g1872.t1                  ---GSAWSEQEKWCPAL---DYDTDSCYNTVAISPSGQL---N--AGQDENK-----------------PAG-EILGWCRKEV---HLQQT----NIYVRSRCNNGWCVHMYDYYFEADF
    VdC07_g2986.t1                  ---GNADEIENKFQPIL---DFDTDGCYNTAAIDPDGNI---N--PGKGAT--------------------G-TPQGDCRDPP---QLENS----NVYSRRRCNNGVCAIMYEYYFEKDQ
    VdC07_g7672.t1                  --HDFAYYFEVKFQPLV---DFDTDSCYSVPAMTMDGTA---S--EGLSPS--------------------D--DVGPCRPRS---ALDRT----NVYVRGRCNRGWCAFVYAYYFQMDW
    Vd-H5_g2477.t1                  VPSGVSGQLMLKFKPYL----KVFNGCVPFPAVNAGGDT---GEHGGLATS--------------------G-SSNGGC-------SSSAG----QVYARAGSYNGANAILYAWYMPKDA
    Vd-H5_g5176.t1                  ---GSAWSEQEKWCPAL---DYDTDSCYNT------------------DENK-----------------PAG-EILGWCRKEV---RLQQT----NIYVRSRCNNGWCVHMYDYYFEADF
    Vd-H5_g6144.t1                  ---GNADEIENKFQPIL---DFDTDGCYNTAAIDPDGNI---N--PGKGAT--------------------G-TPQGDCRDP--------------LSSRTAMFTPVAAATTASALSCT-
    Vd-H5_g7203.t1                  VQTGAIGDAIAKFNPLL----HIANGCQPYTAVNDAGDT---S--G--------------------------------------------G----QTYARAKVVNGQLAIMYSFYMPKDQ
    Vd-H5_g8423.t1                  ---GRAPDLDKRFQPAL---DFDTDSCYNVPAIGPNGDL---A--IGMYPF--------------------EWPPQAGCRNEE---MLDRG----NVYSRQRCNNGYCVIFYAYYFQKDT
    Vd-H5_g8546.t1                  L--GPNGDMIRKFQPLL----HIAHGCQPYSAVNTRGEV---N--AGLQDS--------------------G-TTAGGCK------ETSKG----QTYARSMTLNGQFGIMYAWYWPKDQ
    Vd-H5_g9396.t1                  --HDFAYYFEVKFQPLV---DFDTDSCYSVPAMTMDGTA---S--EGLSPS--------------------D--DVGP----------------------GRCNRGWCAFVYAYYFQMDW
    VdLs16_g2071.t1                 --HDFAYYFEVKFQPLV---DFDTDSCYSVPAMTMDGTA---S--EGLSPS--------------------D--DVGPCRPRS---ALDRT----NVYVRGRCNRGWCAFVYAYYFQMDW
    VdLs16_g7836.t1                 VQTGAIGDAIAKFNPLL----HIANGCQPYTAVNDAGDT---S--GGLQDS--------------------G-NISAGCR------DQSKG----QTYARAKVVNGQLAIMYSFYMPKDQ
    VdLs16_g8455.t1                 ---GNADEIENKFQPIL---DFDTDGCYNTAAIDPDGNI---N--PGKGAT--------------------G-TPQGDCRDPP---QLENS----NVYSRRRCNNGVCAIMYEYYFEKDQ
    VdLs16_g5349.t1                 VPSGVSGQLMLKFKPYL----KVFNGCVPFPAVNAGGDT---G--GGLATS--------------------G-SSNGGC-------SSSAG----QVYARAGSYNGANAILYAWYMPKDA
    VdLs16_g5560.t1                 L--GPNGDMIRKFQPLL----HIAHGCQPYSAVNTRGEV---N--AGLQDS--------------------G-TTAGGCK------ETSKG----QTYARSMTLNGQFGIMYAWYWPKDQ
    VdLs16_g5699.t1                 ---GRAPDLDKRFQPAL---DFDTDSCYNVPAIGPNGDL---A--IGMYPF--------------------EWPPQAGCRNEE---MLDRG----NVYSRQRCNNGYCVIFYAYYFQKDT
    VdLs16_g6952.t1                 ---GSAWSEQEKWCPAL---DYDTDSCYNTVAISPSGQL---N--AGQDENK-----------------PAG-EILGWCRKEV---HLQQT----NIYVRSRCNNGWCVHMYDYYFEADF
    VdLs17_g64.t1                   ---GNADEIENKFQPIL---DFDTDGCYNTAAIDPDGNI---N--PGKGAT--------------------G-TPQGDCRDPP---QLENS----NVYSRRRCNNGVCAIMYEYYFEKDQ
    VdLs17_g1975.t1                 --HDFAYYFEVKFQPLV---DFDTDSCYSVPAMTMDGTA---S--EGLSPS--------------------D--DVGPCRPRS---ALDRT----NVYVRGRCNRGWCAFVYAYYFQMDW
    VdLs17_g3499.t1                 ---KRATENDLRYQPAL---DFDTDSCYNVPAIGCDGKI---A--EGLEPD--------------------G--TTKDYRDLA---DLDNT----NVYSRQRCNSGWCAYMYDYYFEKDH
    VdLs17_g4253.t1                 --------------------------CRFMAPLADLVPT---Y--GFL------------------------------------------A----PLTDGAPRRYTQLSII---------
    VdLs17_g4391.t1                 L--GPNGDMIRKFQPLL----HIAHGCQPYSAVNTRGEV---N--AGLQDS--------------------G-TTAGGCK------ETSKG----QTYARSMTLNGQFGIMYAWYWPKDQ
    VdLs17_g5246.t1                 ---GRAPDLDKRFQPAL---DFDTDSCYNVPAIGPNGDL---A--IGMYPF--------------------EWPPQAGCRNEE---MLDRG----NVYSRQRCNNGYCVIFYAYYFQKDT
    VdLs17_g6770.t1                 VQTGAIGDAIAKFNPLL----HIANGCQPYTAVNDAGDT---S--GGLQDS--------------------G-NISAGCR------DQSKG----QTYARAKVVNGQLAIMYSFYMPKDQ
    VdLs17_g8273.t1                 ---GSAWSEQEKWCPAL---DYDTDSCYNTVAISPSGQL---N--AGQDENK-----------------PAG-EILGWCRKEV---HLQQT----NIYVRSRCNNGWCVHMYDYYFEADF
    VdLs17_g8809.t1                 VPSGVSGQLMLKFKPYL----KVFNGCVPFPAVNAGGDT---G--GGLATS--------------------G-SSNGGC-------SSSAG----QVYARAGSYNGANAILYAWYMPKDA
    Vd-R1_g926.t1                   ------------FKPYL----KVFNGCVPFPAVNAGGDT---GEHGGLATS--------------------G-SSNGGC-------SSSAG----QVYARAGSYNGANAILYAWYMPKDA
    Vd-R1_g3679.t1                  ---GNADEIENKFQPIL---DFDTDGCYNTAAIDPDGNI---N--PGKGAT--------------------G-TPQGDCRDP--------------LSSRTAMFTPVAAATTASALSCT-
    Vd-R1_g4718.t1                  VQTGAIGDAIAKFNPLL----HIA---QPYTAVNDAGDT---S--GGLQD--------------------------------------IQG----QTYARAKVVNGQLAIMYSFYMPKDQ
    Vd-R1_g5037.t1                  ---GSAWSEQEKWCPAL---DYDTDSCYNT--------L---N--AGQDENK-----------------PAG-EILGWCRKEV---RLQQT----NIYVRSRCNNGWCVHMYDYYFEADF
    Vd-R1_g8886.t1                  --HDFAYYFEVKFQPLV---DFDTDSCYSVPAMTMDGTA---S--EGLSPS--------------------D--DVGPCRPRS---ALDRT----NVYVRGRCNRGWCAFVYAYYFQMDW
    Vd-R1_g9491.t1                  ----------RKFQPLL----HIAHGCQPYSAVNTRGEV---N--AGLQDS--------------------G-TTAGGCK------ETSKG----QTYARSMTLNGQFGII---------
    Vd-R1_g9617.t1                  ---GRAPDLDKRFQPAL---DFDTDSCYNVPAIGPNGDL---A--IGMYPF--------------------EWPPQAGCRNEE---MLDRG----NVYSRQRCNNGYCVIFYAYYFQKDT
    VT-2A_g1161.t1                  ---GNADEIENKFQPIL---DFDTDGCYNTAAIDPDGNI---N--PGKGAT--------------------G-TPQGDCRDP--------------LSSRTAMFTPVAAATTASALSCT-
    VT-2A_g1267.t1                  ---GSAWSEQEKWCPAL---DYDTDSCYNTVAISPSGQL---N--AGQDENK-----------------PAG-EILGWCRKEV---HLQQT----NIYVRSRCNNGWCVHMYDYYFEADF
    VT-2A_g4726.t1                  VQTGAIGDAIAKFNPLL----HIANGCQPYTAVNDAGDT---S--GGLQDS--------------------G-NISAGCR------DQSKG----QTYARAKVVNGQLAIMYSFYMPKDQ
    VT-2A_g6128.t1                  VPSGVSGQLMLKFKPYL----KVFNGCVPFPAVNAGGDT---GEHGGLA-----------------------------------------------------TYNGANAILYAWYMPKDA
    VT-2A_g6128.t2                  VPSGVSGQLMLKFKPYL----KVFNGCVPFPAVNAGGDT---G--EQLASSVRPRFLSVLTTFAQRRPRHVG-QQQRRV-------LVERG----PGVRARGSYNGANAILYAWYMPKDA
    VT-2A_g6341.t1                  L--GPNGDMIRKFQPLL----HIAHGCQPYSAVNTRGEV---N--AGLQDS--------------------G-TTAGGCK------ETSKG----QTYARSMTLNGQFGIMYAWYWPKDQ
    VT-2A_g6470.t1                  ---GRAPDLDKRFQPAL---DFDTDSCYNVPAIGPNGDL---A--IGMYPF--------------------EWPPQAGCRNEE---MLDRG----NVYSRQRCNNGYCVIFYAYYFQKDT
    VT-2A_g9510.t1                  ------------------------------------------------------------------------------------------------------------------------
    VT-2A_g9511.t1                  --HDFAYYFEVKFQPLV---DFDTDSCYSVPAMTMDGTA---S--EGLSPS--------------------D--DVGP----------------------GRCNRGWCAFVYAYYFQMDW
    XJ511_g1085.t1                  --HDFAYYFEVKFQPLV---DFDTDSCYSVPAMTMDGTA---S--EGLSPS--------------------D--DVGPCRPRS---ALDRT----NVYVRGRCNRGWCAFVYAYYFQMDW
    XJ511_g1518.t1                  ---GNADEIENKFQPIL---DFDTDGCYNTAAIDPDGNI---N--PGKGAT--------------------G-TPQGDCRDPP---QLENS----NVYSRRRCNNGVCAIMYEYYFEKDQ
    XJ511_g4592.t1                  ---GSAWSEQEKWCPAL---DYDTDSCYNTVAISPSGQL---N--AGQDENK-----------------PAG-EILGWCRKEV---HLQQT----NIYVRSRCNNGWCVHMYDYYFEADF
    XJ511_g5794.t1                  ---GRAPDLDKRFQPAL---DFDTDSCYNVPAIGPNGDL---A--IGMYPF--------------------EWPPQAGCRNEE---MLDRG----NVYSRQRCNNGYCVIFYAYYFQKDT
    XJ511_g5932.t1                  L--GPNGDMIRKFQPLL----HIAHGCQPYSAVNTRGEV---N--AGLQDS--------------------G-TTAGGCK------ETSKG----QTYARSMTLNGQFGIMYAWYWPKDQ
    XJ511_g7291.t1                  VQTGAIGDAIAKFNPLL----HIANGCQPYTAVNDAGDT---S--GGLQDS--------------------G-NISAGCR------DQSKG----QTYARAKVVNGQLAIMYSFYMPKDQ
    XJ511_g8237.t1                  VPSGVSGQLMLKFKPYL----KVFNGCVPFPAVNAGGDT---G--GGLATS--------------------G-SSNGGC-------SSSAG----QVYARAGSYNGANAILYAWYMPKDA
    XJ592_g261.t1                   --HDFAYYFEVKFQPLV---DFDTDSCYSVPAMTMDGTA---S--EGLSPS--------------------D--DVGPCRPRS---ALDRT----NVYVRGRCNRGWCAFVYAYYFQMDW
    XJ592_g3433.t1                  ---GNADEIENKFQPIL---DFDTDGCYNTAAIDPDGNI---N--PGKGAT--------------------G-TPQGDCRDPP---QLENS----NVYSRRRCNNGVCAIMYEYYFEKDQ
    XJ592_g5005.t1                  VQTGAIGDAIAKFNPLL----HIANGCQPYTAVNDAGDT---S--GGLQDS--------------------G-NISAGCR------DQSKG----QTYARAKVVNGQLAIMYSFYMPKDQ
    XJ592_g6487.t1                  ---GSAWSEQEKWCPAL---DYDTDSCYNTVAISPSGQL---N--AGQDENK-----------------PAG-EILGWCRKEV---HLQQT----NIYVRSRCNNGWCVHMYDYYFEADF
    XJ592_g7860.t1                  VPSGVSGQLMLKFKPYL----KVFNGCVPFPAVNAGGDT---G--GGLATS--------------------G-SSNGGC-------SSSAG----QVYARAGSYNGANAILYAWYMPKDA
    XJ592_g8068.t1                  L--GPNGDMIRKFQPLL----HIAHGCQPYSAVNTRGEV---N--AGLQDS--------------------G-TTAGGCK------ETSKG----QTYARSMTLNGQFGIMYAWYWPKDQ
    XJ592_g8206.t1                  ---GRAPDLDKRFQPAL---DFDTDSCYNVPAIGPNGDL---A--IGMYPF--------------------EWPPQAGCRNEE---MLDRG----NVYSRQRCNNGYCVIFYAYYFQKDT
    XJ592_g9637.t1                  ---KRATENDLRYQPAL---DFDTDSCYNVPAIGCDGKI---A--EGLEPD--------------------G--TTKDYRDLA---DLDNT----NVYSRQRCNSGWCAYMYDYYFEKDH
    XJ592_g9637.t2                  ---KRATENDLRYQPAL---DFDTDSCYNVPAIGCDGKI---A--EGLEPD--------------------G--TTKDYRDLA---DLDNT----NVYSRQRCNSGWCAYMYDYYFEKDH
    397_g2445.t1                    LDNSATSQTVKNLQPSL----YIESGCQPYPAIASNGDW---S--GGLNPS--------------------G-SPSGSCR------DLSKA----QVYERGGWHNGVYGIMYAWYMPKDQ
    397_g4853.t1                    VQTGAIGDAIAKFNPRL----HIANGCQPYTAVNDAGDT---S--GGLQDS--------------------G-NISAGCR------DQSKG----QTYARAKVVNGQLAIMYSWYMPKDQ
    397_g6363.t1                    ---GTAWSEQEKWCPAL---DYDKDSCYNTVAISPSGQL---N--AGQDPGK-----------------PAS-EILGWCRKEV---RLQQT----NIYVRSRCDHGWCVHMYDYYFEADF
    397_g7397.t1                    ---GNADEIENKFQPVL---DFDTDGCYNTAAIDPNGNT---N--PGKGAT--------------------G-TPQGDCRDPP---QLENS----NVYSRRRCNNGVCAIMYEYYFEKDQ
    397_g7755.t1                    --YDLAHYFHVKFQPLV---DFDTDSCYSVPAMDLEGTA---S--EGLSPS--------------------G--DVGPCRPRS---ALDRS----NVYVRGRCNRGWCAIVYAYYFQMDW
    397_g8350.t1                    VPSGVSGQLMLKFKPYL----KVFNGCVPFPAVNAGGDT---G--GGLAPS--------------------G-GSNAGC-------SSSPG----QVYARAGSYNGANAIMYSWYMPKDS
    397_g9733.t1                    ---GRAPELDKRFQPAL---DFDTDGCYNVPAIGPNGDL---A--VGLFPF--------------------EWPPQAGCRNAE---MLERG----NVYSRQRCNSGYCVIFYAYYFQKDT
    CBS382.66_g255.t1               --HDFAYYFQVKFQPLV---DFDTDSCYSVPAMTIDGTP---S--EGLSPS--------------------D--DVGPCRPRS---ALDRT----NVYVRGRCNRGWCAFVYAYYFQMDW
    CBS382.66_g255.t2               --HDFAYYFQVKFQPLV---DFDTDSCYSVPAMTIDGTP---S--EGLSPS--------------------D--DVGPCRPRS---ALDRT----NVYVRGRCNRGWCAFVYAYYFQMDW
    CBS382.66_g4327.t1              VPSGVSGQLMLKYKPYL----KVFNGCVPFPAVNAGGDT---G--GGLATS--------------------G-SSNGGC-------SSSPG----QVYARAGTYNGANGILYAWYMPKDA
    CBS382.66_g5334.t1              ---KRATENDLRYQPAL---DFDTDSCYNVPAIGCDGKI---S--EGLEPD--------------------G--TTKDCRDLA---DLDNT----NVYSRQRCNSGWCAYMYDYYFEKDH
    CBS382.66_g5334.t2              --------------------------------------------------------------------------------------------------------------MYDYYFEKDH
    CBS382.66_g5795.t1              VQTGAIGDAIAKFNPLL----HIANGCQPYTAVNDAGDT---S--GGLQDS--------------------G-NISAGCR------DQSKG----QTYARAKVVNGQLAIMYSFYMPKDQ
    CBS382.66_g6578.t1              ---GNADEIENKFQPIL---DFDTDGCYNTAAIDPDGNT---N--PGKGAT--------------------G-TPQGDCRDPP---QLENS----NVYSRRRCNNGVCAIMYEYYFEKDQ
    CBS382.66_g8384.t1              ---GSAWSEQEKWCPAL---DYDTDSCYNTVAISPSGQL---N--AGQDETK-----------------SAG-EILGWCRKDV---RLQQT----NIYVRSRCNNGWCVHMYDYYFEADF
    CBS382.66_g9605.t1              L--GPNGNMIRKFQPLL----HIAHGCQPYSAVNTRGEV---N--AGLQDS--------------------G-TTAGGCK------DTGKG----QTYARSMTLNGQFGIMYAWYWPKDQ
    CBS382.66_g9740.t1              ---GRAPELDKRFQPAL---DFDTDSCYNVPAIGPNGDL---A--IGMFPF--------------------EWPPQAGCRNEE---MLDRG----NVYSRQRCNNGYCVIFYAYYFQKDT
    MUCL9792_g2463.t1               L--GPNGAIIRKFQPLL----HIAHGCQPYTAVNSNNQI---S--GGLQDS--------------------G-TVAGGCR------DTSKG----QTYVRTMNLNGRFGIMYAWYWPKDQ
    MUCL9792_g2802.t1               LDNSATSQTVKTLQPTL----FIESGCQPYPAIAPNGDW---S--GGLNPS--------------------G-SPSGACR------DLTKA----QVYQRGGWHNGVYGIMYAWYMPKDQ
    MUCL9792_g3157.t1               ---PSATAEQEKWCPSL---DYDQDSCYNTVAISPTGQL---N--AGQDHNK-----------------GRD-AILGFCRNEK---RLHNT----NIYVRSRCNNGWCVHMYDYYFESDF
    MUCL9792_g4589.t1               ---KRATENDLRYQPAL---DFDTDSCYNVPAIGCNGNI---V--EGLEPD--------------------G--TTNGCRDVA---DLDNT----NVYSRQRCNSGWCAYMYDYYFEKDH
    MUCL9792_g4589.t2               --------------------------------------------------------------------------------------------------------------MYDYYFEKDH
    MUCL9792_g4589.t3               ---KRATENDLRYQPAL---DFDTDSCYNVPAIGCNGNI---V--EGLEPD--------------------G--TTNGCRDVA---DLDNT----NV-----------------------
    MUCL9792_g6332.t1               ---GNADEIENKFQPIL---DFDTDGCYNTAAIDPDGNI---N--PGKGAT--------------------G-TPQGDCRDPP---QLENS----NVYSRRRCNNGVCAIMYEYYFEKDQ
    MUCL9792_g7308.t1               VPSGAAGQH------------------------------------GGLNPT--------------------G-SSSASC-------SSSAG----QVYARRGTHNGVSAIMYAWYMPKDS
    MUCL9792_g10611.t1              VQTGPIGDAIAKFNPRL----HIAHGCQPYTAVNDAGDT---S--GGLQDS--------------------G-NISAGCR------DQSKG----QTYARAKVVNGQLAIMYSWYWPKDQ
    PD401_g2361.t1                  LDNSATSQTVKTLQPTL----YIESGCQPYPAIAANGDW---S--GGLNPS--------------------G-SPSGACR------DLTKA----QVYQRGGWHNGVYGIMYAWYMPKDQ
    PD401_g3373.t1                  VPSGATGQQYLKHKPYL----KVFTGCVPFPAVNAGGDT---G--GGLNPT--------------------G-SSSASC-------SSSAG----QVYARRGTHNGVSAIMYAWYMPKDS
    PD401_g4730.t1                  ---GNADEIENKFQPIL---DFDTDGCYNTAAIDPDGNI---N--PGKGAT--------------------G-TPQGDCRDPP---QLENS----NVYSRRRCNNGVCAIMYEYYFEKDQ
    PD401_g7339.t1                  VQTGPIGDAIAKFNPRL----HIAHGCQPYTAVNDAGDT---S--GGLQDS--------------------G-NISAGCR------DQAKG----QTYARAKVVNGQLAIMYAWYWPKDQ
    PD401_g10483.t1                 L--GPNGAIIRKFQPLL----HIAHGCQPYTAVNSNNQI---S--GGLQDS--------------------G-TVAGGCR------DTSKG----QTYVRTMNLNGRFGIMYAWYWPKDQ
    PD593_g749.t1                   ---KRATENDLRYQPAL---DFDTDSCYNVPAIGCNGNI---V--EGLEPD--------------------G--TTNGCRDVA---DLDNT----NVYSRQRCNSGWCAYMYDYYFEKDH
    PD593_g2541.t1                  LDNSATSQTVKTLQPTL----FIESGCQPYPAIAPNGDW---S--GGLNPS--------------------G-SPSGACR------DLTKA----QVYQRGGWHNGVYGIMYAWYMPKDQ
    PD593_g2728.t1                  ---GNADEIENKFQPIL---DFDTDGCYNTAAIDPDGNI---N--PGKGAT--------------------G-TPQGDCRDPP---QLENS----NVYSRRRCNNGVCAIMYEYYFEKDQ
    PD593_g7030.t1                  VPSGAAGQQYLKHKPYL----KVFTGCVPFPAVNAGGDT---G--GGLNPT--------------------G-SSSASC-------SSSAG----QVYARRGTHNGVSAIMYAWYMPKDS
    PD593_g8432.t1                  VQTGPIGDAIAKFNPRL----HIAHGCQPYTAVNDAGDT---S--GGLQDS--------------------G-NISAGCR------DQSKG----QTYARAKVVNGQLAIMYSWYWPKDQ
    PD593_g9596.t1                  L--GPNGAIIRKFQPLL----HIAHGCQPYTAVNSNNQI---S--GGLQDS--------------------G-TVAGGCR------DTSKG----QTYVRTMNLNGRFGIMYAWYWPKDQ
    PD618_g114.t1                   LDNSATSQTVKALQPTL----YIESGCQPYPAIAANGDW---S--GGLNPS--------------------G-SPSGACR------DLTKA----QVYQRGGWHNGVYGIMYAWYMPKDQ
    PD618_g3974.t1                  ---GNADEIENKFQPIL---DFDTDGCYNTAAIDPDGNI---N--PGKGAT--------------------G-TPQGDCRDPP---QLENS----NVYSRRRCNNGVCAIMYEYYFEKDQ
    PD618_g5755.t1                  VPSGATGQQYLKHKPYL----KVFTGCVPFPAVNAGGDT---G--GGLNPT--------------------G-SSSASC-------SSSAG----QVYARRGTHNGVSAIMYAWYMPKDS
    PD618_g9213.t1                  VQTGPIGDAIAKFNPRL----HIAHGCQPYTAVNDAGDT---S--GGLQDS--------------------G-NISAGCR------DQAKG----QTYARAKVVNGQLAIMYAWYWPKDQ
    PD618_g9942.t1                  L--GPNGAIIRKFQPLL----HIAHGCQPYTAVNSNNQI---S--GGLQDS--------------------G-TVAGGCR------DTSKG----QTYVRTMNLNGRFGIMYAWYWPKDQ
    PD659_g6753.t1                  LDNSATSQTVKTLQPTL----YIESGCQPYPAIAANGDW---S--GGLNPS--------------------G-SPSGACR------DLTKA----QVYQRGGWHNGVYGIMYTWYMPKDQ
    PD659_g7370.t1                  VQTGPIGDAIAKFNPRL----HIAHGCQPYTAVNDAGDT---S--GGLQDS--------------------G-NISAGCR------DQAKG----QTYARAKVVNGQLAIMYAWYWPKDQ
    PD659_g10785.t1                 L--GPNGAIIRKFQPLL----HIAHGCQPYTAVNSNNQI---S--GGLQDS--------------------G-TVAGGCR------DTSKG----QTYVRTMNLNGRFGIMYAWYWPKDQ
    PD659_g2262.t1                  ---GNADEIENKFQPIL---DFDTDGCYNTAAIDPDGNI---N--PGKGAT--------------------G-TPQGDCRDPP---QLENS----NVYSRRRCNNGVCAIMYEYYFEKDQ
    PD659_g5819.t1                  VPSGATGQQYLKHKPYL----KVFTGCVPFPAVNAGGDT---G--GGLNPT--------------------G-SSSASC-------SSSAG----QVYARRGTHNGVSAIMYAWYMPKDS
    PD660_g1229.t1                  VPSGATGQQYLKHKPYL----KVFTGCVPFPAVNAGGDT---G--GGLNPT--------------------G-SSSASC-------SSSAG----QVYARRGTHNGVSAIMYAWYMPKDS
    PD660_g3973.t1                  ---GNADEIENKFQPIL---DFDTDGCYNTAAIDPDGNI---N--PGKGAT--------------------G-TPQGDCRDPP---QLENS----NVYSRRRCNNGVCAIMYEYYFEKDQ
    PD660_g6305.t1                  L--GPNGAIIRKFQPLL----HIAHGCQPYTAVNSNNQI---S--GGLQDS--------------------G-TVAGGCR------DTSKG----QTYVRTMNLNGRFGIMYAWYWPKDQ
    PD660_g7826.t1                  VQTGPIGDAIAKFNPRL----HIAHGCQPYTAVNDAGDT---S--GGLQDS--------------------G-NISAGCR------DQAKG----QTYARAKVVNGQLAIMYAWYWPKDQ
    PD660_g9669.t1                  LDNSATSQTVKALQPTL----YIESGCQPYPAIAANGDW---S--GGLNPS--------------------G-SPSGACR------DLTKA----QVYQRGGWHNGVYGIMYAWYMPKDQ
    PD660_g10032.t1                 ---PSATSEQEKWCPSL---DYDQDSCYNTVAISPTGQL---N--AGQDHKK-----------------GKD-AILGFCRNEK---RLHNT----NIYVRSCCNNGWCVHMYDYYFESDF
    PD670_g4670.t1                  ---GNADEIENKFQPIL---DFDTDGCYNTAAIDPNGLT---N--PGKGAT--------------------G-TPQGDCRDPH---QLENS----NVYSRRRCNNGVCAIMYEYYFEKDQ
    PD670_g5219.t1                  VPSGVAGQLYLKHKPYL----KVFNGCVPFPAVNAAGDT---G--GGLNPT--------------------G-SSSASC-------SSSTG----QVYARQGVYNGANAIMYSWYMPKDS
    PD670_g7258.t1                  VQTGAIGDAIAKFNPRL----HIANGCQPYTAVNDAGDT---S--GGLQDS--------------------G-NISAGCR------DQSKG----QTYARAKVVNGQLAIMYAFYMPKDQ
    PD670_g8696.t1                  ---GRAPELDKRFQPAL---DFDTDSCYNVPAIGPNGDL---S--LGLYPF--------------------DWPPQRDCRNEE---MLERS----NVYSRQRCNSGYCVILYAYYFQKDQ
    PD670_g10480.t1                 VGSGVTATLYLKYKPVL----RVINGCVPFPAVDKNGNT---S--GGLKNS--------------------G-AMNGKC-------SKSPG----QIYVRSASRKGHFAIMYAWYFPKDQ
    PD670_g10529.t1                 L--GSNGAIIRKFQPLL----HIAHGCQPYTAVNSNNQI---S--GGLQDS--------------------G-TVAGGCR------DTSKG----QTYVRTMNLNGRFGIMYAWYWPKDQ
    PD670_g11082.t1                 LDNSATSKTVKDLQPSL----FIESGCQPYPAINTNGDW---S--AGLNLG--------------------G-SPSGSCR------DLSKA----QVYQRGGWHNNVYGIMYAWYMPKDQ
    PD670_g2580.t1                  ---GTAWSEQEKWCPAL---DYDRNACYNTVAISPSGQL---N--AGQDQNK-----------------GEG-EILGWCRKEE---RLRQT----NIYVRSRCNNGWCVHMYDYYFEADW
    PD670_g3093.t1                  ---KRATENDLRYQPAL---DFDTDSCYNVPAIGCNGNI---V--EGLEPD--------------------V--TTNGCRDVA---DLDNT----NVYSRQRCNSGWCAYMYDYYFEKDH
    PD683_g248.t1                   --HDFAYYFQVKFQPLV---DFDTDSCYSVPAMTIDGTP---S--EGLSPS--------------------D--DVGPCRPRS---ALDRT----NVYVRGRCNRGWCAFVYAYYFQMDW
    PD683_g3766.t1                  ---GRAPELDKRFQPAL---DFDTDSCYNVPAIGPNGDL---A--IGMFPF--------------------EWPPQAGCRNEE---MLDRG----NVYSRQRCNNGYCVIFYAYYFQKDT
    PD683_g3895.t1                  L--GPNGNMIRKFQPLL----HIAHGCQPYSSVNTRGEV---N--AGLQDS--------------------G-TTAGGCK------DTSKG----QTYARSMTLNGQFGIMYAWYWPKDQ
    PD683_g4100.t1                  VPSGVSGQLMLKYKPYL----KVFNGCVPFPAVNAGGDT---G--GGLATS--------------------G-SSNGGC-------SSSPG----QVYARAGTYNGANAILYAWYMPKDA
    PD683_g5003.t1                  ---KRATENDLRYQPAL---DFDTDSCYNVPAIGCDGKI---S--EGLEPD--------------------G--TTKDCRDLA---DLDNT----NVYSRQRCNSGWCAYMYDYYFEKDH
    PD683_g5003.t2                  --------------------------------------------------------------------------------------------------------------MYDYYFEKDH
    PD683_g5713.t1                  VQTGAIGDAIAKFNPLL----HIANGCQPYTAVNDAGDT---S--GGLQDS--------------------G-NISAGCR------DQSKG----QTYARAKVVNGQLAIMYSFYMPKDQ
    PD683_g7092.t1                  ---GNADEIENKFQPIL---DFDTDGCYNTAAIDPDGNT---N--PGKGAT--------------------G-TPQGDCRDPP---QLENS----NVYSRRRCNNGVCAIMYEYYFEKDQ
    PD683_g8130.t1                  ---GSAWSEQEKWCPAL---DYDTDSCYNTVAISPSGQL---N--AGQDETK-----------------SAG-EILGWCRKEV---RLQQT----NIYVRSRCNNGWCVHMYDYYFEADF
    PD736_g919.t1                   VPSGATGQQYLKHKPYL----KVFTGCVPFPAVNAGGDT---G--AGLNPT--------------------G-SSSASC-------SSSAG----QVYARRGAHNGVSAIMYAWYMPKDS
    PD736_g8670.t1                  L--GPNGAIIRKFQPLL----HIAHGCQPYTAVNSNNQI---S--GGLQDS--------------------G-TVAGGCR------DTSKG----QTYVRTMNLNGRFGIMYAWYWPKDQ
    PD736_g9411.t1                  VQTGPIGDAIAKFNPRL----HIAHGCQPYTAVNDAGDT---S--GGLQDS--------------------G-NISAGCR------DQAKG----QTYARAKVVNGQLAIMYSWYWPKDQ
    PD736_g10271.t1                 ---GNADEVENKFQPIL---DFDTDGCYNTAAIDPDGNT---N--PGKGAT--------------------G-TPQGDCRDPH---QLENS----NVYSRRRCNNGVCAIMYEYYFEKDQ
    PD736_g10840.t1                 ---KRATENDLRYQPAL---DFDTDSCYNVPAIGCNGNI---V--EGLEPD--------------------G--TTKGCRDLA---DLDNT----NVYSRQRCNSGWCAYMYDYYFEKDH
    PD736_g10939.t1                 LDNSATSQTVKTLQPSF----YIESGCQPYPAIAANGDW---S--GGLNPS--------------------G-SPSGACR------DLSKA----QVYQRGGWHNGVYGIMYAWYMPKDQ
    PD739_g34.t1                    ---KRATENDLRYQPAL---DFDTDSCYNVPAIGCDGNI---V--EGLEPD--------------------G--TTKGCRDLA---DLDNT----NVYSRQRCNSGWCAYMYDYYFEKDH
    PD739_g131.t1                   LDNSATSQTVKTLQPSL----YIESGCQPYPAIAANGDW---S--GGLNPS--------------------G-SPSGACR------DLSKA----QVYQRGGWHNGVYGIMYAWYMPKDQ
    PD739_g4443.t1                  ---GNADEIENKFQPIL---DFDTDGCYNTAAIDPDGNT---N--PGKGAT--------------------G-TPQGDCRDPH---QLENS----NVYSRRRCNNGVCAIMYEYYFEKDQ
    PD739_g7436.t1                  VPSGATGQQYLKHKPYL----KVFTGCVPFPAVNAGGDT---G--AGLNPT--------------------G-SSSASC-------SSSAG----QVYARRGAHNGVNAIMYAWYMPKDS
    PD739_g8512.t1                  L--GPNGAIIRKFQPLL----HIAHGCQPYTAVNSNNQI---S--GGLQDS--------------------G-TVAGGCR------DTSKG----QTYVRTMNLNGRFGIMYAWYWPKDQ
    PD739_g10815.t1                 VQTGPIGDAIAKFNPRL----HIVHGCQPYTAVNDAGDT---S--GGLQDS--------------------G-NISAGCR------DQAKG----QTYARAKVVNGQLAIMYSWYWPKDQ
    PD747_g20.t1                    ---KRATENDLH---------FDTDSCYNVPAIGCNGNI---V--EGLEPD--------------------V--TTNGCRDVA---DLDNT----NVYSRQRCNSGWCAYMYDYYFEKDH
    PD747_g277.t1                   LDNSATSKTVKDLQPSL----FIESGCQPYPAINTNGDW---S--AGLNLG--------------------G-SPSGSCR------DLSKA----QVYQRGGWHNNVYGIMYAWYMPKDQ
    PD747_g4174.t1                  ---GNADEIENKFQPIL---DFDTDGCYNTAAIDPNGLT---N--PGKGAT--------------------G-TPQGDCRDPH---QLENS----NVYSRRRCNNGVCAIMYEYYFEKDQ
    PD747_g5167.t1                  VPSGVAGQLYLKHKPYL----KVFNGCVPFPAVNAAGDT---G--GGLNPT--------------------G-SSSASC-------SSSTG----QVYARQGVYNGANAIMYSWYMPKDS
    PD747_g9207.t1                  ---GTAWSEQEKWCPAL---DYDRNACYNTVAISPSGQL---N--AGQDQNK-----------------GEG-EILGWCRKEE---RLRQT----NIYVRSRCNNGWCVHMYDYYFEADW
    PD747_g9751.t1                  VQTGAIGDAIAKFNPRL----HIANGCQPYTAVNDAGDT---S--GGLQDS--------------------G-NISAGCR------DQSKG----QTYARAKVVNGQLAIMYAFYMPKDQ
    PD747_g10403.t1                 ---GRAPELDKRFQPAL---DFDTDSCYNVPAIGPNGDL---S--LGLYPF--------------------DWPPQRDCRNEE---MLERS----NVYSRQRCNSGYCVILYAYYFQKDQ
    PD747_g10498.t1                 VGSGVTATLYLKYKPVL----RVINGCVPFPAVDKNGNT---S--GGLKNS--------------------G-AMNGKC-------SKSPG----QIYVRSASRKGHFAIMYAWYFPKDQ
    PD747_g10549.t1                 L--GSNGAIIRKFQPLL----HIAHGCQPYTAVNSNNQI---S--GGLQDS--------------------G-TVAGGCR------DTSKG----QTYVRTMNLNGRFGIMYAWYWPKDQ
    T2_g2030.t1                     --HDFAYYFQVKFQPLV---DFDTDSCYSVPAMTIDGTP---S--EGLSPS--------------------D--DVGPCRPRS---ALDRT----NVYVRGRCNRGWCAFVYAYYFQMDW
    T2_g2030.t2                     --HDFAYYFQVKFQPLV---DFDTDSCYSVPAMTIDGTP---S--EGLSPS--------------------D--DVGPCRPRS---ALDRT----NVYVRGRCNRGWCAFVYAYYFQMDW
    T2_g3751.t1                     ---KRATENDLRYQPAL---DFDTDSCYNVPAIGCDGKI---S--EGLEPD--------------------G--TTKDCRDLA---DLDNT----NVYSRQRCNSGWCAYMYDYYFEKDH
    T2_g3751.t2                     --------------------------------------------------------------------------------------------------------------MYDYYFEKDH
    T2_g4661.t1                     VPSGVSGQLMLKYKPYL----KVFNGCVPFPAVNAGGDT---G--GGLATS--------------------G-SSNGGC-------SSSPG----QVYARAGTYNGANGILYAWYMPKDA
    T2_g4868.t1                     L--GPNGNMIRKFQPLL----HIAHGCQPYSAVNTRGEV---N--AGLQDS--------------------G-TTAGGCK------DTGKG----QTYARSMTLNGQFGIMYAWYWPKDQ
    T2_g5004.t1                     ---GRAPELDKRFQPAL---DFDTDSCYNVPAIGPNGDL---A--IGMFPF--------------------EWPPQAGCRNEE---MLDRG----NVYSRQRCNNGYCVIFYAYYFQKDT
    T2_g5801.t1                     VQTGAIGDAIAKFNPLL----HIANGCQPYTAVNDAGDT---S--GGLQDS--------------------G-NISAGCR------DQSKG----QTYARAKVVNGQLAIMYSFYMPKDQ
    T2_g7120.t1                     ---GSAWSEQEKWCPAL---DYDTDSCYNTVAISPSGQL---N--AGQDETK-----------------SAG-EILGWCRKDV---RLQQT----NIYVRSRCNNGWCVHMYDYYFEADF
    T2_g7997.t1                     ---GNADEIENKFQPIL---DFDTDGCYNTAAIDPDGNT---N--PGKGAT--------------------G-TPQGDCRDPP---QLENS----NVYSRRRCNNGVCAIMYEYYFEKDQ
    VSO1_g539.t1                    VPSGATGQQYLKHKPYL----KVFTGCVPFPAVNAGGDT---G--GGLNPT--------------------G-SSSASC-------SSSAG----QVYARRGTHNGVSAIMYAWYMPKDS
    VSO1_g1387.t1                   LDNSATSQTVKALQPTL----YIESGCQPYPAIAANGDW---S--GGLNPS--------------------G-SPSGACR------DLTKA----QVYQRGGWHNGVYGIMYAWYMPKDQ
    VSO1_g1731.t1                   L--GPNGAIIRKFQPLL----HIAHGCQPYTAVNSNNQI---S--GGLQDS--------------------G-TVAGGCR------DTSKG----QTYVRTMNLNGRFGIMYAWYWPKDQ
    VSO1_g1937.t1                   ---PSATSEQERWCPSL---DYDQDSCYNTVAISPTGQL---N--AGQDHKK-----------------GKD-AILGFCRNEK---RLHNT----NIYIRSRCNNGWCVHMYDYYFESDF
    VSO1_g4972.t1                   ---GNADEIENKFQPIL---DFDTDGCYNTAAIDPDGNI---N--PGKGAT--------------------G-TPQGDCRDPP---QLENS----NVYSRRRCNNGVCAIMYEYYFEKDQ
    VSO1_g5184.t1                   VQTGPIGDAIAKFNPRL----HIAHGCQPYTAVNDAGDT---S--GGLQDS--------------------G-NISAGCR------DQAKG----QTYARAKVVNGQLAIMYAWYWPKDQ
    VL20_g7800.t1                   --HDFAYYFEVKFQPLV---DFDTDSCYSVPAMTMDGTA---S--EGLSPS--------------------D--DVGPCRPRS---ALDRT----NVYVRGRCNRGWCAFVYAYYFQMDW
    VL20_g9415.t1                   ---GSADEIENKFQPIL---DFDTDGCYNTAAIDPDGNI---N--PGKGAT--------------------G-TPQGDCRDPP---QLENS----NVYSRRRCNNGVCAIMYEYYFEKDQ
    VL20_g11025.t1                  L--GPNGNMIRKFQPLL----HIAHGCQPYSAVNTRGEV---N--AGLQDS--------------------G-TTADGCK------LTDKG----QTYARSMTLNGQFGIMYAWYWPKDQ
    VL20_g11172.t1                  --HDFAYYFEVKFQPLV---DIDTDSCYSVPAMTMDGTA---S--EGLSPS--------------------D--DVGPCRPRS---ALDRS----NVYVRGRCNRGWCAFVYAYYFQMDW
    VL20_g12965.t1                  ---GSAWSEQEKWCPAL---DYDTDSCYNTVAISPSGQL---N--AGQDENK-----------------PAG-EILGWCRKEV---RLQQT----NIYVRSRCNNGWCVHMYDYYFEADF
    VL20_g13837.t1                  VPSGVSGQLMLKFKPYL----KVFNGCVPFPAVNAGGDT---G--GGLATS--------------------G-SSNGGC-------SSSAG----QVYARAGSYNGANAILYAWYMPKDA
    VL20_g14042.t1                  L--GPNGDMIRKFQPLL----HIAHGCQPYSAVNTRGEV---N--AGLQDS--------------------G-TTAGGCK------ETSKG----QTYARSMTLNGQFGIMYAWYWPKDQ
    VL20_g14171.t1                  ---GRAPDLDKRFQPAL---DFDTDSCYNVPAIGPNGDL---A--IGMYPF--------------------EWPPQAGCRNEE---MLDRG----NVYSRQRCNNGYCVIFYAYYFQKDT
    VL20_g14284.t1                  ---GNADEIENKFQPIL---DFDTDGCYNTAAIDPDGNT---N--PGKGAT--------------------G-TPQGDCRDPP---QLENS----NVYSRRRCNNGVCAIMYEYYFEKDQ
    VL20_g17450.t1                  VQTGAIGDAIAKFNPLL----HIANGCQPYTAVNDAGDT---S--GGLQDS--------------------G-NISAGCR------DQSKG----QTYARAKVVNGQLAIMYSFYMPKDQ
    VL20_g18857.t1                  VQTGAIGDAIAKFNPLL----HIANGCQPYTAVNDAGDT---S--GGLQDS--------------------G-NISAGCR------DQSKG----QTYARAKVVNGQLAIMYSFYMPKDQ
    VL20_g4752.t1                   VPSGVSGQLMLKYKPYL----KVFNGCVPFPAVNAGGDT---G--GGLATS--------------------G-SSNGGC-------ASSAG----QVYARAGTYNGANAIMYAWYMPKDA
    VL20_g5938.t1                   ---GSAWSEQEKWCPAL---DYDTDSCYNTVAISPSGQL---N--AGQDETK-----------------SGG-EILGWCRKEV---RLQQT----NIYVRSRCNNGWCVHMYDYYFEADF
    VL20_g5938.t2                   ---GSAWSEQEKWCPAL---DYDTDSCYNTVAISPSGQL---N--AGQDETK-----------------SGG-EILGWCRKEV---RLQQT----NIYVRSRCNNGWCVHMYDYYFEADF
    VL20_g6061.t1                   ---GRAPELDKRFQPAL---DFDTDSCYNVPAIGPNGDL---A--IGMFPF--------------------EWPPQAGCRNEE---MLDRG----NVYSRQRCNNGYCVIFYAYYFQKDT
    VL20_g18964.t1                  ---GSAWSEQEKWCPAL---DYDTDSCYNTVAISPSGQL---N--AGQDETK-----------------SGG-EILGWCRKEV---RLQQT----NIYVRSRCNNGWCVHMYDYYFEADF
    VL20_g18964.t2                  ---GSAWSEQEKWCPAL---DYDTDSCYNTVAISPSGQL---N--AGQDETK-----------------SGG-EILGWCRKEV---RLQQT----NIYVRSRCNNGWCVHMYDYYFEADF
    VLB2_g585.t1                    --HDFAYYFEVKFQPLV---DFDTDSCYSVPAMTMDGTA---S--EGLSPS--------------------D--DVGPCRPRS---ALDRT----NVYVRGRCNRGWCAFVYAYYFQMDW
    VLB2_g1761.t1                   L--GPNGNMIRKFQPLL----HIAHGCQPYSAVNTRGEV---N--AGLQDS--------------------G-TTAGGCK------LTDKG----QTYARSMTLNGQFGIMYAWYWPKDQ
    VLB2_g2033.t1                   --HDFAYYFEVKFQPLV---DIDTDSCYSVPAMTMDGTA---S--EGLSPS--------------------D--DVGPCRPRS---ALDRS----NVYVRGRCNRGWCAFVYAYYFQMDW
    VLB2_g12050.t1                  VQTGAIGDAIAKFNPLL----HIANGCQPYTAVNDAGDT---S--GGLQDS--------------------G-NISAGCR------DQSKG----QTYARAKVVNGQLAIMYSFYMPKDQ
    VLB2_g12617.t1                  ---GSAWSEQEKWCPAL---DYDTDSCYNTVAISPSGQL---N--AGQDENK-----------------PAG-EILGWCRKEV---RLQQT----NIYVRSRCNNGWCVHMYDYYFEADF
    VLB2_g13499.t1                  VPSGVSGQLMLKFKPYL----KVFNGCVPFPAVNAGGDT---G--GGLATS--------------------G-SSNGGC-------SSSAG----QVYARAGSYNGANAILYAWYMPKDA
    VLB2_g13703.t1                  L--GPNGDMIRKFQPLL----HIAHGCQPYSAVNTRGEV---N--AGLQDS--------------------G-TTAGGCK------ETSKG----QTYARSMTLNGQFGIMYAWYWPKDQ
    VLB2_g13833.t1                  ---GRAPDLDKRFQPAL---DFDTDSCYNVPAIGPNGDL---A--IGMYPF--------------------EWPPQAGCRNEE---MLDRG----NVYSRQRCNNGYCVIFYAYYFQKDT
    VLB2_g14318.t1                  ---GNADEIENKFQPIL---DFDTDGCYNTAAIDPDGNT---N--PGKGAT--------------------G-TPQGDCRDPP---QLENS----NVYSRRRCNNGVCAIMYEYYFEKDQ
    VLB2_g15187.t1                  ---GSAWSEQEKWCPAL---DYDTDSCYNTVAISPSGQL---N--AGQDETK-----------------SGG-EILGWCRKEV---RLQQT----NIYVRSRCNNGWCVHMYDYYFEADF
    VLB2_g15187.t2                  ---GSAWSEQEKWCPAL---DYDTDSCYNTVAISPSGQL---N--AGQDETK-----------------SGG-EILGWCRKEV---RLQQT----NIYVRSRCNNGWCVHMYDYYFEADF
    VLB2_g16101.t1                  VPSGVSGQLMLKYKPYL----KVFNGCVPFPAVNAGGDT---G--GGLATS--------------------G-SSNGGC-------ASSAG----QVYARAGTYNGANAIMYAWYMPKDA
    VLB2_g16543.t1                  ---GSADEIENKFQPIL---DFDTDGCYNTAAIDPDGNI---N--PGKGAT--------------------G-TPQGDCRDPP---QLENS----NVYSRRRCNNGVCAIMYEYYFEKDQ
    VLB2_g19098.t1                  ---KRATENDLRYQPAL---DFDTDSCYNVPAIGCDGKI---A--EGLEPD--------------------G--TKKDCRDLA---DLDNT----NVYSRQRCNSGWCAYMYDYYFEKDH
    VLB2_g5814.t1                   VQTGAIGDAIAKFNPLL----HIANGCQPYTAVNDAGDT---S--GGLQDS--------------------G-NISAGCR------DQSKG----QTYARAKVVNGQLAIMYSFYMPKDQ
    VLB2_g6534.t1                   ---GRAPELDKRFQPAL---DFDTDSCYNVPAIGPNGDL---A--IGMFPF--------------------EWPPQAGCRNEE---MLDRG----NVYSRQRCNNGYCVIFYAYYFQKDT
    PD589_g51.t1                    ---GSAWSEQEKWCPAL---DYDTDSCYNTVAISPSGQL---N--AGQDENK-----------------PAG-EILGWCRKEV---HLQQT----NIYVRSRCNNGWCVHMYDYYFEADF
    PD589_g856.t1                   ----------------M----ALPHDIAPIHSISV-------------------------------------------------------------------------------------
    PD589_g7665.t1                  ---GNADEIENKFQPIL---DFDTDGCYNTAAIDPDGNI---N--PGKGAT--------------------G-TPQGDCRDPP---QLENS----NVYSRRRCNNGVCAIMYEYYFEKDQ
    PD589_g13197.t1                 --HDFAYYFEVKFQPLV---DFDTDSCYSVPAMTMDGTA---S--EGLSPS--------------------D--DVGPCRPRS---ALDRT----NVYVRGRCNRGWCAFVYAYYFQMDW
    PD589_g13425.t1                 VQTGAIGDAIAKFNPLL----HIANGCQPYTAETNAG-----------------------------------------CRRCRRYQRRSPG----QWQHFCGVVNGQLAIMYSFYMPKDQ
    PD589_g14403.t1                 ---GRAPELGKRFQPVL---DYDTDSYYNVPAIGPNGYF---A--IGMFPF--------------------EWPPQAGCRNEE---MLDRG----NVYSRQRCNNGYCVIFYAYYFQKDT
    PD589_g14539.t1                 ---GRAPDLDKRFQPAL---DFDTDSCYNVPAIGPNGDL---A--IGMYPF--------------------EWPPQAGCRNEE---MLDRG----NVYSRQRCNNGYCVIFYAYYFQKDT
    PD589_g14661.t1                 L--GPNGDMIRKFQPLL----HIAHGCQPYSAVNTRGEV---N--AGLQDS--------------------G-TTAGGCK------ETSKG----QTYARSMTLNGQFGIMYAWYWPKDQ
    PD589_g15310.t1                 VQTGAIGDAIAKFNPLL----HIANGCQPYTAVNDAGDT---S--GGLQDS--------------------G-NISAGCR------DQSKG----QTYARAKVVNGQLAIMYSFYMPKDQ
    PD589_g16171.t1                 ---GSAWSEQEKWCPAL---DYDTDSCYNTVAISPSGQL---N--AGQDETK-----------------SGG-EILGWCRKEV---RLQQT----NIYVRSRCNNGWCVHMYDYYFEADF
    PD589_g16171.t2                 ---GSAWSEQEKWCPAL---DYDTDSCYNTVAISPSGQL---N--AGQDETK-----------------SGG-EILGWCRKEV---RLQQT----NIYVRSRCNNGWCVHMYDYYFEADF
    PD589_g17659.t1                 ---GNADEIENKFQPIL---DFDTDGCYNTAAIDPDGNT---N--PGKGAT--------------------G-TPQGDCRDPP---QLENS----NVYSRRRCNNGVCAIMYEYYFEKDQ
    PD589_g18192.t1                 VPSGVSGQLMLKFKPYL----KVFNGCVPFPAVNAGGDT---G--GGLATS--------------------G-SSNGGC-------SSSAG----QVYARAGSYNGANAILYAWYMPKDA
    PD589_g3050.t1                  L--GPNGNMIRKFQPLL----HIAHGCQPYSAVNTRGEV---N--AGLQDS--------------------G-TTAGGCK------LTDKG----QTYARSMTLNGQFGIMYAWYWPKDQ
    PD589_g6680.t1                  --HDFAYYFEVKFQPLV---DIDTDSCYSVPAMTMDGTA---S--EGLSPS--------------------D--DVGPCRPRS---ALDRS----NVYVRGRCNRGWCAFVYAYYFQMDW
    PD589_g6775.t1                  VPSGVSGQLMLKYKPYL----KVFNGCVPFPAVNAGGDT---G--GGLATS--------------------G-SSNGGC-------ASSAG----QVYARAGTYNGANAIMYAWYMPKDA
    Vt305_g1664.t1                  L--GPNGAIIRKFQPLL----HIAHGCQPYTAVNSNNQI---S--GGLQDS--------------------G-TVAGGCR------DTSKG----QTYVRTMNLNGRFGIMYAWYWPKDQ
    Vt305_g2624.t1                  ---GNADEIENKFQPIL---DFDTDGCYNTAAIDPDGNI---N--PGKGAT--------------------G-TPQGDCRDPP---QLENS----NVYSRRRCNNGVCAIMYEYYFEKDQ
    Vt305_g3295.t1                  VQTGPIGDAIAKFNPRL----HIAHGCQPYTAVNDAGDT---S--GGLQDS--------------------G-NISAGCR------DQAKG----QTYARAKVVNGQLAIMYAWYWPKDQ
    Vt305_g4616.t1                  LDNSATSQTVKALQPTL----YIESGCQPYPAIAANGDW---S--GGLNPS--------------------G-SPSGACR------DLTKA----QVYQRGGWHNGVYGIMYAWYMPKDQ
    Vt305_g7985.t1                  VPSGATGQQYLKHKPYL----KVFTGCVPFPAVNAGGDT---G--GGLNPT--------------------G-SSSASC-------SSSAG----QVYARRGTHNGVSAIMYAWYMPKDS
    WCS072_g2037.t1                 --HDFAYYFQVKFQPLV---DFDTDSCYSVPAMTIDGTP---S--EGLSPS--------------------D--DVGPCRPRS---ALDRT----NVYVRGRCNRGWCAFVYAYYFQMDW
    WCS072_g2037.t2                 --HDFAYYFQVKFQPLV---DFDTDSCYSVPAMTIDGTP---S--EGLSPS--------------------D--DVGPCRPRS---ALDRT----NVYVRGRCNRGWCAFVYAYYFQMDW
    WCS072_g3872.t1                 ---GRAPELDKRFQPAL---DFDTDSCYNVPAIGPNGDL---A--IGMFPF--------------------EWPPQAGCRNEE---MLDRG----NVYSRQRCNNGYCVIFYAYYFQKDT
    WCS072_g4007.t1                 L--GPNGNMIRKFQPLL----HIAHGCQPYSAVNTRGEV---N--AGLQDS--------------------G-TTAGGCK------DTGKG----QTYARSMTLNGQFGIMYAWYWPKDQ
    WCS072_g4219.t1                 VPSGVSGQLMLKYKPYL----KVFNGCVPFPAVNAGGDT---G--GGLATS--------------------G-SSNGGC-------SSSPG----QVYARAGTYNGANGILYAWYMPKDA
    WCS072_g5132.t1                 ---KRATENDLRYQPAL---DFDTDSCYNVPAIGCDGKI---S--EGLEPD--------------------G--TTKDCRDLA---DLDNT----NVYSRQRCNSGWCAYMYDYYFEKDH
    WCS072_g5132.t2                 --------------------------------------------------------------------------------------------------------------MYDYYFEKDH
    WCS072_g5615.t1                 VQTGAIGDAIAKFNPLL----HIANGCQPYTAVNDAGDT---S--GGLQDS--------------------G-NISAGCR------DQSKG----QTYARAKVVNGQLAIMYSFYMPKDQ
    WCS072_g6396.t1                 ---GNADEIENKFQPIL---DFDTDGCYNTAAIDPDGNT---N--PGKGAT--------------------G-TPQGDCRDPP---QLENS----NVYSRRRCNNGVCAIMYEYYFEKDQ
    WCS072_g8181.t1                 ---GSAWSEQEKWCPAL---DYDTDSCYNTVAISPSGQL---N--AGQDETK-----------------SAG-EILGWCRKDV---RLQQT----NIYVRSRCNNGWCVHMYDYYFEADF
    VDAG_JR2_Chr1g22850a-00001      --HDFAYYFEVKFQPLV---DFDTDSCYSVPAMTMDGTA---S--EGLSPS--------------------D--DVGPCRPRS---ALDRT----NVYVRGRCNRGWCAFVYAYYFQMDW
    VDAG_JR2_Chr2g05460a-00001      VPSGVSGQLMLKFKPYL----KVFNGCVPFPAVNAGGDT---G--GGLATS--------------------G-SSNGGC-------SSSAG----QVYARAGSYNGANAILYAWYMPKDA
    VDAG_JR2_Chr6g08770a-00001      L--GPNGDMIRKFQPLL----HIAHGCQPYSAVNTRGEV---N--AGLQDS--------------------G-TTAGGCK------ETSKG----QTYARSMTLNGQFGIMYAWYWPKDQ
    VDAG_JR2_Chr6g10260a-00001      ---GRAPDLDKRFQPAL---DFDTDSCYNVPAIGPNGDL---A--IGMYPF--------------------EWPPQAGCRNEE---MLDRG----NVYSRQRCNNGYCVIFYAYYFQKDT
    VDAG_JR2_Chr3g00800a-00001      ---GNADEIENKFQPIL---DFDTDGCYNTAAIDPDGNI---N--PGKGAT--------------------G-TPQGDCRDPP---QLENS----NVYSRRRCNNGVCAIMYEYYFEKDQ
    VDAG_JR2_Chr4g05950a-00001      VQTGAIGDAIAKFNPLL----HIANGCQPYTAVNDAGDT---S--GGLQDS--------------------G-NISAGCR------DQSKG----QTYARAKVVNGQLAIMYSFYMPKDQ
    VDAG_JR2_Chr8g10650a-00001      ---GSAWSEQEKWCPAL---DYDTDSCYNTVAISPSGQL---N--AGQDENK-----------------PAG-EILGWCRKEV---HLQQT----NIYVRSRCNNGWCVHMYDYYFEADF
    JR2_g2720.t1                    ---KRATENDLRYQPAL---DFDTDSCYNVPAIGCDGKI---A--EGLEPD--------------------G--TTKDYRDLA---DLDNT----NVYSRQRCNSGWCAYMYDYYFEKDH

    Selected Cols:                                                                                                                                          

    Gaps Scores:                                                                                                                                            
    Similarity Scores:                                                                                                                                      

                                           250       260       270       280       290       300       310       320       330       340       350       360
                                    =========+=========+=========+=========+=========+=========+=========+=========+=========+=========+=========+=========+
    12008_g358.t1                   PIAGN-VAGGHRHDWE----NVVVFVDDPAANAAPGLLGGAASGHGEYK-KTATPDRE----------------------GDSVKVEYFT--TFPTNHELQFTATTGK------------
    12008_g4461.t1                  SVSGS-FAGGHRHDWE----NVVVFARGDT------IVRVAPSCHGGYD-GASN------------EF---------PADGTSPQMVYHK--DSAGTHCFRFANDAD--IGGVENFSGSF
    12008_g7340.t1                  ------GWGAHRHDWE----HIAVWVQHGQ------LKFVSISQHGKWD-IRILDGRTAAPR---------------FEHGTHPKVVYHK--DGALTHAFRWANDGD---EPPENHWKSW
    12008_g7652.t1                  PSSGL----GHRHDWE----GAVVWLSSAAADAT--VVGVAASAHGDFD-VRRAADVSFA--------------------GARPKLGYRS--TWPVNHQMVFTADQGG------------
    12008_g7834.t1                  AT----PIDGHRHDWE----HIAVWVRQSDSF----VTHVAVSQHKGYD-IRENSQITWT-----------------AAENGKPAIVYHK--DSILTHCFRFGSGADAGGPGPENHKNQW
    12008_g8465.t1                  AWSWPVSGYNHRHDWE----HVVVWAKEGK------VRGVSVSQHGGYE-SRVAEDQRLRFDYTPKEFPYPAWDPMPTSVAMHPKVVFHK--DGARTHCFRFAKDSDD-YEGQENERGIW
    12008_g8848.t1                  PADGN-LASGHRHDWE----NVVIWFNSNNANQA-GILRGAASGHGDYK-KVNNPQRN----------------------NNNLHVEYFT--SLGKNHELQFKTSPGR------------
    85S_g58.t1                      ------GWGAHRHDWE----HIAVWVQHGQ------LKFVSISQHGKWD-IRILDGRTAAPR---------------FEHGTHPKVVYHK--DGALTHAFRWANDGD---EPPENHWKSW
    85S_g9168.t1                    PSSGL----GHRHDWE----GAVVWLSSAAADAT--VVGVAASAHGDFD-VRPAADVSFA--------------------GARPKLGYRS--TWPVNHQMVFTADQGG------------
    85S_g9863.t1                    SVSGS-FAGGHRHDWE----NVVVFARGDT------IVRVAPSCHGGYD-GASN------------EF---------PADGTSPQMVYHK--DSAGTHCFRFANDAD--IGGVENFSGSF
    85S_g7040.t1                    AWSWPVSGYNHRHDWE----HVVVWAKEGK------VRGVSVSQHGGYE-SRVAEDQRLRFDYTPKEFPYPAWDPMPTSVAMHPKVVFHK--DGARTHCFRFAKDSDD-YEGQENERGIW
    85S_g3154.t1                    PIAGN-VAGGHRHDWE----NVVVFVDDPAANAAPGLLGGAASGHGEYK-KTATPDRE----------------------GDSVKVEYFT--TFPTNHELQFTATTGK------------
    85S_g4608.t1                    AT----PIDGHRHDWE----HIAVWVRQSDSF----VTHVAVSQHKGYD-IRENSQITWT-----------------AAENGKPAIVYHK--DSILTHCFRFGSGADAGGPGPENHKNQW
    85S_g4744.t1                    PADGN-LASGHRHDWE----NVVIWFNSNNANQA-GILRGAASGHGDYK-KVNNPQRN----------------------NNNLHVEYFT--SLGKNHELQFKTSPGR------------
    CBS385.49_g66.t1                AT----PIDGHRHDWE----HIAVWVRQSDSF----VTHVAVSQHKGYD-IRENSQITWT-----------------AAENGKPSIVYHK--DSILTHCFRFGSGADAGGPGPENHKNQW
    CBS385.49_g189.t1               ---------GHRHDWE----NVVIWFNSNNANQA-GILRGAASGHGDYK-K------------------------------------YFT--SLGKNHELQFKTSPGR------------
    CBS385.49_g400.t1               PSSGL----GHRHDWE----GAVVWLSSAAADAT--VVGVAASAHGDFD-VRPAADVSFA--------------------GARPKLGYRS--TWPVNHQMVFTADQGG------------
    CBS385.49_g400.t2               PSSGL----GHRHDWE----GAVVWLSSAAADAT--VVGVAASAHGDFD-VRPAADVSFA--------------------GARPKLGYRS--TWPVNHQMVFTADQGG------------
    CBS385.49_g1381.t1              ------GWGAHRHDWE----HIAVWVQHGQ------LKFVSISQHGKWD-IRILDGRTAALR---------------FEHGTHPRSY-----------------------EPPENHWKSW
    CBS385.49_g5607.t1              PIAGN-VAGGHRHDWE----NIVVFVDDP-ANAAPGLLGGAASGHGEYK-KTATPDRE----------------------GDSVKVEYFT--TFPTNHELQFTATTGK------------
    CBS385.49_g7723.t1              -----------SITLK---GPVVVFARGDT------IVRVAPSCHGGYD-GASN------------EF---------PADGTSPQMVYHK--DSAGTHCFRFANDAD--IGGVENFSGSF
    CBS385.49_g8721.t1              AWSWPVSSYNHRHDWE----HVVVWAKEGK------VRGVSVSQHGGYE-SRVAEDQRLRFDYTPKEFPYPAWDPMPTSVAMHPKVVFHK--DGARTHCFRFAKDSDD-YEGQENERGVW
    CQ2_g254.t1                     AWSWPVSGYNHRHDWE----HVVVWAKEGK------VRGVSVSQHGGYE-SRVAEDQRLRFDYTPKEFPYPAWDPMPTSVAMHPKVVFHK--DGARTHCFRFAKDSDD-YEGQENERGIW
    CQ2_g2332.t3                    A-----DIGAHKHDWE----HIIVWVTDDRTKN---KKYACVSQHGEWL-CHPEDKVLWK--------------------DEHPKIVYHK--DWFQTRAFRFANAEDD--KYQENERHDW
    CQ2_g2332.t4                    A-----DIGAHKHDWE----HIIVWVTDDRTKN---KKYACVSQHGEWL-CHPEDKVLWK--------------------DEHPKIVYHK--DWFQTRAFRFANAEDD--KYQENERHDW
    CQ2_g3243.t1                    PSSGL----GHRHDWE----GAVVWLSSAAADAT--VVGVAASAHGDFD-VRPAADVSFA--------------------GARPKLGYRS--TWPVNHQMVFTADQGG------------
    CQ2_g3454.t1                    PADGN-LASGHRHDWE----NVVIWFNSNNANQA-GILRGAASGHGDYK-KVNNPQRN----------------------NNNLHVEYFT--SLGKNHELQFKTSPGR------------
    CQ2_g3584.t1                    AT----PIDGHRHDWE----HIAVWVRQSDSF----VTHVAVSQHKGYD-IRENSQITWT-----------------AAENGKPAIVYHK--DSILTHCFRFGSGADAGGPGPENHKNQW
    CQ2_g4742.t1                    SVSGS-FAGGHRHDWE----NVVVFARGDT------IVRVAPSCHGGYG-GASN------------EF---------PADGTSPQMVYHK--DSAGTHCFRFANDAD--IGGVENFSGSF
    CQ2_g7512.t1                    PIAGN-VAGGHRHDWE----NVVIFVDDPAANAAPGLLGGAASGHGEYK-KTATPDRE----------------------GDSVKVEYFT--TFPTNHELQFTATTGK------------
    CQ2_g8820.t1                    ------GWGAHRHDWE----HIAVWVQHGQ------LKFVSISQHGKWD-IRILDGRTAAPR---------------FEHGTHPKVVYHK--DGALTHAFRWANDGD---EPPENHWKSW
    DAR82592_g1639.t1               AWSWPVSGYNHRHDWE----HVVVWAKEGK------VRGVSVSQHGGYE-SRVAEDQRLRFDYTPKEFPYPAWDPMPTSVAMHPKVVFHK--DGARTHCFRFAKDSDD-YEGQENER---
    DAR82592_g8837.t1               ------GWGAHRHDWE----HIAVWVQHGQ------LKFVSISQHGKWD-IRILDGRTAAPR---------------FEHGTHPKVVYHK--DGALTHAFRWANDGD---EPPENHWKSW
    DAR82592_g4435.t1               PADGN-LASGHRHDWE----NVVIWFNSNNANQA-GILRGAASGHGDYK-KVNNPQRN----------------------NNNLHVEYFT--SLGKNHELQFKTSPGR------------
    DAR82592_g4647.t1               PSSGL----GHRHDWE----GAVVWLSSAAADAT--VVGVAASAHGDFD-VRPAADVSFA--------------------GARPKLGYRS--TWPVNHQMVFTADQGG------------
    DAR82592_g7268.t1               PIAGN-VAGGHRHDWE----NVVIFVDDPAANAAPGLLGGAASGHGEYK-KTATPDRE----------------------GDSVKVEYFT--TFPTNHELQFTATTGK------------
    DAR82592_g4307.t1               AT----PIDGHRHDWE----HIAVWVRQSDSF----VTHVAVSQHKGYD-IRENSQITWT-----------------AAENGKPAIVYHK--DSILTHCFRFGSGADAGGPGPENHKNQW
    DAR82592_g3508.t1               A-----DI---------------------------------------------EDKVLWK--------------------DEHPKIVYHK--DWFQTRAFRFANAEDD--KYQENERHDW
    DAR82592_g62.t1                 ----------------------IVFARGDT------IVRVAPSCHGGYG-GASN------------EF---------PADGTSPQMVYHK--DSAGTHCFRFANDAD--IGGVENFSGSF
    DAR83138_g1199.t1               AWSWPVSGYNHRHDWE----HVVVWAKEGK------VRGVSVSQHGGYE-SRVAEDQRLRFDYTPKEFPYPAWDPMPTSVAMHPKVVFHK--DGARTHCFRFAKDSDD-YEGQENERGIW
    DAR83138_g60.t1                 SVSGS-FAGGHRHDWE----NVVVFARGDT------IVRVAPSCHGGYG-GASN------------EF---------PADGTSPQMVYHK--DSAGTHCFRFANDAD--IGGVENFSGSF
    DAR83138_g7641.t1               PIAGN-VAGGHRHDWE----NVVIFVDDPAANAAPGLLGGAASGHGEYK-KTATPDRE----------------------GDSVKVEYFT--TFPTNHELQFTATTGK------------
    DAR83138_g9085.t1               ------GWGAHRHDWE----HIAVWVQHGQ------LKFVSISQHGKWD-IRILDGRTAAPR---------------FEHGTHPKVVYHK--DGALTHAFRWANDGD---EPPENHWKSW
    DAR83138_g3565.t3               A-----DIGAHKHDWE----HII---------------------HGEWL-CHPEDKVLWK--------------------DEHPKIVYHK--DWFQTRAFRFANAEDD--KYQENERHDW
    DAR83138_g4173.t1               AT----PIDGHRHDWE----HIAVWVRQSDSF----VTHVAVSQHKGYD-IRENSQITWT-----------------AAENGKPAIVYHK--DSILTHCFRFGSGADAGGPGPENHKNQW
    DAR83138_g4314.t1               PADGN-LASGHRHDWE----NVVIWFNSNNANQA-GILRGAASGHGDYK-KVNNPQRN----------------------NNNLHVEYFT--SLGKNHELQFKTSPGR------------
    DAR83138_g4535.t1               PSSGL----GHRHDWE----GAVVWLSSAAADAT--VVGVAASAHGDFD-VRPAADVSFA--------------------GARPKLGYRS--TWPVNHQMVFTADQGG------------
    DAR83143_g1427.t1               AWSWPVSGYNHRHDWE----HVVVWAKEGK------VRGVSVSQHGGYE-SRVAEDQRLRFDYTPKEFPYPAWDPMPTSVAMHPKVVFHK--DGARTHCFRFAKDSDD-YEGQENERGIW
    DAR83143_g4194.t1               AT----PIDGHRHDWE----HIAVWVRQSDSF----VTHVAVSQHKGYD-IRENSQITWT-----------------AAENGKPAIVYHK--DSILTHCFRFGSGADAGGPGPENHKNQW
    DAR83143_g4335.t1               PADGN-LASGHRHDWE----NVVIWFNSNNANQA-GILRGAASGHGDYK-KVNNPQRN----------------------NNNLHVEYFT--SLGKNHELQFKTSPGR------------
    DAR83143_g4545.t1               PSSGL----GHRHDWE----GAVVWLSSAAADAT--VVGVAASAHGDFD-VRPAADVSFA--------------------GARPKLGYRS--TWPVNHQMVFTADQGG------------
    DAR83143_g7287.t1               PIAGN-VAGGHRHDWE----NVVIFVDDPAANAAPGLLGGAASGHGEYK-KTATPDRE----------------------GDSVKVEYFT--TFPTNHELQFTATTGK------------
    DAR83143_g3521.t1               A-----DIGAHKHDWE----HIIVWVTDDRTKN---KKYACVSQHGEWL-CHPEDKVLWK--------------------DEHPKIVYHK--DWFQTRAFRFANAEDD--KYQENERHDW
    DAR83143_g8715.t1               ------GWGAHRHDWE----HIAVWVQHGQ------LKFVSISQHGKWD-IRILDGRTAAPR---------------FEHGTHPKVVYHK--DGALTHAFRWANDGD---EPPENHWKSW
    DAR83143_g61.t1                 SVSGS-FAGGHRHDWE----NVVVFARGDT------IVRVAPSCHGGYG-GASN------------EF---------PADGTSPQMVYHK--DSAGTHCFRFANDAD--IGGVENFSGSF
    DAR83175_g3551.t1               A-----DIGAHKHDWE----HIIVWVTDDRTKN---KKYACVSQHGEWL-CHPEDKVLWK--------------------DEHPKIVYHK--DWFQTRAFRFANAEDD--KYQENERHDW
    DAR83175_g4514.t1               PSSGL----GHRHDWE----GAVVWLSSAAADAT--VVGVAASAHGDFD-VRPAADVSFA--------------------GARPKLGYRS--TWPVNHQMVFTADQGG------------
    DAR83175_g4731.t1               PADGN-LASGHRHDWE----NVVIWFNSNNANQA-GILRGAASGHGDYK-KVNNPQRN----------------------NNNLHVEYFT--SLGKNHELQFKTSPGR------------
    DAR83175_g7439.t1               PIAGN-VAGGHRHDWE----NVVIFVDDPAANAAPGLLGGAASGHGEYK-KTATPDRE----------------------GDSVKVEYFT--TFPTNHELQFTATTGK------------
    DAR83175_g2075.t1               AWSWPVSGYNHRHDWE----HVVVWAKEGK------VRGVSVSQHGGYE-SRVAEDQRLRFDYTPKEFPYPAWDPMPTSVAMHPKVVFHK--DGARTHCFRFAKDSDD-YEGQENERGIW
    DAR83175_g5817.t1               AT----PIDGHRHDWE----HIAVWVRQSDSF----VTHVAVSQHKGYD-IRENSQITWT-----------------AAENGKPAIVYHK--DSILTHCFRFGSGADAGGPGPENHKNQW
    DAR83175_g61.t1                 SVSGS-FAGGHRHDWE----NVVVFARGDT------IVRVAPSCHGGYG-GASN------------EF---------PADGTSPQMVYHK--DSAGTHCFRFANDAD--IGGVENFSGSF
    DAR83175_g8890.t1               ------GWGAHRHDWE----HIAVWVQHGQ------LKFVSISQHGKWD-IRILDGRTAAPR---------------FEHGTHPKVVYHK--DGALTHAFRWANDGD---EPPENHWKSW
    DK045_g927.t1                   PSSGL----GHRHDWE----GAVVWLSSAAADAT--VVGVAASAHGDFD-VRPAADVSFA--------------------GARPKLGYRS--TWPVNHQMVFTADQGG------------
    DK045_g1138.t1                  PADGN-LASGHRHDWE----NVVIWFNSNNANQA-GILRGAASGHGDYK-KVNNPQRN----------------------NNNLHVEYFT--SLGKNHELQFKTSPGR------------
    DK045_g1272.t1                  AT----PIDGHRHDWE----HIAVWVRQSDSF----VTHVAVSQHKGYD-IRENSQITWT-----------------AAENGKPSIVYHK--DSILTHCFRFGSGADAGGPGPENHKNQW
    DK045_g2451.t1                  SVSGS-FAGGHRHDWE----NVVVFARGDT------IVRVAPSCHGGYD-GASN------------EF---------PADGTSPQMVYHK--DSAGTHCFRFANDAD--IGGVENFSGSF
    DK045_g3667.t1                  AWSWPVSSYNHRHDWE----HVVVWAKEGK------VRGVSVSQHGGYE-SRVAEDQRLRFDYTPKEFPYPAWDPMPTSVAMHPKVVFHK--DGARTHCFRFAKDSDD-YEGQENERGVW
    DK045_g5985.t1                  ------GWGAHRHDWE----HIAVWVQHGQ------LKFVSISQHGKWD-IRILDGRTAAPR---------------FEHGTHPKVVYHK--DGALTHAFRWANDGD---EPPENHWKSW
    DK045_g9032.t1                  PIAGN-VAGGHRHDWE----NIVVFVDDPAANAAPGLLGGAASGHGEYK-KTATPDRE----------------------GDSVKVEYFT--TFPTNHELQFTATTGK------------
    GF1192_g8987.t1                 PADGN-LASGHRHDWE----NVVIWFNSNNANQA-GILRGAASGHGDYK-KVNNPQRN----------------------NNNLHVEYFT--SLGKNHELQFKTSPGR------------
    GF1192_g9126.t1                 AT----PIDGHRHDWE----HIAVWVRQSDSF----VTHVAVSQHKGYD-IRENSQITWT-----------------AAENGKPSIVYHK--DSILTHCFRFGSGADAGGPGPENHKNQW
    GF1192_g257.t1                  AWSWPVSSYNHRHDWE----HVVVWAKEGK------VRGVSVSQHGGYE-SRVAEDQRLRFDYTPKEFPYPAWDPMPTSVAMHPKVVFHK--DGARTHCFRFAKDSDD-YEGQENERGVW
    GF1192_g1728.t1                 PIAGN-VAGGHRHDWE----NIVVFVDDPAANAAPGLLGGAASGHGEYK-KTATPDRE----------------------GDSVKVEYFT--TFPTNHELQFTATTGK------------
    GF1192_g2840.t1                 SVSGS-FAGGHRHDWE----NVVVFARGDT------IVRVAPSCHGGYD-GASN------------EF---------PADGTSPQMVYHK--DSAGTHCFRFANDAD--IGGVENFSGSF
    GF1192_g5025.t1                 PSSGL----GHRHDWE----GAVVWLSSAAADAT--VVGVAASAHGDFD-VRPAADVSFA--------------------GARPKLGYRS--TWPVNHQMVFTADQGG------------
    GF1192_g6069.t1                 ------GWGAHRHDWE----HIAVWVQHGQ------LKFVSISQHGKWD-IRILDGRTAAPR---------------FEHGTHPKVVYHK--DGALTHAFRWANDGD---EPPENHWKSW
    GF1207_g2093.t1                 AWSWPVSSYNHRHDWE----HVVVWAKEGK------VRGV-----------RVAEDQRLRFDYTPKEFPYPAWDPMPTSVAMHPKVVFHK--DGARTHCFRFAKDSDD-YEGQENERGVW
    GF1207_g4890.t1                 ---------------------TIVFARGDT------IVRVAPSCHGGYD-GASN------------EF---------PADGTSPQMVYHK--DSAGTHCFRFANDAD--IGGVENFSGSF
    GF1207_g5214.t1                 ------GWGAHRHDWE----HIAVWVQHGQ------LKFVSISQHGKWD-IRILDGRTAAPR---------------FEHGTHPRSY-----------------------EPPENHWKSW
    GF1207_g6669.t1                 PSSGL----GHRHDWE----GAVVWLSSAAADAT--VVGVAASAHGDFD-VRPAADVSFA--------------------GARPKLGYRS--TWPVNHQMVFTADQGG------------
    GF1207_g6876.t1                 PADGN-LASGHRHDWE----NVVIWFNSNNANQA-GILRGAASGHGDYK-KVNNPQRN----------------------NNNLHVEYFT--SLGKNHELQFKTSPGR------------
    GF1207_g6999.t1                 AT----PIDGHRHDWE----HIAVWVRQSDSF----VTHVAVSQHKGYD-IRENSQITWT-----------------AAENGKPAIVYHK--DSILTHCFRFGSGADAGGPGPENHKNQW
    GF1207_g7831.t1                 PIAGN-VAGGHRHDWE----NIVVFVDDPAANAAPGLLGGAASGHGEYK-KTATPDRE----------------------GDSVKVEYFT--TFPTNHELQFTATTGK------------
    GF1300_g9576.t1                 AT----PIDGHRHDWE----HIAVWVRQSDSF----VTHVAVSQHKGYD-IRENSQITWT-----------------AAENGKPSIVYHK--DSILTHCFRFGSGADAGGPGPENHKNQW
    GF1300_g9714.t1                 PADGN-LASGHRHDWE----NVVIWFNSNNANQA-GILRGAASGHGDYK-KVNNPQRN----------------------NNNLHVEYFT--SLGKNHELQFKTSPGR------------
    GF1300_g259.t1                  AWSWPVSSYNHRHDWE----HVVVWAKEGK------VRGVSVSQHGGYE-SRVAEDQRLRFDYTPKEFPYPAWDPMPTSVAMHPKVVFHK--DGARTHCFRFAKDSDD-YEGQENERGVW
    GF1300_g1512.t1                 SVSGS-FAGGHRHDWE----NVVVFARGDT------IVRVAPSCHGGYD-GASN------------EF---------PADGTSPQMVYHK--DSAGTHCFRFANDAD--IGGVENFSGSF
    GF1300_g3582.t1                 ------GWGAHRHDWE----HIAVWVQHGQ------LKFVSISQHGKWD-IRILDGRTAAPR---------------FEHGTHPKVVYHK--DGALTHAFRWANDGD---EPPENHWKSW
    GF1300_g3720.t1                 PSSGL----GHRHDWE----GAVVWLSSAAADAT--VVGVAASAHGDFD-VRPAADVSFA--------------------GARPKLGYRS--TWPVNHQMVFTADQGG------------
    GF1300_g6171.t1                 PIAGN-VAGGHRHDWE----NIVVFVDDPAANAAPGLLGGAASGHGEYK-KTATPDRE----------------------GDSVKVEYFT--TFPTNHELQFTATTGK------------
    Gf-Ca2_g111.t1                  ---------------------TIVFARGDT------IVRVAPSCHGGYD-GASN------------EF---------PADGTSPQMVYHK--DSAGTHCFRFANDAD--IGGVENFSGSF
    Gf-Ca2_g2398.t1                 AWSWPVSSYNHRHDWE----HVVVWAKEGK------VRGV-----------RVAEDQRLRFDYTPKEFPYPAWDPMPTSVAMHPKVVFHK--DGARTHCFRFAKDSDD-YEGQENERGVW
    Gf-Ca2_g3362.t1                 AT----PIDGHRHDWE----HIAVWVRQSDSF----VTHVAVSQHKGYD-IRENSQITWT-----------------AAENGKPAIVYHK--DSILTHCFRFGSGADAGGPGPENHKNQW
    Gf-Ca2_g3481.t1                 PADGN-LASGHRHDWE----NVVIWFNSNNANQA-GILRGAASGHGDYK-KVNNPQRN----------------------NNNLHVEYFT--SLGKNHELQFKTSPGR------------
    Gf-Ca2_g3687.t1                 PSSGL----GHRHDWE----GAVVWLSSAAADAT--VVGVAASAHGDFD-VRPAAD-----------------------------LGYRS--TWPVNHQMVFTADQGG------------
    Gf-Ca2_g4661.t1                 ------GWGAHRHDWE----HIAVWVQHGQ------LKFVSISQHGKWD-IRILDGRTAAPR---------------FEHGTHPKVVYHK--DGALTHAFRWANDGD---EPPENHWKSW
    Gf-Ca2_g8346.t1                 PIAGN-VAGGHRHDWE----NIVVFVDDPAANAAPGLLGGAASGHGEYK-KTATPDRE----------------------GDSVKVEYFT--TFPTNHELQFTATTGK------------
    Gf-Cb5_g260.t1                  AWSWPVSSYNHRHDWE----HVVVWAKEGK------VRGVSVSQHGGYE-SRVAEDQRLRFDYTPKEFPYPAWDPMPTSVAMHPKVVFHK--DGARTHCFRFAKDSDD-YEGQENERGVW
    Gf-Cb5_g3949.t1                 PADGN-LASGHRHDWE----NVVIWFNSNNANQA-GILRGAASGHGDYK-KVNNPQRN----------------------NNNLHVEYFT--SLGKNHELQFKTSPGR------------
    Gf-Cb5_g4084.t1                 AT----PIDGHRHDWE----HIAVWVRQSDSF----VTHVAVSQHKGYD-IRENSQITWT-----------------AAENGKPSIVYHK--DSILTHCFRFGSGADAGGPGPENHKNQW
    Gf-Cb5_g4605.t1                 PIAGN-VAGGHRHDWE----NIVVFVDDPAANAAPGLLGGAASGHGEYK-KTATPDRE----------------------GDSVKVEYFT--TFPTNHELQFTATTGK------------
    Gf-Cb5_g6249.t1                 A-----DIGAHKHDWE----HIIVWVTDDRTKN---------------------KKCSGK--------------------TNTPRLFTTR--IGFKLVPFALLMR-----KMINTRKMSA
    Gf-Cb5_g7167.t1                 SVSGS-FAGGHRHDWE----NVVVFARGDT------IVRVAPSCHGGYD-GASN------------EF---------PADGTSPQMVYHK--DSAGTHCFRFANDAD--IGGVENFSGSF
    Gf-Cb5_g7934.t1                 PSSGL----GHRHDWE----GAVVWLSSAAADAT--VVGVAASAHGDFD-VRPAADVSFA--------------------GARPKLGYRS--TWPVNHQMVFTADQGG------------
    Gf-Cb5_g8589.t1                 ------GWGAHRHDWE----HIAVWVQHGQ------LKFVSISQHGKWD-IRILDGRTAAPR---------------FEHGTHPKVVYHK--DGALTHAFRWANDGD---EPPENHWKSW
    HoMCF_g1490.t1                  ------GWGAHRHDWE----HIAVWVQHGQ------LKFVSISQHGKWD-IRILDGRTAAR---------------------------------------------D---EPPENHWKSW
    HoMCF_g2875.t1                  PIAGN-VAGGHRHDWE----NVVVF----------------------YK-KTATPDRE----------------------GDSVKVEYFT--TFPTNHELQ-------------------
    HoMCF_g3274.t1                  ---------GHRHDWE----NVVIWFNSNNANQA-GILRGAASGHGDYK-KVNNPQRN----------------------NNNLHVEYFT--SLGKNHELQFKTSPGR------------
    HoMCF_g4303.t1                  AWSWPVSGYNHRHDWE----HVVVWAKEGK------VRGVSVSQHGGYEPRRRGPEASLRLH--AQGVSVSCVGPHADERRHAPQGCLSQ---GRRPHCFRFAKDSDD-YEGQENERG--
    HoMCF_g5967.t1                  AT----PIDGHRHDWE----HIAVWVRQSDSF----VTHVAVSQHKGYD-IRENSQITWT-----------------AAENGKPAIVYHK--DSILTHCFRFGSGADAGGPGPENHKNQW
    HoMCF_g6093.t1                  PADGN-LASGHRHDWE----NVVIWFNSNNANQA-GILRGAASGHGDYK-KVNNPQRN----------------------NNNLHVEYFT--SLGKNHELQFKTSPGR------------
    HoMCF_g6096.t1                  ---------GHRHDWE----NVVIWFNSNNANQA-GILRGAASGHGDYK-KVNNPQRN----------------------NNNLHVEYFT--SLGKNHELQFKTSPGR------------
    HoMCF_g6549.t1                  PSSGL----GHRHDWE----GAVVWLSSAAADAT--VVGVAASAHGDFD------------------------------------LGYRS--TWPVNHQMVFTADQGG------------
    HoMCF_g10173.t1                 -----------SITLK----RTIVFARGDT------IVRVAPSCHGGYD-GASN------------EF---------PADGTSPQMVYHK--DSAGTHCFRFANDAD--IGGVD-FSGSF
    HoMCLT_g1190.t1                 AWSWPVSSYNHRHDWE----HVVVWAKEGK------VRGVSVSQHGGYE-SRVAEDQRLRFDYTPKEFPYPAWDPMPTSVAMHPKVVFHK--DGARTHCFRFAKDSDD-YEGQENERGVW
    HoMCLT_g2195.t1                 PIAGN-VAGGHRHDWE----NIVVFVDDPAANAAPGLLGGAASGHGEYK-KTATPDRE----------------------GDSVKVEYFT--TFPTNHELQFTATTGK------------
    HoMCLT_g2713.t1                 ------GWGAHRHDWE----HIAVWVQHGQ------LKFVSISQHGKWD-IRILDGRTAAPR---------------FEHGTHPKVVYHK--DGALTHAFRWANDGD---EPPENHWKSW
    HoMCLT_g3698.t1                 PSSGL----GHRHDWE----GAVVWLSSAAADAT--VVGVAASAHGDFD-VRPAADVSFA--------------------GARPKLGYRS--TWPVNHQMVFTADQGG------------
    HoMCLT_g4981.t1                 SVSGS-FAGGHRHDWE----NVVVFARGDT------IVRVAPSCHGGYD-GASN------------EF---------PADGTSPQMVYHK--DSAGTHCFRFANDAD--IGGVENFSGSF
    HoMCLT_g6335.t1                 PADGN-LASGHRHDWE----NVVIWFNSNNANQA-GILRGAASGHGDYK-KVNNPQRN----------------------NNNLHVEYFT--SLGKNHELQFKTSPGR------------
    HoMCLT_g6471.t1                 AT----PIDGHRHDWE----HIAVWVRQSDSF----VTHVAVSQHKGYD-IRENSQITWT-----------------AAENGKPSIVYHK--DSILTHCFRFGSGADAGGPGPENHKNQW
    I1V_g7945.t1                    PIAGN-VAGGHRHDWE----NVVIFVDDPAANAAPGLLGGAASGHGEYK-KTATPDRE----------------------GDSVKVEYFT--TFPTNHELQFTATTGK------------
    I1V_g9550.t1                    PADGN-LASGHRHDWE----NVVIWFNSNNANQA-GILRGAASGHGDYK-KVNNPQRN----------------------NNNLHVEYFT--SLGKNHELQFKTSPGR------------
    I1V_g9695.t1                    AWSWPVSGYNHRHDWE----HVVVWAKEGK------VRGVSVSQHGGYE-SRVAEDQRLRFDYTPKEFPYPAWDPMPTSVAMHPKVVFHK--DGARTHCFRFAKDSDD-YEGQENER---
    I1V_g10783.t1                   PSSGL----GHRHDWE----GAVVWLSSAAADAT--VVGVAA----------------FA--------------------GARPKLGYRS--TWPVNHQMVFTADQGG------------
    I1V_g12440.t1                   ------------------------WVRQSDSF----VTHVAVSQHKGYD-IRENSQITWT-----------------AAENGKPAIVYHK--DSILTHCFRFGSGADAGGPGPENHKNQW
    I1V_g13776.t1                   ------GWGAHRHDWE----HIAVWVQHGQ------LKFV----------------------------------------------VYHK--DGALTHAFRWANDGD---EPPENHWKSW
    MPI-CAGE_g5591.t1               SVSGS-FAGGHRHDWE----NVVVFARGDT------IVRVAPSCHGGYD-GASN------------EF---------PADGTSPQMVYHK--DSAGTHCFRFANDAD--IGGVENFSGSF
    MPI-CAGE_g8268.t1               PADGN-LASGHRHDWE----NVVIWFNSNNANQA-GILRGAASGHGDYK-KVNNPQRN----------------------NNNLHVEYFT--SLGKNHELQFKTSPGR------------
    MPI-CAGE_g8414.t1               AT----PIDGHRHDWE----HIAVWVRQSDSF----VTHVAVSQHKGYD-IRENSQITWT-----------------AAENGKPAIVYHK--DSILTHCFRFGSGADAGGPGPENHKNQW
    MPI-CAGE_g9956.t1               PSSGL----GHRHDWE----GAVVWLSSAAADAT--VVGVAASAHGDFD-VRRAADVSFA--------------------GARPKLGYRS--TWPVNHQMVFTADQGG------------
    MPI-CAGE_g1577.t1               AWSWPVSGYNHRHDWE----HVVVWAKEGK------VRGVSVSQHGGYE-SRVAEDQRLRFDYTPKEFPYPAWDPMPTSVAMHPKVVFHK--DGARTHCFRFAKDSDD-YEGQENERGIW
    MPI-CAGE_g2204.t1               A-----DIGAHKHDWE----HIIVWVTDDRTKN---KKYACVSQHGEWL-CHPEDKVLWK--------------------DEHPKIVYHK--DWFQTRAFRFANAEDD--KYQENERHDW
    MPI-CAGE_g10572.t1              AT----PIDGHRHDWE----HIAVWVRQSDSF----VTHVAVSQHKGYD-IRENSQITWT-----------------AAENGKPAIVYHK--DSILTHCFRFGSGADAGGPGPENHKNQW
    MPI-CAGE_g3967.t1               ------GWGAHRHDWE----HIAVWVQHGQ------LKFVSISQHGKWD-IRILDGRTAAPR---------------FEHGTHPKVVYHK--DGALTHAFRWANDGD---EPPENHWKSW
    MPI-CAGE_g4142.t1               PIAGN-VAGGHRHDWE----NVVVFVDDPAANAAPGLLGGAASGHGEYK-KTATPDRE----------------------GDSVKVEYFT--TFPTNHELQFTATTGK------------
    S011_g7486.t1                   A-----DIGAHKHDWE----HIIVWVTDDRTKN---KKYACVSQHGEWL-CHPEDKVLWK--------------------DEHPKIVYHK--DWFQTRAFRFANAEDD--KYQENERHDW
    S011_g8092.t1                   PIAGN-VAGGHRHDWE----NVVIFVDDPAANAAPGLLGGAASGHGD------------------------------------VKVEYFT--TFPTNHELQFTATTGK------------
    S011_g8657.t1                   AT----PIDGHRHDWE----HIAVWVRQSDSF----VTH-----------------ITWT-----------------AAENGKPAIVYHK--DSILTHCFRFGSGADAGGPGPENHKNQW
    S011_g8986.t1                   PSSGL----GHRHDWE----GAVVWLSSAAADAT--VVGVAASAHGDFD-VRPAADVSFA--------------------GARPKLGYRS--TWPVNHQMVFTADQGG------------
    S011_g9203.t1                   PADGN-LASGHRHDWE----NVVIWFNSNNANQA-GILRGAASGHGDYK-KVNNPQRN----------------------NNNLHVEYFT--SLGKNHELQFKTSPGR------------
    S011_g1207.t1                   AWSWPVSGYNHRHDWE----HVVVWAKEGK------VRGVSVSQHGGYE-SRVAEDQRLRFDYTPKDFPYPAWDPMPTSVAMHPKVVFHK--DGARTHCFRFAKDSDD-YEGQENERGVW
    S011_g2838.t1                   SVSGS-FAGGHRHDWE----NVVVFARGDT------IVRVAPSCHGGYG-GASN------------EF---------PADGTSPQMVYHK--DSAGTHCFRFANDAD--IGGVENFSGSF
    S011_g5817.t1                   ------GWGAHRHDWE----HIAVWVQHGQ------LKFVSISQHGKWD-IRILDGRTAAPR---------------FEHGTHPKVVYHK--DGALTHAFRWANDGD---EPPENHWKSW
    S023_g1908.t1                   AWSWPVSGYNHRHDWE----HVVVWAKEGK------VRGVSVSQHGGYE-SRVAEDQRLRFDYTPKEFPYPAWDPMPTSVAMHPKVVFHK--DGARTHCFRFAKDSDD-YEGQENERGIW
    S023_g3148.t1                   PSSGL----GHRHDWE----GAVVWLSSAAADAT--VVGVAASAHGDFD-VRRAADVSFA--------------------GARPKLGYRS--TWPVNHQMVFTADQGG------------
    S023_g4559.t1                   SVSGS-FAGGHRHDWE----NVVVFARGDT------IVRVAPSCHGGYD-GASN------------EF---------PADGTSPQMVYHK--DSAGTHCFRFANDAD--IGGVENFSGSF
    S023_g5760.t1                   AT----PIDGHRHDWE----HIAVWVRQSDSF----VTHVAVSQHKGYD-IRENSQITWT-----------------AAENGKPAIVYHK--DSILTHCFRFGSGADAGGPGPENHKNQW
    S023_g5904.t1                   PADGN-LASGHRHDWE----NVVIWFNSNNANQA-GILRGAASGHGDYK-KVNNPQRN----------------------NNNLHVEYFT--SLGKNHELQFKTSPGR------------
    S023_g7666.t1                   ------GWGAHRHDWE----HIAVWVQHGQ------LKFVSISQHGKWD-IRILDGRTAAPR---------------FEHGTHPKVVYHK--DGALTHAFRWANDGD---EPPENHWKSW
    S023_g9104.t1                   PIAGN-VAGGHRHDWE----NVVVFVDDPAANAAPGLLGGAASGHGEYK-KTATPDRE----------------------GDSVKVEYFT--TFPTNHELQFTATTGK------------
    TO22_g2077.t1                   AWSWPVSSYNHRHDWE----HVVVWAKEGK------VRGVSVSQHGGYE-SRVAEDQRLRFDYTPKEFPYPAWDPMPTSVAMHPKVVFHK--DGARTHCFRFAKDSDD-YEGQENERGVW
    TO22_g2405.t1                   AT----PIDGHRHDWE----HIAVWVRQSDSF----VTHVAVSQHKGYD-IRENSQITWT-----------------AAENGKPAIVYHK--DSILTHCFRFGSGADAGGPGPENHKNQW
    TO22_g2541.t1                   PADGN-LASGHRHDWE----NVVIWFNSNNANQA-GILRGAASGHGDYK-KVNNPQRN----------------------NNNLHVEYFT--SLGKNHELQFKTSPGR------------
    TO22_g2749.t1                   PSSGL----GHRHDWE----GAVVWLSSAAADAT--VVGVAASAHGDFD-VRPAADVSFA--------------------GARPKLGYRS--TWPVNHQMVFTADQGG------------
    TO22_g3776.t1                   SVSGS-FAGGHRHDWE----NVVVFARGDT------IVRVAPSCHGGYD-GASN------------EF---------PADGTSPQMVYHK--DSAGTHCFRFANDAD--IGGVENFSGSF
    TO22_g6379.t1                   PIAGN-VAGGHRHDWE----NIVVFVDDPAANAAPGLLGGAASGHGEYK-KTATPDRE----------------------GDSVKVEYFT--TFPTNHELQFTATTGK------------
    TO22_g9457.t1                   ------GWGAHRHDWE----HIAVWVQHGQ------LKFVSISQHGKWD-IRILDGRTAAPR---------------FEHGTHPKVVYHK--DGALTHAFRWANDGD---EPPENHWKSW
    Ud1-4-1_g9889.t1                ------GWGAHRHDWE----HIAVWVQHGQ------LKFVSISQHGKWD-IRILDGRTAAPR---------------FEHGTHPKVVYHK--DGALTHAFRWANDGD---EPPENHWKSW
    Ud1-4-1_g261.t1                 AWSWPVSSYNHRHDWE----HVVVWAKEGK------VRGVSVSQHGGYE-SRVAEDQRLRFDYTPKEFPYPAWDPMPTSVAMHPKVVFHK--DGARTHCFRFAKDSDD-YEGQENERGVW
    Ud1-4-1_g2536.t1                SVSGS-FAGGHRHDWE----NVVVFARGDT------IVRVAPSCHGGYD-GASN------------EF---------PADGTSPQMVYHK--DSAGTHCFRFANDAD--IGGVENFSGSF
    Ud1-4-1_g2721.t1                AT----PIDGHRHDWE----HIAVWVRQSDSF----VTHVAVSQHKGYD-IRENSQITWT-----------------AAENGKPAIVYHK--DSILTHCFRFGSGADAGGPGPENHKNQW
    Ud1-4-1_g2858.t1                PADGN-LASGHRHDWE----NVVIWFNSNNANQA-GILRGAASGHGDYK-KVNNPQRN----------------------NNNLHVEYFT--SLGKNHELQFKTSPGR------------
    Ud1-4-1_g3064.t1                PSSGL----GHRHDWE----GAVVWLSSAAADAT--VVGVAASAHGDFD-VRPAADVSFA--------------------GARPKLGYRS--TWPVNHQMVFTADQGG------------
    Ud1-4-1_g3975.t1                A-----DIGAHKHDWE----HIIVWVTDDRTKN---KKYACVSQHGEWL-CHPEDKVLWK--------------------DEHPKIVYHK--DWFQTRAFRFANAEDD--KYQENERHDW
    Ud1-4-1_g4565.t1                PIAGN-VAGGHRHDWE----NIVVFVDDPAANAAPGLLGGAASGHGEYK-KTATPDRE----------------------GDSVKVEYFT--TFPTNHELQFTATTGK------------
    V13_g3278.t1                    -------------------------ASASDSF----VTHVAVSQHKGCD-IRENSQITWT-----------------AAENGKPAIVYHK--DSILTHCFRFGSGADAGGPGPENHKNQW
    V13_g3397.t1                    PPP----------RLE----NVVIWFNSNNANQA-GILRGAASSHGDYK-KVNNPQRN----------------------NNNLHVRYFT--SLGKNHELQFKTSPGR------------
    V13_g4590.t1                    GQRGR-RAPA--RLGE----RCRLRRRPGGQRGA-GLLGGAASGHGEYK--TATPDRE----------------------GDSVKVEYFT--TFPTNHELQFTATTGK------------
    V13_g5067.t1                    ------------------------------------------------------------------RF---------PADGTSPQMVYHK--DSAGTHCFRFANDAD--IGGVENSQARS
    V13_g6708.t1                    ------GWGAHRHDWE----HIAVWVQHGQ------LKFVSISQHGKWD-IRILDGRTAAR-------------------G---------------------------------SSMAPT
    V13_g9344.t1                    A-----DIGAHKHDWE----HII-------------------------------DKVLWK--------------------DEHPKIVYHK--DWFQTRAFRFANAEDD--KYQENERHDW
    V13_g9739.t1                    PSSGL----GHQHDWE----GAVVWLSSAAADAT--VVGVAASAHGDFD-VRRAADVSFA---------------------ARGQARLPQ--HLACQPPDGLHGGPGR------------
    V13_g10958.t1                   AWSWPVSGYNHRHDWE----HVVVWAKEGK------VRGVSVSQHGGYE-SRVAEDQRLRFDYTPKEFP-------------------------------------DD-YEGQENER---
    Vd39_g2112.t1                   A-----DIGAHKHDWE----HIIVWVTDDRTKN---KKYACVSQHGEWL-CHPEDKVLWK--------------------DEHPKIVYHK--DWFQTRAFRFANAEDD--KYQENERHDW
    Vd39_g2112.t3                   A-----DIGAHKHDWE----HIIVWVTDDRTKN---KKYACVSQHGEWL-CHPEDKVLWK--------------------DEHPKIVYHK--DWFQTRAFRFANAEDD--KYQENERHDW
    Vd39_g2716.t1                   AWSWPVSGYNHRHDWE----HVVVWAKEGK------VRGVSVSQHGGYE-SRVAEDQRLRFDYTPKDFPYPAWDPMPTSVAMHPKVVFHK--DGARTHCFRFAKDSDD-YEGQENERGVW
    Vd39_g3506.t1                   AT----PIDGHRHDWE----HIAVWVRQSDSF----VTHVA---------------ITWT-----------------AAENGKPAIVYHK--DSILTHCFRFGSGADAGGPGPENHKNQW
    Vd39_g3907.t1                   ------GWGAHRHDWE----HIAVWVQHGQ------LKFVSISQHGKWD-IRILDGRTAAPR---------------FEHGTHPKVVYHK--DGALTHAFRWANDGD---EPPENHWKSW
    Vd39_g4379.t1                   SVSGS-FAGGHRHDWE----NVVVFARGDT------IVRVAPSCHGGYG-GASN------------EF---------PADGTSPQMVYHK--DSAGTHCFRFANDAD--IGGVENFSGSF
    Vd39_g7329.t1                   PIAGN-VAGGHRHDWE----NVVVFVDDPAANAAPGLLGGAASGHGEYK-KTATPDRE----------------------GDSVKVEYFT--TFPTNHELQFTATTGK------------
    Vd39_g8964.t1                   PSSGL----GHRHDWE----GAVVWLSSAAADAT--VVGVAASAHGDFD-VRPAADVSFA--------------------GARPKLGYRS--TWPVNHQMVFTADQGG------------
    Vd39_g9298.t1                   PADGN-LASGHRHDWE----NVVIWFNSNNANQA-GILRGAASGHGDYK-KVNNPQRN----------------------NNNLHVEYFT--SLGKNHELQFKTSPGR------------
    Vd-653_g1291.t1                 PADGN-LASGHRHDWE----NVVIWFNSNNANQA-GILRGAASGHGDYK-K------------------------------------YFT--SLGKNHELQFKTSPGR------------
    Vd-653_g2557.t1                 AWSWPVSGYNHRHDWE----HVVVWAKEGK------VRGVSVSQHGGYE-SRVAEDQRLRFDYTPKEFPYPATAP---------------------AHCFRFAKDSDD-YEGQENERGVW
    Vd-653_g3339.t1                 ------GWGAHRHDWE----HIAVWVQHGQ------LKFVSISQHGKWD-IRILDGRTAAPR---------------FEHGTHPK--------------------------PPENHWKSW
    Vd-653_g3685.t1                 ------------------------------------------------------------------------------------------------------------------------
    Vd-653_g3686.t1                 PSSGL----GHRHDWE----GAVVWLSSAAADAT--VVGVAASAHGDFD-VRPAAD-----------------------------LGYRS--TWPVNHQMVFTADQGG------------
    Vd-653_g5777.t1                 AT----PIDGHRHDWE----HIAVWVRQSDSF----VTHVAVSQHKGYD-IRENSQITWT-----------------AAENGKPSIWRRR--RR----------------SGPENHKNQW
    Vd-653_g8550.t1                 PIAGN-VAGGHRHDWE----NIVVFVDDPAANAAPG-LGGAASGHGEYK-KTATPDRE----------------------GDS------------------FTATTGK------------
    Vd-653_g9566.t1                 ----------------------IVFARGDT------IVRVAPSCHGGYD-GASN------------EF---------PADGTSPQMVYHK--DSAGTHCFRFANDAD--IGGVENSQARS
    VD991_g2070.t1                  AWSWPVSGYNHRHDWE----HVVVWAKEGK------VRGVSVSQHGGYE-SRVAEDQRLRFDYTPKEFPYPAWDPMPTSVAMHPKVVFHK--DGARTHCFRFAKDSDD-YEGQENERGIW
    VD991_g2394.t1                  AT----PIDGHRHDWE----HIAVWVRQSDSF----VTHVAVSQHKGYD-IRENSQITWT-----------------AAENGKPAIVYHK--DSILTHCFRFGSGADAGGPGPENHKNQW
    VD991_g2531.t1                  PADGN-LASGHRHDWE----NVVIWFNSNNANQA-GILRGAASGHGDYK-KVNNPQRN----------------------NNNLHVEYFT--SLGKNHELQFKTSPGR------------
    VD991_g2742.t1                  PSSGL----GHRHDWE----GAVVWLSSAAADAT--VVGVAASAHGDFD-VRPAADVSFA--------------------GARPKLGYRS--TWPVNHQMVFTADQGG------------
    VD991_g3653.t1                  A-----DIGAHKHDWE----HIIVWVTDDRTKN---KKYACVSQHGEWL-CHPEDKVLWK--------------------DEHPKIVYHK--DWFQTRAFRFANAEDD--KYQENERHDW
    VD991_g3653.t2                  A-----DIGAHKHDWE----HIIVWVTDDRTKN---KKYACVSQHGEWL-CHPEDKVLWK--------------------DEHPKIVYHK--DWFQTRAFRFANAEDD--KYQENERHDW
    VD991_g3719.t1                  SVSGS-FAGGHRHDWE----NVVVFARGDT------IVRVAPSCHGGYG-GASN------------EF---------PADGTSPQMVYHK--DSAGTHCFRFANDAD--IGGVENFSGSF
    VD991_g7479.t1                  PIAGN-VAGGHRHDWE----NVVIFVDDPAANAAPGLLGGAASGHGEYK-KTATPDRE----------------------GDSVKVEYFT--TFPTNHELQFTATTGK------------
    VD991_g8969.t1                  ------GWGAHRHDWE----HIAVWVQHGQ------LKFVSISQHGKWD-IRILDGRTAAPR---------------FEHGTHPKVVYHK--DGALTHAFRWANDGD---EPPENHWKSW
    VdB09_g1055.t1                  AT----PIDGHRHDWE----HIAVWVRQSDSF----VTHVAVSQHKGYD-IRENSQITWT-----------------AAENGKPAIVYHK--DSILTHCFRFGSGADAGGPGPENHKNQW
    VdB09_g1685.t1                  ------GWGAHRHDWE----HIAVWVQHGQ------LKFVSISQHGKWD-IRILDGRTAAPR---------------FEHGTHPKVVYHK--DGALTHAFRWANDGD---EPPENHWKSW
    VdB09_g1751.t1                  ----------------------IVFARGDT------IVRVAPSCHGGYD-GASN------------EF---------PADGTSPQMVYHK--DSAGTHCFRFANDAD--IGGVENFSGSF
    VdB09_g3918.t1                  A-----DIGAHKHDWE----HII---------------------HGEWL-CHPEDKVLWK--------------------DEHPKIVYHK--DWFQTRAFRFANAEDD--KYQENERHDW
    VdB09_g4877.t1                  PSSGL----GHRHDWE----GAVVWLSSAAADAT--VVGVAASAHGDFD-VRPAADVSFA--------------------GARPKLGYRS--TWPVNHQMVFTADQGG------------
    VdB09_g5096.t1                  PADGN-LASGHRHDWE----NVVIWFNSNNANQA-GILRGAASGHGDYK-KVNNPQRN----------------------NNNLHVEYFT--SLGKNHELQFKTSPGR------------
    VdB09_g9703.t1                  PIAGN-VAGGHRHDWE----NIVVFVDDPAANAAPGLLGGAASGHGEYK-KTATPDRE----------------------GDSVKVEYFT--TFPTNHELQFTATTGK------------
    VdB09_g7661.t1                  AWSWPVSSYNHRHDWE----HVVVWAKEGK------VRGVSVSQHGGYE-SRVAEDQRLRFDYTPKEFPYPAWDPMPTSVAMHPKVVFHK--DGARTHCFRFAKDSDD-YEGQENER---
    VdC07_g4126.t1                  AT----PIDGHRHDWE----HIAVWVRQSDSF----VTHVAVSQHKGYD-IRENSQITWT-----------------AAENGKPAIVYHK--DSILTHCFRFGSGADAGGPGPENHKNQW
    VdC07_g4261.t1                  PADGN-LASGHRHDWE----NVVIWFNSNNANQA-GILRGAASGHGDYK-KVNNPQRN----------------------NNNLHVEYFT--SLGKNHELQFKTSPGR------------
    VdC07_g4482.t1                  PSSGL----GHRHDWE----GAVVWLSSAAADAT--VVGVAASAHGDFD-VRPAADVSFA--------------------GARPKLGYRS--TWPVNHQMVFTADQGG------------
    VdC07_g605.t1                   PIAGN-VAGGHRHDWE----NIVVFVDDPAANAAPGLLGGAASGHGEYK-KTATPDRE----------------------GDSVKVEYFT--TFPTNHELQFTATTGK------------
    VdC07_g1872.t1                  ------GWGAHRHDWE----HIAVWVQHGQ------LKFVSISQHGKWD-IRILDGRTAAPR---------------FEHGTHPKVVYHK--DGALTHAFRWANDGD---EPPENHWKSW
    VdC07_g2986.t1                  SVSGS-FAGGHRHDWE----NVVVFARGDT------IVRVAPSCHGGYD-GASN------------EF---------PADGTSPQMVYHK--DSAGTHCFRFANDAD--IGGVENFSGSF
    VdC07_g7672.t1                  AWSWPVSSYNHRHDWE----HVVVWAKEGK------VRGVSVSQHGGYE-SRVAEDQRLRFDYTPKEFPYPAWDPMPTSVAMHPKVVFHK--DGARTHCFRFAKDSDD-YEGQENERGVW
    Vd-H5_g2477.t1                  PSSGL----GHRHDWE----GAVVWLSSAAADAT--VVGVAASAHGDFD-VRPAADVSFA--------------------GARPKLGYRS--TWPVNHQMVFTADQGG------------
    Vd-H5_g5176.t1                  ------GWGAHRHDWE----HIAVWVQHGQ------LKFVSISQHGKWD-IRILDGRTAAR---------------------------------------------D---EPPENHWKSW
    Vd-H5_g6144.t1                  ----------------------IVFARGDT------IVRVAPSCHGGYD-GASN------------EF---------PADGTSPQMVYHK--DSAGTHCFRFANDAD--IGGVENFSGSF
    Vd-H5_g7203.t1                  PIAGN-VAGGHRHDWE----NIVVFVDDPAANAAPG-LGGAASGHGEYK-KTATPDRE----------------------GDSVKVEYFT--TFPTNHELQFTATTGK------------
    Vd-H5_g8423.t1                  AT----PIDGHRHDWE----HIAVWVRQSDSF----VTHVAVSQHKGYD-IRENSQITWT-----------------AAENGKPSIVYHK--DSILTHCFRFGSGADAGGPGPENHKNQW
    Vd-H5_g8546.t1                  PADGN-LASGHRHDWE----NVVIWFNSNNANQA-GILRGAASGHGDYK-KVNNPQRN----------------------NNNLHVEYFT--SLGKNHELQFKTSPGR------------
    Vd-H5_g9396.t1                  AWSWPVSGYNHRHDWE----HVVVWAKEGK------VRGVSVSQHGGYE-SRVAEDQRLRFDYTPKEFPYPAWDPMPTSVAMHPKVVFHK--DGARTHCFRFAKDSDD-YEGQENERG--
    VdLs16_g2071.t1                 AWSWPVSSYNHRHDWE----HVVVWAKEGK------VRGVSVSQHGGYE-SRVAEDQRLRFDYTPKEFPYPAWDPMPTSVAMHPKVVFHK--DGARTHCFRFAKDSDD-YEGQENERGVW
    VdLs16_g7836.t1                 PIAGN-VAGGHRHDWE----NIVVFVDDPAANAAPGLLGGAASGHGEYK-KTATPDRE----------------------GDSVKVEYFT--TFPTNHELQFTATTGK------------
    VdLs16_g8455.t1                 SVSGS-FAGGHRHDWE----NVVVFARGDT------IVRVAPSCHGGYD-GASN------------EF---------PADGTSPQMVYHK--DSAGTHCFRFANDAD--IGGVENFSGSF
    VdLs16_g5349.t1                 PSSGL----GHRHDWE----GAVVWLSSAAADAT--VVGVAASAHGDFD-VRPAADVSFA--------------------GARPKLGYRS--TWPVNHQMVFTADQGG------------
    VdLs16_g5560.t1                 PADGN-LASGHRHDWE----NVVIWFNSNNANQA-GILRGAASGHGDYK-KVNNPQRN----------------------NNNLHVEYFT--SLGKNHELQFKTSPGR------------
    VdLs16_g5699.t1                 AT----PIDGHRHDWE----HIAVWVRQSDSF----VTHVAVSQHKGYD-IRENSQITWT-----------------AAENGKPAIVYHK--DSILTHCFRFGSGADAGGPGPENHKNQW
    VdLs16_g6952.t1                 ------GWGAHRHDWE----HIAVWVQHGQ------LKFVSISQHGKWD-IRILDGRTAAPR---------------FEHGTHPKVVYHK--DGALTHAFRWANDGD---EPPENHWKSW
    VdLs17_g64.t1                   SVSGS-FAGGHRHDWE----NVVVFARGDT------IVRVAPSCHGGYD-GASN------------EF---------PADGTSPQMVYHK--DSAGTHCFRFANDAD--IGGVENFSGSF
    VdLs17_g1975.t1                 AWSWPVSGYNHRHDWE----HVVVWAKEGK------VRGVSVSQHGGYE-SRVAEDQRLRFDYTPKEFPYPAWDPMPTSVAMHPKVVFHK--DGARTHCFRFAKDSDD-YEGQENERGIW
    VdLs17_g3499.t1                 A-----DIGAHKHDWE----HIIVWVTDDRTKN---KKYACVSQHGEWL-CHPEDKVLWK--------------------DEHPKIVYHK--DWFQTRAFRFANAEDD--KYQENERHDW
    VdLs17_g4253.t1                 ---------AHRHDFE----NIVVFVDDPAVNPIPAILGGAASGHGEYK-TTATPDVE----------------------GDSVKVEYFT--TFLTNHELQFTATSGK------------
    VdLs17_g4391.t1                 PADGN-LASGHRHDWE----NVVIWFNSNNANQA-GILRGAASGHGDYK-KVNNPQRN----------------------NNNLHVEYFT--SLGKNHELQFKTSPGR------------
    VdLs17_g5246.t1                 AT----PIDGHRHDWE----HIAVWVRQSDSF----VTHVAVSQHKGYD-IRENSQITWT-----------------AAENGKPAIVYHK--DSILTHCFRFGSGADAGGPGPENHKNQW
    VdLs17_g6770.t1                 PIAGN-VAGGHRHDWE----NVVVFVDDPAANAAPGLLGGAASGHGEYK-KTATPDRE----------------------GDSVKVEYFT--TFPTNHELQFTATTGK------------
    VdLs17_g8273.t1                 ------GWGAHRHDWE----HIAVWVQHGQ------LKFVSISQHGKWD-IRILDGRTAAPR---------------FEHGTHPKVVYHK--DGALTHAFRWANDGD---EPPENHWKSW
    VdLs17_g8809.t1                 PSSGL----GHRHDWE----GAVVWLSSAAADAT--VVGVAASAHGDFD-VRRAADVSFA--------------------GARPKLGYRS--TWPVNHQMVFTADQGG------------
    Vd-R1_g926.t1                   PSSGL----GHRHDWE----GAVVWLSSAAADAT--VVGVAASAHGDFD-VRPAADVSFA--------------------GARPKLGYRS--TWPVNHQMVFTADQGG------------
    Vd-R1_g3679.t1                  ----------------------IVFARGDT------IVRVAPSCHGGYD-GASN------------EF---------PADGTSPQMVYHK--DSAGTHCFRFANDAD--IGGVENSQARS
    Vd-R1_g4718.t1                  PIAGN-VAGGHRHDWE----NIVVFVDDPAANAAPGLLGGAASGHGEYK-KTATPDRE----------------------GDSVKVEYFT--TFPTNHELQFTATTGK------------
    Vd-R1_g5037.t1                  ------GWGAHRHDWE----HIAVWVQHGQ------LKFVSISQHGKWD-IRILDGRTAAR-------------------------------------GSSMAPTQD---EPPENHWKSW
    Vd-R1_g8886.t1                  AWSWPVSGYNHRHDWE----HVVVWAKEGK------VRGVSVSQHGGYE-SRVAEDQRLRFDYTPKEFPYPAWDPMPTSVAMHPKVVFHK--DGARTHCFRFAKDSDD-YEGQENERGVW
    Vd-R1_g9491.t1                  ---------GHRHDWE----NVVIWFNSNNANQA-GILRGAASGHGDYK-KVNNPQRN----------------------NNNLHVEYFT--SLGKNHELQFKTSPGR------------
    Vd-R1_g9617.t1                  AT----PIDGHRHDWE----HIAVWVRQSDSF----VTHVAVSQHKGYD-IRENSQITWT-----------------AAENGKPSIVYHK--DSILTHCFRFGSGADAGGPGPENHKNQW
    VT-2A_g1161.t1                  ----------------------IVFARGDT------IVRVAPSCHGGYD-GASN------------EF---------PADGTSPQMVYHK--DSAGTHCFRFANDAD--IGGVENFSGSF
    VT-2A_g1267.t1                  ------GWGAHRHDWE----HIAVWVQHGQ------LKFVSISQHGKWD-IRILDGRTAAR---------------------------------------------D---EPPENHWKSW
    VT-2A_g4726.t1                  PIAGN-VAGGHRHDWE----NIVVFVDDPAANAAPGLLGGAASGHGEYK-KTATPDRE----------------------GDSVKVEYFT--TFPTNHELQFTATTGK------------
    VT-2A_g6128.t1                  PSSGL----GHRHDWE----GAVVWLSSAAADAT--VVGVAASAHGDFD-VRPAADVSFA--------------------GARPKLGYRS--TWPVNHQMVFTADQGG------------
    VT-2A_g6128.t2                  PSSGL----GHRHDWE----GAVVWLSSAAADAT--VVGVAASAHGDFD-VRPAADVSFA--------------------GARPKLGYRS--TWPVNHQMVFTADQGG------------
    VT-2A_g6341.t1                  PADGN-LASGHRHDWE----NVVIWFNSNNANQA-GILRGAASGHGDYK-KVNNPQRN----------------------NNNLHVEYFT--SLGKNHELQFKTSPGR------------
    VT-2A_g6470.t1                  AT----PIDGHRHDWE----HIAVWVRQSDSF----VTHVAVSQHKGYD-IRENSQITWT-----------------AAENGKPSIVYHK--DSILTHCFRFGSGADAGGPGPENHKNQW
    VT-2A_g9510.t1                  ---------------------------------------------------------------------------------MHPKVVFHK--DGARTHCFRFAKDSDD-YEGQENERGVW
    VT-2A_g9511.t1                  AWSWPVSSYNHRHDWE----HVVVWAKEGK------VRGVSVSQHGGYE-SRVAEDQRLRFDYTPKEFPYPAWDPMPTSVAMHPKVVFHK--DGARTHCFRFAKDSDD-YEGQENERGVW
    XJ511_g1085.t1                  AWSWPVSGYNHRHDWE----HVVVWAKEGK------VRGVSVSQHGGYE-SRVAEDQRLRFDYTPKEFPYPAGDPMPTSVAMHPKVVFHK--DGARTHCFRFAKDSDD-YEGQENERGIW
    XJ511_g1518.t1                  SVSGS-FAGGHRHDWE----NVVVFARGDT------IVRVAPSCHGGYD-GASN------------EF---------PADGTSPQMVYHK--DSAGTHCFRFANDAD--IGGVENFSGSF
    XJ511_g4592.t1                  ------GWGAHRHDWE----HIAVWVQHGQ------LKFVSISQHGKWD-IRILDGRTAAPR---------------FEHGTHPKVVYHK--DGALTHAFRWANDGD---EPPENHWKSW
    XJ511_g5794.t1                  AT----PIDGHRHDWE----HIAVWVRQSDSF----VTHVAVSQHKGYD-IRENSQITWT-----------------AAENGKPAIVYHK--DSILTHCFRFGSGADAGGPGPENHKNQW
    XJ511_g5932.t1                  PADGN-LASGHRHDWE----NVVIWFNSNNANQA-GILRGAASGHGDYK-KVNNPQRN----------------------NNNLHVEYFT--SLGKNHELQFKTSPGR------------
    XJ511_g7291.t1                  PIAGN-VAGGHRHDWE----NVVVFVDDPAANAAPGLLGGAASGHGEYK-KTATPDRE----------------------GDSVKVEYFT--TFPTNHELQFTATTGK------------
    XJ511_g8237.t1                  PSSGL----GHRHDWE----GAVVWLSSAAADAT--VVGVAASAHGDFD-VRRAADVSFA--------------------GARPKLGYRS--TWPVNHQMVFTADQGG------------
    XJ592_g261.t1                   AWSWPVSGYNHRHDWE----HVVVWAKEGK------VRGVSVSQHGGYE-SRVAEDQRLRFDYTPKEFPYPAWDPMPTSVAMHPKVVFHK--DGARTHCFRFAKDSDD-YEGQENERGIW
    XJ592_g3433.t1                  SVSGS-FAGGHRHDWE----NVVVFARGDT------IVRVAPSCHGGYG-GASN------------EF---------PADGTSPQMVYHK--DSAGTHCFRFANDAD--IGGVENFSGSF
    XJ592_g5005.t1                  PIAGN-VAGGHRHDWE----NVVIFVDDPAANAAPGLLGGAASGHGEYK-KTATPDRE----------------------GDSVKVEYFT--TFPTNHELQFTATTGK------------
    XJ592_g6487.t1                  ------GWGAHRHDWE----HIAVWVQHGQ------LKFVSISQHGKWD-IRILDGRTAAPR---------------FEHGTHPKVVYHK--DGALTHAFRWANDGD---EPPENHWKSW
    XJ592_g7860.t1                  PSSGL----GHRHDWE----GAVVWLSSAAADAT--VVGVAASAHGDFD-VRPAADVSFA--------------------GARPKLGYRS--TWPVNHQMVFTADQGG------------
    XJ592_g8068.t1                  PADGN-LASGHRHDWE----NVVIWFNSNNANQA-GILRGAASGHGDYK-KVNNPQRN----------------------NNNLHVEYFT--SLGKNHELQFKTSPGR------------
    XJ592_g8206.t1                  AT----PIDGHRHDWE----HIAVWVRQSDSF----VTHVAVSQHKGYD-IRENSQITWT-----------------AAENGKPAIVYHK--DSILTHCFRFGSGADAGGPGPENHKNQW
    XJ592_g9637.t1                  A-----DIGAHKHDWE----HIIVWVTDDRTKN---KKYACVSQHGEWL-CHPEDKVLWK--------------------DEHPKIVYHK--DWFQTRAFRFANAEDD--KYQENERHDW
    XJ592_g9637.t2                  A-----DIGAHKHDWE----HIIVWVTDDRTKN---KKYACVSQHGEWL-CHPEDKVLWK--------------------DEHPKIVYHK--DWFQTRAFRFANAEDD--KYQENERHDW
    397_g2445.t1                    GTGSL-GFIGHRHDWE----CVVVWVKNPAVANP-EVVGISTSAHGKFT-KVPGAIKDVM------------------PGTRHPKIKYYQDVNFFGTHSVGTTGNEGR------------
    397_g4853.t1                    PIAGN-VPGGHRHDFE----NIVVFVDDPAANPTPAILGGAASGHGEYK-KTATPDVE----------------------GDSVKVEYFT--TFPTNHELQFTATSGK------------
    397_g6363.t1                    ------GTGVHRHDWE----HIAVWVQNGQ------LKFVSISMHGDWD-IRFAEGGDIAPR---------------FEYGTHAKVVYHK--DGPYTHAFRWANGGD---EPPENHWQSW
    397_g7397.t1                    SVSGT-FAGGHRHDWE----NVVVFARGDT------IVRVAPSCHGGYG-GASN------------EF---------PADGTSPQMVYHK--DGAGTHCFRFANGAD--IGGVENFSGSF
    397_g7755.t1                    AWSWPIDGYNHRHDWE----HVVVWAKLGK------VRSVAVSQHGGYE-SRVAEDPRLRFDYTPKEFPYPKWDPMPTAVAMHPKVVFHK--DGVRTHCFRFAKDSDD-YEGQENERGVW
    397_g8350.t1                    PSTGL----GHRHDWE----GAVVWLSSAAADAT--VVGVAASAHGSYD-VKNAADVSFT--------------------GVRPRLGYRS--TWPVNHQMIFTSDQGG------------
    397_g9733.t1                    AT----PIDGHRHDWE----HIAVWVRQSDNF----VSHVAVSQHKGYD-IRENSQIQWT-----------------SQGDGKPAVVYHK--DSAFTHCFRFADGADFGNGGPENHWRTW
    CBS382.66_g255.t1               AWSWPVSGYNHRHDWE----HVVVWAKEGK------VRGVSVSQHGGYE-NRVAEDQRLRFDYTPKEFPYPAWDPMPTSVAMHPKVVFHK--DGARTHCFRFAKDSDD-YEGQENERGVW
    CBS382.66_g255.t2               AWSWPVSGYNHRHDWE----HVVVWAKEGK------VRGVSVSQHGGYE-NRVAEDQRLRFDYTPKEFPYPAWDPMPTSVAMHPKVVFHK--DGARTHCFRFAKDSDD-YEGQENERGVW
    CBS382.66_g4327.t1              PSSGL----GHRHDWE----GAVVWLSSAAADAT--VVGVAASAHGDYD-VQRAADVSFA--------------------GARPKLGYRS--TWPVNHQMVFTADQGG------------
    CBS382.66_g5334.t1              A-----DIGAHKHDWE----HIIVWVPDDRANN---KKYACVSQHGEWL-CNPEDKVLWK--------------------DEHPKIVYHK--DWFQTRAFRFANAEDD--KYQENERHDW
    CBS382.66_g5334.t2              A-----DIGAHKHDWE----HIIVWVPDDRANN---KKYACVSQHGEWL-CNPEDKVLWK--------------------DEHPKIVYHK--DWFQTRAFRFANAEDD--KYQENERHDW
    CBS382.66_g5795.t1              PIAGN-VAGGHRHDWE----NIVVFVDDPAANPAPGILGGAASGHGEYK-KTATPDRE----------------------GDSVKVEYFT--TFPTNHELQFTATTGK------------
    CBS382.66_g6578.t1              SVSGS-FAGGHRHDWE----NVVVFARGDT------IVRVAPSCHGGYG-GASN------------EF---------PADGTSPQMVYHK--DSAGTHCFRFANDAD--IGGVENFSGSF
    CBS382.66_g8384.t1              ------GWGAHRHDWE----HIAVWVQHGQ------LKFVSISQHGKWD-IRILDGRTAAPR---------------FEHGTHPKVVYHK--DGALTHAFRWANGGD---EPPENHWKSW
    CBS382.66_g9605.t1              PADGN-LVSGHRHDWE----NVVIWFNSNNANQA-GILRGAASGHGDYK-KVNNPQRN----------------------GNNLHVEYFT--SLGKNHELQFKTSPGR------------
    CBS382.66_g9740.t1              AT----PIDGHRHDWE----HIAVWVRQSDSF----VTHVAVSQHKGYD-IRENSQITWT-----------------AAENGKPAIVYHK--DSILTHCFRFGNGADAGGPGPENHRNQW
    MUCL9792_g2463.t1               PADGN-LVSGHRHDWE----SSVVWLNSQSVN-A-GIVAGAASGHGDFK-RTANPQRR----------------------GNNVKVEYFT--SGGKNHELQFTNTEGR------------
    MUCL9792_g2802.t1               GTGGL-GIVGHRHDWE----CVVVWVRNPAVANP-DVIGISTSAHGKFT-KEPGAIRDNM------------------PGTRHPKIKYYQDVAFFGTHSVHTTGNEGR------------
    MUCL9792_g3157.t1               T--GI-GLGGHRHDWE----HIAVWVSDGQ------LKFISISKHGKWE-IRENPGG-----------------------GSHVKVVYHQ--DGALTHAFRYAKDKNG-DEPPENHWQSW
    MUCL9792_g4589.t1               A-----DIGGHKNDWE----HIIVWVPDDKTRD---KKYVCASQHGGWL-CNPQEDVLWK--------------------DEHPKMVYHK--DWFQTRTFRFANSKDD--AYQENARHEW
    MUCL9792_g4589.t2               A-----DIGGHKNDWE----HIIVWVPDDKTRD---KKYVCASQHGGWL-CNPQEDVLWK--------------------DEHPKMVYHK--DWFQTRTFRFANSKDD--AYQENARHEW
    MUCL9792_g4589.t3               ------------------------------------------------------------------------------------------------------------------------
    MUCL9792_g6332.t1               SVSGS-FAGGHRHDWE----NVVVFARGDT------IVRVAPSCHGGYG-PASN------------EF---------PADGTSPQIVYHK--DGAGTHCFRFANDGD--IGGVENFSGSF
    MUCL9792_g7308.t1               PSSGL----GHRHDWE----AAVVWLSSSAADAT--VLGVAASAHGGFD-PKPAADVSFT--------------------GVRPRLGYRS--TWPVNHQIVFTSDQGG------------
    MUCL9792_g10611.t1              PIAGN-VAGGHRHDFE----NIVIFVDDPATNPTPAILGGAASGHGDYK-KTATPDVE----------------------GDSVKVEYFT--TFPTNHELQFTATSGK------------
    PD401_g2361.t1                  GTGGL-GITGHRHDWE----CVVVWVKNPAVVNP-EVIGISTSAHGKFT-KEPGAIKDNM------------------PGTRHPKIKYYQDVAFFGTHSVHTTGNEGR------------
    PD401_g3373.t1                  PSSGL----GHRHDWE----AAVVWLSSSAADAT--VLGVAASAHGGFD-PKPAADVSFT--------------------GVRPRLGYRS--TWPVNHQIVFTSDQGG------------
    PD401_g4730.t1                  SVSGS-FAGGHRHDWE----NVVVFARGDT------IVRVAPSCHGGYG-PASN------------EF---------PADGTSPQIVYHK--DGAGTHCFRFANDAD--IGGVENFSGSF
    PD401_g7339.t1                  PIAGN-VAGGHRHDFE----NIVIFVDDPAANPTPAILGGAASGHGDYK-KTATPDVE----------------------GDSVKVEYFT--TFPTNHELQFTATSGK------------
    PD401_g10483.t1                 PADGN-LVSGHRHDWE----SSVVWLNSQSVN-A-GIVAGAASGHGDFK-RTANPQRR----------------------GNNVKVEYFT--SGGKNHELQFTNTEGR------------
    PD593_g749.t1                   A-----DIGGHKNDWE----HIIVWVPDDKTRD---KKYVCASQHGGWL-CNPQEDVLWK--------------------DEHPKMVYHK--DWFQTRTFRFANSKDD--AYQENARHEW
    PD593_g2541.t1                  GTGGL-GIVGHRHDWE----CVVVWVRNPAVANP-DVIGISTSAHGKFT-KEPGAIRDNM------------------PGTRHPKIKYYQDVAFFGTHSVHTTGNEGR------------
    PD593_g2728.t1                  SVSGS-FAGGHRHDWE----NVVVFARGDT------IVRVAPSCHGGYG-PASN------------EF---------PADGTSPQIVYHK--DGAGTHCFRFANDGD--IGGVENFSGSF
    PD593_g7030.t1                  PSSGL----GHRHDWE----AAVVWLSSSAADAT--VLGVAASAHGGFD-PKPAADVSFT--------------------GVRPRLGYRS--TWPVNHQIVFTSDQGG------------
    PD593_g8432.t1                  PIAGN-VAGGHRHDFE----NIVIFVDDPATNPTPAILGGAASGHGDYK-KTATPDVE----------------------GDSVKVEYFT--TFPTNHELQFTATSGK------------
    PD593_g9596.t1                  PADGN-LVSGHRHDWE----SSVVWLNSQSVN-A-GIVAGAASGHGDFK-RTANPQRR----------------------GNNVKVEYFT--SGGKNHELQFTNTEGR------------
    PD618_g114.t1                   GTGGL-GIVGHRHDWE----CVVVWVKNPAVVNP-EVIGISTSAHGKFT-KEPGAIRDNM------------------PGTRHPKIKYYQDVAFFGTHSVHTTGNEGR------------
    PD618_g3974.t1                  SVSGS-FAGGHRHDWE----NVVVFARGDT------IVRVAPSCHGGYG-PASN------------EF---------PADGTSPQIVYHK--DGAGTHCFRFANDAD--IGGVENFSGSF
    PD618_g5755.t1                  PSSGL----GHRHDWE----AAVVWLSSSAADAT--VLGVAASAHGGFD-PKPAADVSFT--------------------GVRPRLGYRS--TWPVNHQIVFTSDQGG------------
    PD618_g9213.t1                  PIAGN-VAGGHRHDFE----NIVIFVDDPAANPTPAILGGAASGHGDYK-KTATPDVE----------------------GDSVKVEYFT--TFPTNHELQFTATSGK------------
    PD618_g9942.t1                  PADGN-LVSGHRHDWE----SSVVWLNSQSVN-A-GIVAGAASGHGDFK-RTANPQRR----------------------GNNVKVEYFT--SGGKNHELQFTNTEGR------------
    PD659_g6753.t1                  GTGGL-GITGHRHDWE----CVVVWVKNPAVVNP-EVIGISTSAHGKFT-KEPGAIKDNM------------------PGTRHPKIKYYQDVAFFGTHSVHTTGNEGR------------
    PD659_g7370.t1                  PIAGN-VAGGHRHDFE----NIVIFVDDPAANPTPAILGGAASGHGDYK-KTATPDVE----------------------GDSVKVEYFT--TFPTNHELQFTATSGK------------
    PD659_g10785.t1                 PADGN-LVSGHRHDWE----SSVVWLNSQSVN-A-GIVAGAASGHGDFK-RTANPQRR----------------------GNNVKVEYFT--SGGKNHELQFTNTEGR------------
    PD659_g2262.t1                  SVSGS-FAGGHRHDWE----NVVVFARGDT------IVRVAPSCHGGYG-PASN------------EF---------PADGTSPQIVYHK--DGAGTHCFRFANDAD--IGGVENFSGSF
    PD659_g5819.t1                  PSSGL----GHRHDWE----AAVVWLSSSAADAT--VLGVAASAHGGFD-PKPAADVSFT--------------------GVRPRLGYRS--TWPVNHQIVFTSDQGG------------
    PD660_g1229.t1                  PSSGL----GHRHDWE----AAVVWLSSSAADAT--VLGVAASAHGGFD-PKPAADVSFT--------------------GVRPRLGYRS--TWPVNHQIVFTSDQGG------------
    PD660_g3973.t1                  SVSGS-FAGGHRHDWE----NVVVFARGDT------IVRVAPSCHGGYG-PASN------------EF---------PADGTSPQIVYHK--DGAGTHCFRFANDAD--IGGVENFSGSF
    PD660_g6305.t1                  PADGN-LVSGHRHDWE----SSVVWLNSQSVN-A-GIVAGAASGHGDFK-RTANPQRR----------------------GNNVKVEYFT--SGGKNHELQFTNTEGR------------
    PD660_g7826.t1                  PIAGN-VAGGHRHDFE----NIVVFVDDPAANPTPAILGGAASGHGDYK-KTATPDVE----------------------GDSVKVEYFT--TFPTNHELQFTATSGK------------
    PD660_g9669.t1                  GTGGL-GIVGHRHDWE----CVVVWVKNPAVVNP-EVIGISTSAHGKFT-KEPGPIRDNM------------------PGTRHPKIKYYQDVAFFGTHSVHTTGNEGR------------
    PD660_g10032.t1                 A-----GLGGHRHDWE----HIAVWVSDGQ------LKFISISKHGKWE-IRENPGG-----------------------GSHVKVVYHQ--DGALTHAFRYAKNGNG-DEPPENHWQSW
    PD670_g4670.t1                  AALGT-FAGGHRHDWE----NVVVFARGDT------IVRVAPSCHGGYG-PASN------------EF---------PADGTSPQIVYHK--DGAGTHCFRFANDAD--IGGVENFSGSF
    PD670_g5219.t1                  PSSGL----GHRHDWE----AAVVWLSSAGTDAT--VVSVAASAHGDFD-TKVAADVSFT--------------------GVRPRLGYSS--TWPVNHQIIFTSDQGG------------
    PD670_g7258.t1                  PIAGN-VAGGHRHDWE----NIVVFVDDPAVNPTPAILGGAASGHGEYK-KTATPDVE----------------------GDSVKVEYFT--TFPTNHELQFTATSGK------------
    PD670_g8696.t1                  AT----PIDGHRHDWE----HIAVWVRQSDNY----VTHVAVSQHKGYE-IRENQNIQWT-----------------SQGDGKPAIVYHK--DGPSTHCFRFANGADFGNGGPENHWRQW
    PD670_g10480.t1                 NVDGP-GNRGHRHDWE----HVVVWLSGKSTSAK--VLGVSYSGHGKYT-KESKRGGGNRF-----------------ANTSHVKVGYMN--YGSLNHSLRPDSGNGG------------
    PD670_g10529.t1                 PADGN-LVSGHRHDWE----SSVVWLNGQNVN-A-GIVAGAASGHGDFK-RTANPQRR----------------------GNNVKVEYFT--SGGKNHELQFTGTEGR------------
    PD670_g11082.t1                 GTGSL-AFTGHKHDWE----CVVVWVRNPAVANP-VVLGLSTSAHGGFI-KEPGAVKDNM------------------PGTRHPKIKYHQDFAFFGTHSVHTTGHDGR------------
    PD670_g2580.t1                  ------GLGGHRHDWE----HVAVWVQNGQ------LKFVAISMHGKWD-IRFADSKDAAPR---------------FEYGTHPKVVYHK--DGGFTHAFRWANGGD---EPPENHWQSW
    PD670_g3093.t1                  A-----DIGGHKNDWE----HIIVWVPDDKTRN---KKYVCASQHGGWL-CNPQEDVLWK--------------------DEHPKMVYHK--DWFQTRTFRFANSKDD--AYQENARHEW
    PD683_g248.t1                   AWSWPVSGYNHRHDWE----HVVVWAKEGK------VRGVSVSQHGGYE-NRVAEDQRLRFDYTPKEFPYPAWDPMPTSVAMHPKVVFHK--DGARTHCFRFAKDSDD-YEGQENERGVW
    PD683_g3766.t1                  AT----PIDGHRHDWE----HIAVWVRQSDSF----VTHVAVSQHKGYE-IRENSQVTWT-----------------AAENGKPAIVYHK--DSILTHCFRFGNGADAGGPGPENHRNQW
    PD683_g3895.t1                  PADGN-LVSGHRHDWE----NVVIWFNSNNANQA-GILRGAASGHGNYK-KVNNPQRN----------------------GNNLHVEYFT--SLGKNHELQFKTSPGR------------
    PD683_g4100.t1                  PSSGL----GHRHDWE----GAVVWLSSAAADAT--VVGVAASAHGDYD-VKRAADVSFA--------------------GARPKLGYRS--TWPVNHQMVFTADQGG------------
    PD683_g5003.t1                  A-----DIGAHKHDWE----HIIVWVPDDRANN---KKYACVSQHGEWL-CNPEDKVLWK--------------------DEHPKIVYHK--DWFQTRAFRFANAEDD--KYQENERHDW
    PD683_g5003.t2                  A-----DIGAHKHDWE----HIIVWVPDDRANN---KKYACVSQHGEWL-CNPEDKVLWK--------------------DEHPKIVYHK--DWFQTRAFRFANAEDD--KYQENERHDW
    PD683_g5713.t1                  PIAGN-VAGGHRHDWE----NIVVFVDDPAANPAPGILGGAASGHGEYK-KTATPDRE----------------------GDSVKVEYFT--TFPTNHELQFTATTGK------------
    PD683_g7092.t1                  SVSGS-FAGGHRHDWE----NVVVFARGDT------IVRVAPSCHGGYG-GASN------------EF---------PADGTSPQMVYHK--DSAGTHCFRFANDAD--IGGVENFSGSF
    PD683_g8130.t1                  ------GWGAHRHDWE----HIAVWVQHGQ------LKFVSISQHGKWD-IRILDGRTAAPR---------------FEHGTHPKVVYHK--DGALTHAFRWANGGD---EPPENHWKSW
    PD736_g919.t1                   PSSGL----GHRHDWE----AAVVWLSSAAADAT--VLGVAASAHGGFD-PKPAADVSFT--------------------GVRPRLGYRS--TWPVNHQIVFTSDQGG------------
    PD736_g8670.t1                  PADGN-LVSGHRHDWE----SSVVWLNSQSVN-A-GIVAGAASGHGDFK-RTANPQRR----------------------GNNVKVEYFT--SGGKNHELQFTNTEGR------------
    PD736_g9411.t1                  PIAGN-VAGGHRHDFE----NIVIFVDDPAVNPTPAILGGAASGHGDYK-KTATPDVE----------------------GDSVKVEYFT--TFPTNHELQFTATSGK------------
    PD736_g10271.t1                 SVSGS-FAGGHRHDWE----NVVVFARGDT------IVRVAPSCHGGYG-PASN------------EF---------PADGTSPQIVYHK--DGAGTHCFRFANDAD--IGGVENFSGSF
    PD736_g10840.t1                 A-----DLGGHKNDWE----HIIVWVPDDRTRN---KKYVCASQHGGWL-CNPEEDVLWK--------------------DEHPKIVYHK--DWFQTRAFRFANSDDD--RFQENERHEW
    PD736_g10939.t1                 GTGGL-GVIGHRHDWE----CVVVWVKNPAVVNP-EVIGISTSAHGKFT-KEPGAIKDNM------------------PGTRHPKIKYYQDVAFFGTHSVHTTGNEGR------------
    PD739_g34.t1                    A-----DLGGHKNDWE----HIIVWVPDDRTRN---KKYVCASQHGGWL-CNPEEDVLWK--------------------DEHPKIVYHK--DWFQTRAFRFANSDDD--RYQENERHEW
    PD739_g131.t1                   GTGGL-GVIGHRHDWE----CVVVWVKNPAVVNP-EVIGISTSAHGKFT-KEPGAIKDNM------------------PGTRHPKIKYYQDVAFFGTHSVHTTGNEGR------------
    PD739_g4443.t1                  SVSGS-FAGGHRHDWE----NVVVFARGDT------IVRVAPSCHGGYG-PASN------------EF---------PADGTSPQIVYHK--DGAGTHCFRFANDAD--IGGVENFSGSF
    PD739_g7436.t1                  PSSGL----GHRHDWE----AAVVWLSSAAADAT--VLGVAASAHGGFD-PKPAADVSFT--------------------GVRPRLGYRS--TWPVNHQIVFTSDQGG------------
    PD739_g8512.t1                  PADGN-LVSGHRHDWE----SSVVWLNSQSVN-A-GIVAGAASGHGDFK-RTANPQRR----------------------GNNVKVEYFT--SGGKNHELQFTNTEGR------------
    PD739_g10815.t1                 PIAGN-VAGGHRHDFE----NIVIFVDDPAVNPTPAILGGAASGHGDYK-KTATPDVE----------------------GDSVKVEYFT--TFPTNHELQFTATSGK------------
    PD747_g20.t1                    A-----DIGGHKNDWE----HIIVWVPDDKTRN---KKYVCASQHGGWL-CNPQEDVLWK--------------------DEHPKMVYHK--DWFQTRTFRFANSKDD--AYQENARHEW
    PD747_g277.t1                   GTGSL-AFTGHKHDWE----CVVVWVRNPAVANP-VVLGLSTSAHGGFI-KEPGAVKDNM------------------PGTRHPKIKYHQDFAFFGTHSVHTTGHDGR------------
    PD747_g4174.t1                  AALGT-FAGGHRHDWE----NVVVFARGDT------IVRVAPSCHGGYG-PASN------------EF---------PADGTSPQIVYHK--DGAGTHCFRFANDAD--IGGVENFSGSF
    PD747_g5167.t1                  PSSGL----GHRHDWE----AAVVWLSSAGTDAT--VVSVAASAHGDFD-TKVAADVSFT--------------------GVRPRLGYSS--TWPVNHQIIFTSDQGG------------
    PD747_g9207.t1                  ------GLGGHRHDWE----HVAVWVQNGQ------LKFVAISMHGKWD-IRFADSKDAAPR---------------FEYGTHPKVVYHK--DGGFTHAFRWANGGD---EPPENHWQSW
    PD747_g9751.t1                  PIAGN-VAGGHRHDWE----NIVVFVDDPAVNPTPAILGGAASGHGEYK-KTATPDVE----------------------GDSVKVEYFT--TFPTNHELQFTATSGK------------
    PD747_g10403.t1                 AT----PIDGHRHDWE----HIAVWVRQSDNY----VTHVAVSQHKGYE-IRENQNIQWT-----------------SQGDGKPAIVYHK--DGPSTHCFRFANGADFGNGGPENHWRQW
    PD747_g10498.t1                 NVDGP-GNRGHRHDWE----HVVVWLSGKSTSAK--VLGVSYSGHGKYT-KESKRGGGNRF-----------------ANTSHVKVGYMN--YGSLNHSLRPDSGNGG------------
    PD747_g10549.t1                 PADGN-LVSGHRHDWE----SSVVWLNGQNVN-A-GIVAGAASGHGDFK-RTANPQRR----------------------GNNVKVEYFT--SGGKNHELQFTGTEGR------------
    T2_g2030.t1                     AWSWPVSGYNHRHDWE----HVVVWAKEGK------VRGVSVSQHGGYE-NRVAEDQRLRFDYTPKEFPYPAWDPMPTSVAMHPKVVFHK--DGARTHCFRFAKDSDD-YEGQENERGVW
    T2_g2030.t2                     AWSWPVSGYNHRHDWE----HVVVWAKEGK------VRGVSVSQHGGYE-NRVAEDQRLRFDYTPKEFPYPAWDPMPTSVAMHPKVVFHK--DGARTHCFRFAKDSDD-YEGQENERGVW
    T2_g3751.t1                     A-----DIGAHKHDWE----HIIVWVPDDRANN---KKYACVSQHGEWL-CNPEDKVLWK--------------------DEHPKIVYHK--DWFQTRAFRFANAEDD--KYQENERHDW
    T2_g3751.t2                     A-----DIGAHKHDWE----HIIVWVPDDRANN---KKYACVSQHGEWL-CNPEDKVLWK--------------------DEHPKIVYHK--DWFQTRAFRFANAEDD--KYQENERHDW
    T2_g4661.t1                     PSSGL----GHRHDWE----GAVVWLSSAAADAT--VVGVAASAHGDYD-VQRAADVSFA--------------------GARPKLGYRS--TWPVNHQMVFTADQGG------------
    T2_g4868.t1                     PADGN-LVSGHRHDWE----NVVIWFNSNNANQA-GILRGAASGHGDYK-KVNNPQRN----------------------GNNLHVEYFT--SLGKNHELQFKTSPGR------------
    T2_g5004.t1                     AT----PIDGHRHDWE----HIAVWVRQSDSF----VTHVAVSQHKGYD-IRENSQITWT-----------------AAENGKPAIVYHK--DSILTHCFRFGNGADAGGPGPENHRNQW
    T2_g5801.t1                     PIAGN-VAGGHRHDWE----NIVVFVDDPAANPAPGILGGAASGHGEYK-KTATPDRE----------------------GDSVKVEYFT--TFPTNHELQFTATTGK------------
    T2_g7120.t1                     ------GWGAHRHDWE----HIAVWVQHGQ------LKFVSISQHGKWD-IRILDGRTAAPR---------------FEHGTHPKVVYHK--DGALTHAFRWANGGD---EPPENHWKSW
    T2_g7997.t1                     SVSGS-FAGGHRHDWE----NVVVFARGDT------IVRVAPSCHGGYG-GASN------------EF---------PADGTSPQMVYHK--DSAGTHCFRFANDAD--IGGVENFSGSF
    VSO1_g539.t1                    PSSGL----GHRHDWE----AAVVWLSSSAADAT--VLGVAASAHGGFD-PKPAADVSFT--------------------GVRPRLGYRS--TWPVNHQIVFTSDQGG------------
    VSO1_g1387.t1                   GTGGL-GIVGHRHDWE----CVVVWVKNPAVVNP-EVIGISTSAHGKFT-KEPGPIRDNM------------------PGTRHPKIKYYQDVAFFGTHSVHTTGNEGR------------
    VSO1_g1731.t1                   PADGN-LVSGHRHDWE----SSVVWLNSQSVN-A-GIVAGAASGHGDFK-RTANPQRR----------------------GNNVKVEYFT--SGGKNHELQFTNTEGR------------
    VSO1_g1937.t1                   A-----GLGGHRHDWE----HIAVWVSDGQ------LKFISISKHGKWE-IREYPGG-----------------------GSHVKVVYHQ--DGALTHAFRYAKNGNG-DEPPENHWQSW
    VSO1_g4972.t1                   SVSGS-FAGGHRHDWE----NVVVFARGDT------IVRVAPSCHGGYG-PASN------------EF---------PADGTSPQIVYHK--DGAGTHCFRFANDAD--IGGVENFSGSF
    VSO1_g5184.t1                   PIAGN-VAGGHRHDFE----NIVIFVDDPAANPTPAILGGAASGHGDYK-KTATPDVE----------------------GDSVKVEYFT--TFPTNHELQFTATSGK------------
    VL20_g7800.t1                   AWSWPVSGYNHRHDWE----HVVVWAKEGK------VRGVSVSHHGGYK-SRVAEDQRLRFDYTPKEFPYPAWDPMPTSVAMHPKVVFHK--DGARTHCFRFAKDSDD-YEGQENERGIW
    VL20_g9415.t1                   SVSGS-FAGGHRHDWE----NVVVFARGDT------IVRVAPSCHGGYG-GALN------------EF---------PVDGTSPQMVYHK--DSAGTHCFRFANDAD--IGGVENFSGSF
    VL20_g11025.t1                  PADGN-LVSGHRHDWE----NVVIWFNSNNANQA-GILRGAASGHGNYK-KVNNPQRS----------------------GNNLNVEYFT--SGGKNHELQFKTSPGR------------
    VL20_g11172.t1                  AWSWPVSSYNHRHDWE----HVVVWAKEGK------VRGVSVSQHGGYE-NRVAEDQRLRFDYTPKEFPYPAWDPMPTSVAMHPKVVFHK--DGARTHCFRFAKDSDD-YEGQENERGVW
    VL20_g12965.t1                  ------GWGAHRQDWE----HIAVWVQHGQ------LKFVSISQHGKWD-IRILDGRTAAPR---------------FERGTHPKVVYHK--DGALTHAFRWANDGD---EPPENHWKSW
    VL20_g13837.t1                  PSSGL----GHRHDWE----GAVVWLSSAAADAT--VVGVAASAHGDFD-VRPAADVSFA--------------------GARPKLGYRS--TWPVNHQMVFTADQGG------------
    VL20_g14042.t1                  PADGN-LASGHRHDWE----NVVIWFNSNNANQA-GILRGAASGHGDYK-KVNNPQRN----------------------NNNLHVEYFT--SLGKNHELQFKTSPGR------------
    VL20_g14171.t1                  AT----PIDGHRHDWE----HIAVWVRQSDSF----VTHVAVSQHKGYD-IRENSQITWT-----------------AAENGKPAIVYHK--DSILTHCFRFGSGADAGGPGPENHKNQW
    VL20_g14284.t1                  SVSSS-FAGGHRHDWE----NVVVFARGDT------IVRVAPSCHGGYG-GASN------------EF---------PADGTSPQMVYHK--DSAGTHCFRFANDAD--IGGVENFSGSF
    VL20_g17450.t1                  PIAGN-VAGGHRHDWE----NIVVFVDDPAANPAPGILGGAASGHGEYK-KTATPDRE----------------------GDSVKVEYFT--TFPTNHELQFTATTGK------------
    VL20_g18857.t1                  PIAGN-VAGGHRHDWE----TVVVFVDDPAANAAPGLLGGAASGHGEYK-KTATPDRE----------------------GDSVKVEYFT--TFPTNHELQFTATTGK------------
    VL20_g4752.t1                   PSSGL----GHRHDWE----GAVVWLSSAAADAT--VVGVAASAHGDYD-VKGAADVSFA--------------------GARPKLGYRS--TWPVNHQMVFTSDQGG------------
    VL20_g5938.t1                   ------GWGAHRHDWE----HVAVWVQHGQ------LKFVSISQHGKWD-IRILDGRTEAPR---------------FEHGTHPKVVYHK--DGALTHAFRWANGGD---EPPENHWKSW
    VL20_g5938.t2                   ------GWGAHRHDWE----HVAVWVQHGQ------LKFVSISQHGKWD-IRILDGRTEAPR---------------FEHGTHPKVVYHK--DGALTHAFRWANGGD---EPPENHWKSW
    VL20_g6061.t1                   AT----PIDGHRHDWE----HIAVWVRQSDSF----VTHVAVSQHKGYD-IRENSQVTWT-----------------AAENGKPAIVYHK--DSILTHCFRFGNAADAGGPGPENHRNQW
    VL20_g18964.t1                  ------GWGAHRHDWE----HVAVWVQHGQ------LKFVSISQHGKWD-IRILDGRTEAPR---------------FEHGTHPKVVYHK--DGALTHAFRWANGGD---EPPENHWKSW
    VL20_g18964.t2                  ------GWGAHRHDWE----HVAVWVQHGQ------LKFVSISQHGKWD-IRILDGRTEAPR---------------FEHGTHPKVVYHK--DGALTHAFRWANGGD---EPPENHWKSW
    VLB2_g585.t1                    AWSWPVSGYNHRHDWE----HVVVWAKEGK------VRGVSVSHHGGYK-SRVAEDQRLRFDYTPKEFPYPAWDPMPTSVAMHPKVVFHK--DGARTHCFRFAKDSDD-YEGQENERGIW
    VLB2_g1761.t1                   PADGN-LVSGHRHDWE----NVVIWFNSNNANQA-GILRGAASGHGNYK-KVNNPQRS----------------------GNNLNVEYFT--SGGKNHELQFKTSPGR------------
    VLB2_g2033.t1                   AWSWPVSSYNHRHDWE----HVVVWAKEGK------VRGVSVSQHGGYE-NRVAEDQRLRFDYTPKEFPYPAWDPMPTSVAMHPKVVFHK--DGARTHCFRFAKDSDD-YEGQENERGVW
    VLB2_g12050.t1                  PIAGN-VAGGHRHDWE----NIVVFVDDPAANPAPGILGGAASGHGEYK-KTATPDRE----------------------GDSVKVEYFT--TFPTNHELQFTATTGK------------
    VLB2_g12617.t1                  ------GWGAHRQDWE----HIAVWVQHGQ------LKFVSISQHGKWD-IRILDGRTAAPR---------------FERGTHPKVVYHK--DGALTHAFRWANDGD---EPPENHWKSW
    VLB2_g13499.t1                  PSSGL----GHRHDWE----GAVVWLSSAAADAT--VVGVAASAHGDFD-VRPAADVSFA--------------------GARPKLGYRS--TWPVNHQMVFTADQGG------------
    VLB2_g13703.t1                  PADGN-LASGHRHDWE----NVVIWFNSNNANQA-GILRGAASGHGDYK-KVNNPQRN----------------------NNNLHVEYFT--SLGKNHELQFKTSPGR------------
    VLB2_g13833.t1                  AT----PIDGHRHDWE----HIAVWVRQSDSF----VTHVAVSQHKGYD-IRENSQITWT-----------------AAENGKPAIVYHK--DSILTHCFRFGSGADAGGPGPENHKNQW
    VLB2_g14318.t1                  SVSSS-FAGGHRHDWE----NVVVFARGDT------IVRVAPSCHGGYG-GASN------------EF---------PADGTSPQMVYHK--DSAGTHCFRFANDAD--IGGVENFSGSF
    VLB2_g15187.t1                  ------GWGAHRHDWE----HVAVWVQHGQ------LKFVSISQHGKWD-IRILDGRTEAPR---------------FEHGTHPKVVYHK--DGALTHAFRWANGGD---EPPENHWKSW
    VLB2_g15187.t2                  ------GWGAHRHDWE----HVAVWVQHGQ------LKFVSISQHGKWD-IRILDGRTEAPR---------------FEHGTHPKVVYHK--DGALTHAFRWANGGD---EPPENHWKSW
    VLB2_g16101.t1                  PSSGL----GHRHDWE----GAVVWLSSAAADAT--VVGVAASAHGDYD-VKGAADVSFA--------------------GARPKLGYRS--TWPVNHQMVFTSDQGG------------
    VLB2_g16543.t1                  SVSGS-FAGGHRHDWE----NVVVFARGDT------IVRVAPSCHGGYG-GALN------------EF---------PVDGTSPQMVYHK--DSAGTHCFRFANDAD--IGGVENFSGSF
    VLB2_g19098.t1                  A-----GIGAHKHDWE----HIIVWVPDDRTNN---KKYACVSQHGEWL-CNPEDKVLWK--------------------DEHPKIVYHK--DWFQTRAFRFANAEDD--KYQENERHDW
    VLB2_g5814.t1                   PIAGN-VAGGHRHDWE----TVVVFVDDPAANAAPGLLGGAASGHGEYK-KTATPDRE----------------------GDSVKVEYFT--TFPTNHELQFTATTGK------------
    VLB2_g6534.t1                   AT----PIDGHRHDWE----HIAVWVRQSDSF----VTHVAVSQHKGYD-IRENSQVTWT-----------------AAENGKPAIVYHK--DSILTHCFRFGNAADAGGPGPENHRNQW
    PD589_g51.t1                    ------GWGAHRHDWE----HIAVWVQHGQ------LKFVSISQHGKWD-IRILDGRTAAPR---------------FEHGTHPKVVYHK--DGALTHAFRWANDGD---EPPENHWKSW
    PD589_g856.t1                   PPRGN-GLFAARRPWSEPEGNVNVPQTRLNSN----ILRGAASGHGSYK-KVNNPQRS----------------------GNNVNAEYFT--SGGKNHELQFKTSPGR------------
    PD589_g7665.t1                  SVSGS-FAGGHRHDWE----NVVVFARGDT------IVRVAPSCHGGYG-GASN------------EF---------PADGTSPQMVYHK--DSAGTHGFRFANDAD--IGGVENFSGSF
    PD589_g13197.t1                 AWSWPVSGYNHRHDWE----HVVVWAKEGK------VRGVSVSQHGGYE-SRVAEDQRLRFDYTPKEFPYPAWDPMPTSVAMHPKVVFHK--DGARTHCFRFAKDSDD-YEGQENERGIW
    PD589_g13425.t1                 PIAGN-VAGGHRHDWE----NIVVFVDDPAANPAPGILGGAASGHGEYK-KTATPDRE----------------------GDSVKVEYFT--TFPTNHELQFTATTGK------------
    PD589_g14403.t1                 AT----PIDGHRHDWE----HIAVWVRQSDSF----VTHVAVSQHKGYD-IRENSQVTWT-----------------AAENGKPAIVYHK--DSILTHCFRFGNAADAGGPGPENHRNQW
    PD589_g14539.t1                 AT----PIDGHRHDWE----HIAVWVRQSDSF----VTHVAVSQHKGYD-IRENSQITWT-----------------AAENGKPAIVYHK--DSILTHCFRFGSGADAGGPGPENHKNQW
    PD589_g14661.t1                 PADGN-LASGHRHDWE----NVVIWFNSNNANQA-GILRGAASGHGDYK-KVNNPQRN----------------------NNNLHVEYFT--SLGKNHELQFKTSPGR------------
    PD589_g15310.t1                 PIAGN-VAGGHRHDWE----NVVIFVDDPAANAAPGLLGGAASGHGEYK-KTATPDRE----------------------GDSGKVEYFT--TYPSNPELQYTVSTGK------------
    PD589_g16171.t1                 ------GWGAHRHDWE----HVAVWVQHGQ------LKFVSISQHGKWD-IRILDGRTEAPR---------------FEHGTHPKVVYHK--DGALTHAFRWANGGD---EPPENHWKSW
    PD589_g16171.t2                 ------GWGAHRHDWE----HVAVWVQHGQ------LKFVSISQHGKWD-IRILDGRTEAPR---------------FEHGTHPKVVYHK--DGALTHAFRWANGGD---EPPENHWKSW
    PD589_g17659.t1                 SVSSS-FAGGHRHDWE----NVVVFARGDT------IVRVAPSCHGGYG-GASN------------EF---------PADGTSPQMVYHK--DSAGTHCFRFANDAD--IGGVENFSGSF
    PD589_g18192.t1                 PSSGL----GHRHDWE----GAVVWLSSAAADAT--VVGVAASAHGDFD-VRPAADVSFA--------------------GARPKLGYRS--TWPVNHQMVFTADQGG------------
    PD589_g3050.t1                  PADGN-LVSGHRHDWE----NVVIWFNSNNANQA-GILRGAASGHGNYK-KVNNPQRS----------------------GNNLNVEYFT--SGGKNHELQFKTSPGR------------
    PD589_g6680.t1                  AWSWPVSSYNHRHDWE----HVVVWAKEGK------VRGVSVSQHGGYE-NRVAEDQRLRFDYTPKEFPYPAWDPMPTSVAMHPKVVFHK--DGARTHCFRFAKDSDD-YEGQENERGVW
    PD589_g6775.t1                  PSSGL----GPRHDWE----GAVVWLSSAAADAT--VVGVAASAHGDYD-VKGAADVSFA--------------------GARPKLGYRS--TWPVNHQMVFTSDQGG------------
    Vt305_g1664.t1                  PADGN-LVSGHRHDWE----SSVVWLNSQSVN-A-GIVAGAASGHGDFK-RTANPQRR----------------------GNNVKVEYFT--SGGKNHELQFTNTEGR------------
    Vt305_g2624.t1                  SVSGS-FAGGHRHDWE----NVVVFARGDT------IVRVAPSCHGGYG-PASN------------EF---------PADGTSPQIVYHK--DGAGTHCFRFANDAD--IGGVENFSGSF
    Vt305_g3295.t1                  PIAGN-VAGGHRHDFE----NIVIFVDDPAANPTPAILGGAASGHGDYK-KTATPDVE----------------------GDSVKVEYFT--TFPTNHELQFTATSGK------------
    Vt305_g4616.t1                  GTGGL-GIVGHRHDWE----CVVVWVKNPAVVNP-EVIGISTSAHGKFT-KEPGAIRDNM------------------PGTRHPKIKYYQDVAFFGTHSVHTTGNEGR------------
    Vt305_g7985.t1                  PSSGL----GHRHDWE----AAVVWLSSSAADAT--VLGVAASAHGGFD-PKPAADVSFT--------------------GVRPRLGYRS--TWPVNHQIVFTNDQGG------------
    WCS072_g2037.t1                 AWSWPVSGYNHRHDWE----HVVVWAKEGK------VRGVSVSQHGGYE-NRVAEDQRLRFDYTPKEFPYPAWDPMPTSVAMHPKVVFHK--DGARTHCFRFAKDSDD-YEGQENERGVW
    WCS072_g2037.t2                 AWSWPVSGYNHRHDWE----HVVVWAKEGK------VRGVSVSQHGGYE-NRVAEDQRLRFDYTPKEFPYPAWDPMPTSVAMHPKVVFHK--DGARTHCFRFAKDSDD-YEGQENERGVW
    WCS072_g3872.t1                 AT----PIDGHRHDWE----HIAVWVRQSDSF----VTHVAVSQHKGYD-IRENSQITWT-----------------AAENGKPAIVYHK--DSILTHCFRFGNGADAGGPGPENHRNQW
    WCS072_g4007.t1                 PADGN-LVSGHRHDWE----NVVIWFNSNNANQA-GILRGAASGHGDYK-KVNNPQRN----------------------GNNLHVEYFT--SLGKNHELQFKTSPGR------------
    WCS072_g4219.t1                 PSSGL----GHRHDWE----GAVVWLSSAAADAT--VVGVAASAHGDYD-VQRAADVSFA--------------------GARPKLGYRS--TWPVNHQMVFTADQGG------------
    WCS072_g5132.t1                 A-----DIGAHKHDWE----HIIVWVPDDRANN---KKYACVSQHGEWL-CNPEDKVLWK--------------------DEHPKIVYHK--DWFQTRAFRFANAEDD--KYQENERHDW
    WCS072_g5132.t2                 A-----DIGAHKHDWE----HIIVWVPDDRANN---KKYACVSQHGEWL-CNPEDKVLWK--------------------DEHPKIVYHK--DWFQTRAFRFANAEDD--KYQENERHDW
    WCS072_g5615.t1                 PIAGN-VAGGHRHDWE----NIVVFVDDPAANPAPGILGGAASGHGEYK-KTATPDRE----------------------GDSVKVEYFT--TFPTNHELQFTATTGK------------
    WCS072_g6396.t1                 SVSGS-FAGGHRHDWE----NVVVFARGDT------IVRVAPSCHGGYG-GASN------------EF---------PADGTSPQMVYHK--DSAGTHCFRFANDAD--IGGVENFSGSF
    WCS072_g8181.t1                 ------GWGAHRHDWE----HIAVWVQHGQ------LKFVSISQHGKWD-IRILDGRTAAPR---------------FEHGTHPKVVYHK--DGALTHAFRWANGGD---EPPENHWKSW
    VDAG_JR2_Chr1g22850a-00001      AWSWPVSGYNHRHDWE----HVVVWAKEGK------VRGVSVSQHGGYE-SRVAEDQRLRFDYTPKEFPYPAWDPMPTSVAMHPKVVFHK--DGARTHCFRFAKDSDD-YEGQENERGIW
    VDAG_JR2_Chr2g05460a-00001      PSSGL----GHRHDWE----GAVVWLSSAAADAT--VVGVAASAHGDFD-VRRAADVSFA--------------------GARPKLGYRS--TWPVNHQMVFTADQGG------------
    VDAG_JR2_Chr6g08770a-00001      PADGN-LASGHRHDWE----NVVIWFNSNNANQA-GILRGAASGHGDYK-KVNNPQRN----------------------NNNLHVEYFT--SLGKNHELQFKTSPGR------------
    VDAG_JR2_Chr6g10260a-00001      AT----PIDGHRHDWE----HIAVWVRQSDSF----VTHVAVSQHKGYD-IRENSQITWT-----------------AAENGKPAIVYHK--DSILTHCFRFGSGADAGGPGPENHKNQW
    VDAG_JR2_Chr3g00800a-00001      SVSGS-FAGGHRHDWE----NVVVFARGDT------IVRVAPSCHGGYD-GASN------------EF---------PADGTSPQMVYHK--DSAGTHCFRFANDAD--IGGVENFSGSF
    VDAG_JR2_Chr4g05950a-00001      PIAGN-VAGGHRHDWE----NVVVFVDDPAANAAPGLLGGAASGHGEYK-KTATPDRE----------------------GDSVKVEYFT--TFPTNHELQFTATTGK------------
    VDAG_JR2_Chr8g10650a-00001      ------GWGAHRHDWE----HIAVWVQHGQ------LKFVSISQHGKWD-IRILDGRTAAPR---------------FEHGTHPKVVYHK--DGALTHAFRWANDGD---EPPENHWKSW
    JR2_g2720.t1                    A-----DIGAHKHDWE----HIIVWVTDDRTKN---KKYACVSQHGEWL-CHPEDKVLWK--------------------DEHPKIVYHK--DWFQTRAFRFANAEDD--KYQENERHDW

    Selected Cols:                                                                                                                                          

    Gaps Scores:                                                                                                                                            
    Similarity Scores:                                                                                                                                      

                                           370       380       390       400       410       420       430       440       450       460       470       480
                                    =========+=========+=========+=========+=========+=========+=========+=========+=========+=========+=========+=========+
    12008_g358.t1                   ---T-YPISDW----------DAMPQ-AARDALETTDFGSANVPFKDA-----NFDSNLAKAAL--------------------------------------------------------
    12008_g4461.t1                  YKS---PLVGW----------LSWPNEGLRQTMLG-AFSGGVGPKLDD-----EFAGKLGEAAGDAVPE--------------------FDPNVDE------------------------
    12008_g7340.t1                  RWGVGAGLIEW----------ERMPD-NLRKTLSAKNWGAAEMAVRDKDGSDWNFAWYINESQY------F----------------C-W-----ETYCPG-------------------
    12008_g7652.t1                  ---E-QPLVAW----------ESLTP-AARAALQNTDFGSANVPLKDG-----NFASNLQKAAL--------------------------------------------------------
    12008_g7834.t1                  ITG---PLLGY----------FGWDTVEQRDRMLTHNWEAGSIAIKNE-----NFAENIRKARPAGL---------------------VFDENFDDEGT---------------------
    12008_g8465.t1                  IRG---GLVSM----------LLMPS-DWQDKFRSHGWGSAHMAWANED----DFTGHLVKSMPQEARDD------------------GFDCAYDENPA---------------------
    12008_g8848.t1                  ---T-YWIWDW----------DRMDT-TVQGALNRADFGSANCPFNNN-----NFERNMRAAF---------------------------------------------------------
    85S_g58.t1                      RWGVGAGLIEW----------ERMPD-NLRKTLSAKNWGAAEMAVRDKDGSDWNFAWYINESQY------F----------------C-W-----ETYCPG-------------------
    85S_g9168.t1                    ---E-QPLVAW----------ESLTP-AARAALQNTDFGSANVPLKDG-----NFASNLQKAAL--------------------------------------------------------
    85S_g9863.t1                    YKS---PLVGW----------LSWPNEGLRQTMLG-AFSGGVGPKLDD-----EFAGKLGEAAGDAVPE--------------------FDPNVDE------------------------
    85S_g7040.t1                    IRG---GLVSM----------LLMPS-DWQDKFRSHGWGSAHMAWANED----DFTGHLVKSMPQEARDD------------------GFDCAYDENPA---------------------
    85S_g3154.t1                    ---T-YPISDW----------DAMPQ-AARDALETTDFGSANVPFKDA-----NFDSNLAKAAL--------------------------------------------------------
    85S_g4608.t1                    ITG---PLLGY----------FGWDTVEQRDRMLTHNWEAGSIATKNE-----NFAENIRKARPAGL---------------------VFDENFDDEGT---------------------
    85S_g4744.t1                    ---T-YWIWDW----------DRMDT-TVQGALNRADFGSANCPFNNN-----NFERNMRAAF---------------------------------------------------------
    CBS385.49_g66.t1                ITG---PLLGY----------FGWDTVEQRDRMLTHNWEAGSIAIKNE-----NFAENIRKARPAGL---------------------VFDENFDDEGT---------------------
    CBS385.49_g189.t1               ---T-YWIWDW----------DRMDT-TVQGALNRADFGSANCPFNNN-----NFERNMRAAF---------------------------------------------------------
    CBS385.49_g400.t1               ---E-QPLVAW----------ESLTP-AARAALQNTDFGSANVPLKDG-----NFASNLQKAAL--------------------------------------------------------
    CBS385.49_g400.t2               ---E-QPLVAW----------ESLTP-AARAALQNTDFGSANVPLKDG-----NFASNLQKAAL--------------------------------------------------------
    CBS385.49_g1381.t1              RWGVGAGLIEW----------ERMPD-NLRKTLSAKNWGAAEMAVRDKDGSDWNFAWYINESQY------F----------------C-W-----ETYCPG-------------------
    CBS385.49_g5607.t1              ---T-YPISDW----------DAMPQ-AARDALETTDFGSANVPFKDA-----NFDSNLAKAAL--------------------------------------------------------
    CBS385.49_g7723.t1              YKS---PLVGW----------LSWPNEGLRQTMLG-AFSGGVGPKLDD-----EFAGKLGEAAGDAVPE--------------------FDPNVDE------------------------
    CBS385.49_g8721.t1              IRG---GLVSM----------LLMPS-DWQDKFRSHGWGSAHMAWANED----DFTGHLVKSMPQEARDD------------------GFDCAYDENPS---------------------
    CQ2_g254.t1                     IRG---GTVSM----------LLMPS-DWQDKFRSHGWGSAHMAWANED----DFTGHLVKSMPQEARDD------------------GFDCAYDENPA---------------------
    CQ2_g2332.t3                    QLG---DLVGW----------FGFPTTELRSALETHHFGSASFALGG------SFTNNLKNSLTAWCDDKFPPDGGDLAVCKMQHFPHPFDPARDDNGSPGQPPE--GNGCNPTN---PG
    CQ2_g2332.t4                    QLG---DL-------------FHEQSEELPDCLV--------------------------------------------------------------------------------------
    CQ2_g3243.t1                    ---E-QPLVAW----------ESLTP-AARAALQNTDFGSANVPLKDG-----NFASNLQKAAL--------------------------------------------------------
    CQ2_g3454.t1                    ---T-YWIWDW----------DRMDT-TVQGALNRADFGSANCPFNNN-----NFERNMRAAF---------------------------------------------------------
    CQ2_g3584.t1                    ITG---PLLGY----------FGWDTVEQRDRMLTHNWEAGSIAIKNE-----NFAENIRKARPAGL---------------------VFDENFDDEGT---------------------
    CQ2_g4742.t1                    YKS---PLVGW----------LSWPNEGLRQTMLG-AFSGGVGPKLDD-----EFAGKLGEAAGDAVPE--------------------FDPNVDE------------------------
    CQ2_g7512.t1                    ---T-YPISDW----------DAMPQ-AARDALETTDFGSANVPFKDA-----NFDSNLAKAAL--------------------------------------------------------
    CQ2_g8820.t1                    RWGVGAGLIEW----------ERMPD-NLRKTLSAKNWGAAEMA----------------------------------------------------------------------------
    DAR82592_g1639.t1               --------ISR----------L----------------GSAHMAWANED----DFTGHLVKSMPQEARDD------------------GFDCAYDENPA---------------------
    DAR82592_g8837.t1               RWGVGAGLIEW----------ERMPD-NLRKTLSAKNWGAAEMAVRDKDGSDWNFAWYINESQY------F----------------C-W-----ETYCPG-------------------
    DAR82592_g4435.t1               ---T-YWIWDW----------DRMDT-TVQGALNRADFGSANCPFNNN-----NFERNMRAAF---------------------------------------------------------
    DAR82592_g4647.t1               ---E-QPLVAW----------ESLTP-AARAALQNTDFGSANVPLKDG-----NFASNLQKAAL--------------------------------------------------------
    DAR82592_g7268.t1               ---T-YPISDW----------DAMPQ-AARDALETTDFGSANVPFKDA-----NFDSNLAKAAL--------------------------------------------------------
    DAR82592_g4307.t1               ITG---PLLGY----------FGWDTVEQRDRMLTHNWEAGSIAIKNE-----NFAENIRKARPAGL---------------------VFDENFDDEGT---------------------
    DAR82592_g3508.t1               QLG---DL-------------FHEQSEELPDCLV--------------------------------------------------------------------------------------
    DAR82592_g62.t1                 YKS---PLVGW----------LSWPNEGLRQTMLG-AFSGGVGPKLDD-----EFAGKLGEAAGDAVPE--------------------FDPNVDE------------------------
    DAR83138_g1199.t1               IRG---GTVSM----------LLMPS-DWQDKFRSHGWGSAHMAWANED----DFTGHLVKSMPQEARDD------------------GFDCAYDENPA---------------------
    DAR83138_g60.t1                 YKS---PLVGW----------LSWPNEGLRQTMLG-AFSGGVGPKLDD-----EFAGKLGEAAGDAVPE--------------------FDPNVDE------------------------
    DAR83138_g7641.t1               ---T-YPISDW----------DAMPQ-AARDALETTDFGSANVPFKDA-----NFDSNLAKAAL--------------------------------------------------------
    DAR83138_g9085.t1               RWGVGAGLIEW----------ERMPD-NLRKTLSAKNWGAAEMAVRDKDGSDWNFAWYINESQY------F----------------C-W-----ETYCPG-------------------
    DAR83138_g3565.t3               QLG---DLVGW----------FGFPTTELRSALETHHFGSASFALGG------SFTNNLKNSLTAWCDDKFPPDGGDLAVCKMQHFPHPFDPARDDNGSPGQPPE--GNGCNPTN---PG
    DAR83138_g4173.t1               ITG---PLLGY----------FGWDTVEQRDRMLTHNWEAGSIAIKNE-----NFAENIRKARPAGL---------------------VFDENFDDEGT---------------------
    DAR83138_g4314.t1               ---T-YWIWDW----------DRMDT-TVQGALNRADFGSANCPFNNN-----NFERNMRAAF---------------------------------------------------------
    DAR83138_g4535.t1               ---E-QPLVAW----------ESLTP-AARAALQNTDFGSANVPLKDG-----NFASNLQKAAL--------------------------------------------------------
    DAR83143_g1427.t1               IRG---GTVSM----------LLMPS-DWQDKFRSHGWGSAHMAWANED----DFTGHLVKSMPQEARDD------------------GFDCAYDENPA---------------------
    DAR83143_g4194.t1               ITG---PLLGY----------FGWDTVEQRDRMLTHNWEAGSIAIKNE-----NFAENIRKARPAGL---------------------VFDENFDDEGT---------------------
    DAR83143_g4335.t1               ---T-YWIWDW----------DRMDT-TVQGALNRADFGSANCPFNNN-----NFERNMRAAF---------------------------------------------------------
    DAR83143_g4545.t1               ---E-QPLVAW----------ESLTP-AARAALQNTDFGSANVPLKDG-----NFASNLQKAAL--------------------------------------------------------
    DAR83143_g7287.t1               ---T-YPISDW----------DAMPQ-AARDALETTDFGSANVPFKDA-----NFDSNLAKAAL--------------------------------------------------------
    DAR83143_g3521.t1               QLG---DLV---------------------------------------------------------------------------------------------------------------
    DAR83143_g8715.t1               RWGVGAGLIEW----------ERMPD-NLRKTLSAKNWGAAEMAVRDKDGSDWNFAWYINESQY------F----------------C-W-----ETYCPG-------------------
    DAR83143_g61.t1                 YKS---PLVGW----------LSWPNEGLRQTMLG-AFSGGVGPKLDD-----EFAGKLGEAAGDAVPE--------------------FDPNVDE------------------------
    DAR83175_g3551.t1               QLG---DL-------------FHEQSEELPDCLV--------------------------------------------------------------------------------------
    DAR83175_g4514.t1               ---E-QPLVAW----------ESLTP-AARAALQNTDFGSANVPLKDG-----NFASNLQKAAL--------------------------------------------------------
    DAR83175_g4731.t1               ---T-YWIWDW----------DRMDT-TVQGALNRADFGSANCPFNNN-----NFERNMRAAF---------------------------------------------------------
    DAR83175_g7439.t1               ---T-YPISDW----------DAMPQ-AARDALETTDFGSANVPFKDA-----NFDSNLAKAAL--------------------------------------------------------
    DAR83175_g2075.t1               IRG---GTVSM----------LLMPS-DWQDKFRSHGWGSAHMAWANED----DFTGHLVKSMPQEARDD------------------GFDCAYDENPA---------------------
    DAR83175_g5817.t1               ITG---PLLGY----------FGWDTVEQRDRMLTHNWEAGSIAIKNE-----NFAENIRTARPAGL---------------------VFDENFDDEGT---------------------
    DAR83175_g61.t1                 YKS---PLVGW----------LSWPNEGLRQTMLG-AFSGGVGPKLDD-----EFAGKLGEAAGDAVPE--------------------FDPNVDE------------------------
    DAR83175_g8890.t1               RWGVGAGLIEW----------ERMPD-NLRKTLSAKNWGAAEMAVRDKDGSDWNFAWYINESQY------F----------------C-W-----ETYCPG-------------------
    DK045_g927.t1                   ---E-QPLVAW----------ESLTP-AARAALQNTDFGSANVPLKDG-----NFASNLQKAAL--------------------------------------------------------
    DK045_g1138.t1                  ---T-YWIWDW----------DRMDT-TVQGALNRADFGSANCPFNNN-----NFERNMRAAF---------------------------------------------------------
    DK045_g1272.t1                  ITG---PLLGY----------FGWDTVEQRDRMLTHNWEAGSIAIKNE-----NFAENIRKARPAGL---------------------VFDENFDDEGT---------------------
    DK045_g2451.t1                  YKS---PLVGW----------LSWPNEGLRQTMLG-AFSGGVGPKLDD-----EFAGKLGEAAGDAVPE--------------------FDPNVDE------------------------
    DK045_g3667.t1                  IRG---GLVSM----------LLMPS-DWQDKFRSHGWGSAHMAWANED----DFTGHLVKSMPQEARDD------------------GFDCAYDENPS---------------------
    DK045_g5985.t1                  RWGVGAGLIEW----------ERMPD-NLRKTLSAKNWGAAEMAVRDKDGSDWNFAWYINESQY------F----------------C-W-----ETYCPG-------------------
    DK045_g9032.t1                  ---T-YPISDW----------DAMPQ-AARDALETTDFGSANVPFKDA-----NFDSNLAKAAL--------------------------------------------------------
    GF1192_g8987.t1                 ---T-YWIWDW----------DRMDT-TVQGALNRADFGSANCPFNNN-----NFERNMRAAF---------------------------------------------------------
    GF1192_g9126.t1                 ITG---PLLGY----------FGWDTVEQRDRMLTHNWEAGSIAIKNE-----NFAENIRKARPAGL---------------------VFDENFDDEGT---------------------
    GF1192_g257.t1                  IRG---GLVSM----------LLMPS-DWQDKFRSHGWGSAHMAWANED----DFTGHLVKSMPQEARDD------------------GFDCAYDENPS---------------------
    GF1192_g1728.t1                 ---T-YPISDW----------DAMPQ-AARDALETTDFGSANVPFKDA-----NFDSNLAKAAL--------------------------------------------------------
    GF1192_g2840.t1                 YKS---PLVGW----------LSWPNEGLRQTMLG-AFSGGVGPKLDD-----EFAGKLGEAAGDAVPE--------------------FDPNVDE------------------------
    GF1192_g5025.t1                 ---E-QPLVAW----------ESLTP-AARAALQNTDFGSANVPLKDG-----NFASNLQKAAL--------------------------------------------------------
    GF1192_g6069.t1                 RWGVGAGLIEW----------ERMPD-NLRKTLSAKNWGAAEMAVRDKDGSDWNFAWYINESQY------F----------------C-W-----ETYCPG-------------------
    GF1207_g2093.t1                 IRG---GLVSM----------LLMPS-DWQDKFRSHGWG----------------------SMPQEARDD------------------GFDCAYDENPS---------------------
    GF1207_g4890.t1                 YKS---PLVGW----------LSWPNEGLRQTMLG-AFSGGVGPKLDD-----EFAGKLGEAAGDAVPE--------------------FDPNVDE------------------------
    GF1207_g5214.t1                 RWGVGAGLIEW----------ERMPD-NLRKTLSAKNWGAAEMAVRDKDGSDWNFAWYINESQY------F----------------C-W-----ETYCPG-------------------
    GF1207_g6669.t1                 ---E-QPLVAW----------ESLTP-AARAALQNTDFGSANVPLKDG-----NFASNLQKAAL--------------------------------------------------------
    GF1207_g6876.t1                 ---T-YWIWDW----------DRMDT-TVQGALNRADFGSANCPFNNN-----NFERNMRAAF---------------------------------------------------------
    GF1207_g6999.t1                 ITG---PLLGY----------FGWDTVEQRDRMLTHNWEAGSIAIKNE-----NFAENIRKARPAGL---------------------VFDENFDDEGT---------------------
    GF1207_g7831.t1                 ---T-YPISDW----------DAMPQ-AARDALETTDFGSANAAL---------------------------------------------------------------------------
    GF1300_g9576.t1                 ITG---PLLGY----------FGWDTVEQRDRMLTHNWEAGSIAIKNE-----NFAENIRKARPAGL---------------------VFDENFDDEGT---------------------
    GF1300_g9714.t1                 ---T-YWIWDW----------DRMDT-TVQGALNRADFGSANCPFNNN-----NFERNMRAAF---------------------------------------------------------
    GF1300_g259.t1                  IRG---GLVSM----------LLMPS-DWQDKFRSHGWGSAHMAWANED----DFTGHLVKSMPQEARDD------------------GFDCAYDENPS---------------------
    GF1300_g1512.t1                 YKS---PLVGW----------LSWPNEGLRQTMLG-AFSGGVGPKLDD-----EFAGKLGEAAGDAVPE--------------------FDPNVDE------------------------
    GF1300_g3582.t1                 RWGVGAGLIEW----------ERMPD-NLRKTLSAKNWGAAEMAVRDKDGSDWNFAWYINESQY------F----------------C-W-----ETYCPG-------------------
    GF1300_g3720.t1                 ---E-QPLVAW----------ESLTP-AARAALQNTDFGSANVPLKDG-----NFASNLQKAAL--------------------------------------------------------
    GF1300_g6171.t1                 ---T-YPISDW----------DAMPQ-AARDALETTDFGSANVPFKDA-----NFDSNLAKAAL--------------------------------------------------------
    Gf-Ca2_g111.t1                  YKS---PLVGW----------LSWPNEGLRQTMLG-AFSGGVGPKLDD-----EFAGKLGEAAGDAVPE--------------------FDPNVDE------------------------
    Gf-Ca2_g2398.t1                 IRG---GLVSM----------LLMPS-DWQD--------NAHMAWANED----DFTGHLVKSMPQEARDD------------------GFDCAYDENPS---------------------
    Gf-Ca2_g3362.t1                 ITG---PLLGY----------FGWDTVEQRDRMLTHNWEAGSIAIKNE-----NFAENIRKARPAGL---------------------VFDENFDDEGT---------------------
    Gf-Ca2_g3481.t1                 ---T-YWIWDW----------DRMDT-TVQGALNRADFGSANCPFNNN-----NFERNMRAAF---------------------------------------------------------
    Gf-Ca2_g3687.t1                 ---E-QPLVAW----------ESLTP-AARAALQNTDFGSANVPLKDG-----NFASNLQKAAL--------------------------------------------------------
    Gf-Ca2_g4661.t1                 RWGVGAGLIEW----------ERMPD-NLRKTLSAKNWGAAEMAVRDKDGSDWNFAWYINESQY------F----------------C-W-----ETYCPG-------------------
    Gf-Ca2_g8346.t1                 ---T-YPISDW----------DAMPQ-AARDALETTDFGSANVPFKDA-----NFDSNLAKAAL--------------------------------------------------------
    Gf-Cb5_g260.t1                  IRG---GLVSM----------LLMPS-DWQDKFRSHGWGSAHMAWANED----DFTGHLVKSMPQEARDD------------------GFDCAYDENPS---------------------
    Gf-Cb5_g3949.t1                 ---T-YWIWDW----------DRMDT-TVQGALNRADFGSANCPFNNN-----NFERNMRAAF---------------------------------------------------------
    Gf-Cb5_g4084.t1                 ITG---PLLGY----------FGWDTVEQRDRMLTHNWEAGSIAIKNE-----NFAENIRKARPAGL---------------------VFDENFDDEGT---------------------
    Gf-Cb5_g4605.t1                 ---T-YPISDW----------DAMPQ-AARDALETTDFGSANVPFKDA-----NFDSNLAKAAL--------------------------------------------------------
    Gf-Cb5_g6249.t1                 MTG---NLAIWLVGLDFRQQSFGRPWKPIISAVRA-------------------------------------------------------------------------------------
    Gf-Cb5_g7167.t1                 YKS---PLVGW----------LSWPNEGLRQTMLG-AFSGGVGPKLDD-----EFAGKLGEAAGDAVPE--------------------FDPNVDE------------------------
    Gf-Cb5_g7934.t1                 ---E-QPLVAW----------ESLTP-AARAALQNTDFGSANVPLKDG-----NFASNLQKAAL--------------------------------------------------------
    Gf-Cb5_g8589.t1                 RWGVGAGLIEW----------ERMPD-NLRKTLSAKNWGAAEMAVRDKDGSDWNFAWYINESQY------F----------------C-W-----ETYCPG-------------------
    HoMCF_g1490.t1                  RWGVGAGLIEW----------ERMPD-NLRKTLSAKNWGAAEMA----------------------------------------------------------------------------
    HoMCF_g2875.t1                  ----------------------------RRDALETTDFGSANVPFKDA-----NFDSNLAKAAL--------------------------------------------------------
    HoMCF_g3274.t1                  ---T-YWIWDW----------DRMDT-TVQGALNRADFGSANCPFNNN-----NFERNMRAAF---------------------------------------------------------
    HoMCF_g4303.t1                  -------------------------------QVQISRLGSAHMAWANED----DFTGHLVKSMPQEARDD------------------GFDCAYDENPA---------------------
    HoMCF_g5967.t1                  ITG---PLLGY----------FGWDTVEQRDRMLTHNWEAGSIAIKNE-----NFAENIRKARPAGL---------------------VFDENFDDEGT---------------------
    HoMCF_g6093.t1                  ---T-YWIWDW----------DRMDT-T----------------------------RNMRAAF---------------------------------------------------------
    HoMCF_g6096.t1                  ---T-YWIWDW----------DRMDT-TVQGALNRADFGSANCPF-NN-----NFERNMRAAF---------------------------------------------------------
    HoMCF_g6549.t1                  ---E-QPLVAW----------ESLTP-AARAALQNTDFGSANVPLKDG-----NFASNLQKAAL--------------------------------------------------------
    HoMCF_g10173.t1                 YKS---PLVGW----------LSWPNEGLRQTMLG-AFSGGVGPKLDD-----EFAGKLGEAAGDAVPE--------------------FDPNVDE------------------------
    HoMCLT_g1190.t1                 IRG---GLVSM----------LLMPS-DWQDKFRSHGWGSAHMAWANED----DFTGHLVKSMPQEARDD------------------GFDCAYDENPS---------------------
    HoMCLT_g2195.t1                 ---T-YPISDW----------DAMPQ-AARDALETTDFGSANVPFKDA-----NFDSNLAKAAL--------------------------------------------------------
    HoMCLT_g2713.t1                 RWGVGAGLIEW----------ERMPD-NLRKTLSAKNWGAAEMAVRDKDGSDWNFAWYINESQY------F----------------C-W-----ETYCPG-------------------
    HoMCLT_g3698.t1                 ---E-QPLVAW----------ESLTP-AARAALQNTDFGSANVPLKDG-----NFASNLQKAAL--------------------------------------------------------
    HoMCLT_g4981.t1                 YKS---PLVGW----------LSWPNEGLRQTMLG-AFSGGVGPKLDD-----EFAGKLGEAAGDAVPE--------------------FDPNVDE------------------------
    HoMCLT_g6335.t1                 ---T-YWIWDW----------DRMDT-TVQGALNRADFGSANCPFNNN-----NFERNMRAAF---------------------------------------------------------
    HoMCLT_g6471.t1                 ITG---PLLGY----------FGWDTVEQRDRMLTHNWEAGSIAIKNE-----NFAENIRKARPAGL---------------------VFDENFDDEGT---------------------
    I1V_g7945.t1                    ---T-YPISDW----------DAMPQ-AARDALETTDFGSANVPFKDA-----NFDSNLAKAAL--------------------------------------------------------
    I1V_g9550.t1                    ---T-YWIWDW----------DRMDT-TVQGALNRADFGSANCPFNNN-----NFERNMRAAF---------------------------------------------------------
    I1V_g9695.t1                    ------------------------------------------------------------------------------------------------------------------------
    I1V_g10783.t1                   ---E-QPLVAW----------ESLTP-AARAALQNTDFGSANVPLKDG-----NFASNLQKAAL--------------------------------------------------------
    I1V_g12440.t1                   ITG---PLLGY----------FGWDT-------------AGSIAIKNE-----NFAENIRKARPAGL---------------------VFDENFDDEGT---------------------
    I1V_g13776.t1                   RWGVGAGLIEW----------ERMPD-NLRKTLSAKN------------GSDWNFAWYINESQY------F----------------C-W-----ETYCPG-------------------
    MPI-CAGE_g5591.t1               YKS---PLVGW----------LSWPNEGLRQTMLG-AFSGGVGPKLDD-----EFAGKLGEAAGDAVPE--------------------FDPNVDE------------------------
    MPI-CAGE_g8268.t1               ---T-YWIWDW----------DRMDT-TVQGALNRADFGSANCPFNNN-----NFERNMRAAF---------------------------------------------------------
    MPI-CAGE_g8414.t1               ITG---PLLGY----------FGWDTVEQRDRMLTHNWEAGSIAIKNE-----NFAENIRKARPAGL---------------------VFDENFDDEGT---------------------
    MPI-CAGE_g9956.t1               ---E-QPLVAW----------ESLTP-AARAALQNTDFGSANVPLKDG-----NFASNLQKAAL--------------------------------------------------------
    MPI-CAGE_g1577.t1               IRG---GLVSM----------LLMPS-DWQDKFRSHGWGSAHMAWANED----DFTGHLVKSMPQEARDD------------------GFDCAYDENPA---------------------
    MPI-CAGE_g2204.t1               QLG---DLVGW----------FGFPTTELRSALETHHFGSASFALGG------SFTNNLKNSLTAWCDDKFPPDGGDLAVCKMQHFPHPFDPARDDNGSPGQPPE--GNGCNPTN---PG
    MPI-CAGE_g10572.t1              ITG---PLLGY----------FGWDTVEQRDRMLTHNWEAGSIAIKNE-----NFAENIRKARPAGL---------------------VFDENFDDEGT---------------------
    MPI-CAGE_g3967.t1               RWGVGAGLIEW----------ERMPD-NLRKTLSAKNWGAAEMAVRDKDGSDWNFAWYINESQY------F----------------C-W-----ETYCPG-------------------
    MPI-CAGE_g4142.t1               ---T-YPISDW----------DAMPQ-AARDALETTDFGSANVPFKDA-----NFDSNLAKAAL--------------------------------------------------------
    S011_g7486.t1                   QLG---DL-------------FHEQSEELPDCLV--------------------------------------------------------------------------------------
    S011_g8092.t1                   ---T-YPISDW----------DAMPQ-AARDALETTDFGSANVPFKDA-----NFDSNLAKAAL--------------------------------------------------------
    S011_g8657.t1                   ITG---PLLGY----------FGWDTVEQRDRMLTHNWEAGSIAIKNE-----NFAENIRKARPAGL---------------------VFDENFDDEGT---------------------
    S011_g8986.t1                   ---E-QPLVAW----------ESLTP-AARAALQNTDFGSANVPLKDG-----NFASNLQKAAL--------------------------------------------------------
    S011_g9203.t1                   ---T-YWIWDW----------DRMDT-TVQGALNRADFGSANCPFNNN-----NFERNMRAAF---------------------------------------------------------
    S011_g1207.t1                   IRG---GLVSM----------LLMPS-DWQDKFRSHGWGSAHMAWANED----DFTGHLVKSMPQEARDD------------------GFDCAYDENPA---------------------
    S011_g2838.t1                   YKS---PLVGW----------LSWPNEGLRQTMLG-AFSGGVGPKLDD-----EFAGKLGEAAGDAVPE--------------------FDPNVDE------------------------
    S011_g5817.t1                   RWGVGAGLIEW----------ERMPD-NLRKTLSAKNWGAAEMAVRDKDGSDWNFAWYINESQY------F----------------C-W-----ETYCPG-------------------
    S023_g1908.t1                   IRG---GLVSM----------LLMPS-DWQDKFRSHGWGSAHMAWANED----DFTGHLVKSMPQEARDD------------------GFDCAYDENPA---------------------
    S023_g3148.t1                   ---E-QPLVAW----------ESLTP-AARAALQNTDFGSANVPLKDG-----NFASNLQKAAL--------------------------------------------------------
    S023_g4559.t1                   YKS---PLVGW----------LSWPNEGLRQTMLG-AFSGGVGPKLDD-----EFAGKLGEAAGDAVPE--------------------FDPNVDE------------------------
    S023_g5760.t1                   ITG---PLLGY----------FGWDTVEQRDRMLTHNWEAGSIAIKNE-----NFAENIRKARPAGL---------------------VFDENFDDEGT---------------------
    S023_g5904.t1                   ---T-YWIWDW----------DRMDT-TVQGALNRADFGSANCPFNNN-----NFERNMRAAF---------------------------------------------------------
    S023_g7666.t1                   RWGVGAGLIEW----------ERMPD-NLRKTLSAKNWGAAEMAVRDKDGSDWNFAWYINESQY------F----------------C-W-----ETYCPG-------------------
    S023_g9104.t1                   ---T-YPISDW----------DAMPQ-AARDALETTDFGSANVPFKDA-----NFDSNLAKAAL--------------------------------------------------------
    TO22_g2077.t1                   IRG---GLVSM----------LLMPS-DWQDKFRSHGWGSAHMAWANED----DFTGHLVKSMPQEARDD------------------GFDCAYDENPS---------------------
    TO22_g2405.t1                   ITG---PLLGY----------FGWDTVEQRDRMLTHNWEAGSIAIKNE-----NFAENIRKARPAGL---------------------VFDENFDDEGT---------------------
    TO22_g2541.t1                   ---T-YWIWDW----------DRMDT-TVQGALNRADFGSANCPFNNN-----NFERNMRAAF---------------------------------------------------------
    TO22_g2749.t1                   ---E-QPLVAW----------ESLTP-AARAALQNTDFGSANVPLKDG-----NFASNLQKAAL--------------------------------------------------------
    TO22_g3776.t1                   YKS---PLVGW----------LSWPNEGLRQTMLG-AFSGGVGPKLDD-----EFAGKLGEAAGDAVPE--------------------FDPNVDE------------------------
    TO22_g6379.t1                   ---T-YPISDW----------DAMPQ-AARDALETTDFGSANVPFKDA-----NFDSNLAKAAL--------------------------------------------------------
    TO22_g9457.t1                   RWGVGAGLIEW----------ERMPD-NLRKTLSAKNWGAAEMAVRDKDGSDWNFAWYINESQY------F----------------C-W-----ETYCPG-------------------
    Ud1-4-1_g9889.t1                RWGVGAGLIEW----------ERMPD-NLRKTLSAKNWGAAEMAVRDKDGSDWNFAWYINESQY------F----------------C-W-----ETYCPG-------------------
    Ud1-4-1_g261.t1                 IRG---GLVSM----------LLMPS-DWQDKFRSHGWGSAHMAWANED----DFTGHLVKSMPQEARDD------------------GFDCAYDENPS---------------------
    Ud1-4-1_g2536.t1                YKS---PLVGW----------LSWPNEGLRQTMLG-AFSGGVGPKLDD-----EFAGKLGEAAGDAVPE--------------------FDPNVDE------------------------
    Ud1-4-1_g2721.t1                ITG---PLLGY----------FGWDTVEQRDRMLTHNWEAGSIAIKNE-----NFAENIRKARPAGL---------------------VFDENFDDEGT---------------------
    Ud1-4-1_g2858.t1                ---T-YWIWDW----------DRMDT-TVQGALNRADFGSANCPFNNN-----NFERNMRAAF---------------------------------------------------------
    Ud1-4-1_g3064.t1                ---E-QPLVAW----------ESLTP-AARAALQNTDFGSANVPLKDG-----NFASNLQKAAL--------------------------------------------------------
    Ud1-4-1_g3975.t1                QLG---DLV---------------------------------------------------------------------------------------------------------------
    Ud1-4-1_g4565.t1                ---T-YPISDW----------DAMPQ-AARDALETTDFGSANVPFKDA-----NFDSNLAKAAL--------------------------------------------------------
    V13_g3278.t1                    ITG---PLLGY----------FGWDTVEQRDRMLTHNWEAGSIAIKNE-----NFAENIRKARPAGL---------------------VFDENFDDEGT---------------------
    V13_g3397.t1                    ---T-YWIWDW----------DRMDT-TRQ------------LPFNNN-----NFERNMRAAF---------------------------------------------------------
    V13_g4590.t1                    ---T-YPSPTG----------TPCPG-GARRARDDRLW-QRQCAFKDA-----NFDSNLAKAAL--------------------------------------------------------
    V13_g5067.t1                    TR----------------------------------ARSGGVGPKLDD-----EFAGKLGEAAGDAVPE--------------------FDPNVDE------------------------
    V13_g6708.t1                    QGRVPQGRRAD----------PRLPL-GQRRRRAAGE-PLEELALGR-----------------------------------------------------G-------------------
    V13_g9344.t1                    QLG---DLV---------------------------------------------------------------------------------------------------------------
    V13_g9739.t1                    ---R-AAAVAW----------ESLTP-AARRRCRTRTLEAAL------------------------------------------------------------------------------
    V13_g10958.t1                   --------ISR----------L----------------GSAHMAWANED----DFTGHLVKSMPQEARDD------------------GFDCAYDENPA---------------------
    Vd39_g2112.t1                   QLG---DLVGW----------FGFPTTELRSALETHHFGSASFALGG------SFTNNLKNSLTAWCDDKFPPDGGDLAVCKMQHFPHPFDPARDDNGSPGQPPE--GNGCNPTN---PG
    Vd39_g2112.t3                   QLG---DL-------------FHEQSEELPDCLV--------------------------------------------------------------------------------------
    Vd39_g2716.t1                   IRG---GLVSM----------LLMPS-DWQDKFRSHGWGSAHMAWANED----DFTGHLVKSMPQEARDD------------------GFDCAYDENPA---------------------
    Vd39_g3506.t1                   ITG---PLLGY----------FGWDTVEQRDRMLTHNWEAGSIAIKNE-----NFAENIRKARPAGL---------------------VFDENFDDEGT---------------------
    Vd39_g3907.t1                   RWGVGAGLIEW----------ERMPD-NLRKTLSAKNWGAAEMAVRDKDGSDWNFAWYINESQY------F----------------C-W-----ETYCPG-------------------
    Vd39_g4379.t1                   YKS---PLVGW----------LSWPNEGLRQTMLG-AFSGGVGPKLDD-----EFAGKLGEAAGDAVPE--------------------FDPNVDE------------------------
    Vd39_g7329.t1                   ---T-YPISDW----------DAMPQ-AARDALETTDFGSANVPFKDA-----NFDSNLAKAAL--------------------------------------------------------
    Vd39_g8964.t1                   ---E-QPLVAW----------ESLTP-AARAALQNTDFGSANVPLKDG-----NFASNLQKAAL--------------------------------------------------------
    Vd39_g9298.t1                   ---T-YWIWDW----------DRMDT-TVQGALNRADFGSANCPFNNN-----NFERNMRAAF---------------------------------------------------------
    Vd-653_g1291.t1                 ---T-YWIWDW----------DRMDT-TVQGALNRADFGSANCPFNNN-----NFERNMRAAF---------------------------------------------------------
    Vd-653_g2557.t1                 IRG---GLVSM----------LLMPS-DWQDKFRSHGWGSAHMAWANED----DFTGHLVKSMPQEARDD------------------GFDCAYDENPA---------------------
    Vd-653_g3339.t1                 RWGVGAGLIEW----------ERMPD-NLRKTLSAKNWGAAEMAVRDKDGSDWNFAWYINESQY------F----------------C-W-----ETYCPG-------------------
    Vd-653_g3685.t1                 -------------------------------XLQNTDFGSANVPLKDG-----NFASNLQKAAL--------------------------------------------------------
    Vd-653_g3686.t1                 ---E-QPL--------------------------NTDFGSANVPLKDG-----NFASNLQKAAL--------------------------------------------------------
    Vd-653_g5777.t1                 ITG---PLLGY----------FGWDTVEQRDRMLTHNWEAGSIAIKNE-----NFAENIRKARPAGL---------------------VFDENFDDEGT---------------------
    Vd-653_g8550.t1                 ---T-YPISDW----------DAMPQ-AARDALETTDFGSANVPFKDA-----NFDSNLAKAAL--------------------------------------------------------
    Vd-653_g9566.t1                 TR----------------------------------ARSGGVGPKLDD-----EFAGKLGEAAGDAVPE--------------------FDPNVDE------------------------
    VD991_g2070.t1                  IRG---GTVSM----------LLMPS-DWQDKFRSHGWGSAHMAWANED----DFTGHLVKSMPQEARDD------------------GFDCAYDENPA---------------------
    VD991_g2394.t1                  ITG---PLLGY----------FGWDTVEQRDRMLTHNWEAGSIAIKNE-----NFAENIRKARPAGL---------------------VFDENFDDEGT---------------------
    VD991_g2531.t1                  ---T-YWIWDW----------DRMDT-TVQGALNRADFGSANCPFNNN-----NFERNMRAAF---------------------------------------------------------
    VD991_g2742.t1                  ---E-QPLVAW----------ESLTP-AARAALQNTDFGSANVPLKDG-----NFASNLQKAAL--------------------------------------------------------
    VD991_g3653.t1                  QLG---DLVGW----------FGFPTTELRSALETHHFGSASFALGG------SFTNNLKNSLTAWCDDKFPPDGGDLAVCKMQHFPHPFDPARDDNGSPGQPPE--GNGCNPTN---PG
    VD991_g3653.t2                  QLG---DLVGW----------FGFPTTELRSALETHHFGSASFALGG------SFTNNLKNSLTAWCDDKFPPDGGDLAVCKMQHFPHPFDPARDDNGSPGQPPE--GNGCNPTN---PG
    VD991_g3719.t1                  YKS---PLVGW----------LSWPNEGLRQTMLG-AFSGGVGPKLDD-----EFAGKLGEAAGDAVPE--------------------FDPNVDE------------------------
    VD991_g7479.t1                  ---T-YPISDW----------DAMPQ-AARDALETTDFGSANVPFKDA-----NFDSNLAKAAL--------------------------------------------------------
    VD991_g8969.t1                  RWGVGAGLIEW----------ERMPD-NLRKTLSAKNWGAAEMAVRDKDGSDWNFAWYINESQY------F----------------C-W-----ETYCPG-------------------
    VdB09_g1055.t1                  ITG---PLLGY----------FGWDTVEQRDRMLTHNWEAGSIAIKNE-----NFAENIRKARPAGL---------------------VFDENFDDEGT---------------------
    VdB09_g1685.t1                  RWGVGAGLIEW----------ERMPD-NLRKTLSAKNWGAAEMAVRDKDGSDWNFAWYINESQY------F----------------C-W-----ETYCPG-------------------
    VdB09_g1751.t1                  YKS---PLVGW----------LSWPNEGLRQTMLG-AFSGGVGPKLDD-----EFAGKLGEAAGDAVPE--------------------FDPNVDE------------------------
    VdB09_g3918.t1                  QLG---DL-------------FHEQSEELPDCLV--------------------------------------------------------------------------------------
    VdB09_g4877.t1                  ---E-QPLVAW----------ESLTP-AARAALQNTDFGSANVPLKDG-----NFASNLQKAAL--------------------------------------------------------
    VdB09_g5096.t1                  ---T-YWIWDW----------DRMDT-TVQGALNRADFGSANCPFNNN-----NFERNMRAAF---------------------------------------------------------
    VdB09_g9703.t1                  ---T-YPISDW----------DAMPQ-AARDALETTDFGSANVPFKDA-----NFDSNLAKAAL--------------------------------------------------------
    VdB09_g7661.t1                  ------------------------------DKFRSHGWGSAHMAWANED----DFTGHLVKSMPQEARDD------------------GFDCAYDENPS---------------------
    VdC07_g4126.t1                  ITG---PLLGY----------FGWDTVEQRDRMLTHNWEAGSIAIKNE-----NFAENIRKARPAGL---------------------VFDENFDDEGT---------------------
    VdC07_g4261.t1                  ---T-YWIWDW----------DRMDT-TVQGALNRADFGSANCPFNNN-----NFERNMRAAF---------------------------------------------------------
    VdC07_g4482.t1                  ---E-QPLVAW----------ESLTP-AARAALQNTDFGSANVPLKDG-----NFASNLQKAAL--------------------------------------------------------
    VdC07_g605.t1                   ---T-YPISDW----------DAMPQ-AARDALETTDFGSANVPFKDA-----NFDSNLAKAAL--------------------------------------------------------
    VdC07_g1872.t1                  RWGVGAGLIEW----------ERMPD-NLRKTL------------------DWNFAWYINESQY------F----------------C-W-----ETYCPG-------------------
    VdC07_g2986.t1                  YKS---PLVGW----------LSWPNEGLRQTMLG-AFSGGVGPKLDD-----EFAGKLGEAAGDAVPE--------------------FDPNVDE------------------------
    VdC07_g7672.t1                  IRG---GLVSM----------LLMPS-DWQDKFRSHGWGSAHMAWANED----DFTGHLVKSMPQEARDD------------------GFDCAYDENPS---------------------
    Vd-H5_g2477.t1                  ---E-QPL--------------------------NTDFGSANVPLKDG-----NFASNLQKAAL--------------------------------------------------------
    Vd-H5_g5176.t1                  RWGVGAGLIEW----------ERMPD-NLRKTLSAKNW----------------------------------------------------------------------------------
    Vd-H5_g6144.t1                  YKS---PLVGW----------LSWPNEGLRQTMLG-AFSGGVGPKLDD-----EFAGKLGEAAGDAVPE--------------------FDPNVDE------------------------
    Vd-H5_g7203.t1                  ---T-YPISDW----------DAMPQ-AARDALETTDFGSANVPFKDA-----NFDSNLAKAAL--------------------------------------------------------
    Vd-H5_g8423.t1                  ITG---PLLGY----------FGWDTVEQRDRMLTHNWEAGSIAIKNE-----NFAENIRKARPAGL---------------------VFDENFDDEGT---------------------
    Vd-H5_g8546.t1                  ---T-YWIWDW----------DRMDT-TVQGALNRADFGSANCPFNNN-----NFERNMRAAF---------------------------------------------------------
    Vd-H5_g9396.t1                  --------ISR----------L----------------GSAHMAWANED----DFTGHLVKSMPQEARDD------------------GFDCAYDENPA---------------------
    VdLs16_g2071.t1                 IRG---GLVSM----------LLMPS-DWQDKFRSHGWGSAHMAWANED----DFTGHLVKSMPQEARDD------------------GFDCAYDENPS---------------------
    VdLs16_g7836.t1                 ---T-YPISDW----------DAMPQ-AARDALETTDFGSANVPFKDA-----NFDSNLAKAAL--------------------------------------------------------
    VdLs16_g8455.t1                 YKS---PLVGW----------LSWPNEGLRQTMLG-AFSGGVGPKLDD-----EFAGKLGEAAGDAVPE--------------------FDPNVDE------------------------
    VdLs16_g5349.t1                 ---E-QPLVAW----------ESLTP-AARAALQNTDFGSANVPLKDG-----NFASNLQKAAL--------------------------------------------------------
    VdLs16_g5560.t1                 ---T-YWIWDW----------DRMDT-TVQGALNRADFGSANCPFNNN-----NFERNMRAAF---------------------------------------------------------
    VdLs16_g5699.t1                 ITG---PLLGY----------FGWDTVEQRDRMLTHNWEAGSIAIKNE-----NFAENIRKARPAGL---------------------VFDENFDDEGT---------------------
    VdLs16_g6952.t1                 RWGVGAGLIEW----------ERMPD-NLRKTLSAKNWGAAEMAVRDKDGSDWNFAWYINESQY------F----------------C-W-----ETYCPG-------------------
    VdLs17_g64.t1                   YKS---PLVGW----------LSWPNEGLRQTMLG-AFSGGVGPKLDD-----EFAGKLGEAAGDAVPE--------------------FDPNVDE------------------------
    VdLs17_g1975.t1                 IRG---GLVSM----------LLMPS-DWQDKFRSHGWGSAHMAWANED----DFTGHLVKSMPQEARDD------------------GFDCAYDENPA---------------------
    VdLs17_g3499.t1                 QLG---DLV---------------------------------------------------------------------------------------------------------------
    VdLs17_g4253.t1                 ---T-YPISDW----------DAMPQ-GARDALETTDFGSANVPFKDG-----NFDSNLAKAAL--------------------------------------------------------
    VdLs17_g4391.t1                 ---T-YWIWDW----------DRMDT-TVQGALNRADFGSANCPFNNN-----NFERNMRAAF---------------------------------------------------------
    VdLs17_g5246.t1                 ITG---PLLGY----------FGWDTVEQRDRMLTHNWEAGSIAIKNE-----NFAENIRKARPAGL---------------------VFDENFDDEGT---------------------
    VdLs17_g6770.t1                 ---T-YPISDW----------DAMPQ-AARDALETTDFGSANVPFKDA-----NFDSNLAKAAL--------------------------------------------------------
    VdLs17_g8273.t1                 RWGVGAGLIEW----------ERMPD-NLRKTLSAKNWGAAEMAVRDKDGSDWNFAWYINESQY------F----------------C-W-----ETYCPG-------------------
    VdLs17_g8809.t1                 ---E-QPLVAW----------ESLTP-AARAALQNTDFGSANVPLKDG-----NFASNLQKAAL--------------------------------------------------------
    Vd-R1_g926.t1                   ---E-QPLVAW----------ESLTP-AARAALQNTDFGSANVPLKDG-----NFASNLQKAAL--------------------------------------------------------
    Vd-R1_g3679.t1                  TR----------------------------------ARSGGVGPKLDD-----EFAGKLGEAAGDAVPE--------------------FDPNVDE------------------------
    Vd-R1_g4718.t1                  ---T-YPISDW----------DAMPQ-AARDALETTDFGSANVPFKDA-----NFDSNLAKAAL--------------------------------------------------------
    Vd-R1_g5037.t1                  RWGVGAGLIEW----------ERMPD-NLRKTLSAKNWGAAEMAVRDKDGSDWNFAWYINESQY------F----------------C-W-----ETYCPG-------------------
    Vd-R1_g8886.t1                  IRG---GLVSM----------LLMPS-DWQDKFRSHGWG----------------------SMPQEARDD------------------GFDCAYDENPA---------------------
    Vd-R1_g9491.t1                  ---T-YWIWDW----------DRMDT-TVQGALNRADFGSANCPFNNN-----NFERNMRAAF---------------------------------------------------------
    Vd-R1_g9617.t1                  ITG---PLLGY----------FGWDTVEQRDRMLTHNWEAGSIAIKNE-----NFAENIRKARPAGL---------------------VFDENFDDEGT---------------------
    VT-2A_g1161.t1                  YKS---PLVGW----------LSWPNEGLRQTMLG-AFSGGVGPKLDD-----EFAGKLGEAAGDAVPE--------------------FDPNVDE------------------------
    VT-2A_g1267.t1                  RWGVGAGLIEW----------ERMPD-NLRKTLSAKNWGAAEMAVRDKDGSDWNFAWYINESQY------F----------------C-W-----ETYCPG-------------------
    VT-2A_g4726.t1                  ---T-YPISDW----------DAMPQ-AARDALETTDFGSANVPFKDA-----NFDSNLAKAAL--------------------------------------------------------
    VT-2A_g6128.t1                  ---E-QPLVAW----------ESLTP-AARAALQNTDFGSANVPLKDG-----NFASNLQKAAL--------------------------------------------------------
    VT-2A_g6128.t2                  ---E-QPLVAW----------ESLTP-AARAALQNTDFGSANVPLKDG-----NFASNLQKAAL--------------------------------------------------------
    VT-2A_g6341.t1                  ---T-YWIWDW----------DRMDT-TVQGALNRADFGSANCPFNNN-----NFERNMRAAF---------------------------------------------------------
    VT-2A_g6470.t1                  ITG---PLLGY----------FGWDT-------------------------------------PAGL---------------------VFDENFDDEGT---------------------
    VT-2A_g9510.t1                  IRG---GLVSM----------LLMPS-DWQDKFRSHGWGSAHMAWANED----DFTGHLVKSMPQEARDD------------------GFDCAYDENPS---------------------
    VT-2A_g9511.t1                  IRG---GLVSM----------LLMPS-DWQDKFRSHGWGSAHMAWANED----DFTGHLVKSMPQEARDD------------------GFDCAYDENPS---------------------
    XJ511_g1085.t1                  IRG---GLVSM----------LLMPS-DWQDKFRSHGWGSAHMAWANED----DFTGHLVKSMPQEARDD------------------GFDCAYDENPA---------------------
    XJ511_g1518.t1                  YKS---PLVGW----------LSWPNEGLRQTMLG-AFSGGVGPKLDD-----EFAGKLGEAAGDAVPE--------------------FDPNVDE------------------------
    XJ511_g4592.t1                  RWGVGAGLIEW----------ERMPD-NLRKTLSAKNWGAAEMAVRDKDGSDWNFAWYINESQY------F----------------C-W-----ETYCPG-------------------
    XJ511_g5794.t1                  ITG---PLLGY----------FGWDTVEQRDRMLTHNWEAGSIAIKNE-----NFAENIRKARPAGL---------------------VFDENFDDEGT---------------------
    XJ511_g5932.t1                  ---T-YWIWDW----------DRMDT-TVQGALNRADFGSANCPFNNN-----NFERNMRAAF---------------------------------------------------------
    XJ511_g7291.t1                  ---T-YPISDW----------DAMPQ-AARDALETTDFGSANVPFKDA-----NFDSNLAKAAL--------------------------------------------------------
    XJ511_g8237.t1                  ---E-QPLVAW----------ESLTP-AARAALQNTDFGSANVPLKDG-----NFASNLQKAAL--------------------------------------------------------
    XJ592_g261.t1                   IRG---GTVSM----------LLMPS-DWQDKFRSHGWGSAHMAWANED----DFTGHLVKSMPQEARDD------------------GFDCAYDENPA---------------------
    XJ592_g3433.t1                  YKS---PLVGW----------LSWPNEGLRQTMLG-AFSGGVGPKLDD-----EFAGKLGEAAGDAVPE--------------------FDPNVDE------------------------
    XJ592_g5005.t1                  ---T-YPISDW----------DAMPQ-AARDALETTDFGSANVPFKDA-----NFDSNLAKAAL--------------------------------------------------------
    XJ592_g6487.t1                  RWGVGAGLIEW----------ERMPD-NLRKTLSAKNWGAAEMAVRDKDGSDWNFAWYINESQY------F----------------C-W-----ETYCPG-------------------
    XJ592_g7860.t1                  ---E-QPLVAW----------ESLTP-AARAALQNTDFGSANVPLKDG-----NFASNLQKAAL--------------------------------------------------------
    XJ592_g8068.t1                  ---T-YWIWDW----------DRMDT-TVQGALNRADFGSANCPFNNN-----NFERNMRAAF---------------------------------------------------------
    XJ592_g8206.t1                  ITG---PLLGY----------FGWDTVEQRDRMLTHNWEAGSIAIKNE-----NFAENIRKARPAGL---------------------VFDENFDDEGT---------------------
    XJ592_g9637.t1                  QLG---DLVGW----------FGFPTTELRSALETHHFGSASFALGG------SFTNNLKNSLTAWCDDKFPPDGGDLAVCKMQHFPHPFDPARDDNGSPGQPPE--GNGCNPTN---PG
    XJ592_g9637.t2                  QLG---DLVGW----------FGFPTTELRSALETHHFGSASFALGG------SFTNNLKNSLTAWCDDKFPPDGGDLAVCKMQHFPHPFDPARDDNGSPGQPPE--GNGCNPTN---PG
    397_g2445.t1                    ---Y-QPIIGW----------DFMNS-NEQGTLNSVDWGDANCPINNG-----NFNNNLGKAAL--------------------------------------------------------
    397_g4853.t1                    ---T-YPISDW----------DAMPQ-ATRDALEATDFGSANVPFKDA-----NFDNNLAKAAL--------------------------------------------------------
    397_g6363.t1                    RWGVGAGLIEW----------ERLPQ-NLRTTLSEKNWGSAEMAVRDKDRSDWNFRWYINESRY------H----------------CPT-----EIYCLG-------------------
    397_g7397.t1                    YKS---PLVGW----------LSWPSEGLRNTMLG-AFSGGVGPKLDD-----EFAGKLGEAAGDAVPE--------------------FDPNVDE------------------------
    397_g7755.t1                    IRS---GLISM----------LLMPA-DWQEKFRSHDWGSAHMAWASED----DFTGHLVDSMPQEARDE------------------GFDCAYDEIAE---------------------
    397_g8350.t1                    ---E-QPLIAW----------DSLTA-AARTALQNTDFGSANVPFKDG-----NFASNLQKAAL--------------------------------------------------------
    397_g9733.t1                    ITG---PLLGY----------FGWDTAEQRDRMLTHNWEAGSVAIRNE-----NFAENIRKARPAGL---------------------AFDENRDDDGT---------------------
    CBS382.66_g255.t1               IRG---GLISM----------LLMPS-DWQEKFRSQDWGSAHMAWANED----DFTGHLVKSMPQEARDE------------------GFDCAYDENPA---------------------
    CBS382.66_g255.t2               IRG---GLISM----------LLMPS-DWQEKFRSQDWGSAHMAWANED----DFTGHLVKSMPQEARDE------------------GFDCAYDENPA---------------------
    CBS382.66_g4327.t1              ---E-QPLVAW----------ESLTP-AARAALQNTNFGSANVPFKDG-----NFASNLQKAAL--------------------------------------------------------
    CBS382.66_g5334.t1              QLG---DLVGW----------FGFPTTELRSALETHHFGSASFALGG------SFTTNLKNSLTAWCDDKFPPDGGDLAVCKMQHFPHPFDPARDDNGSPGQPPGGNGNGCNPTN---PG
    CBS382.66_g5334.t2              QLG---DLVGW----------FGFPTTELRSALETHHFGSASFALGG------SFTTNLKNSLTAWCDDKFPPDGGDLAVCKMQHFPHPFDPARDDNGSPGQPPGGNGNGCNPTN---PG
    CBS382.66_g5795.t1              ---T-YPISDW----------DAMPQ-ATRDALETTNFGSANVPFKDA-----NFDSNLAKAAL--------------------------------------------------------
    CBS382.66_g6578.t1              YKS---PLVGW----------LSWPNEGLRQTMLG-AFSGGVGPKLDD-----EFAGKLGEAAGDAVPE--------------------FDPNVDE------------------------
    CBS382.66_g8384.t1              RWGVGAGLIEW----------ERMPD-NLRKTLSAKNWGAAEMAVRDKDGSDWNFAWYINESQY------F----------------C-W-----ETYCPG-------------------
    CBS382.66_g9605.t1              ---T-YWIWDW----------DRMDS-TVQGALNRADFNRANCPFNNN-----NFERNMRAAF---------------------------------------------------------
    CBS382.66_g9740.t1              ITG---PLLGY----------FGWDTVEQRDRMLTHNWEAGSIAIKNE-----NFAENIRKARPAGL---------------------VFDENIDDEGT---------------------
    MUCL9792_g2463.t1               ---T-YWLYDW----------DAMAP-IVKSALNQGKFGKANCPINDN-----NFANNMREAF---------------------------------------------------------
    MUCL9792_g2802.t1               ---L-QPIIGW----------DFMNN-DMRATLNNVNWGSANCPINNG-----NFESNLGKAAL--------------------------------------------------------
    MUCL9792_g3157.t1               RWGMGAGLIEW----------ERIPG-HLRNTLSQKDWGAAQMAVRDKDGSDWNFGWYLDQSRR------K----------------CSGGLGREPVYCI--------------------
    MUCL9792_g4589.t1               QFG---DLVGW----------FGFPTAELRTALETHNFGSASFALGG------RFTNNLRNSMVEWCDENVPPDSGESAVCHLQHFPHPFDPARDDNGSPGQPPG--GNGCNPTNPGTPG
    MUCL9792_g4589.t2               QFG---DLVGW----------FGFPTAELRTALETHNFGSASFALGG------RFTNNLRNSMVEWCDENVPPDSGESAVCHLQHFPHPFDPARDDNGSPGQPPG--GNGCNPTNPGTPG
    MUCL9792_g4589.t3               ------------------------------------------------------------------------------------------------------------------------
    MUCL9792_g6332.t1               YKS---PLVGW----------LSWPNEGLRDSMLG-AFSGGVGPKLDD-----EFAGKLGEAAGDAVPE--------------------FDPNVDE------------------------
    MUCL9792_g7308.t1               ---E-QPLVAW----------ESLTP-AARAALESTNFGSANVPLKDA-----NFASNLQKAAL--------------------------------------------------------
    MUCL9792_g10611.t1              ---T-YPISDW----------DMLPQ-EAKDALENTSFGSANVPFKEA-----NFDSNLAKAAL--------------------------------------------------------
    PD401_g2361.t1                  ---L-QPIIGW----------DFMNN-DMRTTLNDVNWGSANCPINNW-----NFENNLGKAAL--------------------------------------------------------
    PD401_g3373.t1                  ---E-QPLVAW----------ESLTP-AARAALESTDFGSANVPLKDA-----NFASNLQKAAL--------------------------------------------------------
    PD401_g4730.t1                  YKS---PLVGW----------LSWPNEGLRDSMLG-AFSGGVGPKLDD-----EFAGKLGEAAGDAVPE--------------------FDPNVDE------------------------
    PD401_g7339.t1                  ---T-YPISDW----------DMLPQ-EAKDALENTSFGSANVPFKDA-----NFDSNLAKAAL--------------------------------------------------------
    PD401_g10483.t1                 ---T-YWLYDW----------DAMAP-IVKSALNQGKFGKANCPINDN-----NFANNMREAF---------------------------------------------------------
    PD593_g749.t1                   QFG---DLVGW----------FGFPTAELRTALETHNFGSASFALGG------RFTNNLRNSMVEWCDENVPPDSGESAVCHLQHFPHPFDPARDDNGSPGQPPG--GNGCNPTNPGTPG
    PD593_g2541.t1                  ---L-QPIIGW----------DFMNN-DMRATLNNVNWGSANCPINDG-----NFGSNLDKAAL--------------------------------------------------------
    PD593_g2728.t1                  YKS---PLVGW----------LSWPNEGLRDSMLG-AFSGGVGPKLDD-----EFAGKLGEAAGDAVPE--------------------FDPNVDE------------------------
    PD593_g7030.t1                  ---E-QPLVAW----------ESLTP-AARAALESTNFGSANVPLKDA-----NFASNLQKAAL--------------------------------------------------------
    PD593_g8432.t1                  ---T-YPISDW----------DMLPQ-EAKDALENTSFGSANVPFKEA-----NFDSNLAKAAL--------------------------------------------------------
    PD593_g9596.t1                  ---T-YWLYDW----------DAMAP-IVKSALNQGKFGKANCPINDN-----NFANNMREAF---------------------------------------------------------
    PD618_g114.t1                   ---L-QPIIGW----------DFMNN-DMRKTLNDVNWGSANCPINNG-----NFGSNLDKAAL--------------------------------------------------------
    PD618_g3974.t1                  YKS---PLVGW----------LSWPNEGLRDSMLG-AFSGGVGPKLDD-----EFAGKLGEAAGDAVPE--------------------FDPNVDE------------------------
    PD618_g5755.t1                  ---E-QPLVAW----------ESLTP-AARAALESTDFGSANVPLKDA-----NFASNLQKAAL--------------------------------------------------------
    PD618_g9213.t1                  ---T-YPISDW----------DMLPQ-EAKDALENTSFGSANVPFKDA-----NFDSNLAKAAL--------------------------------------------------------
    PD618_g9942.t1                  ---T-YWLYDW----------DAMAP-IVKSALNQGKFGKANCPINDN-----NFANNMREAF---------------------------------------------------------
    PD659_g6753.t1                  ---L-QPIIGW----------DFMNN-DMRTTLNDVNWGSANCPINNW-----NFENNLGKAAL--------------------------------------------------------
    PD659_g7370.t1                  ---T-YPISDW----------DMLPQ-EAKDALENTSFGSANVPFKDA-----NFDSNLAKAAL--------------------------------------------------------
    PD659_g10785.t1                 ---T-YWLYDW----------DAMAP-IVKSALNQGKFGKANCPINDN-----NFANNMREAF---------------------------------------------------------
    PD659_g2262.t1                  YKS---PLVGW----------LSWPNEGLRDSMLG-AFSGGVGPKLDD-----EFAGKLGEAAGDAVPE--------------------FDPNVDE------------------------
    PD659_g5819.t1                  ---E-QPLVAW----------ESLTP-AARAALESTDFGSANVPLKDA-----NFASNLQKAAL--------------------------------------------------------
    PD660_g1229.t1                  ---E-QPLVAW----------ESLTP-AARAALESTDFGSANVPLKDA-----NFASNLQKAAL--------------------------------------------------------
    PD660_g3973.t1                  YKS---PLVGW----------LSWPNEGLRDSMLG-AFSGGVGPKLDD-----EFAGKLGEAAGDAVPE--------------------FDPNVDE------------------------
    PD660_g6305.t1                  ---T-YWLYDW----------DAMAP-IVKSALNQGKFGKANCPINDN-----NFANNMREAF---------------------------------------------------------
    PD660_g7826.t1                  ---T-YPISDW----------DMLPQ-EAKDALENTSFGSANVPFKDA-----NFDSNLAKAAL--------------------------------------------------------
    PD660_g9669.t1                  ---L-QPIIGW----------DFMNN-DMRKTLNDVNWGSANCPINNG-----NFGSNLDKAAL--------------------------------------------------------
    PD660_g10032.t1                 RWGMGAGLIEW----------ERIPG-HLRTTLSQKDWGTAQMAVRDKDGSDWNFGWYLDQSRR------K----------------CSGGLGREPVYCI--------------------
    PD670_g4670.t1                  YKS---PLVGW----------LSWPNEGLRNSMLS-AWSGGVGPKLDD-----EFAGKLGEAAGDAVPE--------------------FDPNVDE------------------------
    PD670_g5219.t1                  ---E-QPLIAW----------ESLTA-AARTALENTNFGSANVPFKDA-----NFASNLQKAAL--------------------------------------------------------
    PD670_g7258.t1                  ---T-YPISDW----------DSLPQ-PARDALENTSFGSANVPFKDA-----NFDSNLAKAAL--------------------------------------------------------
    PD670_g8696.t1                  ITG---PLIGY----------FGWDTVEQRDRMFTHDWQAAQLAIKNE-----NFAENIRKARPNGL---------------------AFDENRDDDGT---------------------
    PD670_g10480.t1                 ---S-QPLINW----------SQLPT-AAKKALQKDLFGKAIVPFQDK-----NINSNIDASWSSSYK--------------------P-------------------------------
    PD670_g10529.t1                 ---T-YWLYDW----------DAMAP-IVKSALNQGDFKSANCPFNDN-----NFANNMRKAF---------------------------------------------------------
    PD670_g11082.t1                 ---L-QPIIGW----------DFMND-NMRSTLNNVNWGSANCPINNN-----NFGSTLGKAAL--------------------------------------------------------
    PD670_g2580.t1                  RWGVGAGLIEW----------ERMPA-NLRTTLSQKNWGTAEMAVRDKDGSDWNFGWYINESRW------H----------------CPW-----ETWCLG-------------------
    PD670_g3093.t1                  QFG---DLVGW----------FGFPTTELRTALETHNFGSASFALGG------SFTNNLRNSMVEWCDENVPPDSGESAVCHLQHFPHPFDPARDDNGSPGQPPG--GNGCNPTN---PG
    PD683_g248.t1                   IRG---GLISM----------LLMPS-DWQEKFRSQDWGSAHMAWANED----DFTGHLVKSMPQEARDE------------------GFDCAYDENPA---------------------
    PD683_g3766.t1                  ITG---PLLGY----------FGWDTVEQRDRMLTHNWEAGAIAIKNE-----NFAENIRKARPAGL---------------------VFDENIDDEGT---------------------
    PD683_g3895.t1                  ---T-YWIWDW----------DRMDT-TVQGALNRADFGSANCPFNNN-----NFERNMRAAF---------------------------------------------------------
    PD683_g4100.t1                  ---E-QPLVAW----------ESLTP-AARAALQNTNFGSANVPFKDG-----NFASNLQKAAL--------------------------------------------------------
    PD683_g5003.t1                  QIG---DLVGW----------FGFPTTELRSALETHYFGSASFALGG------SFTTNLKNSLTAWCDDKFPPDGGDLAVCKMQHFPHPFDPARDDNGSPGQPPGGNGNGCNPTN---PG
    PD683_g5003.t2                  QIG---DLVGW----------FGFPTTELRSALETHYFGSASFALGG------SFTTNLKNSLTAWCDDKFPPDGGDLAVCKMQHFPHPFDPARDDNGSPGQPPGGNGNGCNPTN---PG
    PD683_g5713.t1                  ---T-YPISDW----------DAMPQ-ATRDALETTDFGSANVPFKDA-----NFDSNLAKAAL--------------------------------------------------------
    PD683_g7092.t1                  YKS---PLVGW----------LSWPNEGLRQTMLG-AFSGGVGPKLDD-----EFAGKLGEAAGDAVPE--------------------FDPNVDE------------------------
    PD683_g8130.t1                  RWGVGAGLIEW----------ERMPD-NLRKTLSAKNWGAAEMAVRDKDGSDWNFAWYINESQY------F----------------C-W-----ETYCPG-------------------
    PD736_g919.t1                   ---E-QPLVAW----------ESLTP-AARAALESTDFGSANVPLKDA-----NFASNLQKAAL--------------------------------------------------------
    PD736_g8670.t1                  ---T-YWLYDW----------DAMAP-IVKSALNQGKFGKANCPINDN-----NFANNMREAF---------------------------------------------------------
    PD736_g9411.t1                  ---T-YPISDW----------DTLPQ-EAKDALENTSFGSANVPFKDA-----NFDSNLAKAAL--------------------------------------------------------
    PD736_g10271.t1                 YKS---PLVGW----------LSWPNEGLRDSMLG-AFSGGVGPKLDD-----EFAGKLGEAAGDAVPE--------------------FDPNVDE------------------------
    PD736_g10840.t1                 QLG---DLVGW----------FGFPTTEIRTALETHNFGSASFALGG------SFTNNLRNSMLVWCDENVPRETGEYAVCALQHFPHPFDPARDENGSPGQPSG--GNGCNPTT---PG
    PD736_g10939.t1                 ---L-QPIIGW----------SFMND-DMRTTLNDVNWGSANCPINNW-----NFENNLGKAAL--------------------------------------------------------
    PD739_g34.t1                    QLG---DLVGW----------FGFPTTEIRTALETHNFGSASFALGG------SFTNNLRNSMLVWCDENVPRETGEYAVCALQHFPHPFDPARDENGSPGQPSG--GNGCNPTT---PG
    PD739_g131.t1                   ---L-QPIIGW----------DFMND-DMRTTLNDVNWGSANCPINNW-----NFENNLGKAAL--------------------------------------------------------
    PD739_g4443.t1                  YKS---PLVGW----------LSWPNEGLRDSMLG-AFSGGVGPKLDD-----EFAGKLGEAAGDAVPE--------------------FDPNVDE------------------------
    PD739_g7436.t1                  ---E-QPLVAW----------ESLTP-AARAALESTDFGSANVPLKDA-----NFASNLQKAAL--------------------------------------------------------
    PD739_g8512.t1                  ---T-YWLYDW----------DAMAP-IVKSALNQGKFGKANCPINDN-----NFANNMREAF---------------------------------------------------------
    PD739_g10815.t1                 ---T-YPISDW----------DTLPQ-EAKDALENTSFGSANVPFKDA-----NFDSNLAKAAL--------------------------------------------------------
    PD747_g20.t1                    QFG---DLVGW----------FGFPTTELRTALETHNFGSASFALGG------SFTNNLRNSMVEWCDENVPPDSGESAVCHLQHFPHPFDPARDDNGSPGQPPG--GNGCNPTN---PG
    PD747_g277.t1                   ---L-QPIIGW----------DFMND-NMRSTLNNVNWGSANCPINNN-----NFGSTLGKAAL--------------------------------------------------------
    PD747_g4174.t1                  YKS---PLVGW----------LSWPNEGLRNSMLS-AWSGGVGPKLDD-----EFAGKLGEAAGDAVPE--------------------FDPNVDE------------------------
    PD747_g5167.t1                  ---E-QPLIAW----------ESLTA-AARTALENTNFGSANVPFKDA-----NFASNLQKAAL--------------------------------------------------------
    PD747_g9207.t1                  RWGVGAGLIEW----------ERMPA-NLRTALSQKNWGTAEMAVRDKDGSDWNFGWYINESRW------H----------------CPW-----ETWCLG-------------------
    PD747_g9751.t1                  ---T-YPISDW----------DSLPQ-PARDALENTSFGSANVPFKDA-----NFDSNLAKAAL--------------------------------------------------------
    PD747_g10403.t1                 ITG---PLIGY----------FGWDTVEQRDRMFTHDWQAAQLAIKNE-----NFAENIRKARPNGL---------------------AFDENRDDDGT---------------------
    PD747_g10498.t1                 ---S-QPLINW----------SQLPT-AAKKALQKDLFGKAIVPFQDK-----NINSNIDASWSSSYK--------------------P-------------------------------
    PD747_g10549.t1                 ---T-YWLYDW----------DAMAP-IVKSALNQGDFKSANCPFNDN-----NFANNMRKAF---------------------------------------------------------
    T2_g2030.t1                     IRG---GLISM----------LLMPS-DWQEKFRSQDWGSAHMAWANED----DFTGHLVKSMPQEARDE------------------GFDCAYDENPA---------------------
    T2_g2030.t2                     IRG---GLISM----------LLMPS-DWQEKFRSQDWGSAHMAWANED----DFTGHLVKSMPQEARDE------------------GFDCAYDENPA---------------------
    T2_g3751.t1                     QLG---DLVGW----------FGFPTTELRSALETHHFGSASFALGG------SFTTNLKNSLTAWCDDKFPPDGGDLAVCKMQHFPHPFDPARDDNGSPGQPPGGNGNGCNPTN---PG
    T2_g3751.t2                     QLG---DLVGW----------FGFPTTELRSALETHHFGSASFALGG------SFTTNLKNSLTAWCDDKFPPDGGDLAVCKMQHFPHPFDPARDDNGSPGQPPGGNGNGCNPTN---PG
    T2_g4661.t1                     ---E-QPLVAW----------ESLTP-AARAALQNTNFGSANVPFKDG-----NFASNLQKAAL--------------------------------------------------------
    T2_g4868.t1                     ---T-YWIWDW----------DRMDS-TVQGALNRADFNRANCPFNNN-----NFERNMRAAF---------------------------------------------------------
    T2_g5004.t1                     ITG---PLLGY----------FGWDTVEQRDRMLTHNWEAGSIAIKNE-----NFAENIRKARPAGL---------------------VFDENIDDEGT---------------------
    T2_g5801.t1                     ---T-YPISDW----------DAMPQ-ATRDALETTNFGSANVPFKDA-----NFDSNLAKAAL--------------------------------------------------------
    T2_g7120.t1                     RWGVGAGLIEW----------ERMPD-NLRKTLSAKNWGAAEMAVRDKDGSDWNFAWYINESQY------F----------------C-W-----ETYCPG-------------------
    T2_g7997.t1                     YKS---PLVGW----------LSWPNEGLRQTMLG-AFSGGVGPKLDD-----EFAGKLGEAAGDAVPE--------------------FDPNVDE------------------------
    VSO1_g539.t1                    ---E-QPLVAW----------ESLTP-AARAALESTNFGSANVPLKDA-----NFASNLQKAAL--------------------------------------------------------
    VSO1_g1387.t1                   ---L-QPIIGW----------DFMNN-DMRKTLNDVNWGSANCPINNG-----NFGSNLDKAAL--------------------------------------------------------
    VSO1_g1731.t1                   ---T-YWLYDW----------DAMAP-IVKSALNQGKFGKANCPINDN-----NFANNMREAF---------------------------------------------------------
    VSO1_g1937.t1                   RWGMGAGLIEW----------ERIPG-HLRTTLSQKDWGTAQMAVRDKDGSDWNFGWYLDQSRR------K----------------CSGGLGREPVYCI--------------------
    VSO1_g4972.t1                   YKS---PLVGW----------LSWPNEGLRDSMLG-AFSGGVGPKLDD-----EFAGKLGEAAGDAVPE--------------------FDPNVDE------------------------
    VSO1_g5184.t1                   ---T-YPISDW----------DMLPQ-EAKDALENTSFGSANVPFKDA-----NFDSNLAKAAL--------------------------------------------------------
    VL20_g7800.t1                   IRG---GLVSM----------LLMPS-DWQDKFRSHDWESAHMAWANED----DFTGHLVKSMPQEARDD------------------GFDCAYDENPA---------------------
    VL20_g9415.t1                   YKS---PLVGW----------LSWPNEGLRQTMFG-AFSGGVGPKLDD-----EFAGKLGEAAGDAVPE--------------------FDPNVDE------------------------
    VL20_g11025.t1                  ---T-YWIWDW----------DRMDT-TVQGALNRADFGSANCPFNNN-----NFERNMRAAF---------------------------------------------------------
    VL20_g11172.t1                  IRG---GLISM----------LLMPS-DWQEKFRSQNWGSAHMAWANEE----DFTGHLVKSMPQEARDD------------------GFDCAYDENPA---------------------
    VL20_g12965.t1                  RWGVGAGLIEW----------ERMPD-NLRKTLSAKNWGAAEMAVRDKDGSDWNFAWYINESQY------F----------------C-W-----ETYCPG-------------------
    VL20_g13837.t1                  ---E-QPLVAW----------ESLTP-AARAALQNTDFGSANVPLKDG-----NFASNLQKAAL--------------------------------------------------------
    VL20_g14042.t1                  ---T-YWIWDW----------DRMDS-TVQGALNRADFGSANCPFNNN-----NFERNMRAAF---------------------------------------------------------
    VL20_g14171.t1                  ITG---PLLGY----------FGWDTVEQRDRMLTHNWEAGSIAIKNE-----NFAENIRKARPAGL---------------------VFDENFDDEGT---------------------
    VL20_g14284.t1                  YKS---PLVGW----------LSWPNEGLRQTMLG-AFSGGVGPKLDD-----EFAGKLGEAAGDAVPE--------------------FDPNVDE------------------------
    VL20_g17450.t1                  ---T-YPISDW----------DAMPQ-AARDALETTDFGSANVPFKDA-----NFDSNLAKAAL--------------------------------------------------------
    VL20_g18857.t1                  ---T-YPISDW----------DAMPQ-AARDALETTDFGSANVPFKDA-----NFDSNLAKAAL--------------------------------------------------------
    VL20_g4752.t1                   ---D-QPLVAW----------ESLTA-AARAALQNTNFGSANVPFKDG-----NFASNLQKAAL--------------------------------------------------------
    VL20_g5938.t1                   RWGVGAGLIEW----------ERMPD-NLRKTLSAKNWGAAEMAVRDKDGSDWNFAWYINESQY------F----------------C-W-----ETYCPG-------------------
    VL20_g5938.t2                   RWGVGAGLIEW----------ERMPD-NLRKTLSAKNWGAAEMAVRDKDGSDWNFAWYINESQY------F----------------C-W-----ETYCPG-------------------
    VL20_g6061.t1                   ITG---PLLGY----------FGWETVEQRDRMLTHNWEAGSIAIRNE-----NFAENIRKARPAGL---------------------VFDENIDDEGT---------------------
    VL20_g18964.t1                  RWGVGAGLIEW----------ERMPD-NLRKTLSAKNWGAAEMAVRDKDGSDWNFAWYINESQY------F----------------C-W-----ETYCPG-------------------
    VL20_g18964.t2                  RWGVGAGLIEW----------ERMPD-NLRKTLSAKNWGAAEMAVRDKDGSDWNFAWYINESQY------F----------------C-W-----ETYCPG-------------------
    VLB2_g585.t1                    IRG---GLVSM----------LLMPS-DWQDKFRSHDWESAHMAWANED----DFTGHLVKSMPQEARDD------------------GFDCAYDENPA---------------------
    VLB2_g1761.t1                   ---T-YWIWDW----------DRMDT-TVQGALNRADFGSANCPFNNN-----NFERNMRAAF---------------------------------------------------------
    VLB2_g2033.t1                   IRG---GLISM----------LLMPS-DWQEKFRSQNWGSAHMAWANEE----DFTGHLVKSMPQEARDD------------------GFDCAYDENPA---------------------
    VLB2_g12050.t1                  ---T-YPISDW----------DAMPQ-AARDALETTDFGSANVPFKDA-----NFDSNLAKAAL--------------------------------------------------------
    VLB2_g12617.t1                  RWGVGAGLIEW----------ERMPD-NLRKTLSAKNWGAAEMAVRDKDGSDWNFAWYINESQY------F----------------C-W-----ETYCPG-------------------
    VLB2_g13499.t1                  ---E-QPLVAW----------ESLTP-AARAALQNTDFGSANVPLKDG-----NFASNLQKAAL--------------------------------------------------------
    VLB2_g13703.t1                  ---T-YWIWDW----------DRMDS-TVQGALNRADFGSANCPFNNN-----NFERNMRAAF---------------------------------------------------------
    VLB2_g13833.t1                  ITG---PLLGY----------FGWDTVEQRDRMLTHNWEAGSIAIKNE-----NFAENIRKARPAGL---------------------VFDENFDDEGT---------------------
    VLB2_g14318.t1                  YKS---PLVGW----------LSWPNEGLRQTMLG-AFSGGVGPKLDD-----EFAGKLGEAAGDAVPE--------------------FDPNVDE------------------------
    VLB2_g15187.t1                  RWGVGAGLIEW----------ERMPD-NLRKTLSAKNWGAAEMAVRDKDGSDWNFAWYINESQY------F----------------C-W-----ETYCPG-------------------
    VLB2_g15187.t2                  RWGVGAGLIEW----------ERMPD-NLRKTLSAKNWGAAEMAVRDKDGSDWNFAWYINESQY------F----------------C-W-----ETYCPG-------------------
    VLB2_g16101.t1                  ---D-QPLVAW----------ESLTA-AARAALQNTNFGSANVPFKDG-----NFASNLQKAAL--------------------------------------------------------
    VLB2_g16543.t1                  YKS---PLVGW----------LSWPNEGLRQTMFG-AFSGGVGPKLDD-----EFAGKLGEAAGDAVPE--------------------FDPNVDE------------------------
    VLB2_g19098.t1                  QLG---DLVGW----------FGFPTTELRSALETHHFGSASFALGG------SFTTNLKNSLTAWCDDKFPPDGGDLAVCKMQHFPHPFDPARDDNGSPGQPPG--GNGCNPTTPGTPG
    VLB2_g5814.t1                   ---T-YPISDW----------DAMPQ-AARDALETTDFGSANVPFKDA-----NFDSNLAKAAL--------------------------------------------------------
    VLB2_g6534.t1                   ITG---PLLGY----------FGWETVEQRDRMLTHNWEAGSIAIRNE-----NFAENIRKARPAGL---------------------VFDENIDDEGT---------------------
    PD589_g51.t1                    RWGVGAGLIEW----------ERMPD-NLRKTLWAKNWGAAEMANREKHGSDWNFAWYINESQY------F----------------C-W-----ETYCPG-------------------
    PD589_g856.t1                   ---T-YWIWDW----------AAMDS-TVQGALNHADFDSVNCPFNNN-----NFENNMRAAF---------------------------------------------------------
    PD589_g7665.t1                  YKS---PLVGW----------LSWPNEGLRQTMLG-AFSGGVGPKLDD-----EFAGKLGEAAGDAVPE--------------------FDPNVDE------------------------
    PD589_g13197.t1                 IRG---GLVSM----------LLMPS-DWQDKFRSHGWGSAHMAWANED----DFTGHLVKSMPQEARDD------------------GFDCAYDENPA---------------------
    PD589_g13425.t1                 ---T-YPISDW----------DSMPQ-AARDALETTDFGSANVPFKDA-----NFDSNLAKAAL--------------------------------------------------------
    PD589_g14403.t1                 ITG---PLLGY----------FGWETVEQRDRMLTHNWEAGSIAIRNE-----NFAENIRKARPAGL---------------------VFDENIDDEGT---------------------
    PD589_g14539.t1                 ITG---PLLGY----------FGWDTVEQRDRMLTHNWEAGSIAIKNE-----NFAENIRKARPAGL---------------------VFDENFDDEGT---------------------
    PD589_g14661.t1                 ---T-YWIWDW----------DRMDT-TVQGALNRADFGSANCPFNNN-----NFERNMRAAF---------------------------------------------------------
    PD589_g15310.t1                 ---A-YPISDW----------DVMPQ-AARDALETTDFGSANVPFKDA-----NFDSNLAKAAL--------------------------------------------------------
    PD589_g16171.t1                 RWGVGAGLIEW----------ERMPD-NLRKTLSAKNWGAAEMAVRDKDGSDWNFAWYINESQY------F----------------C-W-----ETYCPG-------------------
    PD589_g16171.t2                 RWGVGAGLIEW----------ERMPD-NLRKTLSAKNWGAAEMAVRDKDGSDWNFAWYINESQY------F----------------C-W-----ETYCPG-------------------
    PD589_g17659.t1                 YKS---PLVGW----------LSWPNEGLRQTMLG-AFSGGVGPKLDD-----EFAGKLGEAAGDAVPE--------------------FDPNVDE------------------------
    PD589_g18192.t1                 ---E-QPLVAW----------ESLTP-AARAALQNTDFGSANVPLKDG-----NFASNLQKAAL--------------------------------------------------------
    PD589_g3050.t1                  ---T-YWIWDW----------DRMDT-TVQGALNRADFGSANCPFNNN-----NFERNMRAAF---------------------------------------------------------
    PD589_g6680.t1                  IRG---GLISM----------LLMPS-DWQEKFRSQNWGSAHMAWANEE----DFTGHLVKSMPQEARDD------------------GFDCAYDENPA---------------------
    PD589_g6775.t1                  ---D-QPLVAW----------ESLTA-AARAALQNTNFGSANVPFKDG-----NFASNLQKAAL--------------------------------------------------------
    Vt305_g1664.t1                  ---T-YWLYDW----------DAMAP-IVKSALNQGKFGKANCPINDN-----NFANNMREAF---------------------------------------------------------
    Vt305_g2624.t1                  YKS---PLVGW----------LSWPNEGLRDSMLG-AFSGGVGPKLDD-----EFAGKLGEAAGDAVPE--------------------FDPNVDE------------------------
    Vt305_g3295.t1                  ---T-YPISDW----------DMLPQ-EAKDALENTSFGSANVPFKDA-----NFDSNLAKAAL--------------------------------------------------------
    Vt305_g4616.t1                  ---L-QPIIGW----------DFMNN-DMRKTLNDVNWGSANCPINNG-----NFGSNLDKAAL--------------------------------------------------------
    Vt305_g7985.t1                  ---E-QPLVAW----------ESLTP-AARAALESTDFGSANVPLKDA-----NFASNLQKAAL--------------------------------------------------------
    WCS072_g2037.t1                 IRG---GLISM----------LLMPS-DWQEKFRSQDWGSAHMAWANED----DFTGHLVKSMPQEARDE------------------GFDCAYDENPA---------------------
    WCS072_g2037.t2                 IRG---GLISM----------LLMPS-DWQEKFRSQDWGSAHMAWANED----DFTGHLVKSMPQEARDE------------------GFDCAYDENPA---------------------
    WCS072_g3872.t1                 ITG---PLLGY----------FGWDTVEQRDRMLTHNWEAGSIAIKNE-----NFAENIRKARPAGL---------------------VFDENIDDEGT---------------------
    WCS072_g4007.t1                 ---T-YWIWDW----------DRMDS-TVQGALNRADFNRANCPFNNN-----NFERNMRAAF---------------------------------------------------------
    WCS072_g4219.t1                 ---E-QPLVAW----------ESLTP-AARAALQNTNFGSANVPFKDG-----NFASNLQKAAL--------------------------------------------------------
    WCS072_g5132.t1                 QLG---DLVGW----------FGFPTTELRSALETHHFGSASFALGG------SFTTNLKNSLTAWCDDKFPPDGGDLAVCKMQHFPHPFDPARDDNGSPGQPPGGNGNGCNPTN---PG
    WCS072_g5132.t2                 QLG---DLVGW----------FGFPTTELRSALETHHFGSASFALGG------SFTTNLKNSLTAWCDDKFPPDGGDLAVCKMQHFPHPFDPARDDNGSPGQPPGGNGNGCNPTN---PG
    WCS072_g5615.t1                 ---T-YPISDW----------DAMPQ-ATRDALETTNFGSANVPFKDA-----NFDSNLAKAAL--------------------------------------------------------
    WCS072_g6396.t1                 YKS---PLVGW----------LSWPNEGLRQTMLG-AFSGGVGPKLDD-----EFAGKLGEAAGDAVPE--------------------FDPNVDE------------------------
    WCS072_g8181.t1                 RWGVGAGLIEW----------ERMPD-NLRKTLSAKNWGAAEMAVRDKDGSDWNFAWYINESQY------F----------------C-W-----ETYCPG-------------------
    VDAG_JR2_Chr1g22850a-00001      IRG---GLVSM----------LLMPS-DWQDKFRSHGWGSAHMAWANED----DFTGHLVKSMPQEARDD------------------GFDCAYDENPA---------------------
    VDAG_JR2_Chr2g05460a-00001      ---E-QPLVAW----------ESLTP-AARAALQNTDFGSANVPLKDG-----NFASNLQKAAL--------------------------------------------------------
    VDAG_JR2_Chr6g08770a-00001      ---T-YWIWDW----------DRMDT-TVQGALNRADFGSANCPFNNN-----NFERNMRAAF---------------------------------------------------------
    VDAG_JR2_Chr6g10260a-00001      ITG---PLLGY----------FGWDTVEQRDRMLTHNWEAGSIAIKNE-----NFAENIRKARPAGL---------------------VFDENFDDEGT---------------------
    VDAG_JR2_Chr3g00800a-00001      YKS---PLVGW----------LSWPNEGLRQTMLG-AFSGGVGPKLDD-----EFAGKLGEAAGDAVPE--------------------FDPNVDE------------------------
    VDAG_JR2_Chr4g05950a-00001      ---T-YPISDW----------DAMPQ-AARDALETTDFGSANVPFKDA-----NFDSNLAKAAL--------------------------------------------------------
    VDAG_JR2_Chr8g10650a-00001      RWGVGAGLIEW----------ERMPD-NLRKTLSAKNWGAAEMAVRDKDGSDWNFAWYINESQY------F----------------C-W-----ETYCPG-------------------
    JR2_g2720.t1                    QLG---DLV---------------------------------------------------------------------------------------------------------------

    Selected Cols:                                                                                                                                          

    Gaps Scores:                                                                                                                                            
    Similarity Scores:                                                                                                                                      

                                           490       500       510       520       530       540       550       560       570       580       590       600
                                    =========+=========+=========+=========+=========+=========+=========+=========+=========+=========+=========+=========+
    12008_g358.t1                   ------------------------------------------------------------------------------------------------------------------------
    12008_g4461.t1                  ------------------------------------------------------------------------------------------------------------------------
    12008_g7340.t1                  ----------------FLAPEF--------------------------------------------------------------------------------------------------
    12008_g7652.t1                  ------------------------------------------------------------------------------------------------------------------------
    12008_g7834.t1                  ------------------------------------------------------------------------------------------------------------------------
    12008_g8465.t1                  ------------------LKGFPMDW----------------------------------------------------------------------------------------------
    12008_g8848.t1                  ------------------------------------------------------------------------------------------------------------------------
    85S_g58.t1                      ----------------FLAPEF--------------------------------------------------------------------------------------------------
    85S_g9168.t1                    ------------------------------------------------------------------------------------------------------------------------
    85S_g9863.t1                    ------------------------------------------------------------------------------------------------------------------------
    85S_g7040.t1                    ------------------LKGFPMDW----------------------------------------------------------------------------------------------
    85S_g3154.t1                    ------------------------------------------------------------------------------------------------------------------------
    85S_g4608.t1                    ------------------------------------------------------------------------------------------------------------------------
    85S_g4744.t1                    ------------------------------------------------------------------------------------------------------------------------
    CBS385.49_g66.t1                ------------------------------------------------------------------------------------------------------------------------
    CBS385.49_g189.t1               ------------------------------------------------------------------------------------------------------------------------
    CBS385.49_g400.t1               ------------------------------------------------------------------------------------------------------------------------
    CBS385.49_g400.t2               ------------------------------------------------------------------------------------------------------------------------
    CBS385.49_g1381.t1              ----------------FLAPEF--------------------------------------------------------------------------------------------------
    CBS385.49_g5607.t1              ------------------------------------------------------------------------------------------------------------------------
    CBS385.49_g7723.t1              ------------------------------------------------------------------------------------------------------------------------
    CBS385.49_g8721.t1              ------------------LKGFPMDW----------------------------------------------------------------------------------------------
    CQ2_g254.t1                     ------------------LKGFPMDW----------------------------------------------------------------------------------------------
    CQ2_g2332.t3                    TPGPAFGVINAMIVGDSITQGFEGDYTWRFRLAEWFTSESITPNFVGPWTGTHTNAEPSDPKPPRLIGTGSQPVPPDERRTGGAYANGVSFK----AIIGLCGAA---------------
    CQ2_g2332.t4                    ------------------------------------------------------------------------------------------------------------------------
    CQ2_g3243.t1                    ------------------------------------------------------------------------------------------------------------------------
    CQ2_g3454.t1                    ------------------------------------------------------------------------------------------------------------------------
    CQ2_g3584.t1                    ------------------------------------------------------------------------------------------------------------------------
    CQ2_g4742.t1                    ------------------------------------------------------------------------------------------------------------------------
    CQ2_g7512.t1                    ------------------------------------------------------------------------------------------------------------------------
    CQ2_g8820.t1                    ------------------------------------------------------------------------------------------------------------------------
    DAR82592_g1639.t1               ------------------LKGFPMDW----------------------------------------------------------------------------------------------
    DAR82592_g8837.t1               ----------------FLAPEF--------------------------------------------------------------------------------------------------
    DAR82592_g4435.t1               ------------------------------------------------------------------------------------------------------------------------
    DAR82592_g4647.t1               ------------------------------------------------------------------------------------------------------------------------
    DAR82592_g7268.t1               ------------------------------------------------------------------------------------------------------------------------
    DAR82592_g4307.t1               ------------------------------------------------------------------------------------------------------------------------
    DAR82592_g3508.t1               ------------------------------------------------------------------------------------------------------------------------
    DAR82592_g62.t1                 ------------------------------------------------------------------------------------------------------------------------
    DAR83138_g1199.t1               ------------------LKGFPMDW----------------------------------------------------------------------------------------------
    DAR83138_g60.t1                 ------------------------------------------------------------------------------------------------------------------------
    DAR83138_g7641.t1               ------------------------------------------------------------------------------------------------------------------------
    DAR83138_g9085.t1               ----------------FLAPEF--------------------------------------------------------------------------------------------------
    DAR83138_g3565.t3               TPGPAFGVINAMIVGDSITQGFEGDYTWRFRLAEWFTSESITPNFVGPWTGTHTNAEPSDPKPPRLIGTGSQPVPPDERRTGGAYANGVSFK----AIIGLCGAA---------------
    DAR83138_g4173.t1               ------------------------------------------------------------------------------------------------------------------------
    DAR83138_g4314.t1               ------------------------------------------------------------------------------------------------------------------------
    DAR83138_g4535.t1               ------------------------------------------------------------------------------------------------------------------------
    DAR83143_g1427.t1               ------------------LKGFPMDW----------------------------------------------------------------------------------------------
    DAR83143_g4194.t1               ------------------------------------------------------------------------------------------------------------------------
    DAR83143_g4335.t1               ------------------------------------------------------------------------------------------------------------------------
    DAR83143_g4545.t1               ------------------------------------------------------------------------------------------------------------------------
    DAR83143_g7287.t1               ------------------------------------------------------------------------------------------------------------------------
    DAR83143_g3521.t1               ------------------------------------------------------------------------------------------------------------------------
    DAR83143_g8715.t1               ----------------FLAPEF--------------------------------------------------------------------------------------------------
    DAR83143_g61.t1                 ------------------------------------------------------------------------------------------------------------------------
    DAR83175_g3551.t1               ------------------------------------------------------------------------------------------------------------------------
    DAR83175_g4514.t1               ------------------------------------------------------------------------------------------------------------------------
    DAR83175_g4731.t1               ------------------------------------------------------------------------------------------------------------------------
    DAR83175_g7439.t1               ------------------------------------------------------------------------------------------------------------------------
    DAR83175_g2075.t1               ------------------LKGFPMDW----------------------------------------------------------------------------------------------
    DAR83175_g5817.t1               ------------------------------------------------------------------------------------------------------------------------
    DAR83175_g61.t1                 ------------------------------------------------------------------------------------------------------------------------
    DAR83175_g8890.t1               ----------------FLAPEF--------------------------------------------------------------------------------------------------
    DK045_g927.t1                   ------------------------------------------------------------------------------------------------------------------------
    DK045_g1138.t1                  ------------------------------------------------------------------------------------------------------------------------
    DK045_g1272.t1                  ------------------------------------------------------------------------------------------------------------------------
    DK045_g2451.t1                  ------------------------------------------------------------------------------------------------------------------------
    DK045_g3667.t1                  ------------------LKGFPMDW----------------------------------------------------------------------------------------------
    DK045_g5985.t1                  ----------------FLAPEF--------------------------------------------------------------------------------------------------
    DK045_g9032.t1                  ------------------------------------------------------------------------------------------------------------------------
    GF1192_g8987.t1                 ------------------------------------------------------------------------------------------------------------------------
    GF1192_g9126.t1                 ------------------------------------------------------------------------------------------------------------------------
    GF1192_g257.t1                  ------------------LKGFPMDW----------------------------------------------------------------------------------------------
    GF1192_g1728.t1                 ------------------------------------------------------------------------------------------------------------------------
    GF1192_g2840.t1                 ------------------------------------------------------------------------------------------------------------------------
    GF1192_g5025.t1                 ------------------------------------------------------------------------------------------------------------------------
    GF1192_g6069.t1                 ----------------FLAPEF--------------------------------------------------------------------------------------------------
    GF1207_g2093.t1                 ------------------LKGFPMDW----------------------------------------------------------------------------------------------
    GF1207_g4890.t1                 ------------------------------------------------------------------------------------------------------------------------
    GF1207_g5214.t1                 ----------------FLAPEF--------------------------------------------------------------------------------------------------
    GF1207_g6669.t1                 ------------------------------------------------------------------------------------------------------------------------
    GF1207_g6876.t1                 ------------------------------------------------------------------------------------------------------------------------
    GF1207_g6999.t1                 ------------------------------------------------------------------------------------------------------------------------
    GF1207_g7831.t1                 ------------------------------------------------------------------------------------------------------------------------
    GF1300_g9576.t1                 ------------------------------------------------------------------------------------------------------------------------
    GF1300_g9714.t1                 ------------------------------------------------------------------------------------------------------------------------
    GF1300_g259.t1                  ------------------LKGFPMDW----------------------------------------------------------------------------------------------
    GF1300_g1512.t1                 ------------------------------------------------------------------------------------------------------------------------
    GF1300_g3582.t1                 ----------------FLAPEF--------------------------------------------------------------------------------------------------
    GF1300_g3720.t1                 ------------------------------------------------------------------------------------------------------------------------
    GF1300_g6171.t1                 ------------------------------------------------------------------------------------------------------------------------
    Gf-Ca2_g111.t1                  ------------------------------------------------------------------------------------------------------------------------
    Gf-Ca2_g2398.t1                 ------------------LKGFPMDW----------------------------------------------------------------------------------------------
    Gf-Ca2_g3362.t1                 ------------------------------------------------------------------------------------------------------------------------
    Gf-Ca2_g3481.t1                 ------------------------------------------------------------------------------------------------------------------------
    Gf-Ca2_g3687.t1                 ------------------------------------------------------------------------------------------------------------------------
    Gf-Ca2_g4661.t1                 ----------------FLAPEF--------------------------------------------------------------------------------------------------
    Gf-Ca2_g8346.t1                 ------------------------------------------------------------------------------------------------------------------------
    Gf-Cb5_g260.t1                  ------------------LKGFPMDW----------------------------------------------------------------------------------------------
    Gf-Cb5_g3949.t1                 ------------------------------------------------------------------------------------------------------------------------
    Gf-Cb5_g4084.t1                 ------------------------------------------------------------------------------------------------------------------------
    Gf-Cb5_g4605.t1                 ------------------------------------------------------------------------------------------------------------------------
    Gf-Cb5_g6249.t1                 ------------------------------------------------------------------------------------------------------------------------
    Gf-Cb5_g7167.t1                 ------------------------------------------------------------------------------------------------------------------------
    Gf-Cb5_g7934.t1                 ------------------------------------------------------------------------------------------------------------------------
    Gf-Cb5_g8589.t1                 ----------------FLAPEF--------------------------------------------------------------------------------------------------
    HoMCF_g1490.t1                  ---------------------F--------------------------------------------------------------------------------------------------
    HoMCF_g2875.t1                  ------------------------------------------------------------------------------------------------------------------------
    HoMCF_g3274.t1                  ------------------------------------------------------------------------------------------------------------------------
    HoMCF_g4303.t1                  ------------------LKGFPMDW----------------------------------------------------------------------------------------------
    HoMCF_g5967.t1                  ------------------------------------------------------------------------------------------------------------------------
    HoMCF_g6093.t1                  ------------------------------------------------------------------------------------------------------------------------
    HoMCF_g6096.t1                  ------------------------------------------------------------------------------------------------------------------------
    HoMCF_g6549.t1                  ------------------------------------------------------------------------------------------------------------------------
    HoMCF_g10173.t1                 ------------------------------------------------------------------------------------------------------------------------
    HoMCLT_g1190.t1                 ------------------LKGFPMDW----------------------------------------------------------------------------------------------
    HoMCLT_g2195.t1                 ------------------------------------------------------------------------------------------------------------------------
    HoMCLT_g2713.t1                 ----------------FLAPEF--------------------------------------------------------------------------------------------------
    HoMCLT_g3698.t1                 ------------------------------------------------------------------------------------------------------------------------
    HoMCLT_g4981.t1                 ------------------------------------------------------------------------------------------------------------------------
    HoMCLT_g6335.t1                 ------------------------------------------------------------------------------------------------------------------------
    HoMCLT_g6471.t1                 ------------------------------------------------------------------------------------------------------------------------
    I1V_g7945.t1                    ------------------------------------------------------------------------------------------------------------------------
    I1V_g9550.t1                    ------------------------------------------------------------------------------------------------------------------------
    I1V_g9695.t1                    --------------------GFPMDW----------------------------------------------------------------------------------------------
    I1V_g10783.t1                   ------------------------------------------------------------------------------------------------------------------------
    I1V_g12440.t1                   ------------------------------------------------------------------------------------------------------------------------
    I1V_g13776.t1                   ----------------FLAPEF--------------------------------------------------------------------------------------------------
    MPI-CAGE_g5591.t1               ------------------------------------------------------------------------------------------------------------------------
    MPI-CAGE_g8268.t1               ------------------------------------------------------------------------------------------------------------------------
    MPI-CAGE_g8414.t1               ------------------------------------------------------------------------------------------------------------------------
    MPI-CAGE_g9956.t1               ------------------------------------------------------------------------------------------------------------------------
    MPI-CAGE_g1577.t1               ------------------LKGFPMDW----------------------------------------------------------------------------------------------
    MPI-CAGE_g2204.t1               TPGPAFGVINAMIVGDSITQGFEGDYTWRFRLAEWFTSESITPNFVGPWTGTHTNAEPSDPKPPRLIGTGSQPVPPDERRTGGAYANGVSFKSNHWALWGRQAAQVKNEIRSQVSTHSPD
    MPI-CAGE_g10572.t1              ------------------------------------------------------------------------------------------------------------------------
    MPI-CAGE_g3967.t1               ----------------FLAPEF--------------------------------------------------------------------------------------------------
    MPI-CAGE_g4142.t1               ------------------------------------------------------------------------------------------------------------------------
    S011_g7486.t1                   ------------------------------------------------------------------------------------------------------------------------
    S011_g8092.t1                   ------------------------------------------------------------------------------------------------------------------------
    S011_g8657.t1                   ------------------------------------------------------------------------------------------------------------------------
    S011_g8986.t1                   ------------------------------------------------------------------------------------------------------------------------
    S011_g9203.t1                   ------------------------------------------------------------------------------------------------------------------------
    S011_g1207.t1                   ------------------LKGFPMDW----------------------------------------------------------------------------------------------
    S011_g2838.t1                   ------------------------------------------------------------------------------------------------------------------------
    S011_g5817.t1                   ----------------FLAPEF--------------------------------------------------------------------------------------------------
    S023_g1908.t1                   ------------------LKGFPMDW----------------------------------------------------------------------------------------------
    S023_g3148.t1                   ------------------------------------------------------------------------------------------------------------------------
    S023_g4559.t1                   ------------------------------------------------------------------------------------------------------------------------
    S023_g5760.t1                   ------------------------------------------------------------------------------------------------------------------------
    S023_g5904.t1                   ------------------------------------------------------------------------------------------------------------------------
    S023_g7666.t1                   ----------------FLAPEF--------------------------------------------------------------------------------------------------
    S023_g9104.t1                   ------------------------------------------------------------------------------------------------------------------------
    TO22_g2077.t1                   ------------------LKGFPMDW----------------------------------------------------------------------------------------------
    TO22_g2405.t1                   ------------------------------------------------------------------------------------------------------------------------
    TO22_g2541.t1                   ------------------------------------------------------------------------------------------------------------------------
    TO22_g2749.t1                   ------------------------------------------------------------------------------------------------------------------------
    TO22_g3776.t1                   ------------------------------------------------------------------------------------------------------------------------
    TO22_g6379.t1                   ------------------------------------------------------------------------------------------------------------------------
    TO22_g9457.t1                   ----------------FLAPEF--------------------------------------------------------------------------------------------------
    Ud1-4-1_g9889.t1                ----------------FLAPEF--------------------------------------------------------------------------------------------------
    Ud1-4-1_g261.t1                 ------------------LKGFPMDW----------------------------------------------------------------------------------------------
    Ud1-4-1_g2536.t1                ------------------------------------------------------------------------------------------------------------------------
    Ud1-4-1_g2721.t1                ------------------------------------------------------------------------------------------------------------------------
    Ud1-4-1_g2858.t1                ------------------------------------------------------------------------------------------------------------------------
    Ud1-4-1_g3064.t1                ------------------------------------------------------------------------------------------------------------------------
    Ud1-4-1_g3975.t1                ------------------------------------------------------------------------------------------------------------------------
    Ud1-4-1_g4565.t1                ------------------------------------------------------------------------------------------------------------------------
    V13_g3278.t1                    ------------------------------------------------------------------------------------------------------------------------
    V13_g3397.t1                    ------------------------------------------------------------------------------------------------------------------------
    V13_g4590.t1                    ------------------------------------------------------------------------------------------------------------------------
    V13_g5067.t1                    ------------------------------------------------------------------------------------------------------------------------
    V13_g6708.t1                    ----------------RRPHRF--------------------------------------------------------------------------------------------------
    V13_g9344.t1                    ------------------------------------------------------------------------------------------------------------------------
    V13_g9739.t1                    ------------------------------------------------------------------------------------------------------------------------
    V13_g10958.t1                   ------------------LKGFPMDW----------------------------------------------------------------------------------------------
    Vd39_g2112.t1                   TPGPAFGVINAMIVGDSITQGFEGDYTWRFRLAEWFTSESITPNFVGPWTGTHTNAEPSDPKPPRLIGTGSQPVPPDERRTGGAYANGVSFKSNHWALWGRQAAQVKNEIRSQVSTHSPD
    Vd39_g2112.t3                   ------------------------------------------------------------------------------------------------------------------------
    Vd39_g2716.t1                   ------------------LKGFPMDW----------------------------------------------------------------------------------------------
    Vd39_g3506.t1                   ------------------------------------------------------------------------------------------------------------------------
    Vd39_g3907.t1                   ----------------FLAPEF--------------------------------------------------------------------------------------------------
    Vd39_g4379.t1                   ------------------------------------------------------------------------------------------------------------------------
    Vd39_g7329.t1                   ------------------------------------------------------------------------------------------------------------------------
    Vd39_g8964.t1                   ------------------------------------------------------------------------------------------------------------------------
    Vd39_g9298.t1                   ------------------------------------------------------------------------------------------------------------------------
    Vd-653_g1291.t1                 ------------------------------------------------------------------------------------------------------------------------
    Vd-653_g2557.t1                 ------------------LKGFPMDW----------------------------------------------------------------------------------------------
    Vd-653_g3339.t1                 ----------------FLAPEF--------------------------------------------------------------------------------------------------
    Vd-653_g3685.t1                 ------------------------------------------------------------------------------------------------------------------------
    Vd-653_g3686.t1                 ------------------------------------------------------------------------------------------------------------------------
    Vd-653_g5777.t1                 ------------------------------------------------------------------------------------------------------------------------
    Vd-653_g8550.t1                 ------------------------------------------------------------------------------------------------------------------------
    Vd-653_g9566.t1                 ------------------------------------------------------------------------------------------------------------------------
    VD991_g2070.t1                  ------------------LKGFPMDW----------------------------------------------------------------------------------------------
    VD991_g2394.t1                  ------------------------------------------------------------------------------------------------------------------------
    VD991_g2531.t1                  ------------------------------------------------------------------------------------------------------------------------
    VD991_g2742.t1                  ------------------------------------------------------------------------------------------------------------------------
    VD991_g3653.t1                  TPGPAFGVINAMIVGDSITQGFEGDYTWRFRLAEWFTSESITPNFVGPWTGTHTNAEPSDPKPPRLIGTG--PVPPDERRTGGAYANGVSFK----AIIGLCGAA---------------
    VD991_g3653.t2                  TPGPAFGVINAMIVGDSITQGFEGDYTWRFRLAEWFTSESITPNFVGPWTGTHTNAEPSDPKPPRLIGTG--PVPPDERRTGGAYANGVSFK----AIIGLCGAA---------------
    VD991_g3719.t1                  ------------------------------------------------------------------------------------------------------------------------
    VD991_g7479.t1                  ------------------------------------------------------------------------------------------------------------------------
    VD991_g8969.t1                  ----------------FLAPEF--------------------------------------------------------------------------------------------------
    VdB09_g1055.t1                  ------------------------------------------------------------------------------------------------------------------------
    VdB09_g1685.t1                  ----------------FLAPEF--------------------------------------------------------------------------------------------------
    VdB09_g1751.t1                  ------------------------------------------------------------------------------------------------------------------------
    VdB09_g3918.t1                  ------------------------------------------------------------------------------------------------------------------------
    VdB09_g4877.t1                  ------------------------------------------------------------------------------------------------------------------------
    VdB09_g5096.t1                  ------------------------------------------------------------------------------------------------------------------------
    VdB09_g9703.t1                  ------------------------------------------------------------------------------------------------------------------------
    VdB09_g7661.t1                  ------------------LKGFPMDW----------------------------------------------------------------------------------------------
    VdC07_g4126.t1                  ------------------------------------------------------------------------------------------------------------------------
    VdC07_g4261.t1                  ------------------------------------------------------------------------------------------------------------------------
    VdC07_g4482.t1                  ------------------------------------------------------------------------------------------------------------------------
    VdC07_g605.t1                   ------------------------------------------------------------------------------------------------------------------------
    VdC07_g1872.t1                  ----------------FLAPEF--------------------------------------------------------------------------------------------------
    VdC07_g2986.t1                  ------------------------------------------------------------------------------------------------------------------------
    VdC07_g7672.t1                  ------------------LKGFPMDW----------------------------------------------------------------------------------------------
    Vd-H5_g2477.t1                  ------------------------------------------------------------------------------------------------------------------------
    Vd-H5_g5176.t1                  ---------------------F--------------------------------------------------------------------------------------------------
    Vd-H5_g6144.t1                  ------------------------------------------------------------------------------------------------------------------------
    Vd-H5_g7203.t1                  ------------------------------------------------------------------------------------------------------------------------
    Vd-H5_g8423.t1                  ------------------------------------------------------------------------------------------------------------------------
    Vd-H5_g8546.t1                  ------------------------------------------------------------------------------------------------------------------------
    Vd-H5_g9396.t1                  ------------------LKGFPMDW----------------------------------------------------------------------------------------------
    VdLs16_g2071.t1                 ------------------LKGFPMDW----------------------------------------------------------------------------------------------
    VdLs16_g7836.t1                 ------------------------------------------------------------------------------------------------------------------------
    VdLs16_g8455.t1                 ------------------------------------------------------------------------------------------------------------------------
    VdLs16_g5349.t1                 ------------------------------------------------------------------------------------------------------------------------
    VdLs16_g5560.t1                 ------------------------------------------------------------------------------------------------------------------------
    VdLs16_g5699.t1                 ------------------------------------------------------------------------------------------------------------------------
    VdLs16_g6952.t1                 ----------------FLAPEF--------------------------------------------------------------------------------------------------
    VdLs17_g64.t1                   ------------------------------------------------------------------------------------------------------------------------
    VdLs17_g1975.t1                 ------------------LKGFPMDW----------------------------------------------------------------------------------------------
    VdLs17_g3499.t1                 ------------------------------------------------------------------------------------------------------------------------
    VdLs17_g4253.t1                 ------------------------------------------------------------------------------------------------------------------------
    VdLs17_g4391.t1                 ------------------------------------------------------------------------------------------------------------------------
    VdLs17_g5246.t1                 ------------------------------------------------------------------------------------------------------------------------
    VdLs17_g6770.t1                 ------------------------------------------------------------------------------------------------------------------------
    VdLs17_g8273.t1                 ----------------FLAPEF--------------------------------------------------------------------------------------------------
    VdLs17_g8809.t1                 ------------------------------------------------------------------------------------------------------------------------
    Vd-R1_g926.t1                   ------------------------------------------------------------------------------------------------------------------------
    Vd-R1_g3679.t1                  ------------------------------------------------------------------------------------------------------------------------
    Vd-R1_g4718.t1                  ------------------------------------------------------------------------------------------------------------------------
    Vd-R1_g5037.t1                  ----------------FLAPEF--------------------------------------------------------------------------------------------------
    Vd-R1_g8886.t1                  ------------------LKGFPMDW----------------------------------------------------------------------------------------------
    Vd-R1_g9491.t1                  ------------------------------------------------------------------------------------------------------------------------
    Vd-R1_g9617.t1                  ------------------------------------------------------------------------------------------------------------------------
    VT-2A_g1161.t1                  ------------------------------------------------------------------------------------------------------------------------
    VT-2A_g1267.t1                  ----------------FLAPEF--------------------------------------------------------------------------------------------------
    VT-2A_g4726.t1                  ------------------------------------------------------------------------------------------------------------------------
    VT-2A_g6128.t1                  ------------------------------------------------------------------------------------------------------------------------
    VT-2A_g6128.t2                  ------------------------------------------------------------------------------------------------------------------------
    VT-2A_g6341.t1                  ------------------------------------------------------------------------------------------------------------------------
    VT-2A_g6470.t1                  ------------------------------------------------------------------------------------------------------------------------
    VT-2A_g9510.t1                  ------------------LKGFPMDW----------------------------------------------------------------------------------------------
    VT-2A_g9511.t1                  ------------------LKGFPMDW----------------------------------------------------------------------------------------------
    XJ511_g1085.t1                  ------------------LKGFPMDW----------------------------------------------------------------------------------------------
    XJ511_g1518.t1                  ------------------------------------------------------------------------------------------------------------------------
    XJ511_g4592.t1                  ----------------FLAPEF--------------------------------------------------------------------------------------------------
    XJ511_g5794.t1                  ------------------------------------------------------------------------------------------------------------------------
    XJ511_g5932.t1                  ------------------------------------------------------------------------------------------------------------------------
    XJ511_g7291.t1                  ------------------------------------------------------------------------------------------------------------------------
    XJ511_g8237.t1                  ------------------------------------------------------------------------------------------------------------------------
    XJ592_g261.t1                   ------------------LKGFPMDW----------------------------------------------------------------------------------------------
    XJ592_g3433.t1                  ------------------------------------------------------------------------------------------------------------------------
    XJ592_g5005.t1                  ------------------------------------------------------------------------------------------------------------------------
    XJ592_g6487.t1                  ----------------FLAPEF--------------------------------------------------------------------------------------------------
    XJ592_g7860.t1                  ------------------------------------------------------------------------------------------------------------------------
    XJ592_g8068.t1                  ------------------------------------------------------------------------------------------------------------------------
    XJ592_g8206.t1                  ------------------------------------------------------------------------------------------------------------------------
    XJ592_g9637.t1                  TPGPAFGVINAMIVGDSITQGFEGDYTWRFRLAEWFTSESITPNFVGPWTGTHTNAEPSDPKPPRLIGTGSQPVPPDERRTGGAYANGVSFK----AIIGLCGAA---------------
    XJ592_g9637.t2                  TPGPAFGVINAMIVGDSITQGFEGDYTWRFRLAEWFTSESITPNFVGPWTGTHTNAEPSDPKPPRLIGTG--PVPPDERRTGGAYANGVSFK----AIIGLCGAA---------------
    397_g2445.t1                    ------------------------------------------------------------------------------------------------------------------------
    397_g4853.t1                    ------------------------------------------------------------------------------------------------------------------------
    397_g6363.t1                    ----------------WLAPEF--------------------------------------------------------------------------------------------------
    397_g7397.t1                    ------------------------------------------------------------------------------------------------------------------------
    397_g7755.t1                    ------------------LKGFPRNW----------------------------------------------------------------------------------------------
    397_g8350.t1                    ------------------------------------------------------------------------------------------------------------------------
    397_g9733.t1                    ------------------------------------------------------------------------------------------------------------------------
    CBS382.66_g255.t1               ------------------LKGFPMDW----------------------------------------------------------------------------------------------
    CBS382.66_g255.t2               ------------------LKGFPMDW----------------------------------------------------------------------------------------------
    CBS382.66_g4327.t1              ------------------------------------------------------------------------------------------------------------------------
    CBS382.66_g5334.t1              TPGPAVGVINAMIVGDSITQGFEGDYTWRFRLAEWFKSESITPNFVGPWTGTHTNAEPSDPKPPRLIGSG--PVPPDAPRTGGAYANGVSFKSNHWALWGRQAAQVKNEIRGQVSTHSPD
    CBS382.66_g5334.t2              TPGPAVGVINAMIVGDSITQGFEGDYTWRFRLAEWFKSESITPNFVGPWTGTHTNAEPSDPKPPRLIGSG--PVPPDAPRTGGAYANGVSFKSNHWALWGRQAAQVKNEIRGQVSTHSPD
    CBS382.66_g5795.t1              ------------------------------------------------------------------------------------------------------------------------
    CBS382.66_g6578.t1              ------------------------------------------------------------------------------------------------------------------------
    CBS382.66_g8384.t1              ----------------FLAPEF--------------------------------------------------------------------------------------------------
    CBS382.66_g9605.t1              ------------------------------------------------------------------------------------------------------------------------
    CBS382.66_g9740.t1              ------------------------------------------------------------------------------------------------------------------------
    MUCL9792_g2463.t1               ------------------------------------------------------------------------------------------------------------------------
    MUCL9792_g2802.t1               ------------------------------------------------------------------------------------------------------------------------
    MUCL9792_g3157.t1               ----------------RIAEKF--------------------------------------------------------------------------------------------------
    MUCL9792_g4589.t1               TPGPAVGVINAMIVGDSITQGFEGDYTWRFRLAEWFRSESITPNFKGPWTGTHTNAEPSDPKPPRLIGSG--PAPPDAPRTSGAYANGVSFNSAHFALWGRQAAQVKNEIRGQVSTHSPD
    MUCL9792_g4589.t2               TPGPAVGVINAMIVGDSITQGFEGDYTWRFRLAEWFRSESITPNFKGPWTGTHTNAEPSDPKPPRLIGSG--PAPPDAPRTSGAYANGVSFNSAHFALWGRQAAQVKNEIRGQVSTHSPD
    MUCL9792_g4589.t3               ------------------------------------------------------------------------------------------------------------------------
    MUCL9792_g6332.t1               ------------------------------------------------------------------------------------------------------------------------
    MUCL9792_g7308.t1               ------------------------------------------------------------------------------------------------------------------------
    MUCL9792_g10611.t1              ------------------------------------------------------------------------------------------------------------------------
    PD401_g2361.t1                  ------------------------------------------------------------------------------------------------------------------------
    PD401_g3373.t1                  ------------------------------------------------------------------------------------------------------------------------
    PD401_g4730.t1                  ------------------------------------------------------------------------------------------------------------------------
    PD401_g7339.t1                  ------------------------------------------------------------------------------------------------------------------------
    PD401_g10483.t1                 ------------------------------------------------------------------------------------------------------------------------
    PD593_g749.t1                   TPGPAVGVINAMIVGDSITQGFEGDYTWRFRLAEWFRSESITPNFKGPWTGTHTNAEPSDPKPPRLIGSG--PAPPDAPRTSGAYANGVSFNSAHFALWGRQAAQVKNEIRGQVSTHSPD
    PD593_g2541.t1                  ------------------------------------------------------------------------------------------------------------------------
    PD593_g2728.t1                  ------------------------------------------------------------------------------------------------------------------------
    PD593_g7030.t1                  ------------------------------------------------------------------------------------------------------------------------
    PD593_g8432.t1                  ------------------------------------------------------------------------------------------------------------------------
    PD593_g9596.t1                  ------------------------------------------------------------------------------------------------------------------------
    PD618_g114.t1                   ------------------------------------------------------------------------------------------------------------------------
    PD618_g3974.t1                  ------------------------------------------------------------------------------------------------------------------------
    PD618_g5755.t1                  ------------------------------------------------------------------------------------------------------------------------
    PD618_g9213.t1                  ------------------------------------------------------------------------------------------------------------------------
    PD618_g9942.t1                  ------------------------------------------------------------------------------------------------------------------------
    PD659_g6753.t1                  ------------------------------------------------------------------------------------------------------------------------
    PD659_g7370.t1                  ------------------------------------------------------------------------------------------------------------------------
    PD659_g10785.t1                 ------------------------------------------------------------------------------------------------------------------------
    PD659_g2262.t1                  ------------------------------------------------------------------------------------------------------------------------
    PD659_g5819.t1                  ------------------------------------------------------------------------------------------------------------------------
    PD660_g1229.t1                  ------------------------------------------------------------------------------------------------------------------------
    PD660_g3973.t1                  ------------------------------------------------------------------------------------------------------------------------
    PD660_g6305.t1                  ------------------------------------------------------------------------------------------------------------------------
    PD660_g7826.t1                  ------------------------------------------------------------------------------------------------------------------------
    PD660_g9669.t1                  ------------------------------------------------------------------------------------------------------------------------
    PD660_g10032.t1                 ----------------RIAEKF--------------------------------------------------------------------------------------------------
    PD670_g4670.t1                  ------------------------------------------------------------------------------------------------------------------------
    PD670_g5219.t1                  ------------------------------------------------------------------------------------------------------------------------
    PD670_g7258.t1                  ------------------------------------------------------------------------------------------------------------------------
    PD670_g8696.t1                  ------------------------------------------------------------------------------------------------------------------------
    PD670_g10480.t1                 ------------------------------------------------------------------------------------------------------------------------
    PD670_g10529.t1                 ------------------------------------------------------------------------------------------------------------------------
    PD670_g11082.t1                 ------------------------------------------------------------------------------------------------------------------------
    PD670_g2580.t1                  ----------------WLAPEF--------------------------------------------------------------------------------------------------
    PD670_g3093.t1                  TPGPAVGVINAMIVGDSITQGFEGDYTWRFRLAEWFKSESITPNFKGPWTGTHTNAEPSDPKPPRLIGSG--PAPPDAPRTSGAYANGVSFNSAHFALWGRQAAQVKNEIRGQVSTHSPD
    PD683_g248.t1                   ------------------LKGFPMDW----------------------------------------------------------------------------------------------
    PD683_g3766.t1                  ------------------------------------------------------------------------------------------------------------------------
    PD683_g3895.t1                  ------------------------------------------------------------------------------------------------------------------------
    PD683_g4100.t1                  ------------------------------------------------------------------------------------------------------------------------
    PD683_g5003.t1                  TPGPAVGVINAMIVGDSITQGFEGDYTWRFRLAEWFKSESITPNFVGPWTGTHTNAEPSDPKPPRLIGSG--PVPPDAPRTGGAYANGVSFKSNHWALWGRQAAQVKNEIRGQVSTHSPD
    PD683_g5003.t2                  TPGPAVGVINAMIVGDSITQGFEGDYTWRFRLAEWFKSESITPNFVGPWTGTHTNAEPSDPKPPRLIGSG--PVPPDAPRTGGAYANGVSFKSNHWALWGRQAAQVKNEIRGQVSTHSPD
    PD683_g5713.t1                  ------------------------------------------------------------------------------------------------------------------------
    PD683_g7092.t1                  ------------------------------------------------------------------------------------------------------------------------
    PD683_g8130.t1                  ----------------FLAPEF--------------------------------------------------------------------------------------------------
    PD736_g919.t1                   ------------------------------------------------------------------------------------------------------------------------
    PD736_g8670.t1                  ------------------------------------------------------------------------------------------------------------------------
    PD736_g9411.t1                  ------------------------------------------------------------------------------------------------------------------------
    PD736_g10271.t1                 ------------------------------------------------------------------------------------------------------------------------
    PD736_g10840.t1                 TPGPAVGTIKAMIVGDSITQGFEGDYTWRFRLTEWFKSQSITPHFVGPWTGTHTNAEPSDPKPPRLIGSG--PAPPDGPRTGGAYANGATFNSAHWALWGRQAAQVKSEIRGQVTTHSPD
    PD736_g10939.t1                 ------------------------------------------------------------------------------------------------------------------------
    PD739_g34.t1                    TPGPAVGTIKAMIVGDSITQGFEGDYTWRFRLAEWFKSQSITPNFVGPWTGTHTNAEPSDPKPPRLIGSG--PAPPDAPRTGGAYANGATFNSAHWALWGRQAAQVKSEIRGQVATHSPD
    PD739_g131.t1                   ------------------------------------------------------------------------------------------------------------------------
    PD739_g4443.t1                  ------------------------------------------------------------------------------------------------------------------------
    PD739_g7436.t1                  ------------------------------------------------------------------------------------------------------------------------
    PD739_g8512.t1                  ------------------------------------------------------------------------------------------------------------------------
    PD739_g10815.t1                 ------------------------------------------------------------------------------------------------------------------------
    PD747_g20.t1                    TPGPAVGVINAMIVGDSITQGFEGDYTWRFRLAEWFKSESITPNFKGPWTGTHTNAEPSDPKPPRLIGSG--PAPPDAPRTSGAYANGVSFNSAHFALWGRQAAQVKNEIRGQVSTHSPD
    PD747_g277.t1                   ------------------------------------------------------------------------------------------------------------------------
    PD747_g4174.t1                  ------------------------------------------------------------------------------------------------------------------------
    PD747_g5167.t1                  ------------------------------------------------------------------------------------------------------------------------
    PD747_g9207.t1                  ----------------WLAPEF--------------------------------------------------------------------------------------------------
    PD747_g9751.t1                  ------------------------------------------------------------------------------------------------------------------------
    PD747_g10403.t1                 ------------------------------------------------------------------------------------------------------------------------
    PD747_g10498.t1                 ------------------------------------------------------------------------------------------------------------------------
    PD747_g10549.t1                 ------------------------------------------------------------------------------------------------------------------------
    T2_g2030.t1                     ------------------LKGFPMDW----------------------------------------------------------------------------------------------
    T2_g2030.t2                     ------------------LKGFPMDW----------------------------------------------------------------------------------------------
    T2_g3751.t1                     TPGPAVGVINAMIVGDSITQGFEGDYTWRFRLAEWFKSESITPNFVGPWTGTHTNAEPSDPKPPRLIGSG--PVPPDAPRTGGAYANGVSFKSNHWALWGRQAAQVKNEIRGQVSTHSPD
    T2_g3751.t2                     TPGPAVGVINAMIVGDSITQGFEGDYTWRFRLAEWFKSESITPNFVGPWTGTHTNAEPSDPKPPRLIGSG--PVPPDAPRTGGAYANGVSFKSNHWALWGRQAAQVKNEIRGQVSTHSPD
    T2_g4661.t1                     ------------------------------------------------------------------------------------------------------------------------
    T2_g4868.t1                     ------------------------------------------------------------------------------------------------------------------------
    T2_g5004.t1                     ------------------------------------------------------------------------------------------------------------------------
    T2_g5801.t1                     ------------------------------------------------------------------------------------------------------------------------
    T2_g7120.t1                     ----------------FLAPEF--------------------------------------------------------------------------------------------------
    T2_g7997.t1                     ------------------------------------------------------------------------------------------------------------------------
    VSO1_g539.t1                    ------------------------------------------------------------------------------------------------------------------------
    VSO1_g1387.t1                   ------------------------------------------------------------------------------------------------------------------------
    VSO1_g1731.t1                   ------------------------------------------------------------------------------------------------------------------------
    VSO1_g1937.t1                   ----------------RIAEKF--------------------------------------------------------------------------------------------------
    VSO1_g4972.t1                   ------------------------------------------------------------------------------------------------------------------------
    VSO1_g5184.t1                   ------------------------------------------------------------------------------------------------------------------------
    VL20_g7800.t1                   ------------------LKGFPMDW----------------------------------------------------------------------------------------------
    VL20_g9415.t1                   ------------------------------------------------------------------------------------------------------------------------
    VL20_g11025.t1                  ------------------------------------------------------------------------------------------------------------------------
    VL20_g11172.t1                  ------------------LKGFPMDW----------------------------------------------------------------------------------------------
    VL20_g12965.t1                  ----------------FLAPEF--------------------------------------------------------------------------------------------------
    VL20_g13837.t1                  ------------------------------------------------------------------------------------------------------------------------
    VL20_g14042.t1                  ------------------------------------------------------------------------------------------------------------------------
    VL20_g14171.t1                  ------------------------------------------------------------------------------------------------------------------------
    VL20_g14284.t1                  ------------------------------------------------------------------------------------------------------------------------
    VL20_g17450.t1                  ------------------------------------------------------------------------------------------------------------------------
    VL20_g18857.t1                  ------------------------------------------------------------------------------------------------------------------------
    VL20_g4752.t1                   ------------------------------------------------------------------------------------------------------------------------
    VL20_g5938.t1                   ----------------FLAPEF--------------------------------------------------------------------------------------------------
    VL20_g5938.t2                   ----------------FLAPEF--------------------------------------------------------------------------------------------------
    VL20_g6061.t1                   ------------------------------------------------------------------------------------------------------------------------
    VL20_g18964.t1                  ----------------FLAPEF--------------------------------------------------------------------------------------------------
    VL20_g18964.t2                  ----------------FLAPEF--------------------------------------------------------------------------------------------------
    VLB2_g585.t1                    ------------------LKGFPMDW----------------------------------------------------------------------------------------------
    VLB2_g1761.t1                   ------------------------------------------------------------------------------------------------------------------------
    VLB2_g2033.t1                   ------------------LKGFPMDW----------------------------------------------------------------------------------------------
    VLB2_g12050.t1                  ------------------------------------------------------------------------------------------------------------------------
    VLB2_g12617.t1                  ----------------FLAPEF--------------------------------------------------------------------------------------------------
    VLB2_g13499.t1                  ------------------------------------------------------------------------------------------------------------------------
    VLB2_g13703.t1                  ------------------------------------------------------------------------------------------------------------------------
    VLB2_g13833.t1                  ------------------------------------------------------------------------------------------------------------------------
    VLB2_g14318.t1                  ------------------------------------------------------------------------------------------------------------------------
    VLB2_g15187.t1                  ----------------FLAPEF--------------------------------------------------------------------------------------------------
    VLB2_g15187.t2                  ----------------FLAPEF--------------------------------------------------------------------------------------------------
    VLB2_g16101.t1                  ------------------------------------------------------------------------------------------------------------------------
    VLB2_g16543.t1                  ------------------------------------------------------------------------------------------------------------------------
    VLB2_g19098.t1                  TPGPAVGVINAMIVGDSITQGFEGDYTWRFRLAEWFKSENITPNFVGPWTGTHTNAEPSDPKPPRLIGSG--PVPPDAPRTGGAYANGVSFKSNHWALWGRQAAQVKNEIRGQVSTHSPD
    VLB2_g5814.t1                   ------------------------------------------------------------------------------------------------------------------------
    VLB2_g6534.t1                   ------------------------------------------------------------------------------------------------------------------------
    PD589_g51.t1                    ----------------FLAPEF--------------------------------------------------------------------------------------------------
    PD589_g856.t1                   ------------------------------------------------------------------------------------------------------------------------
    PD589_g7665.t1                  ------------------------------------------------------------------------------------------------------------------------
    PD589_g13197.t1                 ------------------LKGFPMDW----------------------------------------------------------------------------------------------
    PD589_g13425.t1                 ------------------------------------------------------------------------------------------------------------------------
    PD589_g14403.t1                 ------------------------------------------------------------------------------------------------------------------------
    PD589_g14539.t1                 ------------------------------------------------------------------------------------------------------------------------
    PD589_g14661.t1                 ------------------------------------------------------------------------------------------------------------------------
    PD589_g15310.t1                 ------------------------------------------------------------------------------------------------------------------------
    PD589_g16171.t1                 ----------------FLAPEF--------------------------------------------------------------------------------------------------
    PD589_g16171.t2                 ----------------FLAPEF--------------------------------------------------------------------------------------------------
    PD589_g17659.t1                 ------------------------------------------------------------------------------------------------------------------------
    PD589_g18192.t1                 ------------------------------------------------------------------------------------------------------------------------
    PD589_g3050.t1                  ------------------------------------------------------------------------------------------------------------------------
    PD589_g6680.t1                  ------------------LKGFPMDW----------------------------------------------------------------------------------------------
    PD589_g6775.t1                  ------------------------------------------------------------------------------------------------------------------------
    Vt305_g1664.t1                  ------------------------------------------------------------------------------------------------------------------------
    Vt305_g2624.t1                  ------------------------------------------------------------------------------------------------------------------------
    Vt305_g3295.t1                  ------------------------------------------------------------------------------------------------------------------------
    Vt305_g4616.t1                  ------------------------------------------------------------------------------------------------------------------------
    Vt305_g7985.t1                  ------------------------------------------------------------------------------------------------------------------------
    WCS072_g2037.t1                 ------------------LKGFPMDW----------------------------------------------------------------------------------------------
    WCS072_g2037.t2                 ------------------LKGFPMDW----------------------------------------------------------------------------------------------
    WCS072_g3872.t1                 ------------------------------------------------------------------------------------------------------------------------
    WCS072_g4007.t1                 ------------------------------------------------------------------------------------------------------------------------
    WCS072_g4219.t1                 ------------------------------------------------------------------------------------------------------------------------
    WCS072_g5132.t1                 TPGPAVGVINAMIVGDSITQGFEGDYTWRFRLAEWFKSESITPNFVGPWTGTHTNAEPSDPKPPRLIGSG--PVPPDAPRTGGAYANGVSFKSNHWALWGRQAAQVKNEIRGQVSTHSPD
    WCS072_g5132.t2                 TPGPAVGVINAMIVGDSITQGFEGDYTWRFRLAEWFKSESITPNFVGPWTGTHTNAEPSDPKPPRLIGSG--PVPPDAPRTGGAYANGVSFKSNHWALWGRQAAQVKNEIRGQVSTHSPD
    WCS072_g5615.t1                 ------------------------------------------------------------------------------------------------------------------------
    WCS072_g6396.t1                 ------------------------------------------------------------------------------------------------------------------------
    WCS072_g8181.t1                 ----------------FLAPEF--------------------------------------------------------------------------------------------------
    VDAG_JR2_Chr1g22850a-00001      ------------------LKGFPMDW----------------------------------------------------------------------------------------------
    VDAG_JR2_Chr2g05460a-00001      ------------------------------------------------------------------------------------------------------------------------
    VDAG_JR2_Chr6g08770a-00001      ------------------------------------------------------------------------------------------------------------------------
    VDAG_JR2_Chr6g10260a-00001      ------------------------------------------------------------------------------------------------------------------------
    VDAG_JR2_Chr3g00800a-00001      ------------------------------------------------------------------------------------------------------------------------
    VDAG_JR2_Chr4g05950a-00001      ------------------------------------------------------------------------------------------------------------------------
    VDAG_JR2_Chr8g10650a-00001      ----------------FLAPEF--------------------------------------------------------------------------------------------------
    JR2_g2720.t1                    ------------------------------------------------------------------------------------------------------------------------

    Selected Cols:                                                                                                                                          

    Gaps Scores:                                                                                                                                            
    Similarity Scores:                                                                                                                                      

                                           610       620       630       640       650       660       670       680       690       700       710       720
                                    =========+=========+=========+=========+=========+=========+=========+=========+=========+=========+=========+=========+
    12008_g358.t1                   ------------------------------------------------------------------------------------------------------------------------
    12008_g4461.t1                  ------------------------------------------------------------------------------------------------------------------------
    12008_g7340.t1                  ------------------------------------------------------------------------------------------------------------------------
    12008_g7652.t1                  ------------------------------------------------------------------------------------------------------------------------
    12008_g7834.t1                  ------------------------------------------------------------------------------------------------------------------------
    12008_g8465.t1                  ------------------------------------------------------------------------------------------------------------------------
    12008_g8848.t1                  ------------------------------------------------------------------------------------------------------------------------
    85S_g58.t1                      ------------------------------------------------------------------------------------------------------------------------
    85S_g9168.t1                    ------------------------------------------------------------------------------------------------------------------------
    85S_g9863.t1                    ------------------------------------------------------------------------------------------------------------------------
    85S_g7040.t1                    ------------------------------------------------------------------------------------------------------------------------
    85S_g3154.t1                    ------------------------------------------------------------------------------------------------------------------------
    85S_g4608.t1                    ------------------------------------------------------------------------------------------------------------------------
    85S_g4744.t1                    ------------------------------------------------------------------------------------------------------------------------
    CBS385.49_g66.t1                ------------------------------------------------------------------------------------------------------------------------
    CBS385.49_g189.t1               ------------------------------------------------------------------------------------------------------------------------
    CBS385.49_g400.t1               ------------------------------------------------------------------------------------------------------------------------
    CBS385.49_g400.t2               ------------------------------------------------------------------------------------------------------------------------
    CBS385.49_g1381.t1              ------------------------------------------------------------------------------------------------------------------------
    CBS385.49_g5607.t1              ------------------------------------------------------------------------------------------------------------------------
    CBS385.49_g7723.t1              ------------------------------------------------------------------------------------------------------------------------
    CBS385.49_g8721.t1              ------------------------------------------------------------------------------------------------------------------------
    CQ2_g254.t1                     ------------------------------------------------------------------------------------------------------------------------
    CQ2_g2332.t3                    --------------------KLLSVKAIVDEARAAKSNVRILIANVIHRTFIEGRNDL--------------------------------------------------------------
    CQ2_g2332.t4                    ------------------------------------------------------------------------------------------------------------------------
    CQ2_g3243.t1                    ------------------------------------------------------------------------------------------------------------------------
    CQ2_g3454.t1                    ------------------------------------------------------------------------------------------------------------------------
    CQ2_g3584.t1                    ------------------------------------------------------------------------------------------------------------------------
    CQ2_g4742.t1                    ------------------------------------------------------------------------------------------------------------------------
    CQ2_g7512.t1                    ------------------------------------------------------------------------------------------------------------------------
    CQ2_g8820.t1                    ------------------------------------------------------------------------------------------------------------------------
    DAR82592_g1639.t1               ------------------------------------------------------------------------------------------------------------------------
    DAR82592_g8837.t1               ------------------------------------------------------------------------------------------------------------------------
    DAR82592_g4435.t1               ------------------------------------------------------------------------------------------------------------------------
    DAR82592_g4647.t1               ------------------------------------------------------------------------------------------------------------------------
    DAR82592_g7268.t1               ------------------------------------------------------------------------------------------------------------------------
    DAR82592_g4307.t1               ------------------------------------------------------------------------------------------------------------------------
    DAR82592_g3508.t1               ------------------------------------------------------------------------------------------------------------------------
    DAR82592_g62.t1                 ------------------------------------------------------------------------------------------------------------------------
    DAR83138_g1199.t1               ------------------------------------------------------------------------------------------------------------------------
    DAR83138_g60.t1                 ------------------------------------------------------------------------------------------------------------------------
    DAR83138_g7641.t1               ------------------------------------------------------------------------------------------------------------------------
    DAR83138_g9085.t1               ------------------------------------------------------------------------------------------------------------------------
    DAR83138_g3565.t3               --------------------KLLSVKAIVDEARAAKSNVRILIANVIHRTFIEGRNDL--------------------------------------------------------------
    DAR83138_g4173.t1               ------------------------------------------------------------------------------------------------------------------------
    DAR83138_g4314.t1               ------------------------------------------------------------------------------------------------------------------------
    DAR83138_g4535.t1               ------------------------------------------------------------------------------------------------------------------------
    DAR83143_g1427.t1               ------------------------------------------------------------------------------------------------------------------------
    DAR83143_g4194.t1               ------------------------------------------------------------------------------------------------------------------------
    DAR83143_g4335.t1               ------------------------------------------------------------------------------------------------------------------------
    DAR83143_g4545.t1               ------------------------------------------------------------------------------------------------------------------------
    DAR83143_g7287.t1               ------------------------------------------------------------------------------------------------------------------------
    DAR83143_g3521.t1               ----------------------------------------------VSRTI---------------------------------------------------------------------
    DAR83143_g8715.t1               ------------------------------------------------------------------------------------------------------------------------
    DAR83143_g61.t1                 ------------------------------------------------------------------------------------------------------------------------
    DAR83175_g3551.t1               ------------------------------------------------------------------------------------------------------------------------
    DAR83175_g4514.t1               ------------------------------------------------------------------------------------------------------------------------
    DAR83175_g4731.t1               ------------------------------------------------------------------------------------------------------------------------
    DAR83175_g7439.t1               ------------------------------------------------------------------------------------------------------------------------
    DAR83175_g2075.t1               ------------------------------------------------------------------------------------------------------------------------
    DAR83175_g5817.t1               ------------------------------------------------------------------------------------------------------------------------
    DAR83175_g61.t1                 ------------------------------------------------------------------------------------------------------------------------
    DAR83175_g8890.t1               ------------------------------------------------------------------------------------------------------------------------
    DK045_g927.t1                   ------------------------------------------------------------------------------------------------------------------------
    DK045_g1138.t1                  ------------------------------------------------------------------------------------------------------------------------
    DK045_g1272.t1                  ------------------------------------------------------------------------------------------------------------------------
    DK045_g2451.t1                  ------------------------------------------------------------------------------------------------------------------------
    DK045_g3667.t1                  ------------------------------------------------------------------------------------------------------------------------
    DK045_g5985.t1                  ------------------------------------------------------------------------------------------------------------------------
    DK045_g9032.t1                  ------------------------------------------------------------------------------------------------------------------------
    GF1192_g8987.t1                 ------------------------------------------------------------------------------------------------------------------------
    GF1192_g9126.t1                 ------------------------------------------------------------------------------------------------------------------------
    GF1192_g257.t1                  ------------------------------------------------------------------------------------------------------------------------
    GF1192_g1728.t1                 ------------------------------------------------------------------------------------------------------------------------
    GF1192_g2840.t1                 ------------------------------------------------------------------------------------------------------------------------
    GF1192_g5025.t1                 ------------------------------------------------------------------------------------------------------------------------
    GF1192_g6069.t1                 ------------------------------------------------------------------------------------------------------------------------
    GF1207_g2093.t1                 ------------------------------------------------------------------------------------------------------------------------
    GF1207_g4890.t1                 ------------------------------------------------------------------------------------------------------------------------
    GF1207_g5214.t1                 ------------------------------------------------------------------------------------------------------------------------
    GF1207_g6669.t1                 ------------------------------------------------------------------------------------------------------------------------
    GF1207_g6876.t1                 ------------------------------------------------------------------------------------------------------------------------
    GF1207_g6999.t1                 ------------------------------------------------------------------------------------------------------------------------
    GF1207_g7831.t1                 ------------------------------------------------------------------------------------------------------------------------
    GF1300_g9576.t1                 ------------------------------------------------------------------------------------------------------------------------
    GF1300_g9714.t1                 ------------------------------------------------------------------------------------------------------------------------
    GF1300_g259.t1                  ------------------------------------------------------------------------------------------------------------------------
    GF1300_g1512.t1                 ------------------------------------------------------------------------------------------------------------------------
    GF1300_g3582.t1                 ------------------------------------------------------------------------------------------------------------------------
    GF1300_g3720.t1                 ------------------------------------------------------------------------------------------------------------------------
    GF1300_g6171.t1                 ------------------------------------------------------------------------------------------------------------------------
    Gf-Ca2_g111.t1                  ------------------------------------------------------------------------------------------------------------------------
    Gf-Ca2_g2398.t1                 ------------------------------------------------------------------------------------------------------------------------
    Gf-Ca2_g3362.t1                 ------------------------------------------------------------------------------------------------------------------------
    Gf-Ca2_g3481.t1                 ------------------------------------------------------------------------------------------------------------------------
    Gf-Ca2_g3687.t1                 ------------------------------------------------------------------------------------------------------------------------
    Gf-Ca2_g4661.t1                 ------------------------------------------------------------------------------------------------------------------------
    Gf-Ca2_g8346.t1                 ------------------------------------------------------------------------------------------------------------------------
    Gf-Cb5_g260.t1                  ------------------------------------------------------------------------------------------------------------------------
    Gf-Cb5_g3949.t1                 ------------------------------------------------------------------------------------------------------------------------
    Gf-Cb5_g4084.t1                 ------------------------------------------------------------------------------------------------------------------------
    Gf-Cb5_g4605.t1                 ------------------------------------------------------------------------------------------------------------------------
    Gf-Cb5_g6249.t1                 -----------------------------------------LLLVAVSRTI---------------------------------------------------------------------
    Gf-Cb5_g7167.t1                 ------------------------------------------------------------------------------------------------------------------------
    Gf-Cb5_g7934.t1                 ------------------------------------------------------------------------------------------------------------------------
    Gf-Cb5_g8589.t1                 ------------------------------------------------------------------------------------------------------------------------
    HoMCF_g1490.t1                  ------------------------------------------------------------------------------------------------------------------------
    HoMCF_g2875.t1                  ------------------------------------------------------------------------------------------------------------------------
    HoMCF_g3274.t1                  ------------------------------------------------------------------------------------------------------------------------
    HoMCF_g4303.t1                  ------------------------------------------------------------------------------------------------------------------------
    HoMCF_g5967.t1                  ------------------------------------------------------------------------------------------------------------------------
    HoMCF_g6093.t1                  ------------------------------------------------------------------------------------------------------------------------
    HoMCF_g6096.t1                  ------------------------------------------------------------------------------------------------------------------------
    HoMCF_g6549.t1                  ------------------------------------------------------------------------------------------------------------------------
    HoMCF_g10173.t1                 ------------------------------------------------------------------------------------------------------------------------
    HoMCLT_g1190.t1                 ------------------------------------------------------------------------------------------------------------------------
    HoMCLT_g2195.t1                 ------------------------------------------------------------------------------------------------------------------------
    HoMCLT_g2713.t1                 ------------------------------------------------------------------------------------------------------------------------
    HoMCLT_g3698.t1                 ------------------------------------------------------------------------------------------------------------------------
    HoMCLT_g4981.t1                 ------------------------------------------------------------------------------------------------------------------------
    HoMCLT_g6335.t1                 ------------------------------------------------------------------------------------------------------------------------
    HoMCLT_g6471.t1                 ------------------------------------------------------------------------------------------------------------------------
    I1V_g7945.t1                    ------------------------------------------------------------------------------------------------------------------------
    I1V_g9550.t1                    ------------------------------------------------------------------------------------------------------------------------
    I1V_g9695.t1                    ------------------------------------------------------------------------------------------------------------------------
    I1V_g10783.t1                   ------------------------------------------------------------------------------------------------------------------------
    I1V_g12440.t1                   ------------------------------------------------------------------------------------------------------------------------
    I1V_g13776.t1                   ------------------------------------------------------------------------------------------------------------------------
    MPI-CAGE_g5591.t1               ------------------------------------------------------------------------------------------------------------------------
    MPI-CAGE_g8268.t1               ------------------------------------------------------------------------------------------------------------------------
    MPI-CAGE_g8414.t1               ------------------------------------------------------------------------------------------------------------------------
    MPI-CAGE_g9956.t1               ------------------------------------------------------------------------------------------------------------------------
    MPI-CAGE_g1577.t1               ------------------------------------------------------------------------------------------------------------------------
    MPI-CAGE_g2204.t1               WLLVMLGFNDLGWWVGGPDDTLASVKAIVDEARAAKSNVRILIANVIHRTFIEGRNDL--------------------------------------------------------------
    MPI-CAGE_g10572.t1              ------------------------------------------------------------------------------------------------------------------------
    MPI-CAGE_g3967.t1               ------------------------------------------------------------------------------------------------------------------------
    MPI-CAGE_g4142.t1               ------------------------------------------------------------------------------------------------------------------------
    S011_g7486.t1                   ------------------------------------------------------------------------------------------------------------------------
    S011_g8092.t1                   ------------------------------------------------------------------------------------------------------------------------
    S011_g8657.t1                   ------------------------------------------------------------------------------------------------------------------------
    S011_g8986.t1                   ------------------------------------------------------------------------------------------------------------------------
    S011_g9203.t1                   ------------------------------------------------------------------------------------------------------------------------
    S011_g1207.t1                   ------------------------------------------------------------------------------------------------------------------------
    S011_g2838.t1                   ------------------------------------------------------------------------------------------------------------------------
    S011_g5817.t1                   ------------------------------------------------------------------------------------------------------------------------
    S023_g1908.t1                   ------------------------------------------------------------------------------------------------------------------------
    S023_g3148.t1                   ------------------------------------------------------------------------------------------------------------------------
    S023_g4559.t1                   ------------------------------------------------------------------------------------------------------------------------
    S023_g5760.t1                   ------------------------------------------------------------------------------------------------------------------------
    S023_g5904.t1                   ------------------------------------------------------------------------------------------------------------------------
    S023_g7666.t1                   ------------------------------------------------------------------------------------------------------------------------
    S023_g9104.t1                   ------------------------------------------------------------------------------------------------------------------------
    TO22_g2077.t1                   ------------------------------------------------------------------------------------------------------------------------
    TO22_g2405.t1                   ------------------------------------------------------------------------------------------------------------------------
    TO22_g2541.t1                   ------------------------------------------------------------------------------------------------------------------------
    TO22_g2749.t1                   ------------------------------------------------------------------------------------------------------------------------
    TO22_g3776.t1                   ------------------------------------------------------------------------------------------------------------------------
    TO22_g6379.t1                   ------------------------------------------------------------------------------------------------------------------------
    TO22_g9457.t1                   ------------------------------------------------------------------------------------------------------------------------
    Ud1-4-1_g9889.t1                ------------------------------------------------------------------------------------------------------------------------
    Ud1-4-1_g261.t1                 ------------------------------------------------------------------------------------------------------------------------
    Ud1-4-1_g2536.t1                ------------------------------------------------------------------------------------------------------------------------
    Ud1-4-1_g2721.t1                ------------------------------------------------------------------------------------------------------------------------
    Ud1-4-1_g2858.t1                ------------------------------------------------------------------------------------------------------------------------
    Ud1-4-1_g3064.t1                ------------------------------------------------------------------------------------------------------------------------
    Ud1-4-1_g3975.t1                ----------------------------------------------VSRTI---------------------------------------------------------------------
    Ud1-4-1_g4565.t1                ------------------------------------------------------------------------------------------------------------------------
    V13_g3278.t1                    ------------------------------------------------------------------------------------------------------------------------
    V13_g3397.t1                    ------------------------------------------------------------------------------------------------------------------------
    V13_g4590.t1                    ------------------------------------------------------------------------------------------------------------------------
    V13_g5067.t1                    ------------------------------------------------------------------------------------------------------------------------
    V13_g6708.t1                    ------------------------------------------------------------------------------------------------------------------------
    V13_g9344.t1                    ----------------------------------------------VSRTI---------------------------------------------------------------------
    V13_g9739.t1                    ------------------------------------------------------------------------------------------------------------------------
    V13_g10958.t1                   ------------------------------------------------------------------------------------------------------------------------
    Vd39_g2112.t1                   WLLVMLGFNDLGWWVGGPDDTLASVKAIVDEARAAKSNVRILIANVIPRTFIEGRNDL--------------------------------------------------------------
    Vd39_g2112.t3                   ------------------------------------------------------------------------------------------------------------------------
    Vd39_g2716.t1                   ------------------------------------------------------------------------------------------------------------------------
    Vd39_g3506.t1                   ------------------------------------------------------------------------------------------------------------------------
    Vd39_g3907.t1                   ------------------------------------------------------------------------------------------------------------------------
    Vd39_g4379.t1                   ------------------------------------------------------------------------------------------------------------------------
    Vd39_g7329.t1                   ------------------------------------------------------------------------------------------------------------------------
    Vd39_g8964.t1                   ------------------------------------------------------------------------------------------------------------------------
    Vd39_g9298.t1                   ------------------------------------------------------------------------------------------------------------------------
    Vd-653_g1291.t1                 ------------------------------------------------------------------------------------------------------------------------
    Vd-653_g2557.t1                 ------------------------------------------------------------------------------------------------------------------------
    Vd-653_g3339.t1                 ------------------------------------------------------------------------------------------------------------------------
    Vd-653_g3685.t1                 ------------------------------------------------------------------------------------------------------------------------
    Vd-653_g3686.t1                 ------------------------------------------------------------------------------------------------------------------------
    Vd-653_g5777.t1                 ------------------------------------------------------------------------------------------------------------------------
    Vd-653_g8550.t1                 ------------------------------------------------------------------------------------------------------------------------
    Vd-653_g9566.t1                 ------------------------------------------------------------------------------------------------------------------------
    VD991_g2070.t1                  ------------------------------------------------------------------------------------------------------------------------
    VD991_g2394.t1                  ------------------------------------------------------------------------------------------------------------------------
    VD991_g2531.t1                  ------------------------------------------------------------------------------------------------------------------------
    VD991_g2742.t1                  ------------------------------------------------------------------------------------------------------------------------
    VD991_g3653.t1                  --------------------KLLSVKAIVDEARAAKSNVRILIANVIHRTFIEGRNDL--------------------------------------------------------------
    VD991_g3653.t2                  --------------------KLLSVKAIVDEARAAKSNVRILIANVIHRTFIEGRNDL-------------------------------ERMMCSSPGSHSSAYDGLHPGPKGEYQIAHA
    VD991_g3719.t1                  ------------------------------------------------------------------------------------------------------------------------
    VD991_g7479.t1                  ------------------------------------------------------------------------------------------------------------------------
    VD991_g8969.t1                  ------------------------------------------------------------------------------------------------------------------------
    VdB09_g1055.t1                  ------------------------------------------------------------------------------------------------------------------------
    VdB09_g1685.t1                  ------------------------------------------------------------------------------------------------------------------------
    VdB09_g1751.t1                  ------------------------------------------------------------------------------------------------------------------------
    VdB09_g3918.t1                  ------------------------------------------------------------------------------------------------------------------------
    VdB09_g4877.t1                  ------------------------------------------------------------------------------------------------------------------------
    VdB09_g5096.t1                  ------------------------------------------------------------------------------------------------------------------------
    VdB09_g9703.t1                  ------------------------------------------------------------------------------------------------------------------------
    VdB09_g7661.t1                  ------------------------------------------------------------------------------------------------------------------------
    VdC07_g4126.t1                  ------------------------------------------------------------------------------------------------------------------------
    VdC07_g4261.t1                  ------------------------------------------------------------------------------------------------------------------------
    VdC07_g4482.t1                  ------------------------------------------------------------------------------------------------------------------------
    VdC07_g605.t1                   ------------------------------------------------------------------------------------------------------------------------
    VdC07_g1872.t1                  ------------------------------------------------------------------------------------------------------------------------
    VdC07_g2986.t1                  ------------------------------------------------------------------------------------------------------------------------
    VdC07_g7672.t1                  ------------------------------------------------------------------------------------------------------------------------
    Vd-H5_g2477.t1                  ------------------------------------------------------------------------------------------------------------------------
    Vd-H5_g5176.t1                  ------------------------------------------------------------------------------------------------------------------------
    Vd-H5_g6144.t1                  ------------------------------------------------------------------------------------------------------------------------
    Vd-H5_g7203.t1                  ------------------------------------------------------------------------------------------------------------------------
    Vd-H5_g8423.t1                  ------------------------------------------------------------------------------------------------------------------------
    Vd-H5_g8546.t1                  ------------------------------------------------------------------------------------------------------------------------
    Vd-H5_g9396.t1                  ------------------------------------------------------------------------------------------------------------------------
    VdLs16_g2071.t1                 ------------------------------------------------------------------------------------------------------------------------
    VdLs16_g7836.t1                 ------------------------------------------------------------------------------------------------------------------------
    VdLs16_g8455.t1                 ------------------------------------------------------------------------------------------------------------------------
    VdLs16_g5349.t1                 ------------------------------------------------------------------------------------------------------------------------
    VdLs16_g5560.t1                 ------------------------------------------------------------------------------------------------------------------------
    VdLs16_g5699.t1                 ------------------------------------------------------------------------------------------------------------------------
    VdLs16_g6952.t1                 ------------------------------------------------------------------------------------------------------------------------
    VdLs17_g64.t1                   ------------------------------------------------------------------------------------------------------------------------
    VdLs17_g1975.t1                 ------------------------------------------------------------------------------------------------------------------------
    VdLs17_g3499.t1                 ----------------------------------------------VSRTI---------------------------------------------------------------------
    VdLs17_g4253.t1                 ------------------------------------------------------------------------------------------------------------------------
    VdLs17_g4391.t1                 ------------------------------------------------------------------------------------------------------------------------
    VdLs17_g5246.t1                 ------------------------------------------------------------------------------------------------------------------------
    VdLs17_g6770.t1                 ------------------------------------------------------------------------------------------------------------------------
    VdLs17_g8273.t1                 ------------------------------------------------------------------------------------------------------------------------
    VdLs17_g8809.t1                 ------------------------------------------------------------------------------------------------------------------------
    Vd-R1_g926.t1                   ------------------------------------------------------------------------------------------------------------------------
    Vd-R1_g3679.t1                  ------------------------------------------------------------------------------------------------------------------------
    Vd-R1_g4718.t1                  ------------------------------------------------------------------------------------------------------------------------
    Vd-R1_g5037.t1                  ------------------------------------------------------------------------------------------------------------------------
    Vd-R1_g8886.t1                  ------------------------------------------------------------------------------------------------------------------------
    Vd-R1_g9491.t1                  ------------------------------------------------------------------------------------------------------------------------
    Vd-R1_g9617.t1                  ------------------------------------------------------------------------------------------------------------------------
    VT-2A_g1161.t1                  ------------------------------------------------------------------------------------------------------------------------
    VT-2A_g1267.t1                  ------------------------------------------------------------------------------------------------------------------------
    VT-2A_g4726.t1                  ------------------------------------------------------------------------------------------------------------------------
    VT-2A_g6128.t1                  ------------------------------------------------------------------------------------------------------------------------
    VT-2A_g6128.t2                  ------------------------------------------------------------------------------------------------------------------------
    VT-2A_g6341.t1                  ------------------------------------------------------------------------------------------------------------------------
    VT-2A_g6470.t1                  ------------------------------------------------------------------------------------------------------------------------
    VT-2A_g9510.t1                  ------------------------------------------------------------------------------------------------------------------------
    VT-2A_g9511.t1                  ------------------------------------------------------------------------------------------------------------------------
    XJ511_g1085.t1                  ------------------------------------------------------------------------------------------------------------------------
    XJ511_g1518.t1                  ------------------------------------------------------------------------------------------------------------------------
    XJ511_g4592.t1                  ------------------------------------------------------------------------------------------------------------------------
    XJ511_g5794.t1                  ------------------------------------------------------------------------------------------------------------------------
    XJ511_g5932.t1                  ------------------------------------------------------------------------------------------------------------------------
    XJ511_g7291.t1                  ------------------------------------------------------------------------------------------------------------------------
    XJ511_g8237.t1                  ------------------------------------------------------------------------------------------------------------------------
    XJ592_g261.t1                   ------------------------------------------------------------------------------------------------------------------------
[truncated: 223,256 more chars]
